# Allergic-related skin diseases: Global disease burden from 1990 to 2021 and future trends^☆^

**DOI:** 10.1016/j.waojou.2025.101072

**Published:** 2025-06-03

**Authors:** Xiao Tang, Li Lin, Fangning Yu, Yizhao Ma, Zeyu Liu, Xuying Xu

**Affiliations:** Beijing Hospital of Traditional Chinese Medicine, Capital Medical University, Beijing 100010, China

**Keywords:** Allergic-related skin diseases, Atopic dermatitis, Contact dermatitis, Urticaria, Global burden of disease

## Abstract

**Background:**

Allergic-related skin diseases, including atopic dermatitis (AD), urticaria, and contact dermatitis (CD), are significant global public health challenges. Currently, there is a lack of systematic analysis of allergic-related skin diseases globally.

**Methods:**

This study aimed to quantify the global burden of AD, CD, and urticaria and evaluate their global epidemiology patterns. The Global Burden of Diseases (GBD) database was used to assess incidence, prevalence, and disability-adjusted life years (DALYs) for these allergic-related skin diseases. Additionally, the Bayesian Age-Period-Cohort (BAPC) model was employed to predict disease burden for the next 15 years.

**Results:**

From 1990 to 2021, cases of AD, CD, and urticaria rose steadily. In 2021, AD prevalence reached 129 million, a 20.02% increase from 1990. However, average annual percentage change (AAPC) values for the age-standardized prevalence rate (ASPR) of AD declined constantly (AAPC = −0.28). CD had the highest incidence, with 253 million new cases in 2021, though AAPC for ASPR of CD showed minimal changes. AD and urticaria peaked in early life, while CD peaked at ages 75–79. Moreover, AD had the strongest positive correlation with the Socio-demographic Index (SDI) (*p* = 2.2e-16, ρ = 0.626). AD, CD, and urticaria show the highest age-standardized rate in high, middle, and low-middle SDI regions, respectively, with all 3 conditions declining in high SDI. Health inequality analysis showed AD's burden is now more evenly distributed across SDI groups, while the global burden gap for urticaria and CD change limitedly.

**Conclusion:**

Although the global disease burden of allergic-related skin diseases continues to rise, the overall age-standardized rates of AD have steadily declined and are projected to decrease further. In contrast, CD and urticaria require increased attention.

## Introduction

Allergic-related diseases are a serious and persistent public health issue worldwide, imposing a significant disease burden on various regions.^1^^,^^2^ As an important component of allergic diseases, allergic-related skin diseases mainly consist of atopic dermatitis (AD), urticaria, and contact dermatitis (CD). These diseases are often precursors to other allergic conditions like asthma and food allergies.^3^^,^^4^ These diseases lead to significant productivity loss and a marked reduction in quality of life of patients. In general, allergic-related skin diseases are triggered by exposure to specific allergens, causing immune dysregulation driven by mast cells, eosinophil infiltration, and basophil degranulation. This process releases histamine and other inflammatory mediators, such as leukotrienes, prostaglandins, cytokines, chemokines, and platelet-activating factors, resulting in damage to the skin barrier.^5^, ^6^, ^7^ These inflammatory mediators increase vascular permeability and promote the recruitment and activation of inflammatory cells, leading to various skin lesions such as wheals, angioedema, erythema, swelling, rashes, and blisters.^8^ Previous epidemiological surveys have shown that approximately 13% of children and 5% of adults suffer from AD,^9^ the lifetime prevalence of urticaria is about 20%.^10^ Self-reported lifetime prevalence of CD is 15%, with a medically confirmed rate of around 8.3%.^11^ Generally, the CD is divided into different subtypes, and allergic contact dermatitis is one of the main subtypes mediated by immune mechanisms. The academic community believes that CD belongs to a type of allergic-related skin disease.^12^^,^^13^ Although allergic-related skin diseases are rarely life-threatening, their recurrent nature and persistent symptoms pose a significant global public health burden.

Previous studies have provided limited reports on certain allergic diseases, such as asthma^14^ or single types of allergic skin diseases.^15^ However, the Global Burden of Disease (GBD) Collaborator Network has not yet published a comprehensive analysis covering multiple types of allergic-related skin diseases. Therefore, understanding the disease burden caused by various types of allergic-related skin diseases, as well as the changes across spatial, year, age, gender, and the socio-demographic index (SDI) stratifications, is essential for shaping health policies and determining future clinical priorities.

This study aims to analyze the incidence, prevalence, and disability-adjusted life years (DALYs) of allergic-related skin diseases globally, regionally, and nationally from 1990 to 2021. It also describes the age-standardized incidence rates (ASIR), age-standardized prevalence rates (ASPR), and disability-adjusted life year (DALY) rates across countries/regions with different SDI levels. The study explores distributional differences between regions using frontier and health inequality analysis. Lastly, the Bayesian Age-Period-Cohort (BAPC) model was applied to predict the incidence of allergic-related skin diseases over the next 15 years.

## Methods

### Data sources

The GBD research team estimated the disease burden by executing a Bayesian meta-regression model using DisMod-MR 2.1 software, collecting data from 204 countries and regions, across 21 areas, to determine the incidence, prevalence, and DALYs for 371 diseases, as well as their rates, numbers, and percentage changes.^16^ The data used in this study are available from: https://vizhub.healthdata.org/gbd-results/. The SDI represents the overall level of social and economic conditions related to health in each region. The SDI score is derived from a composite of the total fertility rate for women under 25, average years of education for individuals aged 15 and older, and per capita income divided into high, upper-middle, middle, lower-middle, and low SDI categories.^16^^,^^17^ The University of Washington's Institutional Review Board approved the GBD 2021 study, and informed consent was waived due to the use of de-identified data. Our study adheres to the STROCSS reporting standards.^18^

### Case definition

This study included 3 types of allergic-related skin diseases: atopic dermatitis, urticaria, and contact dermatitis. The classification follows the disease codes from the International Classification of Diseases (ICD). In the 10th revision (ICD-10), the disease codes for AD, urticaria, and CD are L20, L50, and L23-L25 (coding source: https://icd.who.int/browse10/2019/en).

### Statistical analysis

To improve the comparability of statistical indicators, the study applied age-specific weighting to crude rates, resulting in age-standardized rates (ASR). The formula for calculating ASR is:

$$ASR=\frac{\sum_{i=1}^{N}\alpha_{i}\omega_{i}}{\sum_{i=1}^{N}\omega_{i}}$$

Where $\alpha_{i}$ the age-specific rate in the ith age group, and $\omega_{i}$ represents the number of persons (or weight) in the same age group within the GBD 2021 standard population. N is the number of age groups.

We used the estimated annual percentage change (EAPC) to reflect the trend of the ASR over a specific period. EAPC is derived from a regression model that assumes a linear relationship between the natural logarithm of ASR and time, i.e., y=α+βx+ε.Here, y=ln(ASR), x= *calendar year*, ε=errorterm, and β represents the positive or negative trend of ASR. EAPC=100×(exp(β)−1), with the 95% confidence interval obtained from the linear model.^19^ If both EAPC and the lower bound of its confidence interval are greater than 0, the ASR shows an increasing trend; if both are less than 0, the ASR shows a decreasing trend.

#### Joinpoint regression model

A joinpoint regression model, based on linear statistical methods, was employed to analyze temporal trends in disease burden. Inflection points in the trends were determined by minimizing the residual sum of squares between the estimated and observed values.^20^ Joinpoint software (Version 5.0.2, National Cancer Institute, USA) was used for this analysis. We calculated the average annual percentage change (AAPC), annual percentage change (APC), and 95% confidence intervals (CI) to analyze trend fluctuations, with statistical significance set at *p* < 0.05^21^^,^^22^.

#### Frontier analysis

To assess the relationship between the DALYs rate of allergic-related skin diseases and SDI, frontier analysis was applied to determine the minimum achievable ASDR at different levels of SDI. This method allows us to calculate the theoretically achievable minimum ASDR for each country based on its SDI.^23^ In the analysis, the lowest values for each nation or region were identified, and these frontier points were connected with horizontal and vertical lines to ensure all data points fell within the boundaries. To account for random fluctuations within the frontier lines, researchers used 100 bootstrap samples, each consisting of locations randomly selected and resampled from the GBD database.^23^ The best practice boundary represents the minimum disease burden that can be achieved at a given SDI level. If a country's actual disease burden exceeds the level achievable on the “frontier” this suggests room for improvement.

#### Cross-country inequality analysis

This study utilized the Concentration Index (CI) and Slope Index of Inequality (SII) to measure cross-country health inequality.^24^ SII represents the slope of a regression line that links the country-level ASDR to the weighted rank of each country. Due to heteroscedasticity, researchers applied robust regression with repeated iterative weighting. When the SII is positive, the burden is concentrated in higher SDI countries; when negative, it shifts to lower SDI countries. Larger absolute values reflect greater levels of inequality. CI reflects differences in health burdens between countries with varying SDI levels. CI is calculated by numerically integrating the area under the Lorenz concentration curve, ranging from −1 to 1. A negative CI indicates that the burden of disease is more concentrated in countries with lower SDI, whereas a positive CI indicates the opposite.^25^

#### Bayesian age-period-cohort (BAPC) model

The BAPC model, using default parameters, was employed to examine the multiplicative effects of age, period, and cohort.^26^^,^^27^ The model was applied using the BAPC and Integrated Nested Laplace Approximation (INLA) R packages to predict the global burden of allergic-related skin diseases over the next 15 years.

Spearman's rank correlation coefficient was used to validate the correlation between ASR and SDI. The statistical analysis was conducted using R software (version 4.3.0, R Foundation for Statistical Computing, Vienna, Austria, https://cran.r-project.org).

## Results

### Overview of the epidemiology of allergic-related skin diseases

#### Prevalence, incidence, and DALYs data of allergic-related skin diseases from 1990 to 2021

Between 1990 and 2021, the incidence, prevalence, and DALYs for AD increased steadily across all age groups. By 2021, AD prevalence had reached 129,018,669 cases (95% UI: 123,873,853 to 134,154,920). Fortunately, during 1990–2021, the ASPR [1728.51 (95%UI: 1658.51, 1798.60) vs 1885.43 (95% UI: 1808.98, 1962.25, EAPC = −0.27), the ASIR [220.58 (95%UI: 209.51,231.97) vs 234.78 (95% UI: 223.02, 247.07), EAPC = −0.20], and the ASDR [75.45 (95% UI: 38.75, 125.64) vs 82.13 (95% UI: 42.35, 136.74), EAPC = −0.26] of AD showed varying degrees of decline (Table 1).

**Table 1:** All-age cases and age-standardized rates (per 100,000 people) for prevalence, incidence, and DALYs of 3 allergic-related skin diseases in 1990 and 2021

Measure	All-ages cases (95% UI)		Age-standardized rate (per 100,000 people) (95% UI)		EAPC (95% CI)
	1990	2021	1990	2021	
**Atopic dermatitis**					
Prevalence	107,497,662 (103,011,046, 112,077,005)	129,018,669 (123,873,853, 134,154,920)	1885.43 (1808.98, 1962.25)	1728.51 (1658.51, 1798.6)	−0.27 (−0.28, −0.27)
Incidence	13,479,212 (12,764,380, 14,220,772)	16,006,681 (15,211,260, 16,819,872)	234.78 (223.02, 247.07)	220.58 (209.51, 231.97)	−0.20 (-0.21, −0.19)
DALYs	4,697,975 (2,421,178, 7,825,813)	5,621,508 (2,889,266, 9,367,135)	82.13 (42.35, 136.74)	75.45 (38.75, 125.64)	−0.26 (−0.27, −0.26)
**Urticaria**					
Prevalence	47,949,600 (42,267,752, 54,859,774)	66,483,473 (59,240,738, 74,912,351)	865.85 (768.81, 980.76)	868.18 (770.06, 983.86)	0.01 (0.01, 0.02)
Incidence	84,870,288 (74,471,430, 96,206,256)	117,014,587 (104,106,688, 131,017,656)	1529.24 (1355.9, 1720.36)	1533.71 (1358.35, 1726.07)	0.01 (0.01, 0.02)
DALYs	2,883,966 (1,901,101, 4,148,607)	3,980,786 (2,616,526, 5,658,917)	51.86 (34.25, 74)	52.11 (34.21, 74.63)	0.02 (0.02, 0.03)
**Contact dermatitis**					
Prevalence	54,303,939 (44,039,670, 66,799,442)	92,257,646 (75,114,487, 112,189,459)	1123.85 (915.13, 1366.67)	1103.56 (896.89, 1345.42)	0.00 (−0.02, 0.03)
Incidence	146,038,363 (116,708,049, 181,677,341)	253,304,736 (204,699,260, 316,547,750)	3045.09 (2456.54, 3783.71)	3020.33 (2438.77, 3749.1)	0.02 (0.01, 0.04)
DALYs	1,347,229 (826,744, 2,037,567)	2,272,766 (1,395,454, 3,462,432)	27.73 (16.98, 41.73)	27.22 (16.65, 41.23)	0.01 (−0.01, 0.03)

In the meanwhile, the global prevalence of urticaria reached 66,483,473 cases (95% UI: 59,240,738 to 74,912,351), with an ASPR of 868.18 (95% UI: 770.06 to 983.86) per 100,000 people in 2021 (Table 1). For CD, the number of new incidence cases reached an astonishing 253,304,736 (95% UI: 204,699,260 to 316,547,750), with an ASIR of 3020.33 (95% UI: 2438.77 to 3749.10) in 2021 (Table 1). The ASR for both urticaria and CD showed limited variation, indicating that the long-term disease burden has not significantly decreased. The study also found that the prevalence of urticaria and CD was lower than their incidence. This discrepancy may be attributed to classifying both conditions into acute and chronic phases, where the acute phase is characterized by a shorter duration.^28^ Additionally, allergic-related diseases often exhibit self-limiting characteristics, which results in prevalence being lower than incidence when measured at a specific point in time. This finding is consistent with previous studies.^15^

#### Disease burden of allergic-related skin diseases in different genders, years

**Fig. 1:**
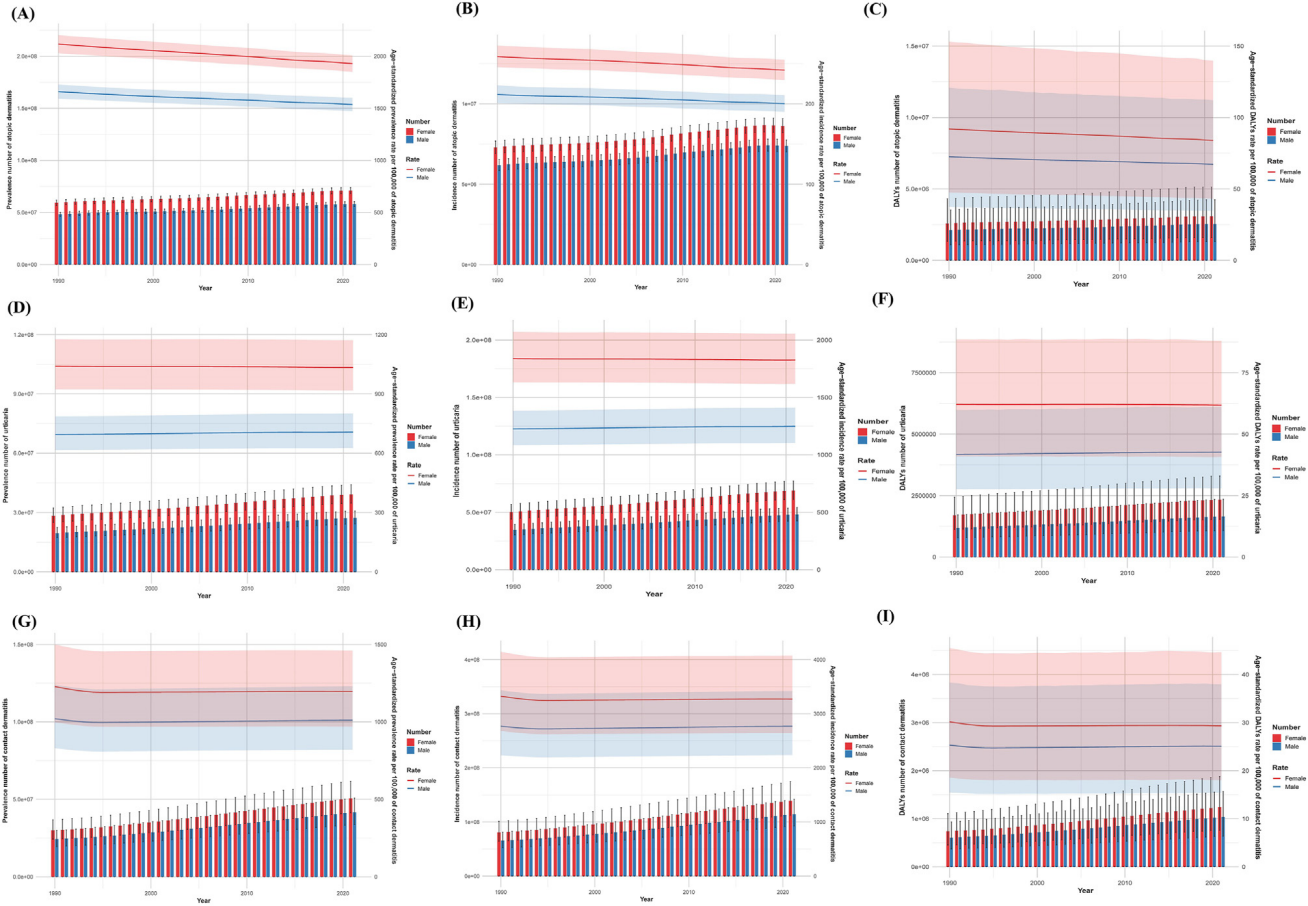
Trends in total cases and age-standardized rates (ASPR, ASIR, ASDR) of allergic-related skin diseases by gender from 1990 to 2021. Yearly changes are shown for: (A) total prevalence number and ASPR of AD; (B) total incidence number and ASIR of AD; (C) total DALYs number and ASDR of AD; (D) total prevalence number and ASPR of urticaria; (E) total incidence number and ASIR of urticaria; (F) total DALYs number and ASDR of urticaria; (G) total prevalence number and ASPR of CD; (H) total incidence number and ASIR of CD; (I) total DALYs number and ASDR of CD.

From 1990 to 2021, the average prevalence, incidence, and DALYs numbers and rates for AD, urticaria, and CD were consistently higher in females than males. For AD, although the prevalence and incidence numbers increased annually for both sexes from 1990 to 2021, the ASPR, ASIR, and ASDR showed a decreasing trend. During this period, the average ASDR in 100,000 people decreased by 187.91 in females, and by 8.04 in males; the ASIR decreased by 16.92 in females and 11.36 in males; the ASPR decreased by 123.91 in females and 5.24 in males. In the nearest year (2021), the female ASPR [1928.51 (95% UI: 1849.66, 2007.77) vs 1535.50 (95% UI: 1471.52, 1598.34)], ASIR [241.9 (95% UI: 229.46 to 255.01) vs 200.21 (95% UI: 189.90 to 210.83)], and ASDR [83.95 (95% UI: 43.22 to 139.69) vs. 67.27 (95% UI: 34.50 to 112.11)] value was higher compared to males (Fig. 1A-C, Supplemental Table 1).

In contrast, the ASPR, ASIR, and ASDR for CD and urticaria exhibited relatively minor fluctuations. The average ASDR (per 100,000 people) in females for CD and urticaria decreased by 32.19 and 6.86, respectively. In males, the average CD ASDR (per 100,000 people) decreased by 9.60, while urticaria increased by 12.67 (Fig. 1D–H, Supplemental Table 1).

#### Trends across age groups

**Fig. 2:**
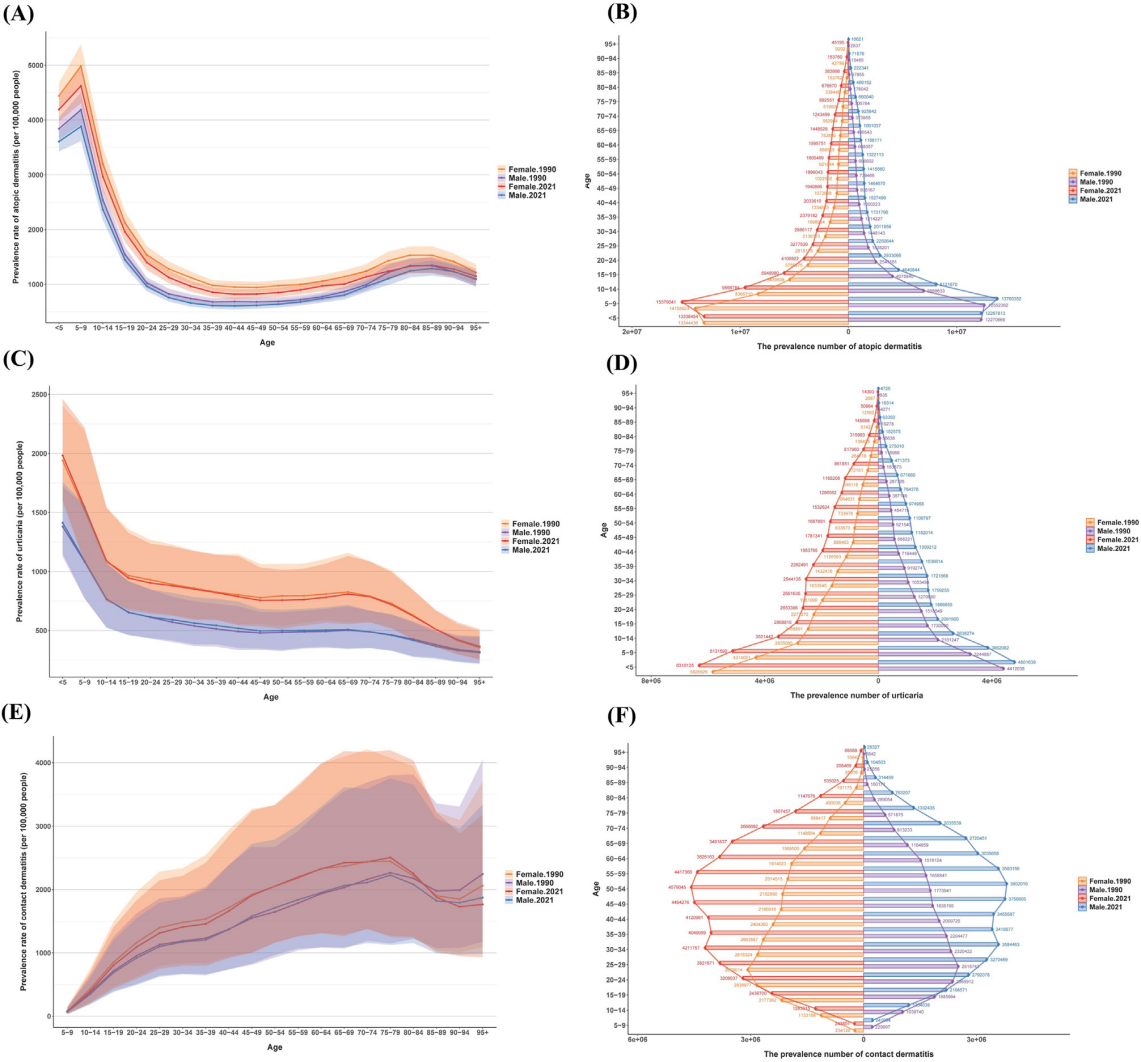
The changing trends of AD, urticaria, and CD in different genders and age groups from 1990 to 2021. (A) Prevalence rate of AD; (B) Prevalence number of AD; (C) Prevalence rate of urticaria; (D) Prevalence number of urticaria; (E) Prevalence rate of CD; (F) Prevalence number of CD.

Our study provides the first comprehensive analysis of the prevalence, incidence, and DALYs number and rate for AD, urticaria, and CD across the entire lifespan. In both 1990 and 2021, the prevalence rate of AD exhibited a peak in the 5–9 year age group, followed by a gradual decline with increasing age, stabilizing at a lower level during middle age. After the age of 75, the prevalence rate of AD rose again to above 1000 per 100,000 people, reaching a second smaller peak in the 80–89 year age group (Fig. 2A). The prevalence number for AD also continuously decreased after the age of 10, which may be related to global age structure changes (Fig. 2B).

The prevalence rate of urticaria peaked in the <5 year age group. At this stage, the ASPR for females reached 1983.12 (95% UI: 1613.08, 2460.02) per 100,000 people, while for males it remained at 1412.33 (95% UI: 1152.13, 1754.28). In middle age, the prevalence rate of urticaria remained relatively stable, with a continued decline observed in the 65–69 year age group (Fig. 2C). The prevalence number of urticaria decreased continuously with increasing age (Fig. 2D). Due to missing data on CD for those under 5 years of age in the GBD database, the study included prevalence data only for ages 5 to 95+. Unlike AD and urticaria, which showed a peak in prevalence during childhood or adolescence, CD exhibited its peak prevalence in the 75–79 year age group. In 2021, the prevalence number for both sexes presented an oval shape, with a peak at the 50–54 year age group (Fig. 2E and F). The trends in ASIR and ASDR generally mirrored those of ASPR (Supplemental Figs. 1–2). However, for AD, the ASIR value reached its peak in the <5 year age group, rather than in the 5–9 year age group. Detailed ASIR and ASDR values for different years are available in Supplemental Table 2.

#### Burden of allergic-related skin diseases in different SDI and regions

The ASPR, ASIR, and ASDR for AD increased with higher SDI levels. In high SDI regions, the ASPR, ASIR, and ASDR for AD reached 3364.38 (95% UI: 3234.39, 3507.08), 364.39 (95% UI: 345.43 to 384.48), and 146.99 (95% UI: 75.55 to 244.81), representing the highest values across all SDI tiers. However, the EAPC for ASPR in high SDI also indicates the most pronounced decline among the 5 SDI tiers. Among 21 regions, Eastern Europe exhibited the most significant increase in EAPC for ASPR at 0.21 (95% UI: 0.17, 0.24), while North Africa and the Middle East experienced the most notable decline at −0.15 (95% UI: −0.15, −0.14) (Table 2, Supplemental Table 3). For urticaria, the most significant declines in ASPR [−0.07 (95% CI: −0.08, −0.06)], ASIR [−0.06 (95% CI: −0.08, −0.05)], and ASDR [−0.06 (95% CI: −0.07, −0.05)] were observed in high-middle SDI, with relatively stable EAPC values across different regions (Table 2, Supplemental Table 3). In the meantime, the ASPR, ASIR, and ASDR values of CD peaked in the middle SDI in both 1990 and 2021. High-income North America had the highest ASPR in 1990, reaching 1932.78 (95% UI: 1604.78, 2297.53), but also had the largest EAPC decline at −0.48 (95% CI: −0.23, −0.72). By 2021, Southeast Asia had the highest ASPR at 1457.38 (95% UI: 1182.83, 1798.7), becoming the region with the greatest disease burden (Table 2, Supplemental Table 3).

**Table 2:** ASPR, ASIR, ASDR, and EAPC values for the 3 allergic-related skin diseases across 5 SDI tiers in 1990 and 2021

Location	ASPR (per 100,000 people)		EAPC in ASPR (95% UI	ASIR (per 100,000 people)		EAPC in ASIR (95% UI	ASDR (per 100,000 people		EAPC in ASDR (95% CI)
	1990	2021		1990	2021		1990	2021	
**Atopic dermatitis**									
Low SDI	1199.62 (1147.07, 1252.31)	1171.1 (1119.89, 1223.87)	−0.08 (−0.09, −0.07)	168.7 (159.8, 178.31)	164.02 (155.25, 173.16)	−0.09 (−0.1, −0.09)	52 (26.92, 87.37)	51.1 (26.14, 85.42)	−0.05 (−0.06, −0.05)
Low-middle SDI	1499.34 (1434.36, 1566.92)	1450.53 (1388.11, 1514.66)	−0.12 (−0.13, −0.11)	205.99 (194.71, 218.53)	199.27 (188.54, 210.86)	−0.13 (−0.13, −0.12)	65.27 (33.66, 109.6)	63.42 (32.6, 105.75)	−0.11 (−0.12, −0.1)
Middle SDI	1589.36 (1523.69, 1658.38)	1591.28 (1526.71, 1657.34)	0 (0, 0.01)	217.88 (206.98, 229.38)	216.92 (205.93, 228.01)	−0.02 (−0.02, −0.01)	69.58 (35.9, 116.47)	69.84 (35.96, 116.91)	0.01 (0.01, 0.02)
High-middle SDI	2103.18 (2018.71, 2191.06)	2065.66 (1978.78, 2151.14)	0.04 (0.01, 0.08)	240.35 (227.55, 253.98)	242.06 (228.98, 255.8)	0.06 (0.03, 0.1)	92.22 (47.52, 153.26)	90.8 (46.58, 151.24)	0.05 (0.02, 0.09)
High SDI	3364.38 (3234.39, 3507.08)	3233.96 (3105.94, 3369.25)	−0.14 (−0.15, −0.13)	364.39 (345.43, 384.48)	356.59 (338.03, 376.39)	−0.08 (−0.1, −0.07)	146.99 (75.55, 244.81)	141.11 (72.7, 234.6)	−0.14 (−0.16, −0.13)
**Urticaria**									
Low SDI	889.71 (787.09, 1010.74)	880.78 (779.38, 1000.97)	−0.03 (−0.03, −0.03)	1568.76 (1384.35, 1772.89)	1553.25 (1369.27, 1755.9)	−0.03 (−0.03, −0.03)	52.78 (34.86, 75.12)	52.59 (34.58, 75.14)	0 (−0.01, 0)
Low-middle SDI	938.01 (826.49, 1070.79)	940.34 (829.37, 1071.99)	0.01 (0.01, 0.01)	1655.91 (1460.85, 1877.51)	1659.3 (1463.91, 1881.72)	0.01 (0.01, 0.01)	55.88 (36.73, 79.61)	56.29 (36.93, 80.77)	0.03 (0.03, 0.03)
Middle SDI	823.42 (727.19, 937.6)	831.29 (734.28, 945.55)	0.04 (0.03, 0.04)	1455.17 (1279.81, 1647.51)	1470.2 (1296.8, 1663.96)	0.04 (0.04, 0.05)	49.47 (32.58, 70.99)	50.02 (32.9, 71.73)	0.05 (0.04, 0.05)
High-middle SDI	845.54 (751.77, 956.68)	823.08 (731.44, 930.93)	−0.07 (−0.08, −0.06)	1492.96 (1319.1, 1687.08)	1455.44 (1284.46, 1643.64)	−0.06 (−0.08, −0.05)	50.9 (33.59, 72.87)	49.65 (32.75, 71.32)	−0.06 (−0.07, −0.05)
High SDI	816.36 (741.79, 894.85)	811.83 (743.91, 881.48)	−0.03 (−0.04, −0.02)	1440.48 (1304.99, 1581.39)	1433.17 (1306.16, 1562.71)	−0.03 (−0.03, −0.02)	49.06 (32.54, 68.41)	48.72 (32.43, 67.89)	−0.03 (−0.04, −0.03)
**Contact dermatitis**									
Low SDI	983.98 (799.69, 1195.88)	982.24 (799.25, 1193.6)	−0.01 (−0.01, 0)	2725.2 (2209.07, 3370.98)	2719.87 (2202.63, 3361.06)	−0.01 (−0.01, −0.01)	23.97 (14.73, 36.33)	24.03 (14.79, 36.26)	0.02 (0.01, 0.02)
Low-middle SDI	1104.5 (892.85, 1352.76)	1098.72 (888.88, 1345.3)	−0.02 (−0.02, −0.02)	3046.53 (2473.13, 3762.88)	3031.77 (2458.62, 3742.09)	−0.02 (−0.02, −0.02)	27.01 (16.63, 40.79)	26.92 (16.56, 40.72)	−0.01 (−0.01, 0)
Middle SDI	1271.55 (1023.89, 1555.78)	1255.09 (1010.33, 1534.49)	−0.04 (−0.04, −0.04)	3506.61 (2824, 4383.92)	3460.05 (2790.69, 4317.89)	−0.04 (−0.04, −0.04)	31.41 (19.27, 47.67)	30.99 (18.98, 47.26)	−0.04 (−0.04, −0.04)
High-middle SDI	1103.57 (901.35, 1336.18)	1127.26 (921.39, 1365.63)	0.07 (0.07, 0.07)	3040.54 (2460.21, 3780.35)	3107.51 (2516.59, 3867.16)	0.07 (0.07, 0.07)	27.35 (16.91, 41.49)	27.97 (17.15, 42.55)	0.08 (0.07, 0.08)
High SDI	1075.55 (894.27, 1281.7)	939.32 (782.48, 1113.83)	−0.08 (−0.21, 0.05)	2676.61 (2177.52, 3324.78)	2410.56 (1968.41, 2957.82)	−0.03 (−0.14, 0.08)	26.63 (16.71, 39.71)	23.2 (14.71, 34.69)	−0.08 (−0.21, 0.04)

### Global distribution of allergic-related skin diseases

**Fig. 3:**
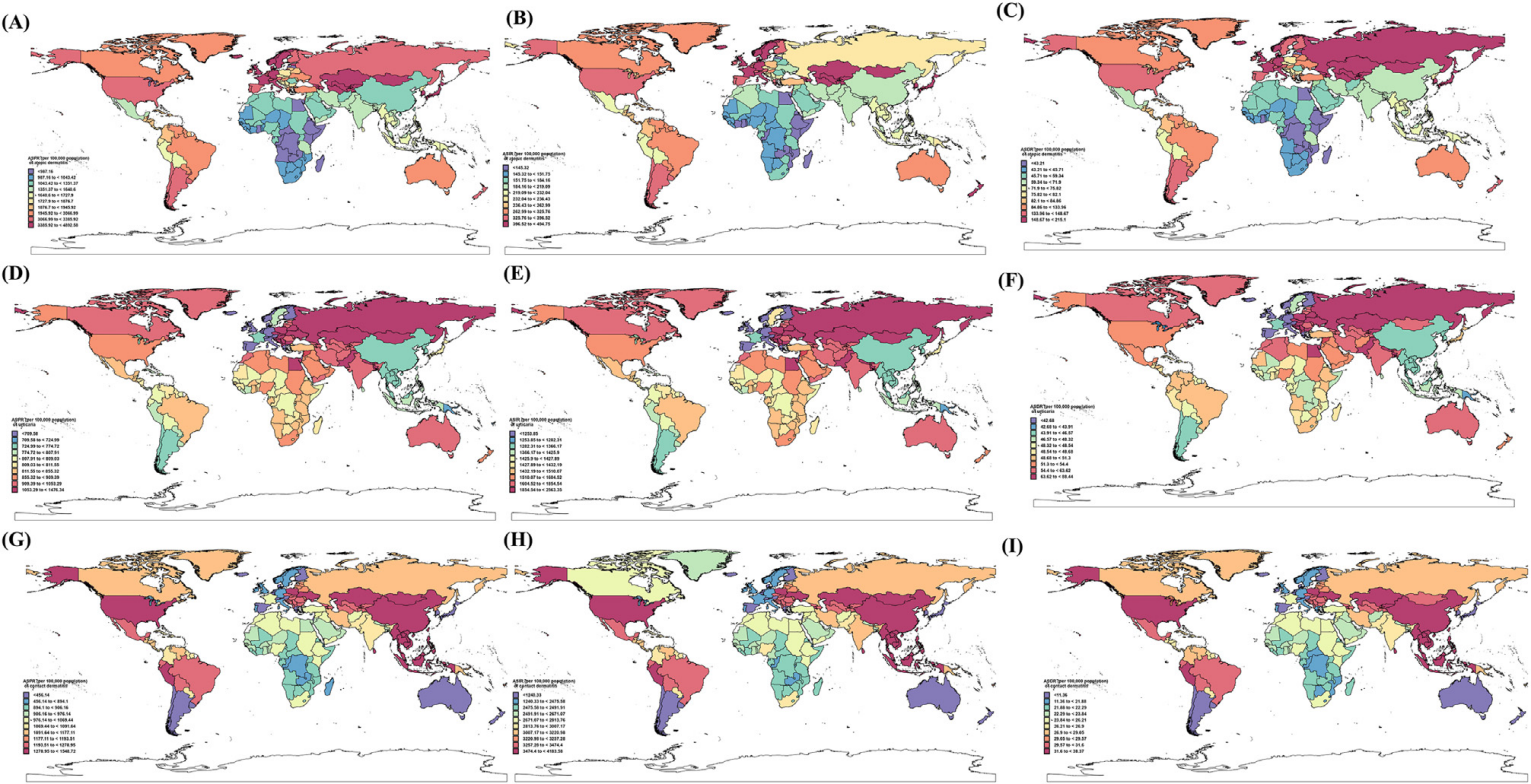
The global distribution of ASPR, ASIR, and ASDR for allergic-related skin diseases in 2021 is stratified by deciles, as shown in the legend in the lower-left corner of the figure. (A) AD ASPR; (B) AD ASIR; (C) AD ASDR; (D) Urticaria ASPR; (E) Urticaria ASIR; (F) Urticaria ASDR; (G) CD ASPR; (H) CD ASIR; (I) CD ASDR. Countries or regions shown in white indicate missing data.

By mapping the global ASPR distribution across countries and regions, the study found that in 2021, Japan had the highest ASPR, ASIR, and ASDR values for AD, reaching 4892.58 (95% UI: 4683.90, 5124.84), 494.74 (95% UI: 467.73, 525.79), and 215.10 (95% UI: 109.77, 358.88), respectively, making it the country with the highest burden of AD. Japan, France, and Kazakhstan were the top 3 countries for ASPR and ASDR values, consistent with the rankings in 1990. In the ASIR rankings, Japan, France, Italy, Denmark, and South Korea were the top 5 countries. In both 1990 and 2021, only Congo and Rwanda had ASPR values below 800 per 100,000 people, with Rwanda having an ASPR of 687.58 (95% UI: 733.76 to 646.68) per 100,000 people in 2021 (Fig. 3A, Supplemental Table 4).

Urticaria's ASPR exceeded 1000 per 100,000 population in 34 countries. Nepal had the highest ASPR, recorded at 1476.34 (95% UI: 1317.43, 1605.26), maintaining its top ranking since 1990. In contrast, 3 European nations—Italy, Germany, and Portugal—had ASPR values below 500, placing them at the lower end of the rankings (Fig. 3C). Nepal, Poland, and Pakistan recorded the highest ASIR and ASDR values among the 204 countries/regions globally. Nepal's ASIR reached 2563.35 (95% UI: 2274.38, 2794.79), while its ASDR was 88.44 (95% UI: 57.09, 124.89). Italy, Germany, and Portugal were among the lowest ASIR and ASDR values across the 204 countries/regions (Fig. 3E-F, Supplemental Table 4).

In Sri Lanka, the Philippines, and Indonesia, CD had the highest ASPR rates. Sri Lanka's ASPR value reached 1548.72 (95% UI: 1274.53, 1870.43) per 100,000 people (Fig. 3G). In 2021, the Philippines ranked first in ASIR, followed by Indonesia and Sri Lanka. The 5 countries with the lowest ASIR values (Australia, South Korea, Singapore, Brunei Darussalam, and Denmark) maintained their rankings in 1990 and 2021 (Fig. 3H). Notably, in 1990, the USA had the highest ASPR, ASIR, and ASDR for CD; however, by 2021, the United States ranked fifth in ASPR, seventeenth in ASIR, and eighth in ASDR (Fig. 3G-I, Supplemental Fig. 3). This indicates significant progress in controlling CD in the United States. Detailed mapping data for 1990 can be found in Supplemental Table 4.

### Joinpoint regression analysis

The Joinpoint regression model identifies shifts in time-series trends. For AD, ASIR, and ASPR showed the best fit with 6 joinpoints. AD metrics (ASIR, ASPR, ASDR) all decreased, with AAPC values of −0.20, −0.28, and −0.27, respectively (Supplemental Fig. 4A–4C, Supplemental Table 5). Urticaria showed mild fluctuations during 1990–2021, with ASIR values between 1528 and 1536 per 100,000 people and an AAPC of 0.01 (95% CI: 0.01, 0.01). The APC values for 1990 to 1994 and 2013 to 2018 were −0.01 and −0.04, respectively, while other periods showed positive values (Supplemental Fig. 4D and 4E, Supplemental Table 5). For CD, the ASPR and ASIR showed negative APC values of −0.67 (95% CI: −0.71, −0.64) and −0.52 (95% CI: −0.54, −0.50) from 1990 to 1994. After 1994, the APC values steadily increased (Supplemental Fig. 4G and 4H, Supplemental Table 5). The ASDR for CD showed a value of −0.06 (95% CI: −0.07, −0.05) over the period from 1990 to 2021. The discrepancy between AAPC and EAPC likely results from data non-linearity, with AAPC better capturing long-term trends as a weighted average.

### Correlation analysis between allergic-related skin diseases ASIR and SDI

**Fig. 4:**
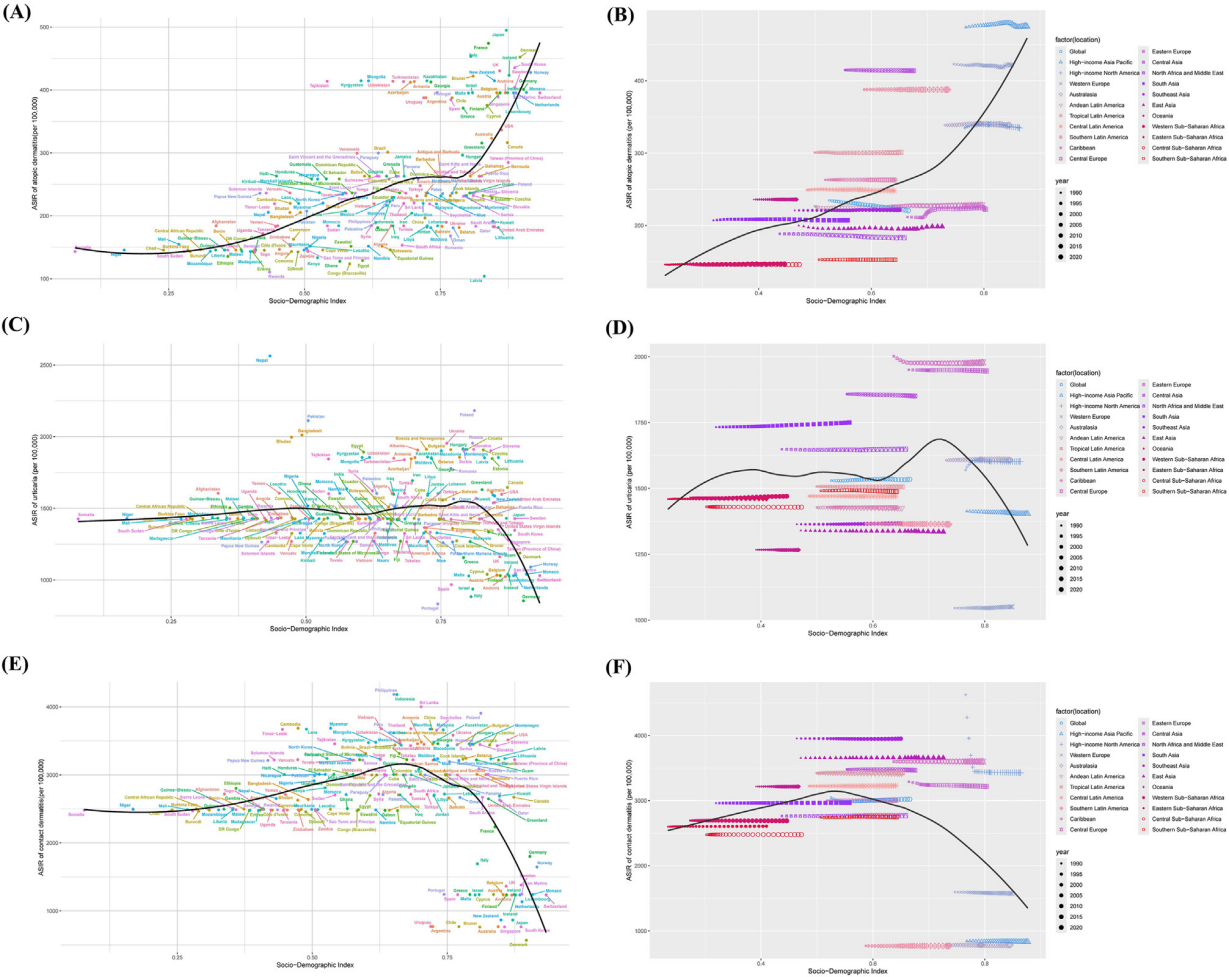
Changes in the ASIR of allergic-related skin diseases globally and across different SDI regions and countries. Different regions are represented by distinct colors and symbol shapes. The black line indicates the expected age-standardized rates in 2021 based solely on SDI. (A) National AD and SDI correlation; (B) Regional AD and SDI correlation; (C) National urticaria and SDI correlation; (D) Regional urticaria and SDI correlation; (E) National CD and SDI correlation; (F) Regional CD and SDI correlation.

Spearman's Rank Correlation analysis shows a significant correlation between the ASIR of AD and SDI (*p* = 2.2e-16, ρ = 0.626). When SDI increases, ASIR also tends to increase. High-income Asia Pacific regions (such as Japan and South Korea), high-income North America (such as the USA and Canada), and Western Europe (such as France, Denmark, and Italy) show higher ASIR, while low SDI countries (such as Sub Saharan Africa and South Asia) tend to have lower ASIR (Fig. 4A and B).

Central Europe, Eastern Europe, and Central Asia are the top 3 regions with the highest urticaria ASIR values, averaging 1978.56, 1945.30, and 1850.83 per 100,000 people. Notably, some South Asian countries, such as Nepal, Pakistan, Bangladesh, and Bhutan, show exceptionally high ASIR values of 2563.35, 2111.54, 2011.38, and 1996.36 per 100,000 people (Fig. 4A and B). These countries may need to focus more on urticaria in their preventive and therapeutic strategies for allergic-related skin diseases.

### Cross-national health inequality analysis based on DALYs

**Fig. 5:**
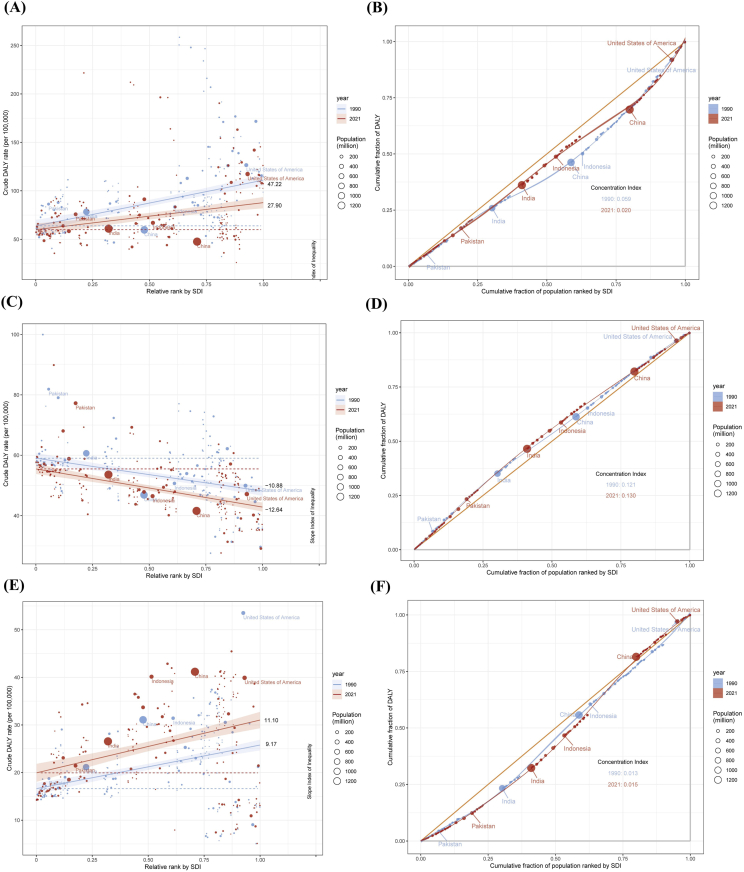
The top 5 most populous countries in the world are highlighted, with red solid points and curves representing 2021 data and blue solid points and curves representing 1990 data. The size of the points reflects the population size of each country or region. (A) Health inequality regression curves for AD; (B) Concentration curves for AD; (C) Health inequality regression curves for urticaria; (D) Concentration curves for urticaria; (E) Health inequality regression curves for CD; (F) Concentration curves for CD.

Both AD and CD had positive Slope Index of Inequality (SII) values, indicating that the burden of these diseases is concentrated in countries with higher SDI. For AD, the SII decreased from 47.22 (95% CI: 36.20, 58.24) in 1990 to 27.90 (95% CI: 17.53, 38.26) in 2021. The intercept for DALYs was 63.79 (95% CI: 56.90, 70.68) and 59.89 (95% CI: 53.37, 66.40) in 1990 and 2021 for AD. Additionally, the concentration index dropped from 0.059 in 1990 to 0.020 in 2021, indicating a more equitable distribution of health burdens by 2021. Among the 5 most populous countries, Indonesia showed the most notable decline in the concentration index from 1990 to 2021 (Fig. 5A and B).

In 1990, the SII for DALYs due to urticaria was −10.88 (95% CI: −14.20, −7.56), changing to −12.64 (95% CI: −15.37, −9.90) by 2021. This suggests a negative correlation between ASDR and the SDI, with the burden of urticaria continuing to widen between high-income and low-income countries. The concentration index rose from 0.121 in 1990 to 0.130 in 2021, with Pakistan showing a significant health burden among the major populous nations (Fig. 5C and D). CD exhibited similar results, with an SII for DALYs of 9.17 (95% CI: 6.50, 11.83) in 1990 and 11.10 (95% CI: 7.41, 14.79) in 2021. However, the average concentration index only increased by 0.002 in 2021 compared to 1990 (Fig. 5E and F).

### Frontier analysis of the disease burden of allergic-related skin diseases

Frontier analysis evaluates country or regional performance at various SDI levels by identifying the “Best Practice Boundary,” marking the lowest achievable disease burden across development stages. For AD, the top 20 regions with the largest effective differences from the frontier ranged from 185.86 to 122.01, with Japan, France, Kazakhstan, Mongolia, and Kyrgyzstan leading. High-SDI regions like Japan and France showed an upward trend, remaining distant from the best practice boundary, while low-SDI countries, Somalia and Rwanda, had the smallest gaps (Supplemental Fig. 5A and 5B). Urticaria shows a marked decrease around an SDI of 0.6, likely due to medical advancements, public health measures, and changing disease patterns. High-SDI countries such as Lithuania, Canada, and the USA indicate room for further improvement, while only Poland and Nepal had effective difference values above 40 (Supplemental Fig. 5C and 5D). The best practice boundary sharply declines around an SDI of 0.6 in CD. Sri Lanka, Indonesia, and the Philippines had the largest gaps from the frontier, and all the top 10 countries, except Poland saw a reduction in DALYs rates compared to 1990 (Supplemental Fig. 5E and 5F).

### The BAPC model predicts the global burden of allergic-related skin diseases for the next 15 years

**Fig. 6:**
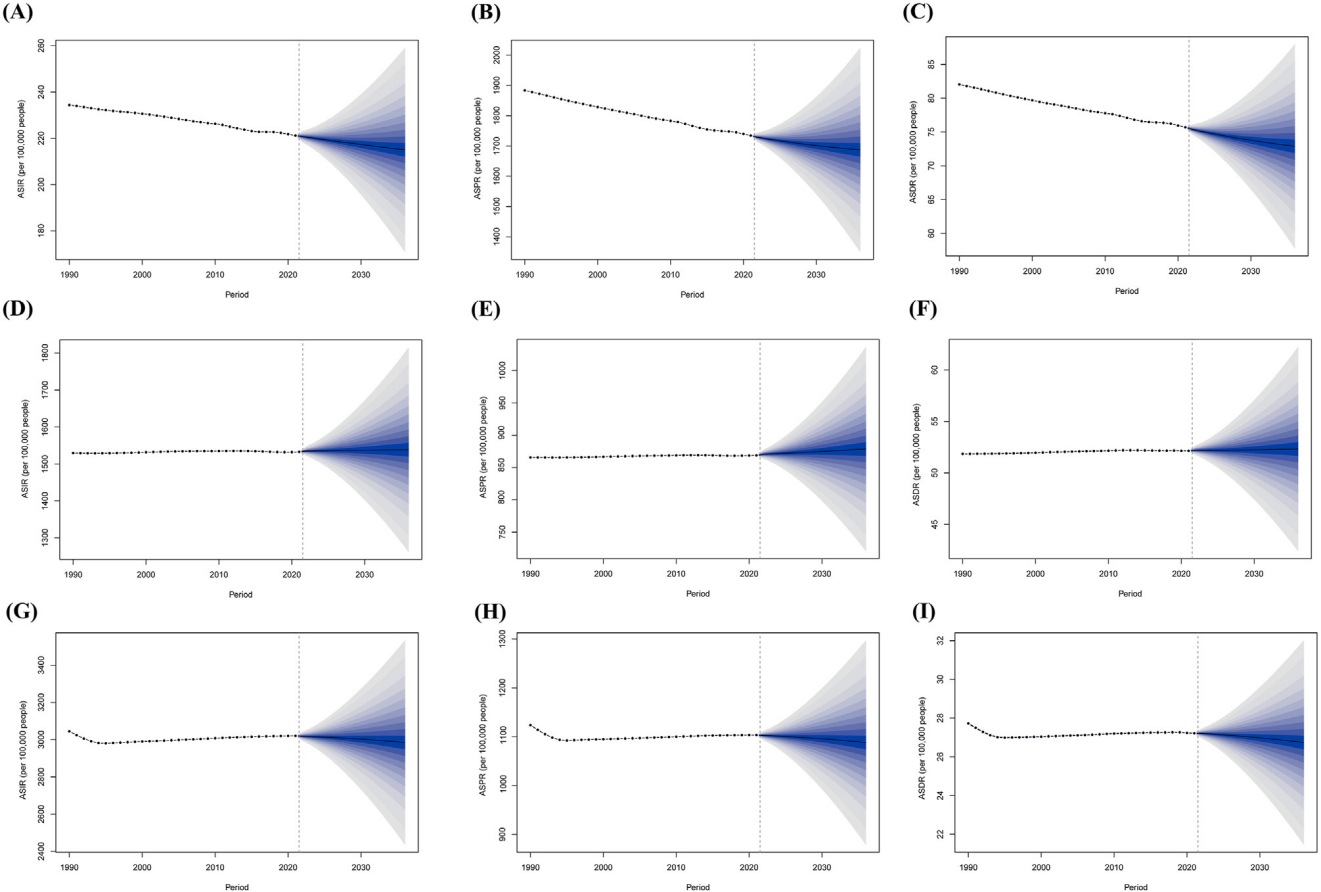
The BAPC model predicts the changes in different age standardized rates by 2036. (A) AD ASIR; (B) AD ASPR; (C) AD ASDR; (D) Urticaria ASIR; (E) Urticaria ASPR; (F) urticaria ASDR; (G) CD ASIR; (H) CD ASPR; (I) CD ASDR.

Predicting the future epidemiological trends of allergic-related skin diseases is crucial for guiding policy priorities. The study suggests that over the next 15 years, the incidence, prevalence, and DALYs for AD per 100,000 people will decline to 214.97, 1687.00, and 72.88, indicating significant improvement compared to the current situation (Fig. 6, Table 1). Globally, the ASIR, ASPR, and ASDR for CD are also expected to decrease by 35.59, 15.38, and 0.48 per 100,000 people by 2036. However, projections for urticaria indicate increases in ASIR, ASPR, and ASDR by 5.87, 9.94, and 0.17. While progress has been made in the prevention and treatment of AD and CD, more effective therapeutic strategies are still required for urticaria.

## Discussion

Allergic-related skin diseases continue to impose a significant global health burden, with high prevalence and incidence rates for all 3 diseases. In the updated data, the total number of people affected by AD has increased annually, reaching 129 million in 2021, which represents a 20.02% rise compared to 1990. Despite the growing number of cases, both the ASIR [AAPC = −0.20 (95% CI: −0.21, −0.20)] and ASPR [AAPC = −0.28 (95% CI: −0.28, −0.28)] of AD have declined from 1990 to 2021. As a chronic, relapsing inflammatory skin disease, AD typically has a long course and is part of the atopic predisposition, requiring long-term intervention.

In recent years, small-molecule targeted drugs (baricitinib, upadacitinib, abrocitinib) and biologics (dupilumab, lebrikizumab) have been reported to significantly relieve the symptoms relief for AD patients.^29^, ^30^, ^31^, ^32^ Multiple clinical trials support the success of these therapies in treating AD while bringing about fewer side effects.^31^^,^^33^^,^^34^ The study found that AD has the highest prevalence in childhood (5–9 years) and increases again in older populations (75 years and above), which is consistent with previous reports.^35^ From a geographical perspective, ASIR for AD is higher in high-income regions, such as the Asia-Pacific, North America, and Western Europe, with a significant positive correlation between ASIR and the SDI. In contrast, South Sub-Saharan Africa generally has lower AD incidence rates, suggesting that lower socioeconomic conditions in these regions may be linked to reduced AD risk. Some studies suggest that high-SDI countries often face greater exposure to pollution and allergens, unhealthier lifestyle and dietary habits, and reduced microbial exposure in early life, all of which can drive immune systems towards allergic responses, increasing the risk of atopic dermatitis.^36^, ^37^, ^38^ Frontier analysis revealed that high-SDI countries like Japan, France, and Italy have a higher actual disease burden than the minimum achievable burden along the “frontier line,” indicating that these nations still have the potential to further reduce their disease burden. Fortunately, the health inequality index (SII) for AD decreased from 47.22 in 1990 to 27.90 in 2021, which indicates the disease burden of AD has become more evenly distributed across different socioeconomic groups.

Urticaria is characterized by acute or chronic episodes and can be triggered by exogenous stimulates. In 2021, the global prevalence of urticaria reached 66.48 million cases. Between 1990 and 2021, both the ASIR and ASPR of urticaria remained stable, with an AAPC of 0.01 (95% CI: 0.01, 0.01). Overall, the incidence, prevalence, and DALYs were higher in females than in males during this period. However, incidence rates in males showed a positive growth trend, while females experienced a decline. What is particularly noteworthy is that across age groups, the prevalence of urticaria peaked in infancy (<5 years), remained stable through early to middle adulthood, and declined again in later years (65–69 years). This age distribution may result from multiple factors, with the immature immune system in infants causing stronger reactions to new foods and environmental allergens, leading to higher prevalence rates in this age group.^39^^,^^40^ As individuals age, the immune system gradually weakens, potentially reducing allergic reactions to external stimuli, which aligns with recent clinical observations.^41^ However, urticaria shows no significant correlation with the SDI. The SDI serves as a general indicator of population health and economic development across nations and regions. It's crucial to recognize that key etiological determinants of urticaria - including environmental triggers (air pollution (such as air pollution,^42^^,^^43^ climate change^5^), lifestyle (such as diet,^44^ mental pressure^45^), and genetic factors are not directly included in the SDI. These multifactorial elements, particularly in the globalized world, exert influences that transcend SDI stratification boundaries. Meanwhile, the transient and self-limiting nature of urticaria (particularly acute cases characterized by rapid onset and spontaneous resolution), combined with potential underreporting due to these characteristics, may collectively contribute to the lack of a clear correlation between SDI levels and urticaria burden. Geographically, urticaria ASIR rates are higher in Central Europe, Eastern Europe, Central Asia, and South Asia, while Western Europe exhibits relatively lower ASIR levels.

CD had the highest number of new cases of allergic-related skin diseases in 2021, reaching 253 million. Joinpoint regression analysis indicates a decreasing trend in CD's ASPR and ASIR before 1994, but a steady rise after 1994, which warrants the attention of epidemiologists and dermatologists. A report suggests that this may be related to the increasing global industrialization and exposure to chemical products.^46^ Additionally, advancements in diagnostic techniques cannot be overlooked as a factor contributing to the rise in ASPR and ASIR values. Allergic contact dermatitis is an immune-mediated delayed hypersensitivity reaction where allergens bind to skin proteins, forming complexes that are taken up by Langerhans cells and activate specific T-cells.^47^^,^^48^ This stage typically shows intercellular edema and damage to keratinocytes in the epidermis. Yasutaka Mitamura and team^49^ believe that a compromised skin barrier is more susceptible to external substances, leading to CD. In this study, we also found that the peak prevalence of CD occurs in the 75–79 age group. In elderly patients, skin becomes drier, thinner, and more prone to damage, increasing sensitivity to chemicals and allergens.^50^ Geographically, the CD is more prevalent in Southeast Asia, East Asia, and Central Europe. In these regions, excessive exposure to irritants, in addition to genetic factors, may increase CD incidence. Literature reports indicate that high exposure to rubber and insect bites. According to recent reports, exposure to rubber, insects,^40^ and chemicals (such as carbon mix, nickel sulfate, and potassium dichromate)^51^^,^^52^ in Southeast Asian countries contributes to its high prevalence. It is also important to note that the global health inequality of CD has shown no significant change since 1990, with persistent disparities in disease burden between countries.

The BAPC model considers the effects of age, time (period), and cohort on health trends. This enables it to comprehensively capture variations across different dimensions, which has an ideal long-term trend prediction. Over the next 15 years, the burden of allergic-related skin diseases is expected to remain significant. The BAPC model predicts that the ASIR, ASPR, and ASDR for urticaria and CD are unlikely to decrease significantly, with urticaria potentially even increasing. However, it is promising that the burden of AD is projected to decrease. This newly revealed epidemiological trend suggests that future prevention strategies should place greater emphasis on rapid response and personalized treatment. However, the BAPC model relies on historical data for predictions, making it susceptible to inaccuracies during unforeseen events such as large-scale epidemics or natural disasters. The BAPC model may underestimate other influential factors such as environmental changes and policy interventions. In the future, more advanced models can be used to improve the accuracy of disease prediction.

Allergy-related skin diseases have seen gradual improvements in diagnostic criteria with advancements in medical knowledge and the enhancement of diagnostic tools. The application of new diagnostic standards and technologies (such as skin patch tests,^53^ serological tests^54^) has led to the timely detection and diagnosis of more cases, improving the diagnostic ability for allergy-related skin diseases. At the same time, with the upgrading of antihistamines,^55^ the development of immunomodulatory therapies, and biologics,^34^^,^^56^ the symptoms of allergy-related skin diseases have been significantly alleviated, improving patients' quality of life and reducing severe complications or hospitalization rates caused by these conditions, thereby impacting DALYs. Additionally, for some long-term allergic diseases, more effective management and treatment may reduce acute flare-ups, lowering the long-term accumulation of disease burden, and thus stabilizing ASPR or ASIR.^57^^,^^58^

AD, urticaria, and CD show varying patterns across different genders, ages, regions, and SDI levels, highlighting current prevention priorities for allergic-related skin diseases and guiding future policy-making. This study addresses gaps in the current epidemiological research on these conditions. However, the main limitation lies in the heterogeneity of the data and differences in diagnostic standards, which may affect direct comparisons between countries and regions. Despite this, the broad coverage of the study and the use of standardized diagnostic criteria enhance the reliability of the results. To improve data quality, future research can quantify the uncertainty in the data and reveal the potential impact of bias on conclusions by using bias-correction models, sensitivity analysis, hypothesis testing, and error range estimation. In the meanwhile, some countries/regions affected by conflicts or with smaller populations, as well as those that are difficult to access data may cause missing data in the GBD database. Future efforts can focus on strengthening data collection and reporting mechanisms, supplementing missing or sparse data by utilizing health data from neighboring regions and employing automated methods such as big data and artificial intelligence technologies. Meanwhile, in future research, we plan to consider these additional socioeconomic factors and conduct more detailed analyses to elucidate their specific impacts on the burden of allergic-related skin diseases. Enhancing data collection and analysis will facilitate a more precise understanding of the burden and contributing factors of allergic skin diseases, thereby providing an evidence-based foundation for the development of targeted preventive interventions and the optimization of clinical management strategies.

## Conclusion

This study provides a comprehensive analysis of the global burden of allergic-related skin diseases, including AD, urticaria, and CD, from 1990 to 2021. It reveals that although incidence and prevalence rates vary across regions and countries, the overall trends are generally consistent with previous research. Despite a decline in the burden of AD in certain areas, the global burden of allergic-related skin diseases remains significant, with marked differences in incidence and prevalence across regions. The treatment and prevention of allergic-related skin diseases still present challenges. It is crucial to focus on the epidemiological characteristics of different regions and countries, explore potential risk factors, and develop effective prevention and treatment strategies for different types of allergic-related skin diseases.

## Abbreviations

Global burden of diseases (GBD); Atopic dermatitis (AD); Contact dermatitis (CD); Socio-Demographic Index (SDI); Disability-adjusted life years (DALYs); Age-standardized incidence rates (ASIR); Age-standardized prevalence rates (ASPR); Age-standardized disability-adjusted life year rates (ASDR); Age-Period-Cohort (BAPC); International Classification of Diseases (ICD); Age-standardized rates (ASR); Estimated annual percentage change (EAPC); Average annual percentage change (AAPC); Annual percentage change (APC); Uncertainty interval (UI); Concentration Index (CI); Slope Index of Inequality (SII)

## Authors’ consent for publication

All authors have given consent for publication.

## Availability of data and materials

The data analyzed during the study are available in the GBD database.

## Author contributions

Xiao Tang designed the study and involved in the writing-original draft of the manuscript. Li Lin made writing-review and editing. Fangning Yu made professional revisions to the article. Yizhao Ma and Zeyu Liu discussed the content ideas and investigation in the article. Xuying Xu was involved in the conceptualization and supervision of this manuscript.

## Ethics statement

Ethics approval was not required for this study as it is based on publicly available data without the direct engagement of participants. The University of Washington’s Institutional Review Board approved the GBD 2021 study (https://www.healthdata.org/research-analysis/gbd). Therefore, open data does not require further approval from the local ethics committee.

## Confirmation of unpublished work

The authors decline manuscript is original, has not been published before, is not currently being considered for publication elsewhere.

## Funding statement

This article is funded by (1) General Project of 10.13039/501100001809National Natural Science Foundation of China (No.82174388); (2) Beijing Hundred, Thousand, and Ten Thousand Talents Project Training Fund Support Project (No.2019A30); (3) Beijing Medical Center The “Peak Climbing” Talent Training Program Project (No.DFL20220801).

## Declaration of competing interest

The authors report no competing interests.
