# Supplementary material for: Allergic-related skin diseases: Global disease burden from 1990 to 2021 and future trends
Source: World Allergy Organ J. 2025 Jun 3;18(7):101072. doi: 10.1016/j.waojou.2025.101072 (PMC12167089; doi:10.1016/j.waojou.2025.101072)
Supplement: Multimedia component 1 [file mmc1.pdf]

Supplemental Table 1: All-age cases and age-standardized rates of prevalence, incidence, and DALYs for allergic skin diseases by gender.(Disability-adjusted life years: DALYs)

Measure	All-ages cases (95%UI)				Age-standardized rate (per 100,000 people) (95%UI)			
	1990 male	1990 female	2021 male	2021 female	1990 male	1990 female	2021 male	2021 female
**Atopic dermatitis**								
Prevalence	48163280 (46111360,50232709)	59334381 (56819416,61816085)	58017761 (55646956,60326509)	71000908 (68185563,73887903)	1659.41 (1592.76,1728.12)	2116.42 (2028.92,2202.51)	1535.50 (1471.52,1598.34)	1928.51 (1849.66,2007.77)
Incidence	6183580 (5850345,6536380)	7295632 (6912355,7687317)	7381252 (7002029,7757744)	8625428 (8207395,9079673)	211.57 (200.76,223.01)	258.82 (245.65,272.5)	200.21 (189.90,210.83)	241.9 (229.46,255.01)
DALYs	2112225 (1087790,3526633)	2585750 (1333388,4304519)	2540263 (1303408,4237268)	3081245 (1588267,5129866)	72.51 (37.35,120.87)	91.99 (47.46,153.14)	67.27 (34.50,112.11)	83.95 (43.22,139.69)
**Urticaria**								
Prevalence	19570993 (17249866,22446256)	28378607 (25018977,32395115)	27295554 (24224116,30814456)	39187919 (34833859,44102385)	693.93 (615.57,786.17)	1040.28 (923.26,1176.78)	706.6 (626.32,800.81)	1033.42 (916.66,1171.83)
Incidence	34655011 (30316679,39373942)	50215277 (44106497,56957967)	48063239 (42546283,54104012)	68951348 (61398516,77346661)	1225.82 (1083.29,1385.02)	1837.47 (1631.32,2071.16)	1248.68 (1102.97,1410.89)	1825.42 (1618.02,2054.93)
DALYs	1183232 (780472,171495	1700734 (1121092,24399	1646426 (1081043,23576	2334360 (1536501,33005	41.77 (27.59,59.82)	62.12 (40.93,88.59)	42.67 (28.00,61.23)	61.79 (40.69,88.03)

	0)	49)	35)	12)				
**Contact dermatitis**								
Prevalence	24329301 (19608399,29924865)	29974637 (24279412,36728246)	41638331 (33738853,50737765)	50619315 (41188943,61647941)	1020.22 (830.09,1239.79)	1227.77 (997.43,1500)	1010.61 (820.79,1229.42)	1195.58 (969.93,1460.58)
Incidence	65421425 (52251736,81332754)	80616937 (64502743,101190464)	114288889 (92697437,141836839)	139015847 (112452433,174408040)	2767.63 (2234.04,3432.71)	3323.04 (2680.96,4145.42)	2767.18 (2236.24,3420.72)	3270.5 (2641.42,4072.34)
DALYs	608090 (372896,932948)	739139 (453848,1104825)	1034488 (636450,1566612)	1238277 (760944,1878441)	25.31 (15.46,38.3)	30.16 (18.55,45.51)	25.10 (15.39,37.93)	29.32 (18.00,44.68)

Supplemental Table 2: Rates and numbers for AD, urticaria, and CD by age and gender in 1990 and 2021.(Atopic dermatitis: AD; Contact dermatitis: CD)

Diseases	Year	Measure name	Metric name	Sex name	Age	Value	Lower	Upper
AD	1990	DALYs	Number	Female	<5	590095	306133	979515
AD	1990	DALYs	Number	Female	5-9	626394	323489	1062209
AD	1990	DALYs	Number	Female	10-14	368195	186943	633909
AD	1990	DALYs	Number	Female	15-19	236992	123274	395079
AD	1990	DALYs	Number	Female	20-24	162600	83694	270279
AD	1990	DALYs	Number	Female	25-29	121398	62624	210024
AD	1990	DALYs	Number	Female	30-34	91760	47248	151504
AD	1990	DALYs	Number	Female	35-39	72825	37036	122117
AD	1990	DALYs	Number	Female	40-44	56871	29168	96580
AD	1990	DALYs	Number	Female	45-49	45551	23153	76706
AD	1990	DALYs	Number	Female	50-54	43114	21869	74614
AD	1990	DALYs	Number	Female	55-59	38587	20365	67238
AD	1990	DALYs	Number	Female	60-64	35925	19524	60814
AD	1990	DALYs	Number	Female	65-69	30702	16217	50942
AD	1990	DALYs	Number	Female	70-74	23422	12533	38643
AD	1990	DALYs	Number	Female	75-79	20473	10587	34426
AD	1990	DALYs	Number	Female	80-84	13121	6983	22205
AD	1990	DALYs	Number	Female	85-89	5813	3149	9899
AD	1990	DALYs	Number	Female	90-94	1580	855	2661
AD	1990	DALYs	Number	Female	95+	333	175	550
AD	1990	DALYs	Number	Male	<5	541526	279671	901049
AD	1990	DALYs	Number	Male	5-9	555373	287950	941865
AD	1990	DALYs	Number	Male	10-14	307307	155455	522646
AD	1990	DALYs	Number	Male	15-19	179108	92210	300104

AD	1990	DALYs	Number	Male	20-24	111406	57569	186256
AD	1990	DALYs	Number	Male	25-29	80451	41560	139413
AD	1990	DALYs	Number	Male	30-34	63171	32092	104434
AD	1990	DALYs	Number	Male	35-39	52838	26276	88252
AD	1990	DALYs	Number	Male	40-44	43289	22259	74659
AD	1990	DALYs	Number	Male	45-49	34666	17581	58738
AD	1990	DALYs	Number	Male	50-54	31626	16256	54556
AD	1990	DALYs	Number	Male	55-59	28234	14923	49584
AD	1990	DALYs	Number	Male	60-64	25375	13838	42573
AD	1990	DALYs	Number	Male	65-69	20394	10866	34127
AD	1990	DALYs	Number	Male	70-74	15103	8153	25333
AD	1990	DALYs	Number	Male	75-79	12160	6281	20388
AD	1990	DALYs	Number	Male	80-84	6929	3627	11909
AD	1990	DALYs	Number	Male	85-89	2585	1376	4467
AD	1990	DALYs	Number	Male	90-94	578	310	957
AD	1990	DALYs	Number	Male	95+	104	54	171
AD	1990	DALYs	Rate	Female	<5	196.44	101.91	326.07
AD	1990	DALYs	Rate	Female	5-9	220.69	113.97	374.24
AD	1990	DALYs	Rate	Female	10-14	140.84	71.51	242.48
AD	1990	DALYs	Rate	Female	15-19	92.74	48.24	154.61
AD	1990	DALYs	Rate	Female	20-24	66.6	34.28	110.71
AD	1990	DALYs	Rate	Female	25-29	55.16	28.45	95.42
AD	1990	DALYs	Rate	Female	30-34	48.27	24.85	79.69
AD	1990	DALYs	Rate	Female	35-39	41.99	21.35	70.40
AD	1990	DALYs	Rate	Female	40-44	40.56	20.80	68.87
AD	1990	DALYs	Rate	Female	45-49	40.03	20.35	67.40

AD	1990	DALYs	Rate	Female	50-54	41.09	20.84	71.12
AD	1990	DALYs	Rate	Female	55-59	41.8	22.06	72.84
AD	1990	DALYs	Rate	Female	60-64	43.78	23.79	74.11
AD	1990	DALYs	Rate	Female	65-69	46.32	24.47	76.86
AD	1990	DALYs	Rate	Female	70-74	49.79	26.64	82.14
AD	1990	DALYs	Rate	Female	75-79	56.36	29.15	94.78
AD	1990	DALYs	Rate	Female	80-84	59.39	31.61	100.51
AD	1990	DALYs	Rate	Female	85-89	57.86	31.34	98.53
AD	1990	DALYs	Rate	Female	90-94	52.21	28.24	87.93
AD	1990	DALYs	Rate	Female	95+	43.89	23.04	72.61
AD	1990	DALYs	Rate	Male	<5	169.47	87.52	281.99
AD	1990	DALYs	Rate	Male	5-9	185.31	96.08	314.27
AD	1990	DALYs	Rate	Male	10-14	112.05	56.68	190.57
AD	1990	DALYs	Rate	Male	15-19	67.87	34.94	113.73
AD	1990	DALYs	Rate	Male	20-24	44.93	23.22	75.12
AD	1990	DALYs	Rate	Male	25-29	36.15	18.68	62.65
AD	1990	DALYs	Rate	Male	30-34	32.34	16.43	53.47
AD	1990	DALYs	Rate	Male	35-39	29.55	14.70	49.36
AD	1990	DALYs	Rate	Male	40-44	29.6	15.22	51.05
AD	1990	DALYs	Rate	Male	45-49	29.28	14.85	49.61
AD	1990	DALYs	Rate	Male	50-54	29.38	15.10	50.68
AD	1990	DALYs	Rate	Male	55-59	30.4	16.07	53.38
AD	1990	DALYs	Rate	Male	60-64	32.31	17.62	54.20
AD	1990	DALYs	Rate	Male	65-69	35.57	18.95	59.53
AD	1990	DALYs	Rate	Male	70-74	40.15	21.67	67.34
AD	1990	DALYs	Rate	Male	75-79	48.19	24.89	80.80

AD	1990	DALYs	Rate	Male	80-84	52.17	27.31	89.65
AD	1990	DALYs	Rate	Male	85-89	51.05	27.17	88.20
AD	1990	DALYs	Rate	Male	90-94	45.94	24.59	75.98
AD	1990	DALYs	Rate	Male	95+	39.94	20.93	65.64
AD	1990	Prevalence	Number	Female	<5	13344436	12646597	14037774
AD	1990	Prevalence	Number	Female	5-9	14155623	13151595	15262146
AD	1990	Prevalence	Number	Female	10-14	8365710	7639823	9132200
AD	1990	Prevalence	Number	Female	15-19	5439508	4924891	5959152
AD	1990	Prevalence	Number	Female	20-24	3755475	3409209	4130061
AD	1990	Prevalence	Number	Female	25-29	2815175	2560050	3112887
AD	1990	Prevalence	Number	Female	30-34	2136713	1930143	2339580
AD	1990	Prevalence	Number	Female	35-39	1698954	1544689	1866024
AD	1990	Prevalence	Number	Female	40-44	1334833	1200475	1483132
AD	1990	Prevalence	Number	Female	45-49	1073998	970626	1190027
AD	1990	Prevalence	Number	Female	50-54	1022552	928079	1128176
AD	1990	Prevalence	Number	Female	55-59	921844	837893	1022294
AD	1990	Prevalence	Number	Female	60-64	869505	786107	958215
AD	1990	Prevalence	Number	Female	65-69	753890	682901	829268
AD	1990	Prevalence	Number	Female	70-74	582964	523975	642856
AD	1990	Prevalence	Number	Female	75-79	519005	464509	579063
AD	1990	Prevalence	Number	Female	80-84	338449	304864	372943
AD	1990	Prevalence	Number	Female	85-89	153762	138068	170303
AD	1990	Prevalence	Number	Female	90-94	42786	38738	47179
AD	1990	Prevalence	Number	Female	95+	9202	8083	10334
AD	1990	Prevalence	Number	Male	<5	12270669	11654295	12938270
AD	1990	Prevalence	Number	Male	5-9	12552392	11735202	13499365

AD	1990	Prevalence	Number	Male	10-14	6959633	6419998	7551709
AD	1990	Prevalence	Number	Male	15-19	4075840	3711967	4455434
AD	1990	Prevalence	Number	Male	20-24	2543161	2312405	2789246
AD	1990	Prevalence	Number	Male	25-29	1838201	1674291	2050959
AD	1990	Prevalence	Number	Male	30-34	1448143	1309407	1594643
AD	1990	Prevalence	Number	Male	35-39	1214227	1100334	1337903
AD	1990	Prevalence	Number	Male	40-44	1000223	890462	1111542
AD	1990	Prevalence	Number	Male	45-49	805167	727615	896476
AD	1990	Prevalence	Number	Male	50-54	739466	671029	817892
AD	1990	Prevalence	Number	Male	55-59	666602	604685	738651
AD	1990	Prevalence	Number	Male	60-64	608057	547384	670673
AD	1990	Prevalence	Number	Male	65-69	496543	444808	547020
AD	1990	Prevalence	Number	Male	70-74	373955	335788	413406
AD	1990	Prevalence	Number	Male	75-79	306784	274191	342961
AD	1990	Prevalence	Number	Male	80-84	178042	160959	195575
AD	1990	Prevalence	Number	Male	85-89	67855	60983	75147
AD	1990	Prevalence	Number	Male	90-94	15485	14064	17107
AD	1990	Prevalence	Number	Male	95+	2837	2504	3175
AD	1990	Prevalence	Rate	Female	<5	4442.21	4209.91	4673.02
AD	1990	Prevalence	Rate	Female	5-9	4987.39	4633.64	5377.24
AD	1990	Prevalence	Rate	Female	10-14	3199.98	2922.32	3493.17
AD	1990	Prevalence	Rate	Female	15-19	2128.64	1927.26	2331.99
AD	1990	Prevalence	Rate	Female	20-24	1538.28	1396.44	1691.71
AD	1990	Prevalence	Rate	Female	25-29	1279.05	1163.13	1414.31
AD	1990	Prevalence	Rate	Female	30-34	1123.94	1015.28	1230.65
AD	1990	Prevalence	Rate	Female	35-39	979.49	890.55	1075.81

AD	1990	Prevalence	Rate	Female	40-44	951.92	856.10	1057.68
AD	1990	Prevalence	Rate	Female	45-49	943.75	852.92	1045.71
AD	1990	Prevalence	Rate	Female	50-54	974.63	884.59	1075.31
AD	1990	Prevalence	Rate	Female	55-59	998.62	907.68	1107.43
AD	1990	Prevalence	Rate	Female	60-64	1059.56	957.93	1167.66
AD	1990	Prevalence	Rate	Female	65-69	1137.46	1030.36	1251.19
AD	1990	Prevalence	Rate	Female	70-74	1239.23	1113.83	1366.54
AD	1990	Prevalence	Rate	Female	75-79	1428.87	1278.84	1594.22
AD	1990	Prevalence	Rate	Female	80-84	1531.97	1379.94	1688.10
AD	1990	Prevalence	Rate	Female	85-89	1530.43	1374.22	1695.06
AD	1990	Prevalence	Rate	Female	90-94	1413.83	1280.06	1558.99
AD	1990	Prevalence	Rate	Female	95+	1214.18	1066.58	1363.62
AD	1990	Prevalence	Rate	Male	<5	3840.14	3647.24	4049.06
AD	1990	Prevalence	Rate	Male	5-9	4188.29	3915.62	4504.26
AD	1990	Prevalence	Rate	Male	10-14	2537.68	2340.91	2753.56
AD	1990	Prevalence	Rate	Male	15-19	1544.55	1406.66	1688.40
AD	1990	Prevalence	Rate	Male	20-24	1025.66	932.60	1124.91
AD	1990	Prevalence	Rate	Male	25-29	826.08	752.42	921.70
AD	1990	Prevalence	Rate	Male	30-34	741.45	670.41	816.45
AD	1990	Prevalence	Rate	Male	35-39	679.13	615.43	748.30
AD	1990	Prevalence	Rate	Male	40-44	683.88	608.84	760.00
AD	1990	Prevalence	Rate	Male	45-49	680.07	614.57	757.19
AD	1990	Prevalence	Rate	Male	50-54	686.89	623.32	759.74
AD	1990	Prevalence	Rate	Male	55-59	717.64	650.98	795.21
AD	1990	Prevalence	Rate	Male	60-64	774.14	696.90	853.86
AD	1990	Prevalence	Rate	Male	65-69	866.1	775.86	954.14

AD	1990	Prevalence	Rate	Male	70-74	994.07	892.61	1098.94
AD	1990	Prevalence	Rate	Male	75-79	1215.81	1086.64	1359.18
AD	1990	Prevalence	Rate	Male	80-84	1340.33	1211.73	1472.32
AD	1990	Prevalence	Rate	Male	85-89	1339.93	1204.23	1483.93
AD	1990	Prevalence	Rate	Male	90-94	1229.95	1117.08	1358.80
AD	1990	Prevalence	Rate	Male	95+	1090.07	962.14	1219.99
AD	1990	Incidence	Number	Female	<5	3616311	3350352	3897276
AD	1990	Incidence	Number	Female	5-9	1079073	893948	1273615
AD	1990	Incidence	Number	Female	10-14	495390	389749	621665
AD	1990	Incidence	Number	Female	15-19	340256	271500	416441
AD	1990	Incidence	Number	Female	20-24	270240	215389	326793
AD	1990	Incidence	Number	Female	25-29	226828	186887	272548
AD	1990	Incidence	Number	Female	30-34	183640	147910	219865
AD	1990	Incidence	Number	Female	35-39	169263	138757	201479
AD	1990	Incidence	Number	Female	40-44	153264	129636	180482
AD	1990	Incidence	Number	Female	45-49	130137	109387	151826
AD	1990	Incidence	Number	Female	50-54	125189	106881	146042
AD	1990	Incidence	Number	Female	55-59	114632	97799	133753
AD	1990	Incidence	Number	Female	60-64	108890	92064	128328
AD	1990	Incidence	Number	Female	65-69	97535	82061	115643
AD	1990	Incidence	Number	Female	70-74	74775	62770	86666
AD	1990	Incidence	Number	Female	75-79	60278	51298	70783
AD	1990	Incidence	Number	Female	80-84	33590	28162	39444
AD	1990	Incidence	Number	Female	85-89	12503	10277	14954
AD	1990	Incidence	Number	Female	90-94	3109	2468	3824
AD	1990	Incidence	Number	Female	95+	730	518	1031

AD	1990	Incidence	Number	Male	<5	3245966	3015419	3499986
AD	1990	Incidence	Number	Male	5-9	915381	760405	1095405
AD	1990	Incidence	Number	Male	10-14	387100	305023	481409
AD	1990	Incidence	Number	Male	15-19	250012	198281	306225
AD	1990	Incidence	Number	Male	20-24	200583	160580	241653
AD	1990	Incidence	Number	Male	25-29	177958	148387	211463
AD	1990	Incidence	Number	Male	30-34	152791	123866	180522
AD	1990	Incidence	Number	Male	35-39	143894	120020	169344
AD	1990	Incidence	Number	Male	40-44	130216	110571	152109
AD	1990	Incidence	Number	Male	45-49	108341	91826	126131
AD	1990	Incidence	Number	Male	50-54	102640	87923	118687
AD	1990	Incidence	Number	Male	55-59	94181	80487	109042
AD	1990	Incidence	Number	Male	60-64	85959	72305	101027
AD	1990	Incidence	Number	Male	65-69	71184	59904	83795
AD	1990	Incidence	Number	Male	70-74	53100	45297	61225
AD	1990	Incidence	Number	Male	75-79	38692	33030	44997
AD	1990	Incidence	Number	Male	80-84	18487	15537	21754
AD	1990	Incidence	Number	Male	85-89	5693	4674	6756
AD	1990	Incidence	Number	Male	90-94	1170	936	1437
AD	1990	Incidence	Number	Male	95+	231	164	326
AD	1990	Incidence	Rate	Female	<5	1203.83	1115.29	1297.36
AD	1990	Incidence	Rate	Female	5-9	380.18	314.96	448.73
AD	1990	Incidence	Rate	Female	10-14	189.49	149.08	237.79
AD	1990	Incidence	Rate	Female	15-19	133.15	106.25	162.97
AD	1990	Incidence	Rate	Female	20-24	110.69	88.23	133.86
AD	1990	Incidence	Rate	Female	25-29	103.06	84.91	123.83

AD	1990	Incidence	Rate	Female	30-34	96.6	77.80	115.65
AD	1990	Incidence	Rate	Female	35-39	97.58	80.00	116.16
AD	1990	Incidence	Rate	Female	40-44	109.3	92.45	128.71
AD	1990	Incidence	Rate	Female	45-49	114.36	96.12	133.41
AD	1990	Incidence	Rate	Female	50-54	119.32	101.87	139.20
AD	1990	Incidence	Rate	Female	55-59	124.18	105.94	144.89
AD	1990	Incidence	Rate	Female	60-64	132.69	112.19	156.38
AD	1990	Incidence	Rate	Female	65-69	147.16	123.81	174.48
AD	1990	Incidence	Rate	Female	70-74	158.95	133.43	184.23
AD	1990	Incidence	Rate	Female	75-79	165.95	141.23	194.87
AD	1990	Incidence	Rate	Female	80-84	152.04	127.47	178.54
AD	1990	Incidence	Rate	Female	85-89	124.45	102.29	148.84
AD	1990	Incidence	Rate	Female	90-94	102.72	81.54	126.35
AD	1990	Incidence	Rate	Female	95+	96.34	68.34	136.00
AD	1990	Incidence	Rate	Male	<5	1015.83	943.68	1095.33
AD	1990	Incidence	Rate	Male	5-9	305.43	253.72	365.50
AD	1990	Incidence	Rate	Male	10-14	141.15	111.22	175.53
AD	1990	Incidence	Rate	Male	15-19	94.74	75.14	116.04
AD	1990	Incidence	Rate	Male	20-24	80.9	64.76	97.46
AD	1990	Incidence	Rate	Male	25-29	79.97	66.68	95.03
AD	1990	Incidence	Rate	Male	30-34	78.23	63.42	92.43
AD	1990	Incidence	Rate	Male	35-39	80.48	67.13	94.72
AD	1990	Incidence	Rate	Male	40-44	89.03	75.60	104.00
AD	1990	Incidence	Rate	Male	45-49	91.51	77.56	106.53
AD	1990	Incidence	Rate	Male	50-54	95.34	81.67	110.25
AD	1990	Incidence	Rate	Male	55-59	101.39	86.65	117.39

AD	1990	Incidence	Rate	Male	60-64	109.44	92.05	128.62
AD	1990	Incidence	Rate	Male	65-69	124.16	104.49	146.16
AD	1990	Incidence	Rate	Male	70-74	141.15	120.41	162.75
AD	1990	Incidence	Rate	Male	75-79	153.34	130.90	178.33
AD	1990	Incidence	Rate	Male	80-84	139.18	116.97	163.77
AD	1990	Incidence	Rate	Male	85-89	112.42	92.30	133.41
AD	1990	Incidence	Rate	Male	90-94	92.97	74.33	114.14
AD	1990	Incidence	Rate	Male	95+	88.82	62.84	125.43
AD	2021	DALYs	Number	Female	<5	591956	304720	987483
AD	2021	DALYs	Number	Female	5-9	681921	351887	1152124
AD	2021	DALYs	Number	Female	10-14	421238	213993	720805
AD	2021	DALYs	Number	Female	15-19	259064	135295	428196
AD	2021	DALYs	Number	Female	20-24	177575	92491	294676
AD	2021	DALYs	Number	Female	25-29	141333	72023	244605
AD	2021	DALYs	Number	Female	30-34	124002	63568	208776
AD	2021	DALYs	Number	Female	35-39	101880	51447	170640
AD	2021	DALYs	Number	Female	40-44	86577	44110	146594
AD	2021	DALYs	Number	Female	45-49	82357	42109	138080
AD	2021	DALYs	Number	Female	50-54	80055	40526	139370
AD	2021	DALYs	Number	Female	55-59	75584	39738	132344
AD	2021	DALYs	Number	Female	60-64	65781	36366	109686
AD	2021	DALYs	Number	Female	65-69	58951	31005	98863
AD	2021	DALYs	Number	Female	70-74	49801	26643	83835
AD	2021	DALYs	Number	Female	75-79	35161	18160	58572
AD	2021	DALYs	Number	Female	80-84	26181	13713	44460
AD	2021	DALYs	Number	Female	85-89	14513	7731	25142

AD	2021	DALYs	Number	Female	90-94	5683	3058	9480
AD	2021	DALYs	Number	Female	95+	1631	855	2708
AD	2021	DALYs	Number	Male	<5	544307	280471	911456
AD	2021	DALYs	Number	Male	5-9	610269	315608	1015859
AD	2021	DALYs	Number	Male	10-14	359471	180094	618320
AD	2021	DALYs	Number	Male	15-19	204217	105490	339885
AD	2021	DALYs	Number	Male	20-24	128542	66880	217415
AD	2021	DALYs	Number	Male	25-29	98705	50496	171953
AD	2021	DALYs	Number	Male	30-34	88077	44604	147839
AD	2021	DALYs	Number	Male	35-39	75517	37885	128936
AD	2021	DALYs	Number	Male	40-44	66266	33728	115637
AD	2021	DALYs	Number	Male	45-49	63198	31796	106535
AD	2021	DALYs	Number	Male	50-54	60618	31050	105342
AD	2021	DALYs	Number	Male	55-59	56062	29849	98302
AD	2021	DALYs	Number	Male	60-64	48232	26467	81356
AD	2021	DALYs	Number	Male	65-69	43622	22978	72466
AD	2021	DALYs	Number	Male	70-74	37412	19744	63896
AD	2021	DALYs	Number	Male	75-79	26205	13603	43467
AD	2021	DALYs	Number	Male	80-84	17778	9308	30410
AD	2021	DALYs	Number	Male	85-89	8487	4494	14586
AD	2021	DALYs	Number	Male	90-94	2667	1439	4454
AD	2021	DALYs	Number	Male	95+	608	322	999
AD	2021	DALYs	Rate	Female	<5	186.04	95.77	310.34
AD	2021	DALYs	Rate	Female	5-9	205.04	105.81	346.42
AD	2021	DALYs	Rate	Female	10-14	130.44	66.27	223.21
AD	2021	DALYs	Rate	Female	15-19	85.32	44.56	141.02

AD	2021	DALYs	Rate	Female	20-24	60.45	31.49	100.31
AD	2021	DALYs	Rate	Female	25-29	48.57	24.75	84.06
AD	2021	DALYs	Rate	Female	30-34	41.48	21.27	69.84
AD	2021	DALYs	Rate	Female	35-39	36.67	18.52	61.42
AD	2021	DALYs	Rate	Female	40-44	34.9	17.78	59.09
AD	2021	DALYs	Rate	Female	45-49	34.95	17.87	58.60
AD	2021	DALYs	Rate	Female	50-54	35.91	18.18	62.51
AD	2021	DALYs	Rate	Female	55-59	37.6	19.77	65.84
AD	2021	DALYs	Rate	Female	60-64	39.99	22.11	66.67
AD	2021	DALYs	Rate	Female	65-69	40.94	21.53	68.65
AD	2021	DALYs	Rate	Female	70-74	45.5	24.34	76.60
AD	2021	DALYs	Rate	Female	75-79	48.77	25.19	81.24
AD	2021	DALYs	Rate	Female	80-84	51.4	26.93	87.29
AD	2021	DALYs	Rate	Female	85-89	50.98	27.16	88.31
AD	2021	DALYs	Rate	Female	90-94	47.12	25.35	78.60
AD	2021	DALYs	Rate	Female	95+	41.41	21.71	68.76
AD	2021	DALYs	Rate	Male	<5	160.1	82.50	268.09
AD	2021	DALYs	Rate	Male	5-9	172.16	89.03	286.58
AD	2021	DALYs	Rate	Male	10-14	104.59	52.40	179.90
AD	2021	DALYs	Rate	Male	15-19	63.75	32.93	106.10
AD	2021	DALYs	Rate	Male	20-24	42.37	22.04	71.66
AD	2021	DALYs	Rate	Male	25-29	33.19	16.98	57.83
AD	2021	DALYs	Rate	Male	30-34	28.83	14.60	48.38
AD	2021	DALYs	Rate	Male	35-39	26.68	13.38	45.55
AD	2021	DALYs	Rate	Male	40-44	26.28	13.38	45.86
AD	2021	DALYs	Rate	Male	45-49	26.57	13.37	44.79

AD	2021	DALYs	Rate	Male	50-54	27.31	13.99	47.46
AD	2021	DALYs	Rate	Male	55-59	28.79	15.33	50.48
AD	2021	DALYs	Rate	Male	60-64	31.01	17.02	52.31
AD	2021	DALYs	Rate	Male	65-69	33.09	17.43	54.97
AD	2021	DALYs	Rate	Male	70-74	38.81	20.48	66.29
AD	2021	DALYs	Rate	Male	75-79	43.83	22.75	72.70
AD	2021	DALYs	Rate	Male	80-84	48.51	25.39	82.97
AD	2021	DALYs	Rate	Male	85-89	49.19	26.05	84.54
AD	2021	DALYs	Rate	Male	90-94	45.76	24.69	76.42
AD	2021	DALYs	Rate	Male	95+	40.24	21.27	66.04
AD	2021	Prevalence	Number	Female	<5	13338454	12653193	14029594
AD	2021	Prevalence	Number	Female	5-9	15379041	14325460	16568844
AD	2021	Prevalence	Number	Female	10-14	9568784	8757298	10418516
AD	2021	Prevalence	Number	Female	15-19	5948980	5404529	6526978
AD	2021	Prevalence	Number	Female	20-24	4106922	3708391	4507857
AD	2021	Prevalence	Number	Female	25-29	3277539	2980030	3644991
AD	2021	Prevalence	Number	Female	30-34	2886117	2604450	3171438
AD	2021	Prevalence	Number	Female	35-39	2379182	2143892	2619696
AD	2021	Prevalence	Number	Female	40-44	2033610	1812225	2265700
AD	2021	Prevalence	Number	Female	45-49	1940896	1758397	2155337
AD	2021	Prevalence	Number	Female	50-54	1896043	1714216	2096188
AD	2021	Prevalence	Number	Female	55-59	1805489	1644356	2003915
AD	2021	Prevalence	Number	Female	60-64	1595751	1442424	1759750
AD	2021	Prevalence	Number	Female	65-69	1448528	1313565	1602431
AD	2021	Prevalence	Number	Female	70-74	1243499	1117233	1374676
AD	2021	Prevalence	Number	Female	75-79	892551	800875	996903

AD	2021	Prevalence	Number	Female	80-84	676570	610674	746295
AD	2021	Prevalence	Number	Female	85-89	383998	344524	427021
AD	2021	Prevalence	Number	Female	90-94	153760	138670	169978
AD	2021	Prevalence	Number	Female	95+	45195	39615	50560
AD	2021	Prevalence	Number	Male	<5	12267813	11657279	12915614
AD	2021	Prevalence	Number	Male	5-9	13760352	12827950	14837174
AD	2021	Prevalence	Number	Male	10-14	8121670	7476448	8834832
AD	2021	Prevalence	Number	Male	15-19	4640844	4224344	5061940
AD	2021	Prevalence	Number	Male	20-24	2933066	2660900	3232360
AD	2021	Prevalence	Number	Male	25-29	2250644	2042112	2503130
AD	2021	Prevalence	Number	Male	30-34	2011958	1816772	2214011
AD	2021	Prevalence	Number	Male	35-39	1731796	1565999	1914272
AD	2021	Prevalence	Number	Male	40-44	1527496	1364993	1709476
AD	2021	Prevalence	Number	Male	45-49	1464670	1323262	1632429
AD	2021	Prevalence	Number	Male	50-54	1415560	1279417	1571095
AD	2021	Prevalence	Number	Male	55-59	1322113	1198756	1462354
AD	2021	Prevalence	Number	Male	60-64	1156171	1039022	1277566
AD	2021	Prevalence	Number	Male	65-69	1061037	955188	1172689
AD	2021	Prevalence	Number	Male	70-74	925842	832953	1024607
AD	2021	Prevalence	Number	Male	75-79	660040	591259	734771
AD	2021	Prevalence	Number	Male	80-84	456152	413099	502817
AD	2021	Prevalence	Number	Male	85-89	222341	199831	246680
AD	2021	Prevalence	Number	Male	90-94	71576	64816	79048
AD	2021	Prevalence	Number	Male	95+	16621	14592	18651
AD	2021	Prevalence	Rate	Female	<5	4191.95	3976.58	4409.15
AD	2021	Prevalence	Rate	Female	5-9	4624.2	4307.41	4981.96

AD	2021	Prevalence	Rate	Female	10-14	2963.15	2711.86	3226.29
AD	2021	Prevalence	Rate	Female	15-19	1959.16	1779.85	2149.51
AD	2021	Prevalence	Rate	Female	20-24	1398.09	1262.42	1534.58
AD	2021	Prevalence	Rate	Female	25-29	1126.35	1024.11	1252.63
AD	2021	Prevalence	Rate	Female	30-34	965.48	871.26	1060.93
AD	2021	Prevalence	Rate	Female	35-39	856.43	771.73	943.01
AD	2021	Prevalence	Rate	Female	40-44	819.71	730.47	913.26
AD	2021	Prevalence	Rate	Female	45-49	823.66	746.21	914.66
AD	2021	Prevalence	Rate	Female	50-54	850.46	768.90	940.24
AD	2021	Prevalence	Rate	Female	55-59	898.24	818.07	996.96
AD	2021	Prevalence	Rate	Female	60-64	970	876.80	1069.69
AD	2021	Prevalence	Rate	Female	65-69	1005.86	912.14	1112.73
AD	2021	Prevalence	Rate	Female	70-74	1136.16	1020.79	1256.01
AD	2021	Prevalence	Rate	Female	75-79	1237.97	1110.82	1382.71
AD	2021	Prevalence	Rate	Female	80-84	1328.4	1199.02	1465.30
AD	2021	Prevalence	Rate	Female	85-89	1348.82	1210.17	1499.95
AD	2021	Prevalence	Rate	Female	90-94	1274.86	1149.75	1409.34
AD	2021	Prevalence	Rate	Female	95+	1147.59	1005.89	1283.82
AD	2021	Prevalence	Rate	Male	<5	3608.4	3428.82	3798.94
AD	2021	Prevalence	Rate	Male	5-9	3881.88	3618.84	4185.66
AD	2021	Prevalence	Rate	Male	10-14	2362.94	2175.22	2570.43
AD	2021	Prevalence	Rate	Male	15-19	1448.77	1318.75	1580.23
AD	2021	Prevalence	Rate	Male	20-24	966.71	877.01	1065.36
AD	2021	Prevalence	Rate	Male	25-29	756.89	686.76	841.80
AD	2021	Prevalence	Rate	Male	30-34	658.47	594.59	724.60
AD	2021	Prevalence	Rate	Male	35-39	611.81	553.23	676.27

AD	2021	Prevalence	Rate	Male	40-44	605.76	541.32	677.93
AD	2021	Prevalence	Rate	Male	45-49	615.76	556.31	686.29
AD	2021	Prevalence	Rate	Male	50-54	637.7	576.36	707.76
AD	2021	Prevalence	Rate	Male	55-59	678.97	615.62	750.99
AD	2021	Prevalence	Rate	Male	60-64	743.34	668.02	821.39
AD	2021	Prevalence	Rate	Male	65-69	804.83	724.54	889.52
AD	2021	Prevalence	Rate	Male	70-74	960.5	864.14	1062.96
AD	2021	Prevalence	Rate	Male	75-79	1103.99	988.95	1228.99
AD	2021	Prevalence	Rate	Male	80-84	1244.55	1127.09	1371.87
AD	2021	Prevalence	Rate	Male	85-89	1288.74	1158.26	1429.81
AD	2021	Prevalence	Rate	Male	90-94	1228.03	1112.06	1356.23
AD	2021	Prevalence	Rate	Male	95+	1099.21	965.06	1233.50
AD	2021	Incidence	Number	Female	<5	3632263	3360023	3908489
AD	2021	Incidence	Number	Female	5-9	1201574	999134	1416472
AD	2021	Incidence	Number	Female	10-14	581302	460707	728816
AD	2021	Incidence	Number	Female	15-19	370406	291531	454674
AD	2021	Incidence	Number	Female	20-24	293053	231700	355565
AD	2021	Incidence	Number	Female	25-29	271503	222009	326349
AD	2021	Incidence	Number	Female	30-34	262462	211745	313763
AD	2021	Incidence	Number	Female	35-39	249108	205540	297332
AD	2021	Incidence	Number	Female	40-44	246308	209766	289062
AD	2021	Incidence	Number	Female	45-49	246304	207539	286106
AD	2021	Incidence	Number	Female	50-54	245524	209826	285850
AD	2021	Incidence	Number	Female	55-59	235690	201235	274266
AD	2021	Incidence	Number	Female	60-64	206899	173187	242438
AD	2021	Incidence	Number	Female	65-69	192761	163176	227093

AD	2021	Incidence	Number	Female	70-74	162406	137295	188149
AD	2021	Incidence	Number	Female	75-79	108241	92170	127000
AD	2021	Incidence	Number	Female	80-84	70931	59492	83271
AD	2021	Incidence	Number	Female	85-89	33143	27198	39254
AD	2021	Incidence	Number	Female	90-94	11835	9406	14498
AD	2021	Incidence	Number	Female	95+	3714	2637	5226
AD	2021	Incidence	Number	Male	<5	3275949	3048975	3527685
AD	2021	Incidence	Number	Male	5-9	1036570	858685	1228755
AD	2021	Incidence	Number	Male	10-14	476215	377884	592596
AD	2021	Incidence	Number	Male	15-19	292646	233354	358718
AD	2021	Incidence	Number	Male	20-24	231677	184818	280996
AD	2021	Incidence	Number	Male	25-29	221182	182456	263226
AD	2021	Incidence	Number	Male	30-34	221226	180234	260734
AD	2021	Incidence	Number	Male	35-39	212732	175583	252595
AD	2021	Incidence	Number	Male	40-44	207195	176888	242090
AD	2021	Incidence	Number	Male	45-49	203871	173086	236610
AD	2021	Incidence	Number	Male	50-54	201654	171812	232527
AD	2021	Incidence	Number	Male	55-59	190762	162484	221937
AD	2021	Incidence	Number	Male	60-64	165297	138953	194654
AD	2021	Incidence	Number	Male	65-69	153770	129994	181152
AD	2021	Incidence	Number	Male	70-74	131581	112193	152079
AD	2021	Incidence	Number	Male	75-79	84650	71922	98687
AD	2021	Incidence	Number	Male	80-84	48565	40921	57093
AD	2021	Incidence	Number	Male	85-89	18951	15632	22421
AD	2021	Incidence	Number	Male	90-94	5418	4324	6670
AD	2021	Incidence	Number	Male	95+	1341	949	1889

AD	2021	Incidence	Rate	Female	<5	1141.53	1055.97	1228.34
AD	2021	Incidence	Rate	Female	5-9	361.29	300.42	425.91
AD	2021	Incidence	Rate	Female	10-14	180.01	142.67	225.69
AD	2021	Incidence	Rate	Female	15-19	121.98	96.01	149.74
AD	2021	Incidence	Rate	Female	20-24	99.76	78.88	121.04
AD	2021	Incidence	Rate	Female	25-29	93.3	76.30	112.15
AD	2021	Incidence	Rate	Female	30-34	87.8	70.83	104.96
AD	2021	Incidence	Rate	Female	35-39	89.67	73.99	107.03
AD	2021	Incidence	Rate	Female	40-44	99.28	84.55	116.52
AD	2021	Incidence	Rate	Female	45-49	104.52	88.07	121.42
AD	2021	Incidence	Rate	Female	50-54	110.13	94.12	128.22
AD	2021	Incidence	Rate	Female	55-59	117.26	100.12	136.45
AD	2021	Incidence	Rate	Female	60-64	125.77	105.27	147.37
AD	2021	Incidence	Rate	Female	65-69	133.85	113.31	157.69
AD	2021	Incidence	Rate	Female	70-74	148.39	125.44	171.91
AD	2021	Incidence	Rate	Female	75-79	150.13	127.84	176.15
AD	2021	Incidence	Rate	Female	80-84	139.27	116.81	163.50
AD	2021	Incidence	Rate	Female	85-89	116.42	95.54	137.88
AD	2021	Incidence	Rate	Female	90-94	98.13	77.99	120.20
AD	2021	Incidence	Rate	Female	95+	94.31	66.96	132.70
AD	2021	Incidence	Rate	Male	<5	963.57	896.81	1037.62
AD	2021	Incidence	Rate	Male	5-9	292.42	242.24	346.64
AD	2021	Incidence	Rate	Male	10-14	138.55	109.94	172.41
AD	2021	Incidence	Rate	Male	15-19	91.36	72.85	111.98
AD	2021	Incidence	Rate	Male	20-24	76.36	60.91	92.61
AD	2021	Incidence	Rate	Male	25-29	74.38	61.36	88.52

AD	2021	Incidence	Rate	Male	30-34	72.4	58.99	85.33
AD	2021	Incidence	Rate	Male	35-39	75.15	62.03	89.24
AD	2021	Incidence	Rate	Male	40-44	82.17	70.15	96.01
AD	2021	Incidence	Rate	Male	45-49	85.71	72.77	99.47
AD	2021	Incidence	Rate	Male	50-54	90.84	77.40	104.75
AD	2021	Incidence	Rate	Male	55-59	97.97	83.44	113.97
AD	2021	Incidence	Rate	Male	60-64	106.27	89.34	125.15
AD	2021	Incidence	Rate	Male	65-69	116.64	98.60	137.41
AD	2021	Incidence	Rate	Male	70-74	136.51	116.39	157.77
AD	2021	Incidence	Rate	Male	75-79	141.59	120.30	165.07
AD	2021	Incidence	Rate	Male	80-84	132.5	111.65	155.77
AD	2021	Incidence	Rate	Male	85-89	109.84	90.60	129.96
AD	2021	Incidence	Rate	Male	90-94	92.96	74.19	114.43
AD	2021	Incidence	Rate	Male	95+	88.68	62.74	124.95
CD	1990	DALYs	Number	Female	<5	0	0	0
CD	1990	DALYs	Number	Female	5-9	6080	2431	12267
CD	1990	DALYs	Number	Female	10-14	29052	13715	56370
CD	1990	DALYs	Number	Female	15-19	55467	26316	103610
CD	1990	DALYs	Number	Female	20-24	71925	34871	136836
CD	1990	DALYs	Number	Female	25-29	77684	38065	144952
CD	1990	DALYs	Number	Female	30-34	70487	34526	120957
CD	1990	DALYs	Number	Female	35-39	66279	31850	120349
CD	1990	DALYs	Number	Female	40-44	59669	27932	116917
CD	1990	DALYs	Number	Female	45-49	53878	24791	103816
CD	1990	DALYs	Number	Female	50-54	52608	25858	94146
CD	1990	DALYs	Number	Female	55-59	48860	24433	87151

CD	1990	DALYs	Number	Female	60-64	45964	21693	84584
CD	1990	DALYs	Number	Female	65-69	37149	17018	71899
CD	1990	DALYs	Number	Female	70-74	26750	13277	50121
CD	1990	DALYs	Number	Female	75-79	20422	8995	35892
CD	1990	DALYs	Number	Female	80-84	11087	5381	20999
CD	1990	DALYs	Number	Female	85-89	4231	2122	7725
CD	1990	DALYs	Number	Female	90-94	1217	595	2195
CD	1990	DALYs	Number	Female	95+	332	142	649
CD	1990	DALYs	Number	Male	<5	0	0	0
CD	1990	DALYs	Number	Male	5-9	5959	2366	11901
CD	1990	DALYs	Number	Male	10-14	26686	12807	51435
CD	1990	DALYs	Number	Male	15-19	48385	23323	91957
CD	1990	DALYs	Number	Male	20-24	60603	29344	113557
CD	1990	DALYs	Number	Male	25-29	64403	30556	124075
CD	1990	DALYs	Number	Male	30-34	58884	27939	100936
CD	1990	DALYs	Number	Male	35-39	55681	26101	102560
CD	1990	DALYs	Number	Male	40-44	50662	23987	99572
CD	1990	DALYs	Number	Male	45-49	45916	21614	88344
CD	1990	DALYs	Number	Male	50-54	43958	21468	78794
CD	1990	DALYs	Number	Male	55-59	40675	20134	73676
CD	1990	DALYs	Number	Male	60-64	36793	17301	67143
CD	1990	DALYs	Number	Male	65-69	27777	12828	54715
CD	1990	DALYs	Number	Male	70-74	19044	9257	36070
CD	1990	DALYs	Number	Male	75-79	13209	5820	23760
CD	1990	DALYs	Number	Male	80-84	6558	3166	12580
CD	1990	DALYs	Number	Male	85-89	2225	1112	4106

CD	1990	DALYs	Number	Male	90-94	547	263	975
CD	1990	DALYs	Number	Male	95+	125	53	246
CD	1990	DALYs	Rate	Female	<5	0	0.00	0.00
CD	1990	DALYs	Rate	Female	5-9	2.14	0.86	4.32
CD	1990	DALYs	Rate	Female	10-14	11.11	5.25	21.56
CD	1990	DALYs	Rate	Female	15-19	21.71	10.30	40.55
CD	1990	DALYs	Rate	Female	20-24	29.46	14.28	56.05
CD	1990	DALYs	Rate	Female	25-29	35.3	17.29	65.86
CD	1990	DALYs	Rate	Female	30-34	37.08	18.16	63.62
CD	1990	DALYs	Rate	Female	35-39	38.21	18.36	69.38
CD	1990	DALYs	Rate	Female	40-44	42.55	19.92	83.38
CD	1990	DALYs	Rate	Female	45-49	47.34	21.78	91.23
CD	1990	DALYs	Rate	Female	50-54	50.14	24.65	89.73
CD	1990	DALYs	Rate	Female	55-59	52.93	26.47	94.41
CD	1990	DALYs	Rate	Female	60-64	56.01	26.44	103.07
CD	1990	DALYs	Rate	Female	65-69	56.05	25.68	108.48
CD	1990	DALYs	Rate	Female	70-74	56.86	28.22	106.54
CD	1990	DALYs	Rate	Female	75-79	56.22	24.77	98.81
CD	1990	DALYs	Rate	Female	80-84	50.19	24.35	95.05
CD	1990	DALYs	Rate	Female	85-89	42.11	21.12	76.89
CD	1990	DALYs	Rate	Female	90-94	40.22	19.66	72.52
CD	1990	DALYs	Rate	Female	95+	43.77	18.76	85.64
CD	1990	DALYs	Rate	Male	<5	0	0.00	0.00
CD	1990	DALYs	Rate	Male	5-9	1.99	0.79	3.97
CD	1990	DALYs	Rate	Male	10-14	9.73	4.67	18.75
CD	1990	DALYs	Rate	Male	15-19	18.34	8.84	34.85

CD	1990	DALYs	Rate	Male	20-24	24.44	11.83	45.80
CD	1990	DALYs	Rate	Male	25-29	28.94	13.73	55.76
CD	1990	DALYs	Rate	Male	30-34	30.15	14.30	51.68
CD	1990	DALYs	Rate	Male	35-39	31.14	14.60	57.36
CD	1990	DALYs	Rate	Male	40-44	34.64	16.40	68.08
CD	1990	DALYs	Rate	Male	45-49	38.78	18.26	74.62
CD	1990	DALYs	Rate	Male	50-54	40.83	19.94	73.19
CD	1990	DALYs	Rate	Male	55-59	43.79	21.68	79.32
CD	1990	DALYs	Rate	Male	60-64	46.84	22.03	85.48
CD	1990	DALYs	Rate	Male	65-69	48.45	22.37	95.44
CD	1990	DALYs	Rate	Male	70-74	50.62	24.61	95.88
CD	1990	DALYs	Rate	Male	75-79	52.35	23.07	94.16
CD	1990	DALYs	Rate	Male	80-84	49.37	23.83	94.70
CD	1990	DALYs	Rate	Male	85-89	43.94	21.96	81.08
CD	1990	DALYs	Rate	Male	90-94	43.49	20.92	77.45
CD	1990	DALYs	Rate	Male	95+	47.9	20.25	94.71
CD	1990	Prevalence	Number	Female	<5	0	0	0
CD	1990	Prevalence	Number	Female	5-9	234128	108631	422590
CD	1990	Prevalence	Number	Female	10-14	1133188	599957	1959857
CD	1990	Prevalence	Number	Female	15-19	2177362	1185851	3807243
CD	1990	Prevalence	Number	Female	20-24	2838977	1524093	4788965
CD	1990	Prevalence	Number	Female	25-29	3079014	1762095	5024126
CD	1990	Prevalence	Number	Female	30-34	2816324	1590736	4443250
CD	1990	Prevalence	Number	Female	35-39	2663587	1456875	4413796
CD	1990	Prevalence	Number	Female	40-44	2404360	1324956	4126149
CD	1990	Prevalence	Number	Female	45-49	2186918	1145516	3722698

CD	1990	Prevalence	Number	Female	50-54	2152890	1226833	3489317
CD	1990	Prevalence	Number	Female	55-59	2014515	1167904	3334750
CD	1990	Prevalence	Number	Female	60-64	1914023	1081758	3269481
CD	1990	Prevalence	Number	Female	65-69	1569500	835368	2712605
CD	1990	Prevalence	Number	Female	70-74	1148604	651657	1981099
CD	1990	Prevalence	Number	Female	75-79	888417	453540	1480331
CD	1990	Prevalence	Number	Female	80-84	490038	272825	870882
CD	1990	Prevalence	Number	Female	85-89	191175	104476	323999
CD	1990	Prevalence	Number	Female	90-94	55976	30571	93690
CD	1990	Prevalence	Number	Female	95+	15642	8277	28050
CD	1990	Prevalence	Number	Male	<5	0	0	0
CD	1990	Prevalence	Number	Male	5-9	229997	109892	420846
CD	1990	Prevalence	Number	Male	10-14	1039740	556192	1782654
CD	1990	Prevalence	Number	Male	15-19	1885994	1055119	3311903
CD	1990	Prevalence	Number	Male	20-24	2365912	1264838	4023455
CD	1990	Prevalence	Number	Male	25-29	2515743	1426996	4137786
CD	1990	Prevalence	Number	Male	30-34	2320422	1314891	3696136
CD	1990	Prevalence	Number	Male	35-39	2204477	1185880	3652731
CD	1990	Prevalence	Number	Male	40-44	2009725	1084856	3497286
CD	1990	Prevalence	Number	Male	45-49	1835195	962880	3164663
CD	1990	Prevalence	Number	Male	50-54	1773941	1000816	2876796
CD	1990	Prevalence	Number	Male	55-59	1658841	966487	2713930
CD	1990	Prevalence	Number	Male	60-64	1519124	854166	2566764
CD	1990	Prevalence	Number	Male	65-69	1164959	614197	2034887
CD	1990	Prevalence	Number	Male	70-74	813233	448240	1387259
CD	1990	Prevalence	Number	Male	75-79	571875	290144	959807

CD	1990	Prevalence	Number	Male	80-84	289054	162411	506459
CD	1990	Prevalence	Number	Male	85-89	100171	54530	169952
CD	1990	Prevalence	Number	Male	90-94	25056	13619	41650
CD	1990	Prevalence	Number	Male	95+	5842	3059	10523
CD	1990	Prevalence	Rate	Female	<5	0	0.00	0.00
CD	1990	Prevalence	Rate	Female	5-9	82.49	38.27	148.89
CD	1990	Prevalence	Rate	Female	10-14	433.46	229.49	749.67
CD	1990	Prevalence	Rate	Female	15-19	852.07	464.06	1489.89
CD	1990	Prevalence	Rate	Female	20-24	1162.87	624.28	1961.61
CD	1990	Prevalence	Rate	Female	25-29	1398.92	800.59	2282.66
CD	1990	Prevalence	Rate	Female	30-34	1481.43	836.75	2337.21
CD	1990	Prevalence	Rate	Female	35-39	1535.62	839.92	2544.66
CD	1990	Prevalence	Rate	Female	40-44	1714.64	944.88	2942.51
CD	1990	Prevalence	Rate	Female	45-49	1921.71	1006.60	3271.24
CD	1990	Prevalence	Rate	Female	50-54	2052	1169.34	3325.79
CD	1990	Prevalence	Rate	Female	55-59	2182.29	1265.17	3612.48
CD	1990	Prevalence	Rate	Female	60-64	2332.38	1318.20	3984.10
CD	1990	Prevalence	Rate	Female	65-69	2368.05	1260.40	4092.75
CD	1990	Prevalence	Rate	Female	70-74	2441.62	1385.25	4211.28
CD	1990	Prevalence	Rate	Female	75-79	2445.9	1248.64	4075.50
CD	1990	Prevalence	Rate	Female	80-84	2218.12	1234.92	3941.98
CD	1990	Prevalence	Rate	Female	85-89	1902.8	1039.87	3224.83
CD	1990	Prevalence	Rate	Female	90-94	1849.7	1010.20	3095.94
CD	1990	Prevalence	Rate	Female	95+	2063.93	1092.21	3701.19
CD	1990	Prevalence	Rate	Male	<5	0	0.00	0.00
CD	1990	Prevalence	Rate	Male	5-9	76.74	36.67	140.42

CD	1990	Prevalence	Rate	Male	10-14	379.12	202.80	650.01
CD	1990	Prevalence	Rate	Male	15-19	714.7	399.84	1255.06
CD	1990	Prevalence	Rate	Male	20-24	954.18	510.11	1622.67
CD	1990	Prevalence	Rate	Male	25-29	1130.57	641.29	1859.51
CD	1990	Prevalence	Rate	Male	30-34	1188.05	673.22	1892.41
CD	1990	Prevalence	Rate	Male	35-39	1232.99	663.28	2043.02
CD	1990	Prevalence	Rate	Male	40-44	1374.11	741.75	2391.21
CD	1990	Prevalence	Rate	Male	45-49	1550.06	813.28	2672.97
CD	1990	Prevalence	Rate	Male	50-54	1647.81	929.65	2672.24
CD	1990	Prevalence	Rate	Male	55-59	1785.85	1040.49	2921.72
CD	1990	Prevalence	Rate	Male	60-64	1934.05	1087.47	3267.84
CD	1990	Prevalence	Rate	Male	65-69	2031.98	1071.31	3549.35
CD	1990	Prevalence	Rate	Male	70-74	2161.79	1191.54	3687.70
CD	1990	Prevalence	Rate	Male	75-79	2266.39	1149.87	3803.80
CD	1990	Prevalence	Rate	Male	80-84	2176.05	1222.65	3812.71
CD	1990	Prevalence	Rate	Male	85-89	1978.08	1076.79	3356.04
CD	1990	Prevalence	Rate	Male	90-94	1990.18	1081.77	3308.28
CD	1990	Prevalence	Rate	Male	95+	2245.03	1175.33	4043.60
CD	1990	Incidence	Number	Female	<5	0	0	0
CD	1990	Incidence	Number	Female	5-9	381426	183570	706336
CD	1990	Incidence	Number	Female	10-14	2218947	1156401	3727837
CD	1990	Incidence	Number	Female	15-19	5062294	2550048	7954199
CD	1990	Incidence	Number	Female	20-24	7383045	4038396	12021751
CD	1990	Incidence	Number	Female	25-29	8411426	4454711	13255517
CD	1990	Incidence	Number	Female	30-34	7860184	4284631	12608575
CD	1990	Incidence	Number	Female	35-39	7524686	4292842	13022770

CD	1990	Incidence	Number	Female	40-44	6674944	3648967	10599319
CD	1990	Incidence	Number	Female	45-49	6041407	3227056	9438840
CD	1990	Incidence	Number	Female	50-54	5955626	3593420	9282305
CD	1990	Incidence	Number	Female	55-59	5592147	3354065	8911961
CD	1990	Incidence	Number	Female	60-64	5332576	2919122	8384780
CD	1990	Incidence	Number	Female	65-69	4414356	2317316	7571521
CD	1990	Incidence	Number	Female	70-74	3275218	1696111	5350827
CD	1990	Incidence	Number	Female	75-79	2456312	1240440	4043892
CD	1990	Incidence	Number	Female	80-84	1326022	748004	2353854
CD	1990	Incidence	Number	Female	85-89	515692	291017	827492
CD	1990	Incidence	Number	Female	90-94	149787	90282	236541
CD	1990	Incidence	Number	Female	95+	40842	20872	69141
CD	1990	Incidence	Number	Male	<5	0	0	0
CD	1990	Incidence	Number	Male	5-9	371648	182356	695655
CD	1990	Incidence	Number	Male	10-14	2010147	1069020	3377472
CD	1990	Incidence	Number	Male	15-19	4357122	2225570	6842895
CD	1990	Incidence	Number	Male	20-24	6153536	3368167	10103551
CD	1990	Incidence	Number	Male	25-29	6886319	3696301	10904918
CD	1990	Incidence	Number	Male	30-34	6492406	3477828	10432649
CD	1990	Incidence	Number	Male	35-39	6249339	3505685	10787734
CD	1990	Incidence	Number	Male	40-44	5617818	3040588	8977022
CD	1990	Incidence	Number	Male	45-49	5112135	2725192	8093379
CD	1990	Incidence	Number	Male	50-54	4943851	2976911	7608497
CD	1990	Incidence	Number	Male	55-59	4633516	2776750	7466476
CD	1990	Incidence	Number	Male	60-64	4250284	2289830	6723834
CD	1990	Incidence	Number	Male	65-69	3288924	1720744	5630014

CD	1990	Incidence	Number	Male	70-74	2333282	1191614	3747545
CD	1990	Incidence	Number	Male	75-79	1590411	805812	2636361
CD	1990	Incidence	Number	Male	80-84	780975	436598	1371836
CD	1990	Incidence	Number	Male	85-89	268300	152416	431003
CD	1990	Incidence	Number	Male	90-94	66334	39697	106117
CD	1990	Incidence	Number	Male	95+	15076	7572	25575
CD	1990	Incidence	Rate	Female	<5	0	0.00	0.00
CD	1990	Incidence	Rate	Female	5-9	134.39	64.68	248.86
CD	1990	Incidence	Rate	Female	10-14	848.77	442.34	1425.94
CD	1990	Incidence	Rate	Female	15-19	1981.03	997.91	3112.72
CD	1990	Incidence	Rate	Female	20-24	3024.17	1654.17	4924.22
CD	1990	Incidence	Rate	Female	25-29	3821.65	2023.95	6022.51
CD	1990	Incidence	Rate	Female	30-34	4134.57	2253.78	6632.29
CD	1990	Incidence	Rate	Female	35-39	4338.16	2474.93	7507.94
CD	1990	Incidence	Rate	Female	40-44	4760.15	2602.21	7558.77
CD	1990	Incidence	Rate	Female	45-49	5308.76	2835.71	8294.19
CD	1990	Incidence	Rate	Female	50-54	5676.52	3425.02	8847.30
CD	1990	Incidence	Rate	Female	55-59	6057.88	3633.40	9654.18
CD	1990	Incidence	Rate	Female	60-64	6498.14	3557.17	10217.48
CD	1990	Incidence	Rate	Female	65-69	6660.34	3496.35	11423.84
CD	1990	Incidence	Rate	Female	70-74	6962.23	3605.48	11374.42
CD	1990	Incidence	Rate	Female	75-79	6762.47	3415.06	11133.24
CD	1990	Incidence	Rate	Female	80-84	6002.14	3385.79	10654.54
CD	1990	Incidence	Rate	Female	85-89	5132.79	2896.56	8236.20
CD	1990	Incidence	Rate	Female	90-94	4949.63	2983.33	7816.36
CD	1990	Incidence	Rate	Female	95+	5389.21	2754.03	9123.26

CD	1990	Incidence	Rate	Male	<5	0	0.00	0.00
CD	1990	Incidence	Rate	Male	5-9	124.01	60.85	232.12
CD	1990	Incidence	Rate	Male	10-14	732.96	389.79	1231.52
CD	1990	Incidence	Rate	Male	15-19	1651.15	843.39	2593.14
CD	1990	Incidence	Rate	Male	20-24	2481.74	1358.39	4074.79
CD	1990	Incidence	Rate	Male	25-29	3094.7	1661.11	4900.64
CD	1990	Incidence	Rate	Male	30-34	3324.1	1780.64	5341.49
CD	1990	Incidence	Rate	Male	35-39	3495.33	1960.77	6033.71
CD	1990	Incidence	Rate	Male	40-44	3841.08	2078.95	6137.88
CD	1990	Incidence	Rate	Male	45-49	4317.87	2301.78	6835.92
CD	1990	Incidence	Rate	Male	50-54	4592.32	2765.24	7067.50
CD	1990	Incidence	Rate	Male	55-59	4988.28	2989.35	8038.15
CD	1990	Incidence	Rate	Male	60-64	5411.2	2915.27	8560.37
CD	1990	Incidence	Rate	Male	65-69	5736.71	3001.41	9820.15
CD	1990	Incidence	Rate	Male	70-74	6202.48	3167.63	9961.97
CD	1990	Incidence	Rate	Male	75-79	6302.94	3193.51	10448.14
CD	1990	Incidence	Rate	Male	80-84	5879.31	3286.79	10327.42
CD	1990	Incidence	Rate	Male	85-89	5298.13	3009.76	8511.01
CD	1990	Incidence	Rate	Male	90-94	5268.97	3153.19	8428.91
CD	1990	Incidence	Rate	Male	95+	5793.33	2909.57	9827.87
CD	2021	DALYs	Number	Female	<5	0	0	0
CD	2021	DALYs	Number	Female	5-9	6331	2495	13175
CD	2021	DALYs	Number	Female	10-14	32872	15089	63966
CD	2021	DALYs	Number	Female	15-19	61995	29722	116811
CD	2021	DALYs	Number	Female	20-24	81244	39136	153919
CD	2021	DALYs	Number	Female	25-29	96410	46898	181970

CD	2021	DALYs	Number	Female	30-34	105454	50976	182027
CD	2021	DALYs	Number	Female	35-39	100672	48443	184324
CD	2021	DALYs	Number	Female	40-44	102291	48021	200932
CD	2021	DALYs	Number	Female	45-49	110725	51489	213570
CD	2021	DALYs	Number	Female	50-54	112005	54422	200187
CD	2021	DALYs	Number	Female	55-59	107190	53988	192540
CD	2021	DALYs	Number	Female	60-64	91684	43135	168336
CD	2021	DALYs	Number	Female	65-69	82563	37294	160143
CD	2021	DALYs	Number	Female	70-74	61958	30974	116508
CD	2021	DALYs	Number	Female	75-79	41392	18207	73009
CD	2021	DALYs	Number	Female	80-84	25804	12136	49440
CD	2021	DALYs	Number	Female	85-89	11745	5826	21573
CD	2021	DALYs	Number	Female	90-94	4483	2162	8026
CD	2021	DALYs	Number	Female	95+	1461	613	2872
CD	2021	DALYs	Number	Male	<5	0	0	0
CD	2021	DALYs	Number	Male	5-9	6265	2481	12821
CD	2021	DALYs	Number	Male	10-14	31001	14717	59255
CD	2021	DALYs	Number	Male	15-19	56527	27286	106983
CD	2021	DALYs	Number	Male	20-24	71642	34172	136713
CD	2021	DALYs	Number	Male	25-29	83749	39203	159446
CD	2021	DALYs	Number	Male	30-34	91187	42997	156355
CD	2021	DALYs	Number	Male	35-39	86386	40548	159178
CD	2021	DALYs	Number	Male	40-44	87117	40776	170783
CD	2021	DALYs	Number	Male	45-49	94073	44588	181677
CD	2021	DALYs	Number	Male	50-54	94298	45812	169573
CD	2021	DALYs	Number	Male	55-59	88039	44691	159585

CD	2021	DALYs	Number	Male	60-64	73562	34336	133371
CD	2021	DALYs	Number	Male	65-69	64964	30046	126845
CD	2021	DALYs	Number	Male	70-74	47712	22880	90580
CD	2021	DALYs	Number	Male	75-79	30768	13537	55775
CD	2021	DALYs	Number	Male	80-84	17329	8329	33936
CD	2021	DALYs	Number	Male	85-89	6986	3534	12857
CD	2021	DALYs	Number	Male	90-94	2279	1120	4057
CD	2021	DALYs	Number	Male	95+	604	257	1200
CD	2021	DALYs	Rate	Female	<5	0	0.00	0.00
CD	2021	DALYs	Rate	Female	5-9	1.9	0.75	3.96
CD	2021	DALYs	Rate	Female	10-14	10.18	4.67	19.81
CD	2021	DALYs	Rate	Female	15-19	20.42	9.79	38.47
CD	2021	DALYs	Rate	Female	20-24	27.66	13.32	52.40
CD	2021	DALYs	Rate	Female	25-29	33.13	16.12	62.54
CD	2021	DALYs	Rate	Female	30-34	35.28	17.05	60.89
CD	2021	DALYs	Rate	Female	35-39	36.24	17.44	66.35
CD	2021	DALYs	Rate	Female	40-44	41.23	19.36	80.99
CD	2021	DALYs	Rate	Female	45-49	46.99	21.85	90.63
CD	2021	DALYs	Rate	Female	50-54	50.24	24.41	89.79
CD	2021	DALYs	Rate	Female	55-59	53.33	26.86	95.79
CD	2021	DALYs	Rate	Female	60-64	55.73	26.22	102.33
CD	2021	DALYs	Rate	Female	65-69	57.33	25.90	111.20
CD	2021	DALYs	Rate	Female	70-74	56.61	28.30	106.45
CD	2021	DALYs	Rate	Female	75-79	57.41	25.25	101.26
CD	2021	DALYs	Rate	Female	80-84	50.66	23.83	97.07
CD	2021	DALYs	Rate	Female	85-89	41.25	20.47	75.78

CD	2021	DALYs	Rate	Female	90-94	37.17	17.93	66.55
CD	2021	DALYs	Rate	Female	95+	37.11	15.57	72.93
CD	2021	DALYs	Rate	Male	<5	0	0.00	0.00
CD	2021	DALYs	Rate	Male	5-9	1.77	0.70	3.62
CD	2021	DALYs	Rate	Male	10-14	9.02	4.28	17.24
CD	2021	DALYs	Rate	Male	15-19	17.65	8.52	33.40
CD	2021	DALYs	Rate	Male	20-24	23.61	11.26	45.06
CD	2021	DALYs	Rate	Male	25-29	28.16	13.18	53.62
CD	2021	DALYs	Rate	Male	30-34	29.84	14.07	51.17
CD	2021	DALYs	Rate	Male	35-39	30.52	14.32	56.23
CD	2021	DALYs	Rate	Male	40-44	34.55	16.17	67.73
CD	2021	DALYs	Rate	Male	45-49	39.55	18.75	76.38
CD	2021	DALYs	Rate	Male	50-54	42.48	20.64	76.39
CD	2021	DALYs	Rate	Male	55-59	45.21	22.95	81.95
CD	2021	DALYs	Rate	Male	60-64	47.3	22.08	85.75
CD	2021	DALYs	Rate	Male	65-69	49.28	22.79	96.22
CD	2021	DALYs	Rate	Male	70-74	49.5	23.74	93.97
CD	2021	DALYs	Rate	Male	75-79	51.46	22.64	93.29
CD	2021	DALYs	Rate	Male	80-84	47.28	22.73	92.59
CD	2021	DALYs	Rate	Male	85-89	40.49	20.48	74.52
CD	2021	DALYs	Rate	Male	90-94	39.1	19.21	69.61
CD	2021	DALYs	Rate	Male	95+	39.93	16.99	79.38
CD	2021	Prevalence	Number	Female	<5	0	0	0
CD	2021	Prevalence	Number	Female	5-9	243801	113150	448029
CD	2021	Prevalence	Number	Female	10-14	1283315	671512	2252191
CD	2021	Prevalence	Number	Female	15-19	2436700	1323561	4265674

CD	2021	Prevalence	Number	Female	20-24	3209037	1734207	5369539
CD	2021	Prevalence	Number	Female	25-29	3821671	2184623	6250912
CD	2021	Prevalence	Number	Female	30-34	4211757	2386859	6630136
CD	2021	Prevalence	Number	Female	35-39	4049059	2212588	6725953
CD	2021	Prevalence	Number	Female	40-44	4120981	2282199	7054749
CD	2021	Prevalence	Number	Female	45-49	4494276	2364950	7631763
CD	2021	Prevalence	Number	Female	50-54	4579045	2583632	7438493
CD	2021	Prevalence	Number	Female	55-59	4417366	2539944	7343402
CD	2021	Prevalence	Number	Female	60-64	3826163	2151931	6555906
CD	2021	Prevalence	Number	Female	65-69	3491837	1853678	6022920
CD	2021	Prevalence	Number	Female	70-74	2666692	1505581	4564984
CD	2021	Prevalence	Number	Female	75-79	1807457	917071	3024445
CD	2021	Prevalence	Number	Female	80-84	1147078	637804	2056291
CD	2021	Prevalence	Number	Female	85-89	535025	289471	917337
CD	2021	Prevalence	Number	Female	90-94	208469	114607	347621
CD	2021	Prevalence	Number	Female	95+	69589	36635	125332
CD	2021	Prevalence	Number	Male	<5	0	0	0
CD	2021	Prevalence	Number	Male	5-9	241094	114039	444759
CD	2021	Prevalence	Number	Male	10-14	1204038	637547	2082661
CD	2021	Prevalence	Number	Male	15-19	2198571	1226216	3841438
CD	2021	Prevalence	Number	Male	20-24	2792078	1500000	4698692
CD	2021	Prevalence	Number	Male	25-29	3270469	1858066	5331766
CD	2021	Prevalence	Number	Male	30-34	3584463	2026267	5690593
CD	2021	Prevalence	Number	Male	35-39	3415677	1832198	5678911
CD	2021	Prevalence	Number	Male	40-44	3455587	1861910	6007923
CD	2021	Prevalence	Number	Male	45-49	3756605	1978984	6479017

CD	2021	Prevalence	Number	Male	50-54	3802016	2152232	6194136
CD	2021	Prevalence	Number	Male	55-59	3583156	2082456	5881084
CD	2021	Prevalence	Number	Male	60-64	3035658	1690919	5142368
CD	2021	Prevalence	Number	Male	65-69	2720451	1432202	4765935
CD	2021	Prevalence	Number	Male	70-74	2035539	1113784	3456119
CD	2021	Prevalence	Number	Male	75-79	1332435	673602	2243927
CD	2021	Prevalence	Number	Male	80-84	763207	423563	1337462
CD	2021	Prevalence	Number	Male	85-89	314459	171779	538533
CD	2021	Prevalence	Number	Male	90-94	104503	56899	172524
CD	2021	Prevalence	Number	Male	95+	28327	14878	50534
CD	2021	Prevalence	Rate	Female	<5	0	0.00	0.00
CD	2021	Prevalence	Rate	Female	5-9	73.31	34.02	134.71
CD	2021	Prevalence	Rate	Female	10-14	397.4	207.95	697.43
CD	2021	Prevalence	Rate	Female	15-19	802.47	435.88	1404.80
CD	2021	Prevalence	Rate	Female	20-24	1092.43	590.36	1827.92
CD	2021	Prevalence	Rate	Female	25-29	1313.35	750.76	2148.17
CD	2021	Prevalence	Rate	Female	30-34	1408.94	798.47	2217.95
CD	2021	Prevalence	Rate	Female	35-39	1457.53	796.46	2421.13
CD	2021	Prevalence	Rate	Female	40-44	1661.08	919.91	2843.63
CD	2021	Prevalence	Rate	Female	45-49	1907.24	1003.62	3238.70
CD	2021	Prevalence	Rate	Female	50-54	2053.91	1158.88	3336.51
CD	2021	Prevalence	Rate	Female	55-59	2197.66	1263.63	3653.37
CD	2021	Prevalence	Rate	Female	60-64	2325.78	1308.08	3985.09
CD	2021	Prevalence	Rate	Female	65-69	2424.74	1287.20	4182.32
CD	2021	Prevalence	Rate	Female	70-74	2436.5	1375.62	4170.92
CD	2021	Prevalence	Rate	Female	75-79	2506.96	1271.98	4194.93

CD	2021	Prevalence	Rate	Female	80-84	2252.21	1252.28	4037.39
CD	2021	Prevalence	Rate	Female	85-89	1879.32	1016.79	3222.21
CD	2021	Prevalence	Rate	Female	90-94	1728.47	950.24	2882.22
CD	2021	Prevalence	Rate	Female	95+	1766.99	930.22	3182.41
CD	2021	Prevalence	Rate	Male	<5	0	0.00	0.00
CD	2021	Prevalence	Rate	Male	5-9	68.01	32.17	125.47
CD	2021	Prevalence	Rate	Male	10-14	350.31	185.49	605.94
CD	2021	Prevalence	Rate	Male	15-19	686.35	382.80	1199.21
CD	2021	Prevalence	Rate	Male	20-24	920.24	494.39	1548.65
CD	2021	Prevalence	Rate	Male	25-29	1099.85	624.86	1793.06
CD	2021	Prevalence	Rate	Male	30-34	1173.12	663.16	1862.41
CD	2021	Prevalence	Rate	Male	35-39	1206.68	647.27	2006.23
CD	2021	Prevalence	Rate	Male	40-44	1370.39	738.38	2382.57
CD	2021	Prevalence	Rate	Male	45-49	1579.32	831.99	2723.86
CD	2021	Prevalence	Rate	Male	50-54	1712.77	969.56	2790.40
CD	2021	Prevalence	Rate	Male	55-59	1840.12	1069.44	3020.21
CD	2021	Prevalence	Rate	Male	60-64	1951.73	1087.15	3306.20
CD	2021	Prevalence	Rate	Male	65-69	2063.55	1086.37	3615.12
CD	2021	Prevalence	Rate	Male	70-74	2111.74	1155.48	3585.50
CD	2021	Prevalence	Rate	Male	75-79	2228.65	1126.67	3753.22
CD	2021	Prevalence	Rate	Male	80-84	2082.32	1155.64	3649.10
CD	2021	Prevalence	Rate	Male	85-89	1822.67	995.67	3121.45
CD	2021	Prevalence	Rate	Male	90-94	1792.96	976.21	2960.01
CD	2021	Prevalence	Rate	Male	95+	1873.41	983.95	3342.10
CD	2021	Incidence	Number	Female	<5	0	0	0
CD	2021	Incidence	Number	Female	5-9	408638	197775	762545

CD	2021	Incidence	Number	Female	10-14	2555548	1331842	4317781
CD	2021	Incidence	Number	Female	15-19	5678142	2848133	8926104
CD	2021	Incidence	Number	Female	20-24	8362380	4584217	13577542
CD	2021	Incidence	Number	Female	25-29	10490093	5535179	16494761
CD	2021	Incidence	Number	Female	30-34	11866276	6502398	19071103
CD	2021	Incidence	Number	Female	35-39	11560013	6592894	20066643
CD	2021	Incidence	Number	Female	40-44	11578145	6340014	18461270
CD	2021	Incidence	Number	Female	45-49	12555378	6710667	19704068
CD	2021	Incidence	Number	Female	50-54	12777405	7715566	19982418
CD	2021	Incidence	Number	Female	55-59	12382660	7429132	19754858
CD	2021	Incidence	Number	Female	60-64	10769320	5886436	16913071
CD	2021	Incidence	Number	Female	65-69	9932985	5198652	16935047
CD	2021	Incidence	Number	Female	70-74	7672492	3968711	12526634
CD	2021	Incidence	Number	Female	75-79	5045690	2539098	8356296
CD	2021	Incidence	Number	Female	80-84	3144917	1771872	5628011
CD	2021	Incidence	Number	Female	85-89	1474538	827599	2374853
CD	2021	Incidence	Number	Female	90-94	574414	349706	906542
CD	2021	Incidence	Number	Female	95+	186813	94807	320636
CD	2021	Incidence	Number	Male	<5	0	0	0
CD	2021	Incidence	Number	Male	5-9	400960	196459	757197
CD	2021	Incidence	Number	Male	10-14	2376640	1257721	3999877
CD	2021	Incidence	Number	Male	15-19	5094529	2583226	7983904
CD	2021	Incidence	Number	Male	20-24	7269620	3965853	11892246
CD	2021	Incidence	Number	Male	25-29	8990005	4836997	14232378
CD	2021	Incidence	Number	Male	30-34	10118264	5430789	16241313
CD	2021	Incidence	Number	Male	35-39	9769471	5477456	16878089

CD	2021	Incidence	Number	Male	40-44	9744340	5258345	15598986
CD	2021	Incidence	Number	Male	45-49	10544791	5619514	16666299
CD	2021	Incidence	Number	Male	50-54	10657619	6392563	16422357
CD	2021	Incidence	Number	Male	55-59	10072481	6019241	16220332
CD	2021	Incidence	Number	Male	60-64	8562340	4627217	13549102
CD	2021	Incidence	Number	Male	65-69	7757654	4065964	13239436
CD	2021	Incidence	Number	Male	70-74	5884406	3014636	9439677
CD	2021	Incidence	Number	Male	75-79	3736581	1887198	6201887
CD	2021	Incidence	Number	Male	80-84	2088488	1159410	3657854
CD	2021	Incidence	Number	Male	85-89	861841	490495	1379821
CD	2021	Incidence	Number	Male	90-94	283919	170277	458902
CD	2021	Incidence	Number	Male	95+	74940	37769	128847
CD	2021	Incidence	Rate	Female	<5	0	0.00	0.00
CD	2021	Incidence	Rate	Female	5-9	122.87	59.47	229.28
CD	2021	Incidence	Rate	Female	10-14	791.37	412.43	1337.08
CD	2021	Incidence	Rate	Female	15-19	1869.96	937.97	2939.60
CD	2021	Incidence	Rate	Female	20-24	2846.75	1560.57	4622.11
CD	2021	Incidence	Rate	Female	25-29	3605	1902.21	5668.55
CD	2021	Incidence	Rate	Female	30-34	3969.57	2175.22	6379.77
CD	2021	Incidence	Rate	Female	35-39	4161.23	2373.23	7223.34
CD	2021	Incidence	Rate	Female	40-44	4666.92	2555.53	7441.36
CD	2021	Incidence	Rate	Female	45-49	5328.15	2847.82	8361.85
CD	2021	Incidence	Rate	Female	50-54	5731.25	3460.79	8963.03
CD	2021	Incidence	Rate	Female	55-59	6160.42	3696.02	9828.12
CD	2021	Incidence	Rate	Female	60-64	6546.26	3578.14	10280.82
CD	2021	Incidence	Rate	Female	65-69	6897.48	3609.95	11759.72

CD	2021	Incidence	Rate	Female	70-74	7010.18	3626.12	11445.30
CD	2021	Incidence	Rate	Female	75-79	6998.41	3521.75	11590.24
CD	2021	Incidence	Rate	Female	80-84	6174.84	3478.95	11050.22
CD	2021	Incidence	Rate	Female	85-89	5179.43	2907.00	8341.85
CD	2021	Incidence	Rate	Female	90-94	4762.62	2899.51	7516.39
CD	2021	Incidence	Rate	Female	95+	4743.54	2407.33	8141.54
CD	2021	Incidence	Rate	Male	<5	0	0.00	0.00
CD	2021	Incidence	Rate	Male	5-9	113.11	55.42	213.61
CD	2021	Incidence	Rate	Male	10-14	691.47	365.93	1163.74
CD	2021	Incidence	Rate	Male	15-19	1590.4	806.43	2492.40
CD	2021	Incidence	Rate	Male	20-24	2396	1307.11	3919.58
CD	2021	Incidence	Rate	Male	25-29	3023.31	1626.67	4786.31
CD	2021	Incidence	Rate	Male	30-34	3311.5	1777.39	5315.45
CD	2021	Incidence	Rate	Male	35-39	3451.34	1935.06	5962.65
CD	2021	Incidence	Rate	Male	40-44	3864.33	2085.31	6186.12
CD	2021	Incidence	Rate	Male	45-49	4433.15	2362.51	7006.71
CD	2021	Incidence	Rate	Male	50-54	4801.15	2879.79	7398.11
CD	2021	Incidence	Rate	Male	55-59	5172.68	3091.16	8329.89
CD	2021	Incidence	Rate	Male	60-64	5505.02	2975.00	8711.18
CD	2021	Incidence	Rate	Male	65-69	5884.44	3084.17	10042.56
CD	2021	Incidence	Rate	Male	70-74	6104.7	3127.49	9793.07
CD	2021	Incidence	Rate	Male	75-79	6249.85	3156.55	10373.35
CD	2021	Incidence	Rate	Male	80-84	5698.18	3163.30	9980.00
CD	2021	Incidence	Rate	Male	85-89	4995.41	2843.01	7997.73
CD	2021	Incidence	Rate	Male	90-94	4871.22	2921.46	7873.42
CD	2021	Incidence	Rate	Male	95+	4956.24	2497.91	8521.36

Urticaria	1990	DALYs	Number	Female	<5	356294	228566	524660
Urticaria	1990	DALYs	Number	Female	5-9	264180	151002	420069
Urticaria	1990	DALYs	Number	Female	10-14	172644	100221	270565
Urticaria	1990	DALYs	Number	Female	15-19	149804	88037	243175
Urticaria	1990	DALYs	Number	Female	20-24	136995	79469	220606
Urticaria	1990	DALYs	Number	Female	25-29	117427	67972	188267
Urticaria	1990	DALYs	Number	Female	30-34	96874	58511	149855
Urticaria	1990	DALYs	Number	Female	35-39	84288	50766	127993
Urticaria	1990	DALYs	Number	Female	40-44	66106	37315	107228
Urticaria	1990	DALYs	Number	Female	45-49	51683	30780	79951
Urticaria	1990	DALYs	Number	Female	50-54	48177	28966	73193
Urticaria	1990	DALYs	Number	Female	55-59	42141	25691	63060
Urticaria	1990	DALYs	Number	Female	60-64	37786	22589	56645
Urticaria	1990	DALYs	Number	Female	65-69	30693	18720	47010
Urticaria	1990	DALYs	Number	Female	70-74	20514	12681	31266
Urticaria	1990	DALYs	Number	Female	75-79	14327	8761	21677
Urticaria	1990	DALYs	Number	Female	80-84	7352	4466	10822
Urticaria	1990	DALYs	Number	Female	85-89	2676	1680	3993
Urticaria	1990	DALYs	Number	Female	90-94	641	393	969
Urticaria	1990	DALYs	Number	Female	95+	134	82	202
Urticaria	1990	DALYs	Number	Male	<5	269068	172743	394399
Urticaria	1990	DALYs	Number	Male	5-9	198606	113965	310002
Urticaria	1990	DALYs	Number	Male	10-14	128403	74286	201828
Urticaria	1990	DALYs	Number	Male	15-19	105820	61659	171639
Urticaria	1990	DALYs	Number	Male	20-24	92745	52708	151840
Urticaria	1990	DALYs	Number	Male	25-29	77218	44503	122569

Urticaria	1990	DALYs	Number	Male	30-34	63406	38253	97923
Urticaria	1990	DALYs	Number	Male	35-39	55074	32641	85413
Urticaria	1990	DALYs	Number	Male	40-44	42941	23782	71245
Urticaria	1990	DALYs	Number	Male	45-49	33692	19508	52218
Urticaria	1990	DALYs	Number	Male	50-54	30606	17947	47400
Urticaria	1990	DALYs	Number	Male	55-59	26363	16142	39689
Urticaria	1990	DALYs	Number	Male	60-64	22238	13377	33436
Urticaria	1990	DALYs	Number	Male	65-69	16224	9745	24576
Urticaria	1990	DALYs	Number	Male	70-74	10182	6428	15466
Urticaria	1990	DALYs	Number	Male	75-79	6358	3861	9836
Urticaria	1990	DALYs	Number	Male	80-84	3017	1839	4495
Urticaria	1990	DALYs	Number	Male	85-89	1008	628	1508
Urticaria	1990	DALYs	Number	Male	90-94	220	136	328
Urticaria	1990	DALYs	Number	Male	95+	42	25	65
Urticaria	1990	DALYs	Rate	Female	<5	118.61	76.09	174.65
Urticaria	1990	DALYs	Rate	Female	5-9	93.08	53.20	148.00
Urticaria	1990	DALYs	Rate	Female	10-14	66.04	38.34	103.49
Urticaria	1990	DALYs	Rate	Female	15-19	58.62	34.45	95.16
Urticaria	1990	DALYs	Rate	Female	20-24	56.11	32.55	90.36
Urticaria	1990	DALYs	Rate	Female	25-29	53.35	30.88	85.54
Urticaria	1990	DALYs	Rate	Female	30-34	50.96	30.78	78.83
Urticaria	1990	DALYs	Rate	Female	35-39	48.59	29.27	73.79
Urticaria	1990	DALYs	Rate	Female	40-44	47.14	26.61	76.47
Urticaria	1990	DALYs	Rate	Female	45-49	45.42	27.05	70.26
Urticaria	1990	DALYs	Rate	Female	50-54	45.92	27.61	69.76
Urticaria	1990	DALYs	Rate	Female	55-59	45.65	27.83	68.31

Urticaria	1990	DALYs	Rate	Female	60-64	46.04	27.53	69.03
Urticaria	1990	DALYs	Rate	Female	65-69	46.31	28.25	70.93
Urticaria	1990	DALYs	Rate	Female	70-74	43.61	26.96	66.46
Urticaria	1990	DALYs	Rate	Female	75-79	39.44	24.12	59.68
Urticaria	1990	DALYs	Rate	Female	80-84	33.28	20.22	48.99
Urticaria	1990	DALYs	Rate	Female	85-89	26.63	16.73	39.74
Urticaria	1990	DALYs	Rate	Female	90-94	21.17	12.99	32.02
Urticaria	1990	DALYs	Rate	Female	95+	17.64	10.78	26.69
Urticaria	1990	DALYs	Rate	Male	<5	84.21	54.06	123.43
Urticaria	1990	DALYs	Rate	Male	5-9	66.27	38.03	103.44
Urticaria	1990	DALYs	Rate	Male	10-14	46.82	27.09	73.59
Urticaria	1990	DALYs	Rate	Male	15-19	40.1	23.37	65.04
Urticaria	1990	DALYs	Rate	Male	20-24	37.4	21.26	61.24
Urticaria	1990	DALYs	Rate	Male	25-29	34.7	20.00	55.08
Urticaria	1990	DALYs	Rate	Male	30-34	32.46	19.59	50.14
Urticaria	1990	DALYs	Rate	Male	35-39	30.8	18.26	47.77
Urticaria	1990	DALYs	Rate	Male	40-44	29.36	16.26	48.71
Urticaria	1990	DALYs	Rate	Male	45-49	28.46	16.48	44.11
Urticaria	1990	DALYs	Rate	Male	50-54	28.43	16.67	44.03
Urticaria	1990	DALYs	Rate	Male	55-59	28.38	17.38	42.73
Urticaria	1990	DALYs	Rate	Male	60-64	28.31	17.03	42.57
Urticaria	1990	DALYs	Rate	Male	65-69	28.3	17.00	42.87
Urticaria	1990	DALYs	Rate	Male	70-74	27.07	17.09	41.11
Urticaria	1990	DALYs	Rate	Male	75-79	25.2	15.30	38.98
Urticaria	1990	DALYs	Rate	Male	80-84	22.72	13.84	33.84
Urticaria	1990	DALYs	Rate	Male	85-89	19.91	12.40	29.77

Urticaria	1990	DALYs	Rate	Male	90-94	17.47	10.82	26.09
Urticaria	1990	DALYs	Rate	Male	95+	16.13	9.52	25.08
Urticaria	1990	Prevalence	Number	Female	<5	5826926	4745025	7219411
Urticaria	1990	Prevalence	Number	Female	5-9	4314001	3026630	6219141
Urticaria	1990	Prevalence	Number	Female	10-14	2835080	1944091	4022120
Urticaria	1990	Prevalence	Number	Female	15-19	2468891	1760468	3465840
Urticaria	1990	Prevalence	Number	Female	20-24	2271272	1610436	3095355
Urticaria	1990	Prevalence	Number	Female	25-29	1961099	1396991	2703307
Urticaria	1990	Prevalence	Number	Female	30-34	1633545	1141206	2238643
Urticaria	1990	Prevalence	Number	Female	35-39	1432478	1033852	1988817
Urticaria	1990	Prevalence	Number	Female	40-44	1126593	778941	1610374
Urticaria	1990	Prevalence	Number	Female	45-49	886463	629709	1209851
Urticaria	1990	Prevalence	Number	Female	50-54	833573	566866	1117728
Urticaria	1990	Prevalence	Number	Female	55-59	733978	523656	984836
Urticaria	1990	Prevalence	Number	Female	60-64	664631	481300	908742
Urticaria	1990	Prevalence	Number	Female	65-69	548118	378702	767877
Urticaria	1990	Prevalence	Number	Female	70-74	372151	269532	511471
Urticaria	1990	Prevalence	Number	Female	75-79	264716	190765	366468
Urticaria	1990	Prevalence	Number	Female	80-84	138425	93823	186217
Urticaria	1990	Prevalence	Number	Female	85-89	51421	36751	67037
Urticaria	1990	Prevalence	Number	Female	90-94	12562	9093	16810
Urticaria	1990	Prevalence	Number	Female	95+	2687	1913	3740
Urticaria	1990	Prevalence	Number	Male	<5	4412038	3600524	5485787
Urticaria	1990	Prevalence	Number	Male	5-9	3244887	2309807	4679227
Urticaria	1990	Prevalence	Number	Male	10-14	2101247	1442895	2926221
Urticaria	1990	Prevalence	Number	Male	15-19	1730090	1224439	2431590

Urticaria	1990	Prevalence	Number	Male	20-24	1518549	1072630	2105598
Urticaria	1990	Prevalence	Number	Male	25-29	1270680	896593	1751205
Urticaria	1990	Prevalence	Number	Male	30-34	1053498	721557	1459298
Urticaria	1990	Prevalence	Number	Male	35-39	919274	649888	1299710
Urticaria	1990	Prevalence	Number	Male	40-44	719449	490211	1032092
Urticaria	1990	Prevalence	Number	Male	45-49	568221	398577	778701
Urticaria	1990	Prevalence	Number	Male	50-54	521540	352026	706225
Urticaria	1990	Prevalence	Number	Male	55-59	454715	322833	620687
Urticaria	1990	Prevalence	Number	Male	60-64	387149	277820	537081
Urticaria	1990	Prevalence	Number	Male	65-69	287795	197316	404743
Urticaria	1990	Prevalence	Number	Male	70-74	183873	132362	251223
Urticaria	1990	Prevalence	Number	Male	75-79	116966	83896	160872
Urticaria	1990	Prevalence	Number	Male	80-84	56638	37502	76601
Urticaria	1990	Prevalence	Number	Male	85-89	19278	13757	25646
Urticaria	1990	Prevalence	Number	Male	90-94	4271	3005	5796
Urticaria	1990	Prevalence	Number	Male	95+	835	582	1182
Urticaria	1990	Prevalence	Rate	Female	<5	1939.72	1579.57	2403.26
Urticaria	1990	Prevalence	Rate	Female	5-9	1519.93	1066.36	2191.16
Urticaria	1990	Prevalence	Rate	Female	10-14	1084.45	743.64	1538.51
Urticaria	1990	Prevalence	Rate	Female	15-19	966.15	688.92	1356.29
Urticaria	1990	Prevalence	Rate	Female	20-24	930.33	659.65	1267.89
Urticaria	1990	Prevalence	Rate	Female	25-29	891.01	634.71	1228.22
Urticaria	1990	Prevalence	Rate	Female	30-34	859.27	600.29	1177.56
Urticaria	1990	Prevalence	Rate	Female	35-39	825.86	596.04	1146.60
Urticaria	1990	Prevalence	Rate	Female	40-44	803.42	555.49	1148.42
Urticaria	1990	Prevalence	Rate	Female	45-49	778.96	553.34	1063.13

Urticaria	1990	Prevalence	Rate	Female	50-54	794.51	540.30	1065.35
Urticaria	1990	Prevalence	Rate	Female	55-59	795.11	567.27	1066.86
Urticaria	1990	Prevalence	Rate	Female	60-64	809.9	586.50	1107.37
Urticaria	1990	Prevalence	Rate	Female	65-69	827	571.38	1158.57
Urticaria	1990	Prevalence	Rate	Female	70-74	791.09	572.95	1087.25
Urticaria	1990	Prevalence	Rate	Female	75-79	728.79	525.19	1008.92
Urticaria	1990	Prevalence	Rate	Female	80-84	626.57	424.68	842.90
Urticaria	1990	Prevalence	Rate	Female	85-89	511.8	365.79	667.24
Urticaria	1990	Prevalence	Rate	Female	90-94	415.1	300.46	555.47
Urticaria	1990	Prevalence	Rate	Female	95+	354.51	252.45	493.46
Urticaria	1990	Prevalence	Rate	Male	<5	1380.76	1126.79	1716.79
Urticaria	1990	Prevalence	Rate	Male	5-9	1082.7	770.70	1561.29
Urticaria	1990	Prevalence	Rate	Male	10-14	766.17	526.12	1066.98
Urticaria	1990	Prevalence	Rate	Male	15-19	655.62	464.01	921.46
Urticaria	1990	Prevalence	Rate	Male	20-24	612.43	432.59	849.19
Urticaria	1990	Prevalence	Rate	Male	25-29	571.04	402.93	786.99
Urticaria	1990	Prevalence	Rate	Male	30-34	539.39	369.44	747.16
Urticaria	1990	Prevalence	Rate	Male	35-39	514.16	363.49	726.94
Urticaria	1990	Prevalence	Rate	Male	40-44	491.91	335.17	705.67
Urticaria	1990	Prevalence	Rate	Male	45-49	479.94	336.65	657.72
Urticaria	1990	Prevalence	Rate	Male	50-54	484.46	327.00	656.01
Urticaria	1990	Prevalence	Rate	Male	55-59	489.53	347.55	668.21
Urticaria	1990	Prevalence	Rate	Male	60-64	492.89	353.70	683.78
Urticaria	1990	Prevalence	Rate	Male	65-69	501.99	344.17	705.97
Urticaria	1990	Prevalence	Rate	Male	70-74	488.78	351.85	667.82
Urticaria	1990	Prevalence	Rate	Male	75-79	463.54	332.49	637.55

Urticaria	1990	Prevalence	Rate	Male	80-84	426.38	282.32	576.67
Urticaria	1990	Prevalence	Rate	Male	85-89	380.68	271.65	506.42
Urticaria	1990	Prevalence	Rate	Male	90-94	339.24	238.69	460.40
Urticaria	1990	Prevalence	Rate	Male	95+	320.91	223.52	454.15
Urticaria	1990	Incidence	Number	Female	<5	11355552	9147280	14032667
Urticaria	1990	Incidence	Number	Female	5-9	7166259	4961110	10345159
Urticaria	1990	Incidence	Number	Female	10-14	4829933	3329473	6911870
Urticaria	1990	Incidence	Number	Female	15-19	4291775	2978215	5877437
Urticaria	1990	Incidence	Number	Female	20-24	3963414	2878623	5445054
Urticaria	1990	Incidence	Number	Female	25-29	3412324	2421296	4558610
Urticaria	1990	Incidence	Number	Female	30-34	2846869	1953510	3873519
Urticaria	1990	Incidence	Number	Female	35-39	2498877	1753571	3495362
Urticaria	1990	Incidence	Number	Female	40-44	1960555	1400716	2723145
Urticaria	1990	Incidence	Number	Female	45-49	1551744	1087162	2131253
Urticaria	1990	Incidence	Number	Female	50-54	1465568	1030877	1946701
Urticaria	1990	Incidence	Number	Female	55-59	1286176	915826	1724697
Urticaria	1990	Incidence	Number	Female	60-64	1176139	846665	1594395
Urticaria	1990	Incidence	Number	Female	65-69	958743	679946	1300422
Urticaria	1990	Incidence	Number	Female	70-74	645803	472268	885205
Urticaria	1990	Incidence	Number	Female	75-79	455526	327811	625557
Urticaria	1990	Incidence	Number	Female	80-84	236556	159969	317914
Urticaria	1990	Incidence	Number	Female	85-89	87275	63173	113473
Urticaria	1990	Incidence	Number	Female	90-94	21572	15732	28871
Urticaria	1990	Incidence	Number	Female	95+	4617	3122	6663
Urticaria	1990	Incidence	Number	Male	<5	8596243	6997491	10593578
Urticaria	1990	Incidence	Number	Male	5-9	5389807	3740520	7802214

Urticaria	1990	Incidence	Number	Male	10-14	3564110	2429546	4974336
Urticaria	1990	Incidence	Number	Male	15-19	2994799	2076298	4134147
Urticaria	1990	Incidence	Number	Male	20-24	2643566	1905165	3688575
Urticaria	1990	Incidence	Number	Male	25-29	2208437	1530379	2949044
Urticaria	1990	Incidence	Number	Male	30-34	1834900	1237923	2530177
Urticaria	1990	Incidence	Number	Male	35-39	1605578	1122610	2277033
Urticaria	1990	Incidence	Number	Male	40-44	1252418	879682	1754190
Urticaria	1990	Incidence	Number	Male	45-49	996025	690291	1365731
Urticaria	1990	Incidence	Number	Male	50-54	918611	635046	1233694
Urticaria	1990	Incidence	Number	Male	55-59	797930	561905	1069623
Urticaria	1990	Incidence	Number	Male	60-64	685736	485780	940586
Urticaria	1990	Incidence	Number	Male	65-69	503731	359931	688685
Urticaria	1990	Incidence	Number	Male	70-74	320530	232482	440291
Urticaria	1990	Incidence	Number	Male	75-79	202830	146879	278547
Urticaria	1990	Incidence	Number	Male	80-84	97802	65439	132606
Urticaria	1990	Incidence	Number	Male	85-89	33098	23918	43027
Urticaria	1990	Incidence	Number	Male	90-94	7408	5369	10017
Urticaria	1990	Incidence	Number	Male	95+	1453	963	2107
Urticaria	1990	Incidence	Rate	Female	<5	3780.14	3045.03	4671.32
Urticaria	1990	Incidence	Rate	Female	5-9	2524.86	1747.93	3644.86
Urticaria	1990	Incidence	Rate	Female	10-14	1847.5	1273.56	2643.87
Urticaria	1990	Incidence	Rate	Female	15-19	1679.5	1165.46	2300.02
Urticaria	1990	Incidence	Rate	Female	20-24	1623.45	1179.11	2230.35
Urticaria	1990	Incidence	Rate	Female	25-29	1550.35	1100.09	2071.16
Urticaria	1990	Incidence	Rate	Female	30-34	1497.49	1027.57	2037.52
Urticaria	1990	Incidence	Rate	Female	35-39	1440.66	1010.98	2015.16

Urticaria	1990	Incidence	Rate	Female	40-44	1398.14	998.90	1941.98
Urticaria	1990	Incidence	Rate	Female	45-49	1363.56	955.32	1872.79
Urticaria	1990	Incidence	Rate	Female	50-54	1396.89	982.57	1855.47
Urticaria	1990	Incidence	Rate	Female	55-59	1393.29	992.10	1868.34
Urticaria	1990	Incidence	Rate	Female	60-64	1433.21	1031.72	1942.89
Urticaria	1990	Incidence	Rate	Female	65-69	1446.54	1025.90	1962.06
Urticaria	1990	Incidence	Rate	Female	70-74	1372.8	1003.92	1881.71
Urticaria	1990	Incidence	Rate	Female	75-79	1254.11	902.50	1722.22
Urticaria	1990	Incidence	Rate	Female	80-84	1070.75	724.09	1439.02
Urticaria	1990	Incidence	Rate	Female	85-89	868.66	628.77	1129.42
Urticaria	1990	Incidence	Rate	Female	90-94	712.84	519.85	954.02
Urticaria	1990	Incidence	Rate	Female	95+	609.24	411.94	879.18
Urticaria	1990	Incidence	Rate	Male	<5	2690.22	2189.88	3315.29
Urticaria	1990	Incidence	Rate	Male	5-9	1798.39	1248.08	2603.32
Urticaria	1990	Incidence	Rate	Male	10-14	1299.57	885.88	1813.78
Urticaria	1990	Incidence	Rate	Male	15-19	1134.89	786.82	1566.65
Urticaria	1990	Incidence	Rate	Male	20-24	1066.16	768.36	1487.61
Urticaria	1990	Incidence	Rate	Male	25-29	992.47	687.75	1325.29
Urticaria	1990	Incidence	Rate	Male	30-34	939.46	633.81	1295.44
Urticaria	1990	Incidence	Rate	Male	35-39	898.02	627.89	1273.57
Urticaria	1990	Incidence	Rate	Male	40-44	856.32	601.47	1199.40
Urticaria	1990	Incidence	Rate	Male	45-49	841.27	583.04	1153.54
Urticaria	1990	Incidence	Rate	Male	50-54	853.29	589.89	1145.97
Urticaria	1990	Incidence	Rate	Male	55-59	859.02	604.93	1151.52
Urticaria	1990	Incidence	Rate	Male	60-64	873.04	618.46	1197.50
Urticaria	1990	Incidence	Rate	Male	65-69	878.63	627.81	1201.24

Urticaria	1990	Incidence	Rate	Male	70-74	852.05	618.00	1170.41
Urticaria	1990	Incidence	Rate	Male	75-79	803.83	582.09	1103.91
Urticaria	1990	Incidence	Rate	Male	80-84	736.27	492.64	998.28
Urticaria	1990	Incidence	Rate	Male	85-89	653.58	472.31	849.66
Urticaria	1990	Incidence	Rate	Male	90-94	588.41	426.50	795.66
Urticaria	1990	Incidence	Rate	Male	95+	558.17	369.97	809.62
Urticaria	2021	DALYs	Number	Female	<5	388119	249177	575891
Urticaria	2021	DALYs	Number	Female	5-9	314953	180133	498015
Urticaria	2021	DALYs	Number	Female	10-14	214604	124300	331730
Urticaria	2021	DALYs	Number	Female	15-19	173830	102988	283348
Urticaria	2021	DALYs	Number	Female	20-24	159877	93235	259911
Urticaria	2021	DALYs	Number	Female	25-29	153216	88007	244080
Urticaria	2021	DALYs	Number	Female	30-34	150895	90375	233818
Urticaria	2021	DALYs	Number	Female	35-39	134313	80491	205066
Urticaria	2021	DALYs	Number	Female	40-44	114439	64201	185205
Urticaria	2021	DALYs	Number	Female	45-49	103764	61126	159819
Urticaria	2021	DALYs	Number	Female	50-54	97566	57956	148465
Urticaria	2021	DALYs	Number	Female	55-59	87852	54078	131560
Urticaria	2021	DALYs	Number	Female	60-64	72943	43559	109931
Urticaria	2021	DALYs	Number	Female	65-69	65117	39714	97990
Urticaria	2021	DALYs	Number	Female	70-74	47372	29650	72418
Urticaria	2021	DALYs	Number	Female	75-79	27934	17127	42516
Urticaria	2021	DALYs	Number	Female	80-84	16707	10141	24818
Urticaria	2021	DALYs	Number	Female	85-89	7552	4748	11417
Urticaria	2021	DALYs	Number	Female	90-94	2592	1583	3940
Urticaria	2021	DALYs	Number	Female	95+	713	437	1083

Urticaria	2021	DALYs	Number	Male	<5	295027	187316	437271
Urticaria	2021	DALYs	Number	Male	5-9	237272	134891	374746
Urticaria	2021	DALYs	Number	Male	10-14	161838	94120	253640
Urticaria	2021	DALYs	Number	Male	15-19	128136	75186	207508
Urticaria	2021	DALYs	Number	Male	20-24	114161	66120	186137
Urticaria	2021	DALYs	Number	Male	25-29	107089	61101	169109
Urticaria	2021	DALYs	Number	Male	30-34	103874	61734	160617
Urticaria	2021	DALYs	Number	Male	35-39	92126	54735	142805
Urticaria	2021	DALYs	Number	Male	40-44	78271	43385	129748
Urticaria	2021	DALYs	Number	Male	45-49	70151	41361	109369
Urticaria	2021	DALYs	Number	Male	50-54	65167	37821	99179
Urticaria	2021	DALYs	Number	Male	55-59	56658	34782	85585
Urticaria	2021	DALYs	Number	Male	60-64	45050	27053	68925
Urticaria	2021	DALYs	Number	Male	65-69	37880	23143	57521
Urticaria	2021	DALYs	Number	Male	70-74	26103	16258	39521
Urticaria	2021	DALYs	Number	Male	75-79	14939	9041	23045
Urticaria	2021	DALYs	Number	Male	80-84	8135	4910	12131
Urticaria	2021	DALYs	Number	Male	85-89	3317	2030	4961
Urticaria	2021	DALYs	Number	Male	90-94	994	601	1503
Urticaria	2021	DALYs	Number	Male	95+	238	143	364
Urticaria	2021	DALYs	Rate	Female	<5	121.98	78.31	180.99
Urticaria	2021	DALYs	Rate	Female	5-9	94.7	54.16	149.74
Urticaria	2021	DALYs	Rate	Female	10-14	66.46	38.49	102.73
Urticaria	2021	DALYs	Rate	Female	15-19	57.25	33.92	93.31
Urticaria	2021	DALYs	Rate	Female	20-24	54.43	31.74	88.48
Urticaria	2021	DALYs	Rate	Female	25-29	52.65	30.24	83.88

Urticaria	2021	DALYs	Rate	Female	30-34	50.48	30.23	78.22
Urticaria	2021	DALYs	Rate	Female	35-39	48.35	28.97	73.82
Urticaria	2021	DALYs	Rate	Female	40-44	46.13	25.88	74.65
Urticaria	2021	DALYs	Rate	Female	45-49	44.03	25.94	67.82
Urticaria	2021	DALYs	Rate	Female	50-54	43.76	26.00	66.59
Urticaria	2021	DALYs	Rate	Female	55-59	43.71	26.90	65.45
Urticaria	2021	DALYs	Rate	Female	60-64	44.34	26.48	66.82
Urticaria	2021	DALYs	Rate	Female	65-69	45.22	27.58	68.04
Urticaria	2021	DALYs	Rate	Female	70-74	43.28	27.09	66.17
Urticaria	2021	DALYs	Rate	Female	75-79	38.74	23.76	58.97
Urticaria	2021	DALYs	Rate	Female	80-84	32.8	19.91	48.73
Urticaria	2021	DALYs	Rate	Female	85-89	26.53	16.68	40.10
Urticaria	2021	DALYs	Rate	Female	90-94	21.49	13.13	32.67
Urticaria	2021	DALYs	Rate	Female	95+	18.1	11.10	27.51
Urticaria	2021	DALYs	Rate	Male	<5	86.78	55.10	128.62
Urticaria	2021	DALYs	Rate	Male	5-9	66.94	38.05	105.72
Urticaria	2021	DALYs	Rate	Male	10-14	47.09	27.38	73.79
Urticaria	2021	DALYs	Rate	Male	15-19	40	23.47	64.78
Urticaria	2021	DALYs	Rate	Male	20-24	37.63	21.79	61.35
Urticaria	2021	DALYs	Rate	Male	25-29	36.01	20.55	56.87
Urticaria	2021	DALYs	Rate	Male	30-34	34	20.20	52.57
Urticaria	2021	DALYs	Rate	Male	35-39	32.55	19.34	50.45
Urticaria	2021	DALYs	Rate	Male	40-44	31.04	17.21	51.45
Urticaria	2021	DALYs	Rate	Male	45-49	29.49	17.39	45.98
Urticaria	2021	DALYs	Rate	Male	50-54	29.36	17.04	44.68
Urticaria	2021	DALYs	Rate	Male	55-59	29.1	17.86	43.95

Urticaria	2021	DALYs	Rate	Male	60-64	28.96	17.39	44.31
Urticaria	2021	DALYs	Rate	Male	65-69	28.73	17.55	43.63
Urticaria	2021	DALYs	Rate	Male	70-74	27.08	16.87	41.00
Urticaria	2021	DALYs	Rate	Male	75-79	24.99	15.12	38.55
Urticaria	2021	DALYs	Rate	Male	80-84	22.2	13.40	33.10
Urticaria	2021	DALYs	Rate	Male	85-89	19.23	11.77	28.75
Urticaria	2021	DALYs	Rate	Male	90-94	17.06	10.32	25.78
Urticaria	2021	DALYs	Rate	Male	95+	15.75	9.49	24.05
Urticaria	2021	Prevalence	Number	Female	<5	6310125	5132705	7827607
Urticaria	2021	Prevalence	Number	Female	5-9	5131590	3596489	7377786
Urticaria	2021	Prevalence	Number	Female	10-14	3521442	2412636	4981108
Urticaria	2021	Prevalence	Number	Female	15-19	2868815	2046740	4029908
Urticaria	2021	Prevalence	Number	Female	20-24	2653396	1889025	3630772
Urticaria	2021	Prevalence	Number	Female	25-29	2561635	1823309	3525770
Urticaria	2021	Prevalence	Number	Female	30-34	2544135	1765420	3504356
Urticaria	2021	Prevalence	Number	Female	35-39	2282491	1637691	3174676
Urticaria	2021	Prevalence	Number	Female	40-44	1953765	1349820	2807321
Urticaria	2021	Prevalence	Number	Female	45-49	1781341	1261532	2437581
Urticaria	2021	Prevalence	Number	Female	50-54	1687891	1148579	2261459
Urticaria	2021	Prevalence	Number	Female	55-59	1532624	1090856	2059185
Urticaria	2021	Prevalence	Number	Female	60-64	1286582	929983	1756383
Urticaria	2021	Prevalence	Number	Female	65-69	1165208	799716	1631474
Urticaria	2021	Prevalence	Number	Female	70-74	861851	623303	1187987
Urticaria	2021	Prevalence	Number	Female	75-79	517960	371900	716659
Urticaria	2021	Prevalence	Number	Female	80-84	315993	212805	425932
Urticaria	2021	Prevalence	Number	Female	85-89	145698	102419	191845

Urticaria	2021	Prevalence	Number	Female	90-94	50984	36104	68649
Urticaria	2021	Prevalence	Number	Female	95+	14393	10113	20190
Urticaria	2021	Prevalence	Number	Male	<5	4801639	3917015	5964173
Urticaria	2021	Prevalence	Number	Male	5-9	3862082	2751518	5572126
Urticaria	2021	Prevalence	Number	Male	10-14	2638274	1804694	3697954
Urticaria	2021	Prevalence	Number	Male	15-19	2091900	1478154	2925738
Urticaria	2021	Prevalence	Number	Male	20-24	1866855	1324255	2575374
Urticaria	2021	Prevalence	Number	Male	25-29	1759255	1238885	2437498
Urticaria	2021	Prevalence	Number	Male	30-34	1721568	1172582	2392773
Urticaria	2021	Prevalence	Number	Male	35-39	1536614	1084784	2181248
Urticaria	2021	Prevalence	Number	Male	40-44	1309212	890998	1878418
Urticaria	2021	Prevalence	Number	Male	45-49	1182014	824968	1622025
Urticaria	2021	Prevalence	Number	Male	50-54	1108797	748621	1500622
Urticaria	2021	Prevalence	Number	Male	55-59	974958	691821	1332889
Urticaria	2021	Prevalence	Number	Male	60-64	784378	564934	1084237
Urticaria	2021	Prevalence	Number	Male	65-69	671660	459520	946751
Urticaria	2021	Prevalence	Number	Male	70-74	471373	339372	644804
Urticaria	2021	Prevalence	Number	Male	75-79	275010	195922	377716
Urticaria	2021	Prevalence	Number	Male	80-84	152575	100845	207507
Urticaria	2021	Prevalence	Number	Male	85-89	63350	44902	84226
Urticaria	2021	Prevalence	Number	Male	90-94	19314	13611	26156
Urticaria	2021	Prevalence	Number	Male	95+	4725	3283	6704
Urticaria	2021	Prevalence	Rate	Female	<5	1983.12	1613.08	2460.02
Urticaria	2021	Prevalence	Rate	Female	5-9	1542.98	1081.40	2218.37
Urticaria	2021	Prevalence	Rate	Female	10-14	1090.48	747.12	1542.49
Urticaria	2021	Prevalence	Rate	Female	15-19	944.78	674.05	1327.16

Urticaria	2021	Prevalence	Rate	Female	20-24	903.28	643.07	1236.00
Urticaria	2021	Prevalence	Rate	Female	25-29	880.33	626.59	1211.66
Urticaria	2021	Prevalence	Rate	Female	30-34	851.08	590.58	1172.30
Urticaria	2021	Prevalence	Rate	Female	35-39	821.62	589.52	1142.78
Urticaria	2021	Prevalence	Rate	Female	40-44	787.52	544.09	1131.57
Urticaria	2021	Prevalence	Rate	Female	45-49	755.95	535.36	1034.44
Urticaria	2021	Prevalence	Rate	Female	50-54	757.1	515.19	1014.37
Urticaria	2021	Prevalence	Rate	Female	55-59	762.49	542.70	1024.45
Urticaria	2021	Prevalence	Rate	Female	60-64	782.06	565.30	1067.64
Urticaria	2021	Prevalence	Rate	Female	65-69	809.12	555.32	1132.90
Urticaria	2021	Prevalence	Rate	Female	70-74	787.45	569.50	1085.44
Urticaria	2021	Prevalence	Rate	Female	75-79	718.41	515.83	994.01
Urticaria	2021	Prevalence	Rate	Female	80-84	620.43	417.83	836.29
Urticaria	2021	Prevalence	Rate	Female	85-89	511.78	359.76	673.87
Urticaria	2021	Prevalence	Rate	Female	90-94	422.73	299.35	569.18
Urticaria	2021	Prevalence	Rate	Female	95+	365.48	256.78	512.65
Urticaria	2021	Prevalence	Rate	Male	<5	1412.33	1152.13	1754.28
Urticaria	2021	Prevalence	Rate	Male	5-9	1089.52	776.22	1571.93
Urticaria	2021	Prevalence	Rate	Male	10-14	767.59	525.06	1075.89
Urticaria	2021	Prevalence	Rate	Male	15-19	653.05	461.45	913.35
Urticaria	2021	Prevalence	Rate	Male	20-24	615.3	436.46	848.82
Urticaria	2021	Prevalence	Rate	Male	25-29	591.63	416.63	819.72
Urticaria	2021	Prevalence	Rate	Male	30-34	563.43	383.76	783.11
Urticaria	2021	Prevalence	Rate	Male	35-39	542.85	383.23	770.59
Urticaria	2021	Prevalence	Rate	Male	40-44	519.2	353.35	744.93
Urticaria	2021	Prevalence	Rate	Male	45-49	496.93	346.83	681.92

Urticaria	2021	Prevalence	Rate	Male	50-54	499.5	337.25	676.02
Urticaria	2021	Prevalence	Rate	Male	55-59	500.69	355.28	684.50
Urticaria	2021	Prevalence	Rate	Male	60-64	504.3	363.22	697.09
Urticaria	2021	Prevalence	Rate	Male	65-69	509.48	348.56	718.14
Urticaria	2021	Prevalence	Rate	Male	70-74	489.02	352.08	668.94
Urticaria	2021	Prevalence	Rate	Male	75-79	459.98	327.70	631.77
Urticaria	2021	Prevalence	Rate	Male	80-84	416.28	275.14	566.16
Urticaria	2021	Prevalence	Rate	Male	85-89	367.19	260.26	488.19
Urticaria	2021	Prevalence	Rate	Male	90-94	331.37	233.53	448.76
Urticaria	2021	Prevalence	Rate	Male	95+	312.49	217.15	443.37
Urticaria	2021	Incidence	Number	Female	<5	12190686	9833418	15117552
Urticaria	2021	Incidence	Number	Female	5-9	8511662	5880600	12260566
Urticaria	2021	Incidence	Number	Female	10-14	5977214	4145554	8506718
Urticaria	2021	Incidence	Number	Female	15-19	4972629	3455136	6796774
Urticaria	2021	Incidence	Number	Female	20-24	4635674	3357221	6335860
Urticaria	2021	Incidence	Number	Female	25-29	4463939	3169519	5986959
Urticaria	2021	Incidence	Number	Female	30-34	4433751	3033064	6059631
Urticaria	2021	Incidence	Number	Female	35-39	3982830	2795733	5580166
Urticaria	2021	Incidence	Number	Female	40-44	3399384	2420535	4730998
Urticaria	2021	Incidence	Number	Female	45-49	3117540	2181861	4294808
Urticaria	2021	Incidence	Number	Female	50-54	2967494	2079302	3958175
Urticaria	2021	Incidence	Number	Female	55-59	2688090	1915187	3612548
Urticaria	2021	Incidence	Number	Female	60-64	2279702	1641747	3085412
Urticaria	2021	Incidence	Number	Female	65-69	2042641	1444865	2780573
Urticaria	2021	Incidence	Number	Female	70-74	1496660	1096811	2058205
Urticaria	2021	Incidence	Number	Female	75-79	890762	637249	1231012

Urticaria	2021	Incidence	Number	Female	80-84	540469	364977	729482
Urticaria	2021	Incidence	Number	Female	85-89	247710	177784	325603
Urticaria	2021	Incidence	Number	Female	90-94	87710	63268	118281
Urticaria	2021	Incidence	Number	Female	95+	24802	16656	35875
Urticaria	2021	Incidence	Number	Male	<5	9279223	7538160	11477286
Urticaria	2021	Incidence	Number	Male	5-9	6406283	4445118	9279090
Urticaria	2021	Incidence	Number	Male	10-14	4465971	3041482	6238592
Urticaria	2021	Incidence	Number	Male	15-19	3617641	2521150	4967593
Urticaria	2021	Incidence	Number	Male	20-24	3259481	2353710	4574819
Urticaria	2021	Incidence	Number	Male	25-29	3063360	2127878	4092138
Urticaria	2021	Incidence	Number	Male	30-34	2999719	2018844	4149337
Urticaria	2021	Incidence	Number	Male	35-39	2684486	1873915	3802299
Urticaria	2021	Incidence	Number	Male	40-44	2278443	1596556	3189668
Urticaria	2021	Incidence	Number	Male	45-49	2071579	1433951	2848815
Urticaria	2021	Incidence	Number	Male	50-54	1952026	1351601	2633560
Urticaria	2021	Incidence	Number	Male	55-59	1710017	1205877	2293326
Urticaria	2021	Incidence	Number	Male	60-64	1388668	985173	1904838
Urticaria	2021	Incidence	Number	Male	65-69	1175373	837797	1610224
Urticaria	2021	Incidence	Number	Male	70-74	821319	596248	1126882
Urticaria	2021	Incidence	Number	Male	75-79	476095	344279	653055
Urticaria	2021	Incidence	Number	Male	80-84	263133	175515	356555
Urticaria	2021	Incidence	Number	Male	85-89	108701	78433	141921
Urticaria	2021	Incidence	Number	Male	90-94	33501	24191	45220
Urticaria	2021	Incidence	Number	Male	95+	8218	5433	11908
Urticaria	2021	Incidence	Rate	Female	<5	3831.23	3090.40	4751.07
Urticaria	2021	Incidence	Rate	Female	5-9	2559.3	1768.19	3686.53

Urticaria	2021	Incidence	Rate	Female	10-14	1850.95	1283.75	2634.26
Urticaria	2021	Incidence	Rate	Female	15-19	1637.62	1137.87	2238.36
Urticaria	2021	Incidence	Rate	Female	20-24	1578.09	1142.88	2156.87
Urticaria	2021	Incidence	Rate	Female	25-29	1534.07	1089.23	2057.46
Urticaria	2021	Incidence	Rate	Female	30-34	1483.2	1014.64	2027.10
Urticaria	2021	Incidence	Rate	Female	35-39	1433.69	1006.37	2008.68
Urticaria	2021	Incidence	Rate	Female	40-44	1370.22	975.67	1906.97
Urticaria	2021	Incidence	Rate	Female	45-49	1323	925.92	1822.59
Urticaria	2021	Incidence	Rate	Female	50-54	1331.06	932.66	1775.42
Urticaria	2021	Incidence	Rate	Female	55-59	1337.33	952.81	1797.26
Urticaria	2021	Incidence	Rate	Female	60-64	1385.75	997.96	1875.51
Urticaria	2021	Incidence	Rate	Female	65-69	1418.41	1003.32	1930.83
Urticaria	2021	Incidence	Rate	Female	70-74	1367.46	1002.13	1880.54
Urticaria	2021	Incidence	Rate	Female	75-79	1235.49	883.87	1707.42
Urticaria	2021	Incidence	Rate	Female	80-84	1061.17	716.61	1432.29
Urticaria	2021	Incidence	Rate	Female	85-89	870.1	624.48	1143.70
Urticaria	2021	Incidence	Rate	Female	90-94	727.23	524.57	980.70
Urticaria	2021	Incidence	Rate	Female	95+	629.76	422.93	910.92
Urticaria	2021	Incidence	Rate	Male	<5	2729.35	2217.24	3375.88
Urticaria	2021	Incidence	Rate	Male	5-9	1807.25	1254.00	2617.69
Urticaria	2021	Incidence	Rate	Male	10-14	1299.34	884.90	1815.07
Urticaria	2021	Incidence	Rate	Male	15-19	1129.35	787.05	1550.77
Urticaria	2021	Incidence	Rate	Male	20-24	1074.3	775.76	1507.82
Urticaria	2021	Incidence	Rate	Male	25-29	1030.2	715.60	1376.17
Urticaria	2021	Incidence	Rate	Male	30-34	981.75	660.73	1357.99
Urticaria	2021	Incidence	Rate	Male	35-39	948.37	662.01	1343.27

Urticaria	2021	Incidence	Rate	Male	40-44	903.57	633.15	1264.93
Urticaria	2021	Incidence	Rate	Male	45-49	870.92	602.85	1197.68
Urticaria	2021	Incidence	Rate	Male	50-54	879.37	608.88	1186.39
Urticaria	2021	Incidence	Rate	Male	55-59	878.17	619.27	1177.73
Urticaria	2021	Incidence	Rate	Male	60-64	892.82	633.40	1224.69
Urticaria	2021	Incidence	Rate	Male	65-69	891.56	635.50	1221.41
Urticaria	2021	Incidence	Rate	Male	70-74	852.07	618.57	1169.07
Urticaria	2021	Incidence	Rate	Male	75-79	796.32	575.84	1092.31
Urticaria	2021	Incidence	Rate	Male	80-84	717.92	478.87	972.82
Urticaria	2021	Incidence	Rate	Male	85-89	630.05	454.61	822.60
Urticaria	2021	Incidence	Rate	Male	90-94	574.78	415.05	775.84
Urticaria	2021	Incidence	Rate	Male	95+	543.48	359.33	787.55

Supplemental Table 3: ASPR, ASIR, ASDR, and EAPC values for the three allergic skin diseases across 21 regions in 1990 and 2021. (Age-standardized disability-adjusted life year rates: ASDR; Age-standardized incidence rates: ASIR; Age-standardized prevalence rates: ASPR).

Location	ASPR (per 100,000 people)		EAPC in ASPR (95% UI)	ASIR (per 100,000 people)		EAPC in ASIR (95% UI)	ASDR (per 100,000 people)		EAPC in ASDR (95% UI)
	1990	2021		1990	2021		1990	2021	
**Atopic dermatitis**									
Andean Latin America	1694.36 (1622.41,1772.01)	1692.65 (1620.76,1770.4)	0.01 (0, 0.02)	226.66 (214.67,239.34)	226.52 (214.55,239.16)	0.01 (0, 0.02)	73.7 (37.91,123.22)	73.89 (38.34,123.07)	0.03 (0.02, 0.04)
Australasia	2534.56 (2389.13,2672.41)	2535.67 (2392.77,2686.33)	0.01 (-0.01, 0.02)	338.61 (315.82,361.52)	338.68 (314.33,364.6)	0 (-0.01, 0.01)	110.82 (56.77,184.05)	111.17 (57.59,187.59)	0.01 (0, 0.02)
Caribbean	1947.47 (1842.36,2049.01)	1945.56 (1840.66,2046.99)	0 (0, 0)	263.22 (244.18,283.46)	263.09 (244,283.32)	0 (0, 0)	84.7 (43.93,141.95)	84.56 (44.1,142.12)	0 (-0.01, 0)
Central Asia	4399.59 (4163.54,4640.9)	4387.01 (4148.98,4628.2)	-0.01 (-0.01, -0.01)	414.92 (383.23,447.64)	413.83 (381.95,446.64)	-0.01 (-0.01, -0.01)	192.48 (99.3,323.8)	192.37 (98.8,324.14)	0 (0, 0)
Central Europe	1758.86 (1679.17,1836.85)	1774.87 (1691.64,1858.31)	0.02 (0.02, 0.03)	225.86 (212.63,241.46)	228.01 (214.91,244.41)	0.02 (0.01, 0.02)	77.58 (39.94,130.56)	78.36 (40.37,132.13)	0.03 (0.02, 0.04)
Central Latin America	1790.73 (1709.66,1874.78)	1773.33 (1690.09,1853.45)	-0.04 (-0.04, -0.03)	249.05 (234.79,264.98)	248.13 (233.79,263.46)	-0.02 (-0.03, -0.02)	78.34 (40.31,131.14)	77.72 (39.95,130.08)	-0.03 (-0.04, -0.02)
Central Sub-Saharan Africa	982.02 (920.51,1037.71)	981.16 (919.73,1036.77)	0 (-0.01, 0)	146.16 (135.79,157.19)	146.06 (135.69,157.03)	0 (-0.01, 0)	42.56 (21.73,72.23)	42.89 (22.11,71.52)	0.03 (0.02, 0.03)
East Asia	1380.79 (1320.67,1441.77)	1369.02 (1309.83,1425.29)	0 (-0.02, 0.02)	199.95 (189.77,210.52)	198.49 (189,208.52)	0 (-0.03, 0.02)	60.84 (31.52,102.16)	60.48 (31.37,101.51)	0.01 (-0.01, 0.03)
Eastern Europe	2856.81 (2762.95,2960.24)	3003.42 (2904.17,3107.67)	0.21 (0.17, 0.24)	211.61 (200.84,223.39)	222.53 (211.03,235.27)	0.22 (0.18, 0.27)	125.53 (64.4,207.81)	132.17 (67.33,219.48)	0.22 (0.18, 0.26)
Eastern Sub-Saharan Africa	1022.21 (977.67,1064.8)	1034.1 (987.87,1080.04)	0.05 (0.04, 0.06)	146.11 (138.01,154.02)	147.77 (139.31,155.78)	0.05 (0.04, 0.06)	44.33 (22.81,74.38)	45.19 (23.19,75.5)	0.08 (0.07, 0.09)
High-income Asia Pacific	4605.74 (4402.01,4811.25)	4596.03 (4402.96,4811.08)	-0.02 (-0.04, 0)	473.98 (447.59,501.7)	474.81 (448.38,505.41)	0 (-0.02, 0.01)	202.21 (103.15,337.13)	202.07 (103.47,337.3)	-0.02 (-0.04, 0)
High-income	3139.72	3131.86	-0.03	334.7	334.63	-0.02	136.34	135.37	-0.05

North America	(3036.67,3247.61)	(3023.58,3244.21)	(-0.06, -0.01)	(319.72,350.29)	(319.39,349.65)	(-0.03, 0)	(70.34,225.64)	(69.85,222.95)	(-0.07, -0.02)
North Africa and Middle East	1430.18 (1354.47,1502.71)	1374.93 (1300.26,1444.3)	-0.15 (-0.15, -0.14)	188.43 (176.31,200.36)	183.14 (171.05,194.83)	-0.11 (-0.11, -0.11)	62.58 (32.42,105.74)	60.23 (30.95,101.13)	-0.14 (-0.14, -0.13)
Oceania	1725.64 (1632.78,1833.8)	1725.48 (1632.52,1833.58)	0 (0, 0)	236.05 (217.85,254.74)	235.99 (217.79,254.67)	0 (0, 0)	75.31 (38.83,126.44)	75.49 (38.93,127.9)	0.01 (0, 0.01)
South Asia	1466.66 (1400.48,1539.32)	1467.56 (1400.88,1540.43)	-0.02 (-0.03, -0.01)	207.18 (195.37,220.53)	207.29 (195.41,220.57)	-0.02 (-0.02, -0.01)	63.8 (32.98,107.16)	64.23 (33.06,106.96)	0 (-0.01, 0.01)
Southeast Asia	1645.5 (1575.15,1719.11)	1645.49 (1571.3,1719.24)	0 (0, 0.01)	220.97 (208.65,233.73)	221.53 (209.56,233.87)	0.01 (0.01, 0.02)	71.93 (37.13,122.04)	72.22 (37.33,122.5)	0.02 (0.02, 0.02)
Southern Latin America	3213.96 (3034.27,3397.32)	3210.54 (3031.03,3393.55)	0 (0, 0)	388.03 (357.85,417.58)	387.79 (357.7,417.31)	0 (0, 0)	140.54 (71.91,237.06)	140.43 (71.94,236.14)	0 (-0.01, 0)
Southern Sub-Saharan Africa	1041.19 (994.77,1090.18)	1039.97 (992.88,1089.07)	0 (-0.01, 0)	152.89 (143.91,161.75)	152.75 (143.72,161.57)	0 (0, 0)	45.53 (23.63,76.7)	45.48 (23.48,76.47)	0 (0, 0)
Tropical Latin America	2362.95 (2270.41,2460.92)	2365.36 (2272.94,2464.23)	0 (-0.01, 0)	300.93 (286.23,315.73)	301.13 (286.24,316.05)	0 (-0.01, 0)	102.14 (53.31,168.78)	102.49 (53.33,169.99)	0.01 (0, 0.02)
Western Europe	3770.89 (3598.11,3954.62)	3761.1 (3583.37,3938.45)	-0.02 (-0.03, 0)	423.07 (395.94,450.91)	421.72 (394.56,448.97)	-0.02 (-0.02, -0.01)	164.94 (85.25,275.87)	164.47 (85.21,275.32)	-0.02 (-0.03, 0)
Western Sub-Saharan Africa	1048.05 (1004.21,1090.18)	1052.92 (1008.44,1095.34)	0.02 (0.02, 0.03)	146.5 (139.16,153.87)	147 (139.61,154.2)	0.02 (0.01, 0.02)	45.49 (23.41,76.98)	45.94 (23.77,77.64)	0.05 (0.04, 0.06)
**Urticaria**									
Andean Latin America	809 (715.86,926.73)	807.42 (714.46,924.84)	-0.01 (-0.01, -0.01)	1427.75 (1254.03,1619.46)	1425.03 (1251.78,1616.54)	-0.01 (-0.01, -0.01)	48.61 (31.84,68.63)	48.6 (32.02,69.46)	0.01 (0, 0.01)
Australasia	913.25 (800.65,1030.78)	913.62 (800.93,1031.53)	0 (0, 0)	1606.36 (1409.74,1807.18)	1606.94 (1410.36,1807.09)	0 (0, 0)	54.67 (36.03,77.29)	54.71 (36.1,77.09)	0 (0, 0.01)
Caribbean	809.59 (716.29,927.44)	808.61 (715.52,926.2)	0 (-0.01, 0)	1428.75 (1254.92,1620.56)	1427.06 (1253.54,1618.72)	0 (-0.01, 0)	48.59 (32.08,69.28)	48.44 (32.15,68.24)	-0.01 (-0.01, -0.01)
Central Asia	1055.52 (930.03,1211.74)	1051.2 (926,1207.3)	-0.01 (-0.01, -0.01)	1858.39 (1631.88,2128.08)	1850.83 (1624.93,2118.64)	-0.01 (-0.01, -0.01)	63.53 (42.49,90)	63.43 (42.24,89.23)	0 (0, 0)
Central Europe	1136.47	1123.19	-0.02	2002.02	1978.56	-0.02	68.59	67.9	-0.01

	(1012.93,1293.06)	(1011,1259.44)	(-0.03, -0.01)	(1779.1,2256.77)	(1775.54,2205.44)	(-0.03, -0.01)	(45.59,97.43)	(44.99,96.14)	(-0.02, 0)
Central Latin America	833.09 (734.88,950.72)	833.13 (734.93,950.79)	0 (0, 0)	1470.52 (1294.04,1672.2)	1470.59 (1294.44,1672.07)	0 (0, 0)	50.03 (33,71.26)	50.14 (32.96,71.36)	0 (0, 0.01)
Central Sub-Saharan Africa	810.26 (716.83,928.23)	809.57 (716.24,927.18)	0 (0, 0)	1429.93 (1256.1,1621.83)	1428.72 (1255.13,1620.52)	0 (0, 0)	48.01 (31.79,67.72)	48.33 (31.74,69.09)	0.03 (0.03, 0.04)
East Asia	757.92 (668.24,859.6)	755.03 (666,855.88)	-0.01 (-0.01, -0.01)	1340.63 (1174.89,1518.79)	1335.66 (1170.92,1512.77)	-0.01 (-0.01, -0.01)	45.79 (30.24,65.52)	45.74 (30.08,65.31)	0 (0, 0)
Eastern Europe	1107.06 (974.72,1273.49)	1104.06 (971.74,1270.35)	-0.01 (-0.01, 0)	1950.45 (1712.69,2226.45)	1945.3 (1707.69,2221.08)	0 (-0.01, 0)	66.66 (44.1,94.62)	66.56 (44.29,95.09)	0 (0, 0.01)
Eastern Sub-Saharan Africa	826.72 (730.64,944.8)	826.44 (730.34,944.74)	0 (0, 0)	1459.22 (1283.34,1657.61)	1458.66 (1282.96,1656.27)	0 (0, 0)	49.17 (32.52,70.04)	49.48 (32.8,70.6)	0.03 (0.03, 0.03)
High-income Asia Pacific	800.9 (708.04,910.4)	795.19 (701.95,905.77)	-0.02 (-0.02, -0.02)	1413.23 (1246.69,1602.65)	1403.51 (1237.7,1592.59)	-0.02 (-0.02, -0.02)	48.36 (31.97,68.65)	48.1 (31.64,68.95)	-0.02 (-0.02, -0.02)
High-income North America	889.16 (833.44,949.24)	907.37 (856.56,962.28)	0.02 (0, 0.03)	1569.62 (1461.56,1669.2)	1602.35 (1507.35,1697.23)	0.02 (0, 0.04)	53.48 (35.96,73.69)	54.35 (36.57,75.72)	0.01 (-0.01, 0.02)
North Africa and Middle East	935.26 (824.61,1059.5)	936.13 (825.75,1058.59)	0 (0, 0)	1648.18 (1454.89,1855.73)	1649.2 (1452.74,1854.32)	0 (0, 0)	56.03 (36.87,79.13)	56.1 (36.77,79.22)	0.01 (0.01, 0.01)
Oceania	716.48 (633.78,820.58)	716.27 (633.63,820.42)	0 (0, 0)	1267.03 (1110.92,1447.69)	1266.63 (1110.74,1446.97)	0 (0, 0)	42.86 (28.3,61.75)	43 (28.37,61.56)	0.01 (0.01, 0.01)
South Asia	981.1 (865,1118.1)	990.98 (874.14,1128.31)	0.03 (0.03, 0.03)	1732.92 (1532.88,1964.07)	1750.38 (1549.58,1983.76)	0.03 (0.03, 0.03)	58.29 (38.49,83.21)	59.23 (38.94,85.47)	0.05 (0.05, 0.06)
Southeast Asia	770.44 (673.9,878.97)	772.28 (675.82,881.4)	0.01 (0.01, 0.01)	1364 (1195.71,1552.36)	1367.36 (1198.53,1557.15)	0.01 (0.01, 0.01)	46.28 (30.35,66.38)	46.55 (30.63,66.94)	0.02 (0.02, 0.03)
Southern Latin America	774.83 (684.84,880.47)	773.21 (683.36,878.83)	-0.01 (-0.01, 0)	1366.47 (1207.25,1546.16)	1363.55 (1204.53,1542.91)	-0.01 (-0.01, 0)	46.47 (30.42,66.32)	46.44 (30.46,66.53)	0 (-0.01, 0)
Southern Sub-Saharan Africa	844.5 (744.98,965.35)	842.67 (743.59,962.52)	-0.01 (-0.01, -0.01)	1490.58 (1313.63,1694)	1487.38 (1311.16,1690.56)	-0.01 (-0.01, -0.01)	50.49 (33.07,72.26)	50.2 (33.28,70.91)	-0.01 (-0.02, -0.01)
Tropical Latin America	853.46 (752.53,974.22)	852.78 (752.13,973.34)	0 (0, 0)	1506.71 (1327.6,1709.31)	1505.54 (1326.59,1707.65)	0 (0, 0)	50.93 (33.67,72.69)	50.97 (33.52,72)	0.01 (0.01, 0.01)
Western Europe	591.49 (527.97,656.99)	594.23 (530.15,659.57)	0.01 (0.01, 0.02)	1046.11 (934.19,1152.84)	1050.96 (938.24,1158.35)	0.02 (0.01, 0.02)	35.15 (23.31,48.76)	35.31 (23.53,49)	0.02 (0.01, 0.02)

Western Sub-Saharan Africa	829.43 (732.19,947.26)	832.89 (735.18,950.59)	0.02 (0.01, 0.02)	1463.93 (1287.21,1664.7)	1470.06 (1293.73,1671.38)	0.02 (0.01, 0.02)	49.31 (32.6,70.29)	49.8 (32.84,70.86)	0.04 (0.03, 0.05)
**Contact dermatitis**									
Andean Latin America	1285.87 (1065.78,1547.61)	1282.86 (1062.92,1545.15)	-0.01 (-0.01, -0.01)	3424.29 (2798.93,4227.24)	3419.6 (2796.01,4221.58)	-0.01 (-0.01, 0)	31.85 (19.54,48.5)	31.75 (19.86,48.02)	-0.01 (-0.01, -0.01)
Australasia	278.86 (225.75,340.05)	279.26 (226.13,340.45)	0 (0, 0.01)	783.18 (639.47,963.89)	784.36 (640.58,965.83)	0 (0, 0.01)	6.86 (4.32,10.4)	6.88 (4.26,10.76)	0.01 (0, 0.01)
Caribbean	1090.2 (887.31,1335.31)	1090.26 (887.18,1334.73)	0 (0, 0)	3003.11 (2439.17,3733.19)	3003.33 (2439.36,3733.14)	0 (0, 0)	26.93 (16.52,40.98)	26.83 (16.4,40.53)	-0.01 (-0.01, -0.01)
Central Asia	1281.14 (1051.5,1558.93)	1276.06 (1047.05,1552.07)	-0.01 (-0.01, -0.01)	3480.09 (2834.21,4297.21)	3466.8 (2822.7,4278.07)	-0.01 (-0.01, -0.01)	31.68 (19.59,47.92)	31.53 (19.61,48.03)	-0.01 (-0.01, 0)
Central Europe	1317.89 (1081.04,1591.47)	1317.59 (1081.67,1588.55)	0 (0, 0)	3599.74 (2924.38,4437)	3600.64 (2923.43,4439.67)	0 (0, 0)	32.43 (20.14,48.8)	32.49 (19.96,48.81)	0.01 (0.01, 0.02)
Central Latin America	1163.25 (940.78,1426.63)	1165.77 (942.92,1430.08)	0.01 (0, 0.01)	3222.61 (2603.41,4023.98)	3229.55 (2608.02,4033.88)	0.01 (0, 0.01)	28.6 (17.75,43.5)	28.65 (17.86,43.6)	0.01 (0, 0.01)
Central Sub-Saharan Africa	893.67 (718.08,1087.61)	894.12 (718.45,1087.96)	0 (0, 0)	2476.74 (1990.85,3091.8)	2478.08 (1992.3,3091.65)	0 (0, 0)	21.71 (13.49,33)	21.84 (13.64,33.03)	0.03 (0.02, 0.03)
East Asia	1317.66 (1058.33,1609.19)	1317.94 (1058.1,1609.49)	0 (0, 0)	3655.88 (2942.07,4604.48)	3656.98 (2943.67,4610.87)	0 (0, 0)	32.73 (20.1,49.66)	32.78 (20.01,49.64)	0.01 (0, 0.01)
Eastern Europe	1199.33 (1014.71,1415.73)	1172.97 (996.83,1378.39)	-0.05 (-0.06, -0.04)	3290.24 (2719.61,3996.93)	3215.8 (2670.2,3879.14)	-0.05 (-0.06, -0.04)	29.54 (19.22,43.68)	28.91 (18.65,42.41)	-0.04 (-0.05, -0.03)
Eastern Sub-Saharan Africa	934.96 (756.72,1145.01)	938.22 (759.78,1147.72)	0.01 (0.01, 0.01)	2598.45 (2094.16,3230.05)	2607.26 (2100.63,3239.08)	0.01 (0.01, 0.01)	22.84 (14.12,34.48)	23 (14.14,34.92)	0.03 (0.03, 0.04)
High-income Asia Pacific	302.19 (245.9,369.07)	296.7 (241.99,362.74)	-0.06 (-0.06, -0.06)	846.7 (688.42,1046.94)	831.86 (677.29,1028.37)	-0.06 (-0.06, -0.06)	7.51 (4.7,11.38)	7.38 (4.52,11.28)	-0.05 (-0.06, -0.05)
High-income North America	1932.78 (1604.78,2297.53)	1367.33 (1154.47,1607.48)	-0.48 (-0.72, -0.23)	4625.51 (3778.5,5707.22)	3427.23 (2823.99,4188.54)	-0.41 (-0.62, -0.2)	47.77 (29.95,70.47)	33.52 (21.32,49.12)	-0.5 (-0.74, -0.25)
North Africa and	996.99	998.89	0.01 (0.01,	2763.59	2767.99	0.01 (0.01,	24.5	24.47	0 (0, 0)

Middle East	(810.16,1211.14)	(811.63,1213.1)	0.01)	(2236.09,3397.9)	(2241.3,3399.49)	0.01)	(15.12,36.93)	(15.01,37.02)	
Oceania	1174.95 (962.39,1430.1)	1175.32 (962.39,1430.32)	0 (0, 0)	3215.12 (2605.32,4007.76)	3216.17 (2606.59,4009.23)	0 (0, 0)	28.92 (18.09,44.32)	28.95 (18.01,44.81)	0 (0, 0.01)
South Asia	1068.5 (865.18,1303.34)	1070.5 (867.59,1305.35)	0.01 (0.01, 0.01)	2961.56 (2400.97,3647.42)	2967.26 (2404.05,3657.34)	0.01 (0.01, 0.01)	25.99 (16.01,39.52)	26.13 (16.12,39.87)	0.02 (0.02, 0.02)
Southeast Asia	1459.46 (1184.74,1800.92)	1457.38 (1182.83,1798.7)	-0.01 (-0.01, -0.01)	3953.68 (3202.83,4951.29)	3951.85 (3199.96,4949.23)	0 (-0.01, 0)	36.17 (22.4,55.15)	36.21 (22.34,55.45)	0 (0, 0.01)
Southern Latin America	274.04 (221.56,332.41)	273.83 (221.41,332.16)	0 (0, 0)	770.16 (629.94,944.35)	769.55 (629.46,943.68)	0 (0, 0)	6.79 (4.24,10.3)	6.77 (4.15,10.35)	0 (-0.01, 0)
Southern Sub-Saharan Africa	983.83 (795.01,1201.4)	987.46 (797.68,1204.94)	0.01 (0.01, 0.02)	2739.67 (2200.99,3389.67)	2749.53 (2207.61,3400.43)	0.01 (0.01, 0.02)	24.08 (14.87,36.55)	23.94 (14.74,36.16)	-0.01 (-0.02, -0.01)
Tropical Latin America	1231.13 (987.75,1517.55)	1232.07 (988.91,1518.55)	0 (0, 0)	3425.68 (2752.32,4313.93)	3428.24 (2753.46,4316.55)	0 (0, 0)	30.03 (18.59,45.55)	30.05 (18.65,45.75)	0.01 (0.01, 0.01)
Western Europe	640.47 (528,765.44)	630.83 (519.43,756.49)	-0.05 (-0.06, -0.05)	1595.11 (1286.36,1983.58)	1574.06 (1271.28,1956.52)	-0.04 (-0.05, -0.04)	15.93 (9.84,23.48)	15.68 (9.61,23.41)	-0.05 (-0.06, -0.05)
Western Sub-Saharan Africa	984.62 (801.63,1200.87)	980.88 (798.05,1197.14)	-0.01 (-0.02, -0.01)	2694.92 (2195.02,3340.45)	2690.48 (2190.83,3332.02)	-0.01 (-0.01, -0.01)	24.08 (14.94,36.39)	24.11 (14.76,36.5)	0.01 (0.01, 0.01)

Supplemental Table 4: Distribution of ASIR, ASPR, and ASDR across different regions or countries in 1990 and 2021. (Age-standardized disability-adjusted life year rates: ASDR; Age-standardized incidence rates: ASIR; Age-standardized prevalence rates: ASPR).

Measure name	Location name	Cause name	Year	Value	Upper	Lower
ASPR	Afghanistan	AD	2021	1350.54	1425.72	1279.81
ASIR	Afghanistan	AD	2021	184.16	196.48	171.92
ASDR	Afghanistan	AD	2021	58.89	99.41	30.36
ASPR	Afghanistan	AD	1990	1355.66	1430.77	1284.24
ASIR	Afghanistan	AD	1990	184.51	196.78	172.31
ASDR	Afghanistan	AD	1990	58.90	99.77	30.23
ASPR	Albania	AD	2021	1797.12	1895.48	1697.98
ASIR	Albania	AD	2021	231.56	248.95	215.94
ASDR	Albania	AD	2021	79.42	136.00	41.55
ASPR	Albania	AD	1990	1795.49	1894.08	1696.79
ASIR	Albania	AD	1990	230.99	248.41	215.45
ASDR	Albania	AD	1990	79.15	136.42	41.10
ASPR	Algeria	AD	2021	1348.44	1469.49	1233.05
ASIR	Algeria	AD	2021	184.23	199.93	168.79
ASDR	Algeria	AD	2021	59.29	98.23	30.61
ASPR	Algeria	AD	1990	1349.23	1470.54	1233.78
ASIR	Algeria	AD	1990	184.35	200.06	168.91
ASDR	Algeria	AD	1990	59.14	97.30	30.42
ASPR	American Samoa	AD	2021	1728.01	1836.11	1634.81
ASIR	American Samoa	AD	2021	236.37	255.07	218.12
ASDR	American Samoa	AD	2021	75.70	128.73	38.58
ASPR	American Samoa	AD	1990	1727.48	1835.49	1634.68
ASIR	American Samoa	AD	1990	236.16	254.87	217.94
ASDR	American Samoa	AD	1990	75.77	129.34	38.47
ASPR	Andorra	AD	2021	3373.14	3523.86	3211.25
ASIR	Andorra	AD	2021	395.44	424.35	366.20
ASDR	Andorra	AD	2021	147.67	246.24	76.82
ASPR	Andorra	AD	1990	3362.41	3512.86	3200.75
ASIR	Andorra	AD	1990	394.58	423.53	365.40
ASDR	Andorra	AD	1990	147.49	249.30	77.37
ASPR	Angola	AD	2021	986.20	1041.51	924.56
ASIR	Angola	AD	2021	146.67	157.75	136.24

ASDR	Angola	AD	2021	43.27	72.38	22.35
ASPR	Angola	AD	1990	984.02	1039.22	922.60
ASIR	Angola	AD	1990	146.42	157.51	136.01
ASDR	Angola	AD	1990	42.90	72.45	21.81
ASPR	Antigua and Barbuda	AD	2021	1945.85	2047.34	1840.88
ASIR	Antigua and Barbuda	AD	2021	263.12	283.38	244.05
ASDR	Antigua and Barbuda	AD	2021	84.82	142.57	43.84
ASPR	Antigua and Barbuda	AD	1990	1949.45	2051.18	1844.25
ASIR	Antigua and Barbuda	AD	1990	263.46	283.72	244.44
ASDR	Antigua and Barbuda	AD	1990	84.98	142.33	44.36
ASPR	Argentina	AD	2021	3211.48	3394.53	3031.98
ASIR	Argentina	AD	2021	387.82	417.34	357.72
ASDR	Argentina	AD	2021	140.49	236.68	71.65
ASPR	Argentina	AD	1990	3214.17	3397.61	3034.41
ASIR	Argentina	AD	1990	388.08	417.64	357.88
ASDR	Argentina	AD	1990	140.57	237.26	71.65
ASPR	Armenia	AD	2021	4383.20	4625.70	4144.06
ASIR	Armenia	AD	2021	413.28	446.01	381.47
ASDR	Armenia	AD	2021	192.57	320.82	97.98
ASPR	Armenia	AD	1990	4398.68	4640.95	4158.21
ASIR	Armenia	AD	1990	414.65	447.58	382.76
ASDR	Armenia	AD	1990	192.60	321.76	99.56
ASPR	Australia	AD	2021	2362.08	2526.64	2209.25
ASIR	Australia	AD	2021	323.06	350.95	298.28
ASDR	Australia	AD	2021	103.67	174.29	53.97
ASPR	Australia	AD	1990	2365.21	2515.47	2208.64
ASIR	Australia	AD	1990	322.98	346.59	298.64
ASDR	Australia	AD	1990	103.50	171.68	53.20
ASPR	Austria	AD	2021	3372.32	3523.92	3211.09
ASIR	Austria	AD	2021	395.23	424.18	365.95
ASDR	Austria	AD	2021	147.93	246.43	75.72
ASPR	Austria	AD	1990	3377.47	3529.64	3216.66
ASIR	Austria	AD	1990	395.54	424.45	366.22
ASDR	Austria	AD	1990	147.96	246.53	77.45
ASPR	Azerbaijan	AD	2021	4371.03	4613.17	4132.09
ASIR	Azerbaijan	AD	2021	412.26	444.88	380.52
ASDR	Azerbaijan	AD	2021	191.94	320.47	98.16
ASPR	Azerbaijan	AD	1990	4402.67	4645.18	4162.19

ASIR	Azerbaijan	AD	1990	414.72	447.66	382.86
ASDR	Azerbaijan	AD	1990	192.92	327.90	99.06
ASPR	Bahamas	AD	2021	1948.80	2050.52	1843.80
ASIR	Bahamas	AD	2021	263.35	283.61	244.30
ASDR	Bahamas	AD	2021	84.99	144.89	43.88
ASPR	Bahamas	AD	1990	1948.14	2049.83	1843.16
ASIR	Bahamas	AD	1990	263.35	283.61	244.29
ASDR	Bahamas	AD	1990	84.98	140.81	44.83
ASPR	Bahrain	AD	2021	1340.11	1415.30	1269.58
ASIR	Bahrain	AD	2021	183.24	195.68	171.04
ASDR	Bahrain	AD	2021	58.97	101.22	30.52
ASPR	Bahrain	AD	1990	1344.77	1419.92	1274.22
ASIR	Bahrain	AD	1990	183.61	196.04	171.37
ASDR	Bahrain	AD	1990	59.03	101.44	30.51
ASPR	Bangladesh	AD	2021	1466.63	1553.54	1381.03
ASIR	Bangladesh	AD	2021	205.42	222.33	190.45
ASDR	Bangladesh	AD	2021	64.49	109.07	32.61
ASPR	Bangladesh	AD	1990	1463.70	1550.75	1378.48
ASIR	Bangladesh	AD	1990	205.09	221.99	190.06
ASDR	Bangladesh	AD	1990	63.97	108.26	32.87
ASPR	Barbados	AD	2021	1945.43	2046.87	1840.46
ASIR	Barbados	AD	2021	263.08	283.33	243.98
ASDR	Barbados	AD	2021	84.85	143.46	43.97
ASPR	Barbados	AD	1990	1948.26	2050.05	1843.23
ASIR	Barbados	AD	1990	263.42	283.67	244.33
ASDR	Barbados	AD	1990	85.15	143.94	44.64
ASPR	Belarus	AD	2021	1936.16	2013.45	1860.26
ASIR	Belarus	AD	2021	181.36	192.83	170.39
ASDR	Belarus	AD	2021	85.32	145.21	43.34
ASPR	Belarus	AD	1990	1943.29	2021.12	1867.15
ASIR	Belarus	AD	1990	182.02	193.53	171.01
ASDR	Belarus	AD	1990	85.47	143.90	43.75
ASPR	Belgium	AD	2021	3377.15	3528.91	3215.77
ASIR	Belgium	AD	2021	395.55	424.50	366.26
ASDR	Belgium	AD	2021	147.98	245.92	77.06
ASPR	Belgium	AD	1990	3376.50	3528.48	3215.36
ASIR	Belgium	AD	1990	395.47	424.39	366.18
ASDR	Belgium	AD	1990	147.89	245.22	77.33
ASPR	Belize	AD	2021	1946.46	2047.88	1841.34
ASIR	Belize	AD	2021	263.15	283.38	244.11
ASDR	Belize	AD	2021	84.76	142.42	44.21
ASPR	Belize	AD	1990	1944.63	2045.86	1839.79
ASIR	Belize	AD	1990	262.98	283.16	243.91
ASDR	Belize	AD	1990	84.86	146.05	44.67

ASPR	Benin	AD	2021	1042.30	1089.44	995.94
ASIR	Benin	AD	2021	145.62	153.80	137.21
ASDR	Benin	AD	2021	45.50	77.72	23.01
ASPR	Benin	AD	1990	1042.38	1089.41	996.10
ASIR	Benin	AD	1990	145.61	153.77	137.20
ASDR	Benin	AD	1990	45.25	76.17	23.49
ASPR	Bermuda	AD	2021	1946.24	2047.66	1841.27
ASIR	Bermuda	AD	2021	263.12	283.34	244.04
ASDR	Bermuda	AD	2021	84.99	142.95	43.60
ASPR	Bermuda	AD	1990	1947.73	2049.41	1842.81
ASIR	Bermuda	AD	1990	263.29	283.54	244.20
ASDR	Bermuda	AD	1990	85.19	144.46	43.61
ASPR	Bhutan	AD	2021	1464.87	1552.51	1379.49
ASIR	Bhutan	AD	2021	205.32	222.33	190.30
ASDR	Bhutan	AD	2021	64.17	107.89	32.86
ASPR	Bhutan	AD	1990	1461.75	1550.12	1376.87
ASIR	Bhutan	AD	1990	205.17	222.15	190.16
ASDR	Bhutan	AD	1990	63.81	106.78	33.07
ASPR	Bolivia (Plurinational State of)	AD	2021	1700.52	1781.64	1625.67
ASIR	Bolivia (Plurinational State of)	AD	2021	227.24	240.36	214.55
ASDR	Bolivia (Plurinational State of)	AD	2021	73.97	124.91	38.00
ASPR	Bolivia (Plurinational State of)	AD	1990	1702.50	1783.50	1627.52
ASIR	Bolivia (Plurinational State of)	AD	1990	227.39	240.54	214.69
ASDR	Bolivia (Plurinational State of)	AD	1990	73.87	125.97	38.54
ASPR	Bosnia and Herzegovina	AD	2021	1801.69	1900.84	1702.66
ASIR	Bosnia and Herzegovina	AD	2021	232.03	249.53	216.38
ASDR	Bosnia and Herzegovina	AD	2021	79.56	135.18	40.85
ASPR	Bosnia and Herzegovina	AD	1990	1802.96	1902.14	1703.90

ASIR	Bosnia and Herzegovina	AD	1990	232.27	249.80	216.59
ASDR	Bosnia and Herzegovina	AD	1990	79.50	133.89	41.50
ASPR	Botswana	AD	2021	1036.68	1101.39	978.53
ASIR	Botswana	AD	2021	151.54	164.54	139.94
ASDR	Botswana	AD	2021	45.34	79.04	22.91
ASPR	Botswana	AD	1990	1039.19	1104.55	981.21
ASIR	Botswana	AD	1990	151.77	164.81	140.16
ASDR	Botswana	AD	1990	45.49	78.99	23.16
ASPR	Brazil	AD	2021	2363.70	2463.95	2271.91
ASIR	Brazil	AD	2021	301.19	316.19	286.17
ASDR	Brazil	AD	2021	102.41	169.88	53.31
ASPR	Brazil	AD	1990	2361.53	2460.88	2269.63
ASIR	Brazil	AD	1990	300.96	315.76	286.20
ASDR	Brazil	AD	1990	102.07	168.67	53.29
ASPR	Brunei Darussalam	AD	2021	3823.19	4041.61	3624.54
ASIR	Brunei Darussalam	AD	2021	421.93	451.23	390.99
ASDR	Brunei Darussalam	AD	2021	167.94	283.33	86.20
ASPR	Brunei Darussalam	AD	1990	3824.15	4042.49	3624.80
ASIR	Brunei Darussalam	AD	1990	421.88	451.27	390.90
ASDR	Brunei Darussalam	AD	1990	167.82	280.00	85.90
ASPR	Bulgaria	AD	2021	1800.75	1899.80	1701.71
ASIR	Bulgaria	AD	2021	231.96	249.48	216.30
ASDR	Bulgaria	AD	2021	79.48	134.56	40.42
ASPR	Bulgaria	AD	1990	1803.76	1903.19	1704.67
ASIR	Bulgaria	AD	1990	232.36	249.87	216.68
ASDR	Bulgaria	AD	1990	79.53	138.39	40.70
ASPR	Burkina Faso	AD	2021	1043.59	1090.72	997.16
ASIR	Burkina Faso	AD	2021	145.74	153.93	137.31
ASDR	Burkina Faso	AD	2021	45.41	77.59	23.54
ASPR	Burkina Faso	AD	1990	1043.42	1090.60	997.07
ASIR	Burkina Faso	AD	1990	145.74	153.93	137.31
ASDR	Burkina Faso	AD	1990	45.22	76.84	23.08
ASPR	Burundi	AD	2021	985.51	1031.92	941.15
ASIR	Burundi	AD	2021	143.31	151.88	133.71
ASDR	Burundi	AD	2021	43.15	72.30	22.32
ASPR	Burundi	AD	1990	987.37	1033.66	943.07

ASIR	Burundi	AD	1990	143.60	152.17	133.98
ASDR	Burundi	AD	1990	42.97	71.39	22.39
ASPR	Côte d'Ivoire	AD	2021	1038.25	1085.40	992.31
ASIR	Côte d'Ivoire	AD	2021	145.20	153.38	136.81
ASPR	Côte d'Ivoire	AD	1990	1039.21	1086.36	993.03
ASIR	Côte d'Ivoire	AD	1990	145.27	153.43	136.88
ASDR	Côte d'Ivoire	AD	1990	45.11	75.69	22.92
ASPR	Cabo Verde	AD	2021	1040.74	1087.81	994.53
ASIR	Cabo Verde	AD	2021	145.50	153.66	137.07
ASDR	Cabo Verde	AD	2021	45.65	77.59	23.37
ASPR	Cabo Verde	AD	1990	1045.06	1092.37	998.76
ASIR	Cabo Verde	AD	1990	145.99	154.18	137.55
ASDR	Cabo Verde	AD	1990	45.71	77.85	23.43
ASPR	Cambodia	AD	2021	1641.82	1727.78	1555.10
ASIR	Cambodia	AD	2021	219.88	235.86	205.19
ASDR	Cambodia	AD	2021	71.80	122.94	37.00
ASPR	Cambodia	AD	1990	1661.51	1748.60	1572.41
ASIR	Cambodia	AD	1990	221.07	237.32	206.38
ASDR	Cambodia	AD	1990	72.21	122.53	37.36
ASPR	Cameroon	AD	2021	1246.75	1306.11	1190.18
ASIR	Cameroon	AD	2021	162.67	172.36	153.19
ASDR	Cameroon	AD	2021	54.54	91.98	27.78
ASPR	Cameroon	AD	1990	1249.61	1304.75	1197.67
ASIR	Cameroon	AD	1990	162.80	173.20	153.38
ASDR	Cameroon	AD	1990	54.35	90.30	28.00
ASPR	Canada	AD	2021	2855.17	2972.52	2747.90
ASIR	Canada	AD	2021	316.37	334.49	299.46
ASDR	Canada	AD	2021	124.20	206.89	63.70
ASPR	Canada	AD	1990	2856.58	2973.78	2749.00
ASIR	Canada	AD	1990	316.39	334.44	299.47
ASDR	Canada	AD	1990	124.29	205.57	64.95
ASPR	Central African Republic	AD	2021	985.20	1040.35	923.69
ASIR	Central African Republic	AD	2021	146.53	157.58	136.12
ASDR	Central African Republic	AD	2021	42.87	71.84	22.20
ASPR	Central African Republic	AD	1990	985.01	1040.33	923.47

ASIR	Central African Republic	AD	1990	146.55	157.62	136.14
ASDR	Central African Republic	AD	1990	42.78	73.02	21.80
ASPR	Chad	AD	2021	1041.13	1088.49	994.84
ASIR	Chad	AD	2021	145.46	153.67	137.05
ASDR	Chad	AD	2021	45.42	77.45	23.34
ASPR	Chad	AD	1990	1043.50	1090.77	997.11
ASIR	Chad	AD	1990	145.74	153.94	137.30
ASDR	Chad	AD	1990	45.35	77.34	23.20
ASPR	Chile	AD	2021	3208.04	3390.95	3028.48
ASIR	Chile	AD	2021	387.73	417.26	357.64
ASDR	Chile	AD	2021	140.32	239.85	72.50
ASPR	Chile	AD	1990	3213.67	3396.86	3034.14
ASIR	Chile	AD	1990	387.93	417.46	357.81
ASDR	Chile	AD	1990	140.44	236.42	71.91
ASPR	China	AD	2021	1347.11	1402.16	1288.65
ASIR	China	AD	2021	196.63	206.73	187.20
ASDR	China	AD	2021	59.53	99.89	30.92
ASPR	China	AD	1990	1357.93	1417.71	1298.98
ASIR	China	AD	1990	197.94	208.35	187.94
ASDR	China	AD	1990	59.85	100.45	31.01
ASPR	Colombia	AD	2021	1877.87	1984.99	1772.21
ASIR	Colombia	AD	2021	258.38	278.07	239.97
ASDR	Colombia	AD	2021	82.32	136.26	42.01
ASPR	Colombia	AD	1990	1879.99	1987.47	1774.65
ASIR	Colombia	AD	1990	258.47	278.20	240.02
ASDR	Colombia	AD	1990	82.22	135.72	42.05
ASPR	Comoros	AD	2021	985.02	1031.18	940.88
ASIR	Comoros	AD	2021	143.30	151.83	133.71
ASDR	Comoros	AD	2021	43.21	71.51	22.38
ASPR	Comoros	AD	1990	985.60	1031.79	941.35
ASIR	Comoros	AD	1990	143.36	151.93	133.78
ASDR	Comoros	AD	1990	43.14	73.66	22.43
ASPR	Congo	AD	2021	788.56	848.50	725.30
ASIR	Congo	AD	2021	123.77	133.86	113.57
ASDR	Congo	AD	2021	34.52	58.95	17.71
ASPR	Congo	AD	1990	789.68	849.72	726.57
ASIR	Congo	AD	1990	123.95	134.05	113.75
ASDR	Congo	AD	1990	34.48	58.67	17.56
ASPR	Cook Islands	AD	2021	1737.11	1845.35	1644.05
ASIR	Cook Islands	AD	2021	236.94	255.60	218.58

ASDR	Cook Islands	AD	2021	76.36	127.99	38.92
ASPR	Cook Islands	AD	1990	1725.40	1833.65	1632.67
ASIR	Cook Islands	AD	1990	235.98	254.67	217.76
ASDR	Cook Islands	AD	1990	75.90	126.03	38.81
ASPR	Costa Rica	AD	2021	1880.18	1987.54	1774.56
ASIR	Costa Rica	AD	2021	258.55	278.25	240.09
ASDR	Costa Rica	AD	2021	82.21	137.08	42.39
ASPR	Costa Rica	AD	1990	1877.64	1984.91	1772.30
ASIR	Costa Rica	AD	1990	258.29	277.98	239.87
ASDR	Costa Rica	AD	1990	82.24	138.90	41.92
ASDR	Côte d'Ivoire	AD	2021	45.46	78.17	23.43
ASPR	Croatia	AD	2021	1801.58	1900.75	1702.53
ASIR	Croatia	AD	2021	232.06	249.57	216.40
ASDR	Croatia	AD	2021	79.69	138.06	41.09
ASPR	Croatia	AD	1990	1804.14	1903.49	1705.13
ASIR	Croatia	AD	1990	232.28	249.81	216.63
ASDR	Croatia	AD	1990	79.76	134.16	41.45
ASPR	Cuba	AD	2021	1942.18	2043.40	1837.62
ASIR	Cuba	AD	2021	262.74	282.94	243.58
ASDR	Cuba	AD	2021	84.75	141.90	44.18
ASPR	Cuba	AD	1990	1943.17	2044.36	1838.30
ASIR	Cuba	AD	1990	262.80	283.01	243.69
ASDR	Cuba	AD	1990	84.79	142.82	45.08
ASPR	Cyprus	AD	2021	3043.36	3314.61	2774.13
ASIR	Cyprus	AD	2021	375.77	406.32	343.03
ASDR	Cyprus	AD	2021	133.31	219.19	67.98
ASPR	Cyprus	AD	1990	3042.60	3313.62	2773.83
ASIR	Cyprus	AD	1990	375.77	406.30	343.02
ASDR	Cyprus	AD	1990	133.37	220.16	68.13
ASPR	Czechia	AD	2021	1801.04	1900.25	1701.99
ASIR	Czechia	AD	2021	232.05	249.58	216.36
ASDR	Czechia	AD	2021	79.61	134.84	40.89
ASPR	Czechia	AD	1990	1804.63	1904.09	1705.53
ASIR	Czechia	AD	1990	232.44	249.96	216.75
ASDR	Czechia	AD	1990	79.62	135.23	40.75
ASPR	Democratic People's Republic of Korea	AD	2021	1745.08	1841.26	1648.62
ASIR	Democratic People's Republic of Korea	AD	2021	234.28	249.79	219.67

ASDR	Democratic People's Republic of Korea	AD	2021	76.71	130.38	39.57
ASPR	Democratic People's Republic of Korea	AD	1990	1754.08	1852.02	1658.38
ASIR	Democratic People's Republic of Korea	AD	1990	235.09	250.67	220.49
ASDR	Democratic People's Republic of Korea	AD	1990	76.92	133.09	39.14
ASPR	Democratic Republic of the Congo	AD	2021	983.82	1038.90	922.42
ASIR	Democratic Republic of the Congo	AD	2021	146.40	157.46	136.01
ASDR	Democratic Republic of the Congo	AD	2021	42.96	72.45	21.96
ASPR	Democratic Republic of the Congo	AD	1990	984.81	1040.19	923.30
ASIR	Democratic Republic of the Congo	AD	1990	146.53	157.64	136.11
ASDR	Democratic Republic of the Congo	AD	1990	42.59	72.83	21.76
ASPR	Denmark	AD	2021	4298.20	4646.22	3937.65
ASIR	Denmark	AD	2021	452.19	486.90	413.12
ASDR	Denmark	AD	2021	188.08	313.32	98.83
ASPR	Denmark	AD	1990	4300.78	4648.98	3940.61
ASIR	Denmark	AD	1990	452.33	487.03	413.23
ASDR	Denmark	AD	1990	187.72	311.57	97.69
ASPR	Djibouti	AD	2021	978.74	1024.81	935.11
ASIR	Djibouti	AD	2021	142.61	151.10	133.09
ASDR	Djibouti	AD	2021	43.02	71.70	22.29
ASPR	Djibouti	AD	1990	981.26	1027.24	937.30

ASIR	Djibouti	AD	1990	142.84	151.33	133.31
ASDR	Djibouti	AD	1990	42.85	72.21	22.18
ASPR	Dominica	AD	2021	1943.57	2044.83	1838.86
ASIR	Dominica	AD	2021	262.95	283.12	243.86
ASDR	Dominica	AD	2021	84.59	139.57	43.84
ASPR	Dominica	AD	1990	1943.70	2045.30	1839.10
ASIR	Dominica	AD	1990	263.03	283.28	243.86
ASDR	Dominica	AD	1990	84.83	144.17	43.77
ASPR	Dominican Republic	AD	2021	1944.45	2045.76	1839.64
ASIR	Dominican Republic	AD	2021	262.97	283.18	243.86
ASDR	Dominican Republic	AD	2021	84.91	143.07	44.54
ASPR	Dominican Republic	AD	1990	1948.92	2050.53	1843.71
ASIR	Dominican Republic	AD	1990	263.30	283.53	244.30
ASDR	Dominican Republic	AD	1990	84.96	140.53	44.35
ASPR	Ecuador	AD	2021	1728.94	1812.27	1654.14
ASIR	Ecuador	AD	2021	229.38	244.19	216.45
ASDR	Ecuador	AD	2021	75.53	125.00	39.02
ASPR	Ecuador	AD	1990	1729.91	1813.37	1655.25
ASIR	Ecuador	AD	1990	229.42	244.22	216.49
ASDR	Ecuador	AD	1990	75.29	126.72	39.12
ASPR	Egypt	AD	2021	833.60	886.43	777.57
ASIR	Egypt	AD	2021	128.84	138.11	118.69
ASDR	Egypt	AD	2021	36.78	61.04	19.20
ASPR	Egypt	AD	1990	845.50	902.15	786.86
ASIR	Egypt	AD	1990	130.68	140.44	120.88
ASDR	Egypt	AD	1990	37.15	64.30	19.23
ASPR	El Salvador	AD	2021	1881.17	1988.55	1775.09
ASIR	El Salvador	AD	2021	258.56	278.15	240.08
ASDR	El Salvador	AD	2021	82.24	136.72	42.22
ASPR	El Salvador	AD	1990	1881.21	1988.69	1775.67
ASIR	El Salvador	AD	1990	258.61	278.32	240.13
ASDR	El Salvador	AD	1990	81.92	137.31	42.03
ASPR	Equatorial Guinea	AD	2021	977.39	1031.56	917.77
ASIR	Equatorial Guinea	AD	2021	145.81	156.89	135.43
ASDR	Equatorial Guinea	AD	2021	42.73	73.01	22.05

ASPR	Equatorial Guinea	AD	1990	985.48	1040.74	924.03
ASIR	Equatorial Guinea	AD	1990	146.52	157.56	136.14
ASDR	Equatorial Guinea	AD	1990	42.50	71.02	21.74
ASPR	Eritrea	AD	2021	984.45	1030.54	940.56
ASIR	Eritrea	AD	2021	143.27	151.76	133.68
ASDR	Eritrea	AD	2021	43.05	73.23	22.19
ASPR	Eritrea	AD	1990	985.27	1031.30	941.29
ASIR	Eritrea	AD	1990	143.35	151.88	133.78
ASDR	Eritrea	AD	1990	42.72	72.09	21.64
ASPR	Estonia	AD	2021	4113.27	4287.48	3947.84
ASIR	Estonia	AD	2021	227.75	242.01	211.63
ASDR	Estonia	AD	2021	180.86	299.30	91.83
ASPR	Estonia	AD	1990	4130.96	4306.00	3965.73
ASIR	Estonia	AD	1990	229.15	243.46	212.96
ASDR	Estonia	AD	1990	181.33	305.93	93.56
ASPR	Eswatini	AD	2021	1036.49	1101.16	978.27
ASIR	Eswatini	AD	2021	151.53	164.54	139.93
ASDR	Eswatini	AD	2021	45.38	78.48	23.12
ASPR	Eswatini	AD	1990	1039.67	1105.18	981.66
ASIR	Eswatini	AD	1990	151.84	164.87	140.23
ASDR	Eswatini	AD	1990	45.65	77.34	23.61
ASPR	Ethiopia	AD	2021	900.19	943.42	861.51
ASIR	Ethiopia	AD	2021	135.63	142.73	128.95
ASDR	Ethiopia	AD	2021	39.41	66.59	20.31
ASPR	Ethiopia	AD	1990	900.45	943.73	861.85
ASIR	Ethiopia	AD	1990	135.64	142.75	128.94
ASDR	Ethiopia	AD	1990	39.11	65.06	20.23
ASPR	Fiji	AD	2021	1729.05	1837.38	1635.73
ASIR	Fiji	AD	2021	236.49	255.18	218.25
ASDR	Fiji	AD	2021	75.80	127.05	38.77
ASPR	Fiji	AD	1990	1729.23	1837.51	1635.99
ASIR	Fiji	AD	1990	236.46	255.14	218.22
ASDR	Fiji	AD	1990	75.77	125.96	39.52
ASPR	Finland	AD	2021	3374.64	3526.08	3213.28
ASIR	Finland	AD	2021	395.46	424.38	366.19
ASDR	Finland	AD	2021	147.89	248.19	76.28
ASPR	Finland	AD	1990	3378.06	3530.19	3217.21
ASIR	Finland	AD	1990	395.64	424.52	366.32
ASDR	Finland	AD	1990	147.81	248.22	76.56
ASPR	France	AD	2021	4704.28	5039.86	4397.67
ASIR	France	AD	2021	474.06	515.16	436.90

ASDR	France	AD	2021	205.27	345.38	105.28
ASPR	France	AD	1990	4703.81	5039.26	4397.52
ASIR	France	AD	1990	474.02	515.10	436.86
ASDR	France	AD	1990	205.25	346.14	105.27
ASPR	Gabon	AD	2021	1313.16	1405.14	1219.23
ASIR	Gabon	AD	2021	181.16	195.62	165.88
ASDR	Gabon	AD	2021	57.47	96.53	29.99
ASPR	Gabon	AD	1990	1315.99	1417.91	1226.24
ASIR	Gabon	AD	1990	181.66	196.82	166.68
ASDR	Gabon	AD	1990	57.20	95.38	30.09
ASPR	Gambia	AD	2021	1042.07	1089.25	995.72
ASIR	Gambia	AD	2021	145.58	153.78	137.17
ASDR	Gambia	AD	2021	45.52	78.96	23.51
ASPR	Gambia	AD	1990	1041.06	1088.29	994.74
ASIR	Gambia	AD	1990	145.45	153.67	137.03
ASDR	Gambia	AD	1990	45.40	77.62	23.16
ASPR	Georgia	AD	2021	4365.07	4609.71	4148.19
ASIR	Georgia	AD	2021	412.64	443.88	379.39
ASDR	Georgia	AD	2021	191.72	328.19	96.85
ASPR	Georgia	AD	1990	4352.43	4582.52	4131.33
ASIR	Georgia	AD	1990	411.89	444.71	377.59
ASDR	Georgia	AD	1990	191.08	318.58	98.19
ASPR	Germany	AD	2021	3460.05	3626.20	3298.40
ASIR	Germany	AD	2021	400.88	427.86	373.93
ASDR	Germany	AD	2021	151.29	252.14	77.56
ASPR	Germany	AD	1990	3469.69	3620.55	3327.08
ASIR	Germany	AD	1990	402.12	429.35	376.27
ASDR	Germany	AD	1990	151.95	253.79	78.04
ASPR	Ghana	AD	2021	860.11	896.31	824.34
ASIR	Ghana	AD	2021	127.69	134.86	121.31
ASDR	Ghana	AD	2021	37.65	63.52	19.30
ASPR	Ghana	AD	1990	859.29	895.49	823.45
ASIR	Ghana	AD	1990	127.57	134.71	121.21
ASDR	Ghana	AD	1990	37.44	63.41	19.63
ASPR	Greece	AD	2021	2973.01	3244.68	2730.98
ASIR	Greece	AD	2021	371.25	402.38	340.45
ASDR	Greece	AD	2021	130.25	211.99	67.56
ASPR	Greece	AD	1990	2949.56	3201.36	2720.73
ASIR	Greece	AD	1990	368.91	400.69	338.16
ASDR	Greece	AD	1990	129.37	212.59	66.04
ASPR	Greenland	AD	2021	2841.89	2960.00	2735.11
ASIR	Greenland	AD	2021	315.73	334.11	298.90
ASDR	Greenland	AD	2021	123.14	206.07	63.72
ASPR	Greenland	AD	1990	2826.11	2943.71	2719.68

ASIR	Greenland	AD	1990	315.27	333.39	298.46
ASDR	Greenland	AD	1990	122.73	204.45	64.14
ASPR	Grenada	AD	2021	1941.76	2042.97	1837.28
ASIR	Grenada	AD	2021	262.79	282.95	243.65
ASDR	Grenada	AD	2021	84.47	141.34	43.84
ASPR	Grenada	AD	1990	1947.16	2048.83	1842.29
ASIR	Grenada	AD	1990	263.37	283.61	244.27
ASDR	Grenada	AD	1990	84.74	141.02	43.89
ASPR	Guam	AD	2021	1726.14	1834.36	1632.85
ASIR	Guam	AD	2021	236.23	254.92	218.02
ASDR	Guam	AD	2021	75.87	128.80	39.42
ASPR	Guam	AD	1990	1720.46	1828.03	1627.18
ASIR	Guam	AD	1990	235.90	254.60	217.77
ASDR	Guam	AD	1990	75.71	127.90	38.41
ASPR	Guatemala	AD	2021	1881.69	1989.16	1775.93
ASIR	Guatemala	AD	2021	258.70	278.45	240.24
ASDR	Guatemala	AD	2021	82.19	136.91	41.63
ASPR	Guatemala	AD	1990	1881.96	1989.75	1776.49
ASIR	Guatemala	AD	1990	258.61	278.38	240.14
ASDR	Guatemala	AD	1990	81.74	136.34	41.97
ASPR	Guinea	AD	2021	1042.58	1089.84	996.22
ASIR	Guinea	AD	2021	145.62	153.82	137.20
ASDR	Guinea	AD	2021	45.52	77.80	23.69
ASPR	Guinea	AD	1990	1042.45	1089.64	996.15
ASIR	Guinea	AD	1990	145.61	153.80	137.18
ASDR	Guinea	AD	1990	45.37	77.06	23.49
ASPR	Guinea-Bissau	AD	2021	1042.74	1089.89	996.41
ASIR	Guinea-Bissau	AD	2021	145.69	153.88	137.28
ASDR	Guinea-Bissau	AD	2021	45.54	77.36	23.50
ASPR	Guinea-Bissau	AD	1990	1043.64	1090.99	997.25
ASIR	Guinea-Bissau	AD	1990	145.79	153.98	137.35
ASDR	Guinea-Bissau	AD	1990	45.25	76.27	23.50
ASPR	Guyana	AD	2021	1946.39	2047.87	1841.43
ASIR	Guyana	AD	2021	263.10	283.34	244.03
ASDR	Guyana	AD	2021	84.41	140.19	43.61
ASPR	Guyana	AD	1990	1948.17	2049.81	1843.21
ASIR	Guyana	AD	1990	263.24	283.46	244.19
ASDR	Guyana	AD	1990	84.48	141.41	44.16
ASPR	Haiti	AD	2021	1947.80	2049.40	1842.62
ASIR	Haiti	AD	2021	263.29	283.57	244.26
ASDR	Haiti	AD	2021	84.08	142.33	43.36
ASPR	Haiti	AD	1990	1951.40	2053.18	1845.83
ASIR	Haiti	AD	1990	263.56	283.81	244.60
ASDR	Haiti	AD	1990	84.04	141.83	43.24

ASPR	Honduras	AD	2021	1880.53	1988.01	1774.93
ASIR	Honduras	AD	2021	258.56	278.25	240.10
ASDR	Honduras	AD	2021	82.10	139.84	42.16
ASPR	Honduras	AD	1990	1879.47	1986.80	1774.10
ASIR	Honduras	AD	1990	258.44	278.15	239.99
ASDR	Honduras	AD	1990	81.93	136.03	41.68
ASPR	Hungary	AD	2021	2622.78	2814.44	2444.24
ASIR	Hungary	AD	2021	296.46	324.96	272.22
ASDR	Hungary	AD	2021	115.28	193.44	59.18
ASPR	Hungary	AD	1990	2628.81	2820.56	2449.61
ASIR	Hungary	AD	1990	297.05	325.61	272.78
ASDR	Hungary	AD	1990	115.41	192.85	59.24
ASPR	Iceland	AD	2021	3792.06	4100.35	3497.59
ASIR	Iceland	AD	2021	423.32	459.33	389.46
ASDR	Iceland	AD	2021	165.87	274.12	85.51
ASPR	Iceland	AD	1990	3813.00	4127.68	3505.58
ASIR	Iceland	AD	1990	424.90	458.54	390.78
ASDR	Iceland	AD	1990	167.00	278.31	87.17
ASPR	India	AD	2021	1468.09	1541.41	1400.25
ASIR	India	AD	2021	207.53	220.95	196.07
ASDR	India	AD	2021	64.22	106.61	33.00
ASPR	India	AD	1990	1467.50	1540.79	1399.68
ASIR	India	AD	1990	207.51	220.90	196.05
ASDR	India	AD	1990	63.78	107.26	32.92
ASPR	Indonesia	AD	2021	1649.30	1723.76	1580.82
ASIR	Indonesia	AD	2021	222.89	235.30	210.90
ASDR	Indonesia	AD	2021	72.45	120.99	37.88
ASPR	Indonesia	AD	1990	1639.17	1711.50	1568.08
ASIR	Indonesia	AD	1990	220.99	233.08	208.91
ASDR	Indonesia	AD	1990	71.72	119.94	36.88
ASPR	Iran (Islamic Republic of)	AD	2021	1538.60	1612.00	1466.98
ASIR	Iran (Islamic Republic of)	AD	2021	204.18	216.84	192.55
ASDR	Iran (Islamic Republic of)	AD	2021	67.53	113.83	34.36
ASPR	Iran (Islamic Republic of)	AD	1990	1518.56	1581.75	1449.29
ASIR	Iran (Islamic Republic of)	AD	1990	202.42	214.00	191.18
ASDR	Iran (Islamic Republic of)	AD	1990	66.47	110.91	34.40
ASPR	Iraq	AD	2021	1349.58	1424.78	1278.89
ASIR	Iraq	AD	2021	184.06	196.39	171.83

ASDR	Iraq	AD	2021	59.18	99.36	30.04
ASPR	Iraq	AD	1990	1349.93	1425.13	1279.29
ASIR	Iraq	AD	1990	184.11	196.43	171.88
ASDR	Iraq	AD	1990	59.08	100.34	30.54
ASPR	Ireland	AD	2021	3378.93	3530.80	3217.45
ASIR	Ireland	AD	2021	395.59	424.57	366.27
ASDR	Ireland	AD	2021	147.80	248.32	77.07
ASPR	Ireland	AD	1990	3376.83	3528.82	3215.57
ASIR	Ireland	AD	1990	395.43	424.40	366.13
ASDR	Ireland	AD	1990	147.79	250.02	76.21
ASPR	Israel	AD	2021	3376.70	3528.44	3215.45
ASIR	Israel	AD	2021	395.49	424.45	366.20
ASDR	Israel	AD	2021	148.17	246.83	77.35
ASPR	Israel	AD	1990	3379.49	3531.53	3218.41
ASIR	Israel	AD	1990	395.62	424.62	366.28
ASDR	Israel	AD	1990	148.18	243.70	77.00
ASPR	Italy	AD	2021	4141.92	4318.33	3982.12
ASIR	Italy	AD	2021	453.41	480.54	427.77
ASDR	Italy	AD	2021	181.10	305.36	94.01
ASPR	Italy	AD	1990	4147.57	4324.21	3987.49
ASIR	Italy	AD	1990	453.55	480.67	427.93
ASDR	Italy	AD	1990	181.25	304.27	94.05
ASPR	Jamaica	AD	2021	1945.33	2046.68	1840.37
ASIR	Jamaica	AD	2021	263.08	283.31	244.02
ASDR	Jamaica	AD	2021	84.77	144.20	43.97
ASPR	Jamaica	AD	1990	1948.13	2049.72	1842.96
ASIR	Jamaica	AD	1990	263.32	283.57	244.28
ASDR	Jamaica	AD	1990	85.04	145.76	43.97
ASPR	Japan	AD	2021	4892.58	5124.84	4683.90
ASIR	Japan	AD	2021	494.75	525.79	467.73
ASDR	Japan	AD	2021	215.10	358.88	109.77
ASPR	Japan	AD	1990	4885.26	5118.21	4663.48
ASIR	Japan	AD	1990	494.16	521.68	467.66
ASDR	Japan	AD	1990	214.48	357.97	109.22
ASPR	Jordan	AD	2021	1325.05	1422.86	1222.95
ASIR	Jordan	AD	2021	182.22	197.68	166.36
ASDR	Jordan	AD	2021	58.30	96.17	30.54
ASPR	Jordan	AD	1990	1326.04	1423.80	1223.87
ASIR	Jordan	AD	1990	182.35	197.84	166.55
ASDR	Jordan	AD	1990	58.29	95.90	29.92
ASPR	Kazakhstan	AD	2021	4398.90	4641.02	4158.21
ASIR	Kazakhstan	AD	2021	414.54	447.44	382.63
ASDR	Kazakhstan	AD	2021	193.06	324.16	99.32
ASPR	Kazakhstan	AD	1990	4404.42	4646.81	4163.56

ASIR	Kazakhstan	AD	1990	415.23	448.22	383.20
ASDR	Kazakhstan	AD	1990	192.55	321.58	99.21
ASPR	Kenya	AD	2021	880.76	921.84	840.40
ASIR	Kenya	AD	2021	133.53	140.24	126.59
ASDR	Kenya	AD	2021	38.64	64.59	19.91
ASPR	Kenya	AD	1990	834.33	871.13	800.43
ASIR	Kenya	AD	1990	126.92	133.01	121.15
ASDR	Kenya	AD	1990	36.50	61.79	18.86
ASPR	Kiribati	AD	2021	1734.55	1843.05	1640.83
ASIR	Kiribati	AD	2021	236.88	255.56	218.60
ASDR	Kiribati	AD	2021	75.78	126.01	38.52
ASPR	Kiribati	AD	1990	1733.36	1841.65	1639.73
ASIR	Kiribati	AD	1990	236.80	255.49	218.53
ASDR	Kiribati	AD	1990	75.57	126.94	38.92
ASPR	Kuwait	AD	2021	1426.21	1560.42	1308.13
ASIR	Kuwait	AD	2021	191.17	207.88	174.26
ASDR	Kuwait	AD	2021	62.72	104.89	31.81
ASPR	Kuwait	AD	1990	1432.71	1558.69	1313.23
ASIR	Kuwait	AD	1990	191.85	208.33	174.99
ASDR	Kuwait	AD	1990	63.07	103.64	32.61
ASPR	Kyrgyzstan	AD	2021	4395.71	4637.72	4155.15
ASIR	Kyrgyzstan	AD	2021	414.58	447.51	382.64
ASDR	Kyrgyzstan	AD	2021	192.76	321.42	99.34
ASPR	Kyrgyzstan	AD	1990	4403.33	4645.48	4162.29
ASIR	Kyrgyzstan	AD	1990	415.24	448.25	383.24
ASDR	Kyrgyzstan	AD	1990	192.97	320.71	99.34
ASPR	Lao People's Democratic Republic	AD	2021	1639.92	1725.89	1552.76
ASIR	Lao People's Democratic Republic	AD	2021	219.66	235.63	204.99
ASDR	Lao People's Democratic Republic	AD	2021	71.97	124.16	37.02
ASPR	Lao People's Democratic Republic	AD	1990	1651.19	1738.04	1562.70
ASIR	Lao People's Democratic Republic	AD	1990	220.40	236.48	205.71
ASDR	Lao People's Democratic Republic	AD	1990	72.10	121.36	36.93

ASPR	Latvia	AD	2021	871.63	907.33	836.42
ASIR	Latvia	AD	2021	104.03	109.82	98.40
ASDR	Latvia	AD	2021	38.56	66.52	19.75
ASPR	Latvia	AD	1990	876.07	913.75	837.34
ASIR	Latvia	AD	1990	104.71	110.79	98.95
ASDR	Latvia	AD	1990	38.70	65.84	19.62
ASPR	Lebanon	AD	2021	1349.30	1424.32	1278.67
ASIR	Lebanon	AD	2021	184.09	196.36	171.85
ASDR	Lebanon	AD	2021	59.15	100.39	30.06
ASPR	Lebanon	AD	1990	1351.51	1426.67	1280.62
ASIR	Lebanon	AD	1990	184.23	196.51	172.03
ASDR	Lebanon	AD	1990	59.05	99.60	30.51
ASPR	Lesotho	AD	2021	1038.02	1102.96	979.90
ASIR	Lesotho	AD	2021	151.73	164.77	140.10
ASDR	Lesotho	AD	2021	45.33	77.46	23.40
ASPR	Lesotho	AD	1990	1041.35	1107.11	983.64
ASIR	Lesotho	AD	1990	152.02	165.11	140.35
ASDR	Lesotho	AD	1990	45.61	76.52	23.65
ASPR	Liberia	AD	2021	1039.84	1086.98	993.69
ASIR	Liberia	AD	2021	145.33	153.53	136.94
ASDR	Liberia	AD	2021	45.19	77.24	23.38
ASPR	Liberia	AD	1990	1040.91	1088.18	994.60
ASIR	Liberia	AD	1990	145.45	153.63	137.03
ASDR	Liberia	AD	1990	44.94	77.90	23.27
ASPR	Libya	AD	2021	1350.50	1425.72	1279.81
ASIR	Libya	AD	2021	184.16	196.49	171.92
ASDR	Libya	AD	2021	59.32	103.37	30.20
ASPR	Libya	AD	1990	1350.98	1426.23	1280.21
ASIR	Libya	AD	1990	184.23	196.68	171.94
ASDR	Libya	AD	1990	59.55	103.37	30.65
ASPR	Lithuania	AD	2021	1935.98	2013.33	1859.99
ASIR	Lithuania	AD	2021	181.38	192.86	170.40
ASDR	Lithuania	AD	2021	85.12	141.00	43.35
ASPR	Lithuania	AD	1990	1942.31	2020.06	1866.13
ASIR	Lithuania	AD	1990	182.02	193.53	171.00
ASDR	Lithuania	AD	1990	85.50	146.13	43.87
ASPR	Luxembourg	AD	2021	3372.46	3524.12	3211.04
ASIR	Luxembourg	AD	2021	395.35	424.31	366.06
ASDR	Luxembourg	AD	2021	147.67	248.28	76.60
ASPR	Luxembourg	AD	1990	3376.93	3529.06	3216.09
ASIR	Luxembourg	AD	1990	395.58	424.51	366.26
ASDR	Luxembourg	AD	1990	147.88	246.81	77.00
ASPR	Madagascar	AD	2021	986.11	1032.36	941.76
ASIR	Madagascar	AD	2021	143.40	151.96	133.81

ASDR	Madagascar	AD	2021	43.14	74.30	22.65
ASPR	Madagascar	AD	1990	985.35	1031.52	941.01
ASIR	Madagascar	AD	1990	143.32	151.88	133.74
ASDR	Madagascar	AD	1990	42.87	71.38	22.22
ASPR	Malawi	AD	2021	987.27	1033.71	942.81
ASIR	Malawi	AD	2021	143.55	152.11	133.94
ASDR	Malawi	AD	2021	43.06	72.23	22.23
ASPR	Malawi	AD	1990	986.61	1032.93	942.28
ASIR	Malawi	AD	1990	143.48	152.05	133.88
ASDR	Malawi	AD	1990	42.65	72.24	21.51
ASPR	Malaysia	AD	2021	1630.76	1716.15	1544.92
ASIR	Malaysia	AD	2021	218.95	234.83	204.31
ASDR	Malaysia	AD	2021	71.54	122.17	36.80
ASPR	Malaysia	AD	1990	1640.00	1726.06	1553.02
ASIR	Malaysia	AD	1990	219.51	235.49	204.85
ASDR	Malaysia	AD	1990	71.70	121.56	36.79
ASPR	Maldives	AD	2021	1590.87	1672.41	1507.49
ASIR	Maldives	AD	2021	216.87	232.34	202.25
ASDR	Maldives	AD	2021	69.86	119.77	36.01
ASPR	Maldives	AD	1990	1636.81	1722.72	1549.34
ASIR	Maldives	AD	1990	219.14	235.07	204.46
ASDR	Maldives	AD	1990	71.20	123.42	37.08
ASPR	Mali	AD	2021	1146.01	1221.81	1070.13
ASIR	Mali	AD	2021	154.88	165.22	144.71
ASDR	Mali	AD	2021	49.75	84.10	25.92
ASPR	Mali	AD	1990	1152.05	1228.74	1071.04
ASIR	Mali	AD	1990	155.28	166.06	144.49
ASDR	Mali	AD	1990	49.89	84.32	25.62
ASPR	Malta	AD	2021	3370.11	3521.71	3208.82
ASIR	Malta	AD	2021	395.04	424.00	365.74
ASDR	Malta	AD	2021	147.74	249.28	76.22
ASPR	Malta	AD	1990	3376.57	3528.39	3215.42
ASIR	Malta	AD	1990	395.58	424.51	366.26
ASDR	Malta	AD	1990	147.97	245.59	76.88
ASPR	Marshall Islands	AD	2021	1727.86	1836.18	1634.66
ASIR	Marshall Islands	AD	2021	236.39	255.08	218.14
ASDR	Marshall Islands	AD	2021	75.71	129.74	38.87
ASPR	Marshall Islands	AD	1990	1728.60	1836.88	1635.55
ASIR	Marshall Islands	AD	1990	236.42	255.14	218.19

ASDR	Marshall Islands	AD	1990	75.62	128.84	38.88
ASPR	Mauritania	AD	2021	1041.97	1089.17	995.63
ASIR	Mauritania	AD	2021	145.57	153.75	137.16
ASDR	Mauritania	AD	2021	45.72	76.71	23.96
ASPR	Mauritania	AD	1990	1041.52	1088.58	995.25
ASIR	Mauritania	AD	1990	145.52	153.71	137.13
ASDR	Mauritania	AD	1990	45.41	78.17	23.21
ASPR	Mauritius	AD	2021	1641.06	1727.10	1553.66
ASIR	Mauritius	AD	2021	219.84	235.82	205.16
ASDR	Mauritius	AD	2021	71.82	122.07	36.95
ASPR	Mauritius	AD	1990	1640.42	1726.32	1553.18
ASIR	Mauritius	AD	1990	219.86	235.83	205.19
ASDR	Mauritius	AD	1990	71.68	122.64	36.45
ASPR	Mexico	AD	2021	1575.14	1644.57	1504.27
ASIR	Mexico	AD	2021	229.25	242.62	217.62
ASDR	Mexico	AD	2021	69.19	116.51	35.65
ASPR	Mexico	AD	1990	1604.64	1674.77	1534.21
ASIR	Mexico	AD	1990	231.20	244.50	219.35
ASDR	Mexico	AD	1990	70.35	119.19	36.21
ASPR	Micronesia (Federated States of)	AD	2021	1727.76	1836.00	1634.48
ASIR	Micronesia (Federated States of)	AD	2021	236.38	255.08	218.16
ASDR	Micronesia (Federated States of)	AD	2021	75.77	128.21	39.16
ASPR	Micronesia (Federated States of)	AD	1990	1728.33	1836.57	1635.51
ASIR	Micronesia (Federated States of)	AD	1990	236.32	255.03	218.10
ASDR	Micronesia (Federated States of)	AD	1990	75.57	125.81	39.03
ASPR	Monaco	AD	2021	3382.85	3534.37	3221.18
ASIR	Monaco	AD	2021	396.04	424.93	366.68
ASDR	Monaco	AD	2021	148.14	247.67	77.03
ASPR	Monaco	AD	1990	3373.98	3525.88	3213.08
ASIR	Monaco	AD	1990	395.07	424.06	365.69
ASDR	Monaco	AD	1990	147.85	247.79	75.87

ASPR	Mongolia	AD	2021	4396.36	4638.41	4155.80
ASIR	Mongolia	AD	2021	414.54	447.45	382.61
ASDR	Mongolia	AD	2021	192.80	321.28	98.97
ASPR	Mongolia	AD	1990	4392.96	4634.58	4152.23
ASIR	Mongolia	AD	1990	414.80	447.81	382.80
ASDR	Mongolia	AD	1990	192.19	325.33	99.84
ASPR	Montenegro	AD	2021	1799.52	1898.30	1700.47
ASIR	Montenegro	AD	2021	231.78	249.21	216.15
ASDR	Montenegro	AD	2021	79.51	134.20	40.88
ASPR	Montenegro	AD	1990	1802.82	1902.07	1703.74
ASIR	Montenegro	AD	1990	232.22	249.71	216.53
ASDR	Montenegro	AD	1990	79.62	135.16	41.07
ASPR	Morocco	AD	2021	1351.27	1426.44	1280.67
ASIR	Morocco	AD	2021	184.23	196.56	172.01
ASDR	Morocco	AD	2021	59.30	101.15	30.44
ASPR	Morocco	AD	1990	1353.09	1428.38	1282.43
ASIR	Morocco	AD	1990	184.40	196.73	172.17
ASDR	Morocco	AD	1990	59.40	99.47	30.67
ASPR	Mozambique	AD	2021	987.98	1034.28	943.42
ASIR	Mozambique	AD	2021	143.62	152.19	134.01
ASDR	Mozambique	AD	2021	42.87	72.88	22.07
ASPR	Mozambique	AD	1990	988.02	1034.25	943.45
ASIR	Mozambique	AD	1990	143.59	152.19	134.00
ASDR	Mozambique	AD	1990	42.75	73.03	22.19
ASPR	Myanmar	AD	2021	1647.78	1734.18	1560.08
ASIR	Myanmar	AD	2021	220.22	236.29	205.54
ASDR	Myanmar	AD	2021	72.11	122.33	37.34
ASPR	Myanmar	AD	1990	1647.41	1733.91	1559.20
ASIR	Myanmar	AD	1990	220.26	236.31	205.56
ASDR	Myanmar	AD	1990	71.70	122.56	37.17
ASPR	Namibia	AD	2021	1038.07	1103.14	980.01
ASIR	Namibia	AD	2021	151.71	164.74	140.09
ASDR	Namibia	AD	2021	45.57	78.15	23.10
ASPR	Namibia	AD	1990	1037.84	1102.89	979.77
ASIR	Namibia	AD	1990	151.64	164.66	140.04
ASDR	Namibia	AD	1990	45.43	76.42	23.28
ASPR	Nauru	AD	2021	1729.27	1837.67	1635.68
ASIR	Nauru	AD	2021	236.39	255.09	218.18
ASDR	Nauru	AD	2021	75.81	128.60	39.00
ASPR	Nauru	AD	1990	1727.14	1835.33	1634.49
ASIR	Nauru	AD	1990	236.16	254.84	217.91
ASDR	Nauru	AD	1990	75.78	127.84	39.04
ASPR	Nepal	AD	2021	1467.36	1554.21	1382.01
ASIR	Nepal	AD	2021	205.54	222.40	190.58

ASDR	Nepal	AD	2021	64.61	109.28	33.42
ASPR	Nepal	AD	1990	1465.56	1552.67	1380.32
ASIR	Nepal	AD	1990	205.35	222.26	190.36
ASDR	Nepal	AD	1990	63.90	109.12	33.07
ASPR	Netherlands	AD	2021	3376.06	3527.70	3214.62
ASIR	Netherlands	AD	2021	395.50	424.45	366.21
ASDR	Netherlands	AD	2021	147.96	250.69	76.15
ASPR	Netherlands	AD	1990	3376.75	3528.60	3215.60
ASIR	Netherlands	AD	1990	395.57	424.47	366.28
ASDR	Netherlands	AD	1990	148.10	248.70	76.25
ASPR	New Zealand	AD	2021	3379.68	3531.55	3240.97
ASIR	New Zealand	AD	2021	414.66	438.66	390.18
ASDR	New Zealand	AD	2021	147.58	245.99	75.76
ASPR	New Zealand	AD	1990	3354.94	3505.59	3202.35
ASIR	New Zealand	AD	1990	412.80	439.74	388.77
ASDR	New Zealand	AD	1990	146.26	244.41	74.81
ASPR	Nicaragua	AD	2021	1877.97	1985.09	1772.31
ASIR	Nicaragua	AD	2021	258.37	278.03	239.95
ASDR	Nicaragua	AD	2021	82.20	136.42	41.75
ASPR	Nicaragua	AD	1990	1881.82	1989.34	1776.39
ASIR	Nicaragua	AD	1990	258.64	278.37	240.16
ASDR	Nicaragua	AD	1990	81.97	135.68	41.93
ASPR	Niger	AD	2021	1041.70	1088.83	995.37
ASIR	Niger	AD	2021	145.57	153.72	137.17
ASDR	Niger	AD	2021	45.57	77.95	23.50
ASPR	Niger	AD	1990	1041.75	1088.94	995.32
ASIR	Niger	AD	1990	145.48	153.70	137.07
ASDR	Niger	AD	1990	45.22	76.42	23.20
ASPR	Nigeria	AD	2021	1054.41	1101.76	1008.71
ASIR	Nigeria	AD	2021	147.98	155.41	140.97
ASDR	Nigeria	AD	2021	45.97	77.36	23.78
ASPR	Nigeria	AD	1990	1052.71	1100.04	1006.88
ASIR	Nigeria	AD	1990	147.76	155.18	140.85
ASDR	Nigeria	AD	1990	45.65	77.42	23.58
ASPR	Niue	AD	2021	1726.64	1834.36	1633.31
ASIR	Niue	AD	2021	236.26	254.98	218.02
ASDR	Niue	AD	2021	75.82	126.19	39.06
ASPR	Niue	AD	1990	1725.84	1833.57	1632.19
ASIR	Niue	AD	1990	236.21	254.93	217.99
ASDR	Niue	AD	1990	75.93	129.67	39.51
ASPR	North Macedonia	AD	2021	1797.86	1896.64	1698.84
ASIR	North Macedonia	AD	2021	231.60	249.08	215.95

ASDR	North Macedonia	AD	2021	79.32	133.76	41.06
ASPR	North Macedonia	AD	1990	1801.95	1901.09	1702.89
ASIR	North Macedonia	AD	1990	232.08	249.57	216.43
ASDR	North Macedonia	AD	1990	79.50	133.30	41.15
ASPR	Northern Mariana Islands	AD	2021	1723.36	1831.44	1630.90
ASIR	Northern Mariana Islands	AD	2021	235.95	254.64	217.75
ASDR	Northern Mariana Islands	AD	2021	75.82	128.72	38.46
ASPR	Northern Mariana Islands	AD	1990	1732.50	1839.53	1639.91
ASIR	Northern Mariana Islands	AD	1990	236.85	255.54	218.51
ASDR	Northern Mariana Islands	AD	1990	76.15	128.10	39.09
ASPR	Norway	AD	2021	3726.25	3877.65	3572.90
ASIR	Norway	AD	2021	427.69	452.01	404.22
ASDR	Norway	AD	2021	163.18	274.92	83.38
ASPR	Norway	AD	1990	3706.93	3870.42	3547.47
ASIR	Norway	AD	1990	426.50	449.97	403.14
ASDR	Norway	AD	1990	162.11	270.99	82.79
ASPR	Oman	AD	2021	1313.35	1419.24	1216.42
ASIR	Oman	AD	2021	180.94	195.19	166.44
ASDR	Oman	AD	2021	57.77	98.54	29.18
ASPR	Oman	AD	1990	1317.48	1420.79	1219.43
ASIR	Oman	AD	1990	181.64	196.44	166.45
ASDR	Oman	AD	1990	57.77	94.43	29.81
ASPR	Pakistan	AD	2021	1465.06	1538.36	1397.24
ASIR	Pakistan	AD	2021	207.40	220.85	195.95
ASDR	Pakistan	AD	2021	64.04	107.87	32.98
ASPR	Pakistan	AD	1990	1463.58	1536.85	1395.70
ASIR	Pakistan	AD	1990	207.25	220.68	195.81
ASDR	Pakistan	AD	1990	63.75	105.52	32.70

ASPR	Palau	AD	2021	1713.30	1820.70	1620.45
ASIR	Palau	AD	2021	235.36	253.92	217.30
ASDR	Palau	AD	2021	75.32	127.07	38.36
ASPR	Palau	AD	1990	1725.82	1833.92	1632.47
ASIR	Palau	AD	1990	236.24	254.93	218.04
ASDR	Palau	AD	1990	75.81	125.89	39.00
ASPR	Palestine	AD	2021	1402.70	1509.92	1298.03
ASIR	Palestine	AD	2021	188.91	203.46	173.86
ASDR	Palestine	AD	2021	61.60	104.32	32.17
ASPR	Palestine	AD	1990	1425.32	1534.83	1313.02
ASIR	Palestine	AD	1990	191.39	206.58	175.17
ASDR	Palestine	AD	1990	62.60	105.55	32.27
ASPR	Panama	AD	2021	1875.91	1983.03	1770.57
ASIR	Panama	AD	2021	258.15	277.83	239.77
ASDR	Panama	AD	2021	82.10	137.45	42.10
ASPR	Panama	AD	1990	1876.74	1983.98	1771.53
ASIR	Panama	AD	1990	258.22	277.95	239.83
ASDR	Panama	AD	1990	82.13	138.66	42.08
ASPR	Papua New Guinea	AD	2021	1724.46	1832.48	1631.60
ASIR	Papua New Guinea	AD	2021	235.87	254.54	217.67
ASDR	Papua New Guinea	AD	2021	75.41	127.55	39.01
ASPR	Papua New Guinea	AD	1990	1724.75	1832.88	1631.95
ASIR	Papua New Guinea	AD	1990	235.95	254.64	217.76
ASDR	Papua New Guinea	AD	1990	75.11	126.96	38.72
ASPR	Paraguay	AD	2021	2411.58	2516.95	2307.74
ASIR	Paraguay	AD	2021	299.79	319.58	279.28
ASDR	Paraguay	AD	2021	104.76	175.42	54.63
ASPR	Paraguay	AD	1990	2413.71	2519.19	2309.56
ASIR	Paraguay	AD	1990	299.95	319.76	279.41
ASDR	Paraguay	AD	1990	104.80	173.95	54.03
ASPR	Peru	AD	2021	1671.32	1750.40	1599.22
ASIR	Peru	AD	2021	224.82	237.72	212.08
ASDR	Peru	AD	2021	73.02	123.51	38.19
ASPR	Peru	AD	1990	1675.55	1754.59	1602.80
ASIR	Peru	AD	1990	225.17	238.01	212.46
ASDR	Peru	AD	1990	72.92	122.51	37.16
ASPR	Philippines	AD	2021	1647.65	1724.88	1578.72
ASIR	Philippines	AD	2021	222.89	234.42	210.92

ASDR	Philippines	AD	2021	72.17	120.69	37.32
ASPR	Philippines	AD	1990	1652.34	1730.13	1583.11
ASIR	Philippines	AD	1990	223.25	234.76	211.26
ASDR	Philippines	AD	1990	72.14	120.74	37.59
ASPR	Poland	AD	2021	1821.52	1910.88	1744.32
ASIR	Poland	AD	2021	236.73	251.32	223.18
ASDR	Poland	AD	2021	80.40	133.63	41.88
ASPR	Poland	AD	1990	1824.40	1914.03	1747.21
ASIR	Poland	AD	1990	237.10	251.68	223.56
ASDR	Poland	AD	1990	80.48	134.91	41.85
ASPR	Portugal	AD	2021	3380.80	3532.88	3219.58
ASIR	Portugal	AD	2021	395.80	424.76	366.46
ASDR	Portugal	AD	2021	148.07	248.88	76.46
ASPR	Portugal	AD	1990	3381.49	3533.78	3220.39
ASIR	Portugal	AD	1990	395.73	424.69	366.39
ASDR	Portugal	AD	1990	147.72	242.99	75.87
ASPR	Puerto Rico	AD	2021	1946.08	2047.60	1841.11
ASIR	Puerto Rico	AD	2021	263.11	283.37	244.02
ASDR	Puerto Rico	AD	2021	84.81	142.14	44.24
ASPR	Puerto Rico	AD	1990	1947.08	2048.64	1841.88
ASIR	Puerto Rico	AD	1990	263.17	283.44	244.11
ASDR	Puerto Rico	AD	1990	84.99	144.73	43.87
ASPR	Qatar	AD	2021	1336.26	1411.29	1265.63
ASIR	Qatar	AD	2021	182.86	195.32	170.69
ASDR	Qatar	AD	2021	58.86	101.31	30.09
ASPR	Qatar	AD	1990	1334.96	1410.04	1264.35
ASIR	Qatar	AD	1990	182.72	195.16	170.57
ASDR	Qatar	AD	1990	58.65	98.20	29.93
ASPR	Republic of Korea	AD	2021	4061.06	4290.60	3835.24
ASIR	Republic of Korea	AD	2021	435.35	468.57	403.91
ASDR	Republic of Korea	AD	2021	178.59	302.14	92.05
ASPR	Republic of Korea	AD	1990	4060.92	4297.59	3837.26
ASIR	Republic of Korea	AD	1990	434.92	468.11	402.59
ASDR	Republic of Korea	AD	1990	178.31	298.25	91.52
ASPR	Republic of Moldova	AD	2021	1934.42	2011.69	1858.50
ASIR	Republic of Moldova	AD	2021	181.16	192.62	170.18

ASDR	Republic of Moldova	AD	2021	85.11	140.23	43.62
ASPR	Republic of Moldova	AD	1990	1944.98	2022.76	1868.77
ASIR	Republic of Moldova	AD	1990	182.27	193.77	171.22
ASDR	Republic of Moldova	AD	1990	85.54	141.17	43.60
ASPR	Romania	AD	2021	1218.73	1286.70	1151.60
ASIR	Romania	AD	2021	168.13	180.02	156.44
ASDR	Romania	AD	2021	53.93	91.67	27.50
ASPR	Romania	AD	1990	1202.21	1266.57	1133.82
ASIR	Romania	AD	1990	165.40	177.26	153.77
ASDR	Romania	AD	1990	53.14	91.46	26.96
ASPR	Russian Federation	AD	2021	3385.44	3504.09	3277.12
ASIR	Russian Federation	AD	2021	236.20	249.38	224.27
ASDR	Russian Federation	AD	2021	148.92	247.16	75.96
ASPR	Russian Federation	AD	1990	3289.36	3416.85	3180.08
ASIR	Russian Federation	AD	1990	224.32	237.09	212.94
ASDR	Russian Federation	AD	1990	144.47	240.17	74.12
ASPR	Rwanda	AD	2021	687.58	733.76	646.68
ASIR	Rwanda	AD	2021	110.60	118.35	102.95
ASDR	Rwanda	AD	2021	30.12	51.39	15.45
ASPR	Rwanda	AD	1990	687.97	734.25	646.90
ASIR	Rwanda	AD	1990	110.65	118.41	102.98
ASDR	Rwanda	AD	1990	30.00	50.25	15.67
ASPR	Saint Kitts and Nevis	AD	2021	1946.40	2047.81	1841.59
ASIR	Saint Kitts and Nevis	AD	2021	263.17	283.38	244.10
ASDR	Saint Kitts and Nevis	AD	2021	84.87	145.18	44.21
ASPR	Saint Kitts and Nevis	AD	1990	1947.80	2049.50	1842.86
ASIR	Saint Kitts and Nevis	AD	1990	263.32	283.58	244.24
ASDR	Saint Kitts and Nevis	AD	1990	84.80	144.15	43.97

ASPR	Saint Lucia	AD	2021	1943.58	2044.85	1838.83
ASIR	Saint Lucia	AD	2021	262.93	283.14	243.81
ASDR	Saint Lucia	AD	2021	84.65	143.92	43.88
ASPR	Saint Lucia	AD	1990	1949.86	2051.72	1844.74
ASIR	Saint Lucia	AD	1990	263.49	283.73	244.45
ASDR	Saint Lucia	AD	1990	84.85	141.16	43.82
ASPR	Saint Vincent and the Grenadines	AD	2021	1943.22	2044.39	1838.35
ASIR	Saint Vincent and the Grenadines	AD	2021	262.91	283.09	243.82
ASDR	Saint Vincent and the Grenadines	AD	2021	84.61	141.37	43.74
ASPR	Saint Vincent and the Grenadines	AD	1990	1946.52	2048.15	1841.78
ASIR	Saint Vincent and the Grenadines	AD	1990	263.24	283.47	244.13
ASDR	Saint Vincent and the Grenadines	AD	1990	84.84	141.05	43.88
ASPR	Samoa	AD	2021	1726.91	1835.27	1633.82
ASIR	Samoa	AD	2021	236.22	254.91	218.01
ASDR	Samoa	AD	2021	75.88	127.17	39.53
ASPR	Samoa	AD	1990	1721.44	1829.38	1628.55
ASIR	Samoa	AD	1990	235.80	254.49	217.67
ASDR	Samoa	AD	1990	75.61	127.26	38.26
ASPR	San Marino	AD	2021	3376.04	3528.18	3215.22
ASIR	San Marino	AD	2021	395.17	424.22	365.79
ASDR	San Marino	AD	2021	147.92	247.16	77.25
ASPR	San Marino	AD	1990	3380.90	3532.98	3219.69
ASIR	San Marino	AD	1990	395.75	424.71	366.42
ASDR	San Marino	AD	1990	148.33	251.20	76.29
ASPR	Sao Tome and Principe	AD	2021	1041.03	1088.14	994.76
ASIR	Sao Tome and Principe	AD	2021	145.48	153.67	137.08
ASDR	Sao Tome and Principe	AD	2021	45.69	76.89	23.63
ASPR	Sao Tome and Principe	AD	1990	1042.03	1089.29	995.73

ASIR	Sao Tome and Principe	AD	1990	145.61	153.82	137.20
ASDR	Sao Tome and Principe	AD	1990	45.67	78.02	23.48
ASPR	Saudi Arabia	AD	2021	1341.92	1417.13	1271.04
ASIR	Saudi Arabia	AD	2021	183.36	195.69	171.14
ASDR	Saudi Arabia	AD	2021	58.98	99.50	30.39
ASPR	Saudi Arabia	AD	1990	1344.48	1419.67	1273.47
ASIR	Saudi Arabia	AD	1990	183.66	196.07	171.44
ASDR	Saudi Arabia	AD	1990	59.02	99.48	29.89
ASPR	Senegal	AD	2021	1040.08	1087.25	994.06
ASIR	Senegal	AD	2021	145.43	153.60	137.02
ASDR	Senegal	AD	2021	45.37	77.59	23.76
ASPR	Senegal	AD	1990	1043.34	1090.66	996.87
ASIR	Senegal	AD	1990	145.71	153.92	137.28
ASDR	Senegal	AD	1990	45.26	77.24	23.15
ASPR	Serbia	AD	2021	1794.61	1892.65	1695.50
ASIR	Serbia	AD	2021	231.29	248.74	215.71
ASDR	Serbia	AD	2021	79.47	131.84	40.48
ASPR	Serbia	AD	1990	1812.90	1913.13	1712.22
ASIR	Serbia	AD	1990	234.80	252.37	218.84
ASDR	Serbia	AD	1990	80.17	137.19	41.29
ASPR	Seychelles	AD	2021	1625.86	1710.72	1540.01
ASIR	Seychelles	AD	2021	218.99	234.78	204.34
ASDR	Seychelles	AD	2021	71.41	122.97	37.11
ASPR	Seychelles	AD	1990	1640.95	1727.15	1553.74
ASIR	Seychelles	AD	1990	219.89	235.85	205.21
ASDR	Seychelles	AD	1990	71.92	121.83	36.44
ASPR	Sierra Leone	AD	2021	1041.70	1088.95	995.34
ASIR	Sierra Leone	AD	2021	145.55	153.74	137.13
ASDR	Sierra Leone	AD	2021	45.41	77.05	23.44
ASPR	Sierra Leone	AD	1990	1042.06	1089.27	995.70
ASIR	Sierra Leone	AD	1990	145.56	153.75	137.13
ASDR	Sierra Leone	AD	1990	45.21	76.56	23.08
ASPR	Singapore	AD	2021	3253.32	3499.02	3009.67
ASIR	Singapore	AD	2021	389.04	420.79	358.98
ASDR	Singapore	AD	2021	143.23	240.14	73.22
ASPR	Singapore	AD	1990	3282.37	3527.82	3030.97
ASIR	Singapore	AD	1990	391.09	422.10	358.06
ASDR	Singapore	AD	1990	144.51	243.18	75.43
ASPR	Slovakia	AD	2021	1801.94	1901.19	1702.90
ASIR	Slovakia	AD	2021	232.12	249.65	216.45
ASDR	Slovakia	AD	2021	79.55	133.56	40.64
ASPR	Slovakia	AD	1990	1805.92	1905.53	1706.77

ASIR	Slovakia	AD	1990	232.63	250.16	216.94
ASDR	Slovakia	AD	1990	79.79	135.40	41.88
ASPR	Slovenia	AD	2021	1798.57	1897.47	1699.54
ASIR	Slovenia	AD	2021	231.70	249.22	216.04
ASDR	Slovenia	AD	2021	79.45	134.17	40.79
ASPR	Slovenia	AD	1990	1804.69	1904.07	1705.62
ASIR	Slovenia	AD	1990	232.37	249.93	216.71
ASDR	Slovenia	AD	1990	79.65	134.89	40.61
ASPR	Solomon Islands	AD	2021	1727.94	1836.09	1634.82
ASIR	Solomon Islands	AD	2021	236.20	254.88	217.98
ASDR	Solomon Islands	AD	2021	75.73	127.99	38.81
ASPR	Solomon Islands	AD	1990	1725.94	1834.01	1633.35
ASIR	Solomon Islands	AD	1990	236.01	254.70	217.78
ASDR	Solomon Islands	AD	1990	75.59	125.59	38.75
ASPR	Somalia	AD	2021	984.19	1030.19	940.39
ASIR	Somalia	AD	2021	143.24	151.73	133.66
ASDR	Somalia	AD	2021	42.92	73.00	22.31
ASPR	Somalia	AD	1990	981.63	1027.47	938.07
ASIR	Somalia	AD	1990	142.96	151.46	133.42
ASDR	Somalia	AD	1990	42.68	71.99	21.84
ASPR	South Africa	AD	2021	1041.09	1087.55	996.08
ASIR	South Africa	AD	2021	153.33	161.65	144.93
ASDR	South Africa	AD	2021	45.51	77.11	23.57
ASPR	South Africa	AD	1990	1042.60	1089.55	997.44
ASIR	South Africa	AD	1990	153.47	161.78	145.05
ASDR	South Africa	AD	1990	45.54	77.10	23.78
ASPR	South Sudan	AD	2021	983.92	1029.81	939.87
ASIR	South Sudan	AD	2021	143.14	151.71	133.62
ASDR	South Sudan	AD	2021	42.80	73.57	22.27
ASPR	South Sudan	AD	1990	980.58	1026.67	936.64
ASIR	South Sudan	AD	1990	142.74	151.27	133.23
ASDR	South Sudan	AD	1990	42.55	72.92	22.41
ASPR	Spain	AD	2021	3180.95	3475.46	2907.72
ASIR	Spain	AD	2021	384.75	419.57	351.57
ASDR	Spain	AD	2021	139.12	231.86	71.96
ASPR	Spain	AD	1990	3182.02	3476.47	2908.75
ASIR	Spain	AD	1990	384.80	419.61	351.63
ASDR	Spain	AD	1990	139.52	230.22	71.26

ASPR	Sri Lanka	AD	2021	1653.80	1751.12	1557.94
ASIR	Sri Lanka	AD	2021	220.59	235.90	205.43
ASDR	Sri Lanka	AD	2021	72.52	122.81	37.33
ASPR	Sri Lanka	AD	1990	1668.59	1768.24	1569.06
ASIR	Sri Lanka	AD	1990	221.38	236.75	205.72
ASDR	Sri Lanka	AD	1990	73.02	121.41	37.50
ASPR	Sudan	AD	2021	1350.08	1425.33	1279.31
ASIR	Sudan	AD	2021	184.07	196.33	171.86
ASDR	Sudan	AD	2021	59.03	99.29	30.37
ASPR	Sudan	AD	1990	1350.74	1426.05	1279.89
ASIR	Sudan	AD	1990	183.99	196.28	171.75
ASDR	Sudan	AD	1990	58.99	98.77	30.74
ASPR	Suriname	AD	2021	1944.65	2045.96	1839.58
ASIR	Suriname	AD	2021	263.05	283.30	243.95
ASDR	Suriname	AD	2021	84.45	140.10	44.16
ASPR	Suriname	AD	1990	1946.07	2047.68	1841.15
ASIR	Suriname	AD	1990	263.32	283.57	244.25
ASDR	Suriname	AD	1990	84.75	141.42	44.15
ASPR	Sweden	AD	2021	3390.25	3547.55	3238.75
ASIR	Sweden	AD	2021	403.91	427.76	382.77
ASDR	Sweden	AD	2021	148.64	245.45	75.65
ASPR	Sweden	AD	1990	3393.68	3551.41	3242.30
ASIR	Sweden	AD	1990	404.07	427.92	382.93
ASDR	Sweden	AD	1990	148.70	248.04	75.87
ASPR	Switzerland	AD	2021	3372.74	3524.22	3211.38
ASIR	Switzerland	AD	2021	395.30	424.24	366.03
ASDR	Switzerland	AD	2021	147.66	248.20	76.39
ASPR	Switzerland	AD	1990	3374.84	3526.54	3213.73
ASIR	Switzerland	AD	1990	395.50	424.41	366.22
ASDR	Switzerland	AD	1990	147.68	248.20	77.36
ASPR	Syrian Arab Republic	AD	2021	1355.96	1431.04	1284.37
ASIR	Syrian Arab Republic	AD	2021	184.55	196.84	172.34
ASDR	Syrian Arab Republic	AD	2021	59.35	99.20	30.48
ASPR	Syrian Arab Republic	AD	1990	1350.54	1425.74	1279.91
ASIR	Syrian Arab Republic	AD	1990	184.14	196.45	171.91
ASDR	Syrian Arab Republic	AD	1990	59.18	100.44	30.66
ASPR	Taiwan (Province of	AD	2021	2369.65	2534.99	2202.89

	China)					
ASIR	Taiwan (Province of China)	AD	2021	284.82	307.04	263.57
ASDR	Taiwan (Province of China)	AD	2021	104.06	174.32	53.35
ASPR	Taiwan (Province of China)	AD	1990	2322.88	2477.47	2165.85
ASIR	Taiwan (Province of China)	AD	1990	281.25	302.51	260.15
ASDR	Taiwan (Province of China)	AD	1990	101.89	169.40	52.55
ASPR	Tajikistan	AD	2021	4381.31	4622.99	4141.44
ASIR	Tajikistan	AD	2021	413.73	446.59	381.88
ASDR	Tajikistan	AD	2021	192.19	327.69	99.57
ASPR	Tajikistan	AD	1990	4395.93	4637.51	4154.86
ASIR	Tajikistan	AD	1990	414.72	447.67	382.80
ASDR	Tajikistan	AD	1990	192.39	328.16	98.81
ASPR	Thailand	AD	2021	1643.94	1730.01	1556.80
ASIR	Thailand	AD	2021	219.76	235.79	205.09
ASDR	Thailand	AD	2021	72.27	124.17	37.36
ASPR	Thailand	AD	1990	1643.16	1729.14	1555.73
ASIR	Thailand	AD	1990	219.87	235.89	205.20
ASDR	Thailand	AD	1990	72.11	124.42	37.60
ASPR	Timor-Leste	AD	2021	1639.63	1725.48	1552.78
ASIR	Timor-Leste	AD	2021	219.28	235.26	204.63
ASDR	Timor-Leste	AD	2021	71.80	121.30	37.67
ASPR	Timor-Leste	AD	1990	1630.10	1715.12	1544.77
ASIR	Timor-Leste	AD	1990	218.72	234.55	204.06
ASDR	Timor-Leste	AD	1990	71.00	121.57	36.44
ASPR	Togo	AD	2021	1004.89	1079.36	937.60
ASIR	Togo	AD	2021	142.44	152.57	132.81
ASDR	Togo	AD	2021	43.82	75.52	22.73
ASPR	Togo	AD	1990	1005.73	1080.29	938.30
ASIR	Togo	AD	1990	142.48	152.65	132.86
ASDR	Togo	AD	1990	43.69	72.60	21.98
ASPR	Tokelau	AD	2021	1725.23	1833.28	1632.01
ASIR	Tokelau	AD	2021	235.99	254.70	217.80
ASDR	Tokelau	AD	2021	75.66	127.01	38.34

ASPR	Tokelau	AD	1990	1729.28	1837.26	1635.62
ASIR	Tokelau	AD	1990	236.39	255.05	218.11
ASDR	Tokelau	AD	1990	75.96	128.04	39.25
ASPR	Tonga	AD	2021	1731.28	1839.42	1637.93
ASIR	Tonga	AD	2021	236.42	255.08	218.17
ASDR	Tonga	AD	2021	76.09	127.45	38.58
ASPR	Tonga	AD	1990	1729.85	1837.98	1636.39
ASIR	Tonga	AD	1990	236.39	255.04	218.14
ASDR	Tonga	AD	1990	75.95	131.18	38.90
ASPR	Trinidad and Tobago	AD	2021	1944.18	2045.46	1839.39
ASIR	Trinidad and Tobago	AD	2021	262.96	283.16	243.85
ASDR	Trinidad and Tobago	AD	2021	84.57	139.12	43.73
ASPR	Trinidad and Tobago	AD	1990	1945.93	2047.38	1841.00
ASIR	Trinidad and Tobago	AD	1990	263.17	283.40	244.09
ASDR	Trinidad and Tobago	AD	1990	84.75	140.93	43.69
ASPR	Tunisia	AD	2021	1350.47	1425.63	1279.80
ASIR	Tunisia	AD	2021	184.11	196.39	171.88
ASDR	Tunisia	AD	2021	59.29	100.71	30.34
ASPR	Tunisia	AD	1990	1351.23	1426.46	1280.62
ASIR	Tunisia	AD	1990	184.20	196.51	171.98
ASDR	Tunisia	AD	1990	59.32	102.92	30.35
ASPR	Turkey	AD	2021	2161.66	2302.64	2016.31
ASIR	Turkey	AD	2021	248.02	268.80	227.29
ASDR	Turkey	AD	2021	94.69	157.57	48.63
ASPR	Turkey	AD	1990	2152.35	2297.34	2024.09
ASIR	Turkey	AD	1990	247.30	267.31	228.30
ASDR	Turkey	AD	1990	94.03	158.19	48.75
ASPR	Turkmenistan	AD	2021	4370.87	4612.49	4131.12
ASIR	Turkmenistan	AD	2021	413.27	446.10	381.36
ASDR	Turkmenistan	AD	2021	191.70	322.39	98.94
ASPR	Turkmenistan	AD	1990	4401.52	4643.64	4160.72
ASIR	Turkmenistan	AD	1990	415.11	448.12	383.14
ASDR	Turkmenistan	AD	1990	192.49	327.64	99.33
ASPR	Tuvalu	AD	2021	1721.96	1830.02	1629.11
ASIR	Tuvalu	AD	2021	235.81	254.52	217.68
ASDR	Tuvalu	AD	2021	75.64	126.12	38.42
ASPR	Tuvalu	AD	1990	1735.36	1843.63	1641.93
ASIR	Tuvalu	AD	1990	236.61	255.24	218.32

ASDR	Tuvalu	AD	1990	76.17	127.17	38.78
ASPR	Uganda	AD	2021	1288.47	1382.37	1199.69
ASIR	Uganda	AD	2021	171.75	185.15	158.73
ASDR	Uganda	AD	2021	56.27	94.91	29.12
ASPR	Uganda	AD	1990	1288.84	1383.40	1200.13
ASIR	Uganda	AD	1990	171.75	185.15	158.69
ASDR	Uganda	AD	1990	55.80	96.32	28.53
ASPR	Ukraine	AD	2021	1965.29	2045.57	1890.17
ASIR	Ukraine	AD	2021	189.57	201.58	178.77
ASDR	Ukraine	AD	2021	86.53	144.19	43.92
ASPR	Ukraine	AD	1990	1973.01	2053.40	1897.56
ASIR	Ukraine	AD	1990	190.39	202.41	179.55
ASDR	Ukraine	AD	1990	86.79	146.08	44.20
ASPR	United Arab Emirates	AD	2021	1338.05	1413.36	1266.56
ASIR	United Arab Emirates	AD	2021	182.74	195.22	170.43
ASDR	United Arab Emirates	AD	2021	58.83	100.09	29.86
ASPR	United Arab Emirates	AD	1990	1337.36	1412.65	1266.54
ASIR	United Arab Emirates	AD	1990	182.89	195.35	170.69
ASDR	United Arab Emirates	AD	1990	58.71	97.57	29.88
ASPR	United Kingdom	AD	2021	3807.40	3961.73	3651.09
ASIR	United Kingdom	AD	2021	430.30	455.45	406.36
ASDR	United Kingdom	AD	2021	166.44	279.55	85.62
ASPR	United Kingdom	AD	1990	3877.12	4039.82	3711.26
ASIR	United Kingdom	AD	1990	435.62	458.99	412.31
ASDR	United Kingdom	AD	1990	169.57	281.31	87.09
ASPR	United Republic of Tanzania	AD	2021	1427.07	1496.89	1360.42
ASIR	United Republic of Tanzania	AD	2021	182.87	196.04	170.88

ASDR	United Republic of Tanzania	AD	2021	62.33	103.23	32.17
ASPR	United Republic of Tanzania	AD	1990	1440.45	1504.70	1375.06
ASIR	United Republic of Tanzania	AD	1990	183.85	196.01	172.23
ASDR	United Republic of Tanzania	AD	1990	62.02	102.68	32.46
ASPR	United States of America	AD	2021	3161.54	3272.83	3052.34
ASIR	United States of America	AD	2021	336.57	351.42	321.52
ASDR	United States of America	AD	2021	136.56	224.92	70.53
ASPR	United States of America	AD	1990	3169.60	3277.88	3064.98
ASIR	United States of America	AD	1990	336.56	351.78	321.57
ASDR	United States of America	AD	1990	137.61	227.96	70.98
ASPR	United States Virgin Islands	AD	2021	1943.39	2044.62	1838.55
ASIR	United States Virgin Islands	AD	2021	262.72	282.93	243.64
ASDR	United States Virgin Islands	AD	2021	84.70	141.28	43.66
ASPR	United States Virgin Islands	AD	1990	1948.91	2050.54	1843.55
ASIR	United States Virgin Islands	AD	1990	263.35	283.62	244.34
ASDR	United States Virgin Islands	AD	1990	85.07	141.07	43.68
ASPR	Uruguay	AD	2021	3211.17	3394.03	3031.86
ASIR	Uruguay	AD	2021	387.74	417.26	357.70
ASDR	Uruguay	AD	2021	140.51	237.05	72.56
ASPR	Uruguay	AD	1990	3212.51	3395.66	3032.93
ASIR	Uruguay	AD	1990	387.94	417.48	357.80
ASDR	Uruguay	AD	1990	140.67	235.54	71.44
ASPR	Uzbekistan	AD	2021	4386.45	4628.25	4146.27
ASIR	Uzbekistan	AD	2021	413.74	446.55	381.89

ASDR	Uzbekistan	AD	2021	192.15	326.03	98.23
ASPR	Uzbekistan	AD	1990	4400.42	4642.28	4159.32
ASIR	Uzbekistan	AD	1990	415.02	448.01	383.06
ASDR	Uzbekistan	AD	1990	192.31	324.35	99.31
ASPR	Vanuatu	AD	2021	1730.09	1838.23	1636.88
ASIR	Vanuatu	AD	2021	236.45	255.13	218.20
ASDR	Vanuatu	AD	2021	75.90	127.67	39.13
ASPR	Vanuatu	AD	1990	1728.03	1836.14	1635.35
ASIR	Vanuatu	AD	1990	236.16	254.83	217.93
ASDR	Vanuatu	AD	1990	75.72	126.07	38.71
ASPR	Venezuela (Bolivarian Republic of)	AD	2021	2352.60	2503.24	2201.32
ASIR	Venezuela (Bolivarian Republic of)	AD	2021	299.40	322.22	277.48
ASDR	Venezuela (Bolivarian Republic of)	AD	2021	102.76	174.81	53.36
ASPR	Venezuela (Bolivarian Republic of)	AD	1990	2356.45	2517.36	2214.48
ASIR	Venezuela (Bolivarian Republic of)	AD	1990	299.82	323.90	277.12
ASDR	Venezuela (Bolivarian Republic of)	AD	1990	102.97	172.86	53.27
ASPR	Viet Nam	AD	2021	1635.46	1721.01	1549.78
ASIR	Viet Nam	AD	2021	219.16	235.09	204.53
ASDR	Viet Nam	AD	2021	71.96	123.85	36.83
ASPR	Viet Nam	AD	1990	1650.13	1736.61	1562.43
ASIR	Viet Nam	AD	1990	220.31	236.38	205.62
ASDR	Viet Nam	AD	1990	72.11	124.51	37.14
ASPR	Yemen	AD	2021	1351.41	1426.61	1280.78
ASIR	Yemen	AD	2021	184.24	196.56	172.01
ASDR	Yemen	AD	2021	58.59	102.25	30.35
ASPR	Yemen	AD	1990	1350.45	1425.67	1279.66
ASIR	Yemen	AD	1990	184.20	196.51	171.99
ASDR	Yemen	AD	1990	58.54	100.55	30.34
ASPR	Zambia	AD	2021	986.14	1032.50	941.79
ASIR	Zambia	AD	2021	143.41	151.97	133.81
ASDR	Zambia	AD	2021	42.95	72.58	22.35
ASPR	Zambia	AD	1990	986.32	1032.60	941.82

ASIR	Zambia	AD	1990	143.42	152.01	133.83
ASDR	Zambia	AD	1990	42.90	72.43	21.88
ASPR	Zimbabwe	AD	2021	1038.55	1103.78	980.43
ASIR	Zimbabwe	AD	2021	151.76	164.81	140.13
ASDR	Zimbabwe	AD	2021	45.48	77.60	23.55
ASPR	Zimbabwe	AD	1990	1037.67	1102.66	979.65
ASIR	Zimbabwe	AD	1990	151.62	164.60	140.04
ASDR	Zimbabwe	AD	1990	45.48	77.45	23.20
ASPR	Afghanistan	CD	2021	982.15	1191.84	799.10
ASIR	Afghanistan	CD	2021	2719.36	3335.89	2207.87
ASDR	Afghanistan	CD	2021	23.68	36.07	14.39
ASPR	Afghanistan	CD	1990	982.34	1195.37	798.97
ASIR	Afghanistan	CD	1990	2718.85	3335.28	2212.04
ASDR	Afghanistan	CD	1990	23.86	36.49	14.61
ASPR	Albania	CD	2021	1269.37	1542.75	1041.91
ASIR	Albania	CD	2021	3448.94	4257.73	2810.59
ASDR	Albania	CD	2021	31.36	46.95	19.47
ASPR	Albania	CD	1990	1259.23	1533.64	1033.82
ASIR	Albania	CD	1990	3421.91	4224.01	2788.96
ASDR	Albania	CD	1990	31.16	47.52	19.32
ASPR	Algeria	CD	2021	978.10	1188.81	796.44
ASIR	Algeria	CD	2021	2707.46	3320.72	2199.44
ASDR	Algeria	CD	2021	24.01	36.23	14.71
ASPR	Algeria	CD	1990	978.99	1189.53	796.74
ASIR	Algeria	CD	1990	2710.06	3324.10	2201.56
ASDR	Algeria	CD	1990	24.13	36.43	14.71
ASPR	American Samoa	CD	2021	1176.17	1431.98	963.30
ASIR	American Samoa	CD	2021	3218.55	4012.08	2608.11
ASDR	American Samoa	CD	2021	28.85	43.93	18.13
ASPR	American Samoa	CD	1990	1175.72	1431.69	963.13
ASIR	American Samoa	CD	1990	3217.36	4010.14	2606.03
ASDR	American Samoa	CD	1990	29.08	44.36	18.27
ASPR	Andorra	CD	2021	453.30	553.33	364.34
ASIR	Andorra	CD	2021	1232.48	1535.49	999.24
ASDR	Andorra	CD	2021	11.25	17.06	6.92
ASPR	Andorra	CD	1990	451.08	550.49	362.76
ASIR	Andorra	CD	1990	1226.42	1527.41	993.98
ASDR	Andorra	CD	1990	11.27	17.04	7.11

ASPR	Angola	CD	2021	896.14	1089.38	719.94
ASIR	Angola	CD	2021	2483.58	3099.12	1997.19
ASDR	Angola	CD	2021	21.93	33.40	13.86
ASPR	Angola	CD	1990	890.96	1085.15	716.50
ASIR	Angola	CD	1990	2469.17	3081.22	1986.59
ASDR	Angola	CD	1990	21.72	32.94	13.56
ASPR	Antigua and Barbuda	CD	2021	1090.81	1335.69	887.81
ASIR	Antigua and Barbuda	CD	2021	3005.01	3734.74	2440.90
ASDR	Antigua and Barbuda	CD	2021	26.84	40.66	16.75
ASPR	Antigua and Barbuda	CD	1990	1093.07	1337.75	889.96
ASIR	Antigua and Barbuda	CD	1990	3011.27	3742.33	2446.14
ASDR	Antigua and Barbuda	CD	1990	26.99	41.18	16.63
ASPR	Argentina	CD	2021	273.90	332.26	221.44
ASIR	Argentina	CD	2021	769.76	943.93	629.58
ASDR	Argentina	CD	2021	6.78	10.38	4.11
ASPR	Argentina	CD	1990	273.95	332.31	221.49
ASIR	Argentina	CD	1990	769.89	943.99	629.74
ASDR	Argentina	CD	1990	6.80	10.37	4.24
ASPR	Armenia	CD	2021	1281.37	1558.18	1051.20
ASIR	Armenia	CD	2021	3481.77	4297.27	2832.45
ASDR	Armenia	CD	2021	31.71	48.16	19.08
ASPR	Armenia	CD	1990	1282.02	1559.93	1052.13
ASIR	Armenia	CD	1990	3483.16	4299.84	2833.98
ASDR	Armenia	CD	1990	31.73	48.09	19.70
ASPR	Australia	CD	2021	273.45	331.76	221.17
ASIR	Australia	CD	2021	768.53	942.61	628.71
ASDR	Australia	CD	2021	6.74	10.62	4.18
ASPR	Australia	CD	1990	272.91	331.25	220.62
ASIR	Australia	CD	1990	766.92	940.49	627.28
ASDR	Australia	CD	1990	6.73	10.16	4.21
ASPR	Austria	CD	2021	454.79	555.33	365.63
ASIR	Austria	CD	2021	1236.66	1540.00	1002.40
ASDR	Austria	CD	2021	11.31	17.03	6.85
ASPR	Austria	CD	1990	455.88	557.04	366.73
ASIR	Austria	CD	1990	1239.71	1543.89	1004.45
ASDR	Austria	CD	1990	11.33	16.96	6.64
ASPR	Azerbaijan	CD	2021	1272.73	1548.15	1044.36
ASIR	Azerbaijan	CD	2021	3458.26	4268.26	2815.30

ASDR	Azerbaijan	CD	2021	31.51	47.47	19.38
ASPR	Azerbaijan	CD	1990	1283.94	1562.96	1053.65
ASIR	Azerbaijan	CD	1990	3488.49	4306.51	2839.61
ASDR	Azerbaijan	CD	1990	31.84	48.25	19.34
ASPR	Bahamas	CD	2021	1092.50	1336.98	889.13
ASIR	Bahamas	CD	2021	3009.61	3740.88	2444.53
ASDR	Bahamas	CD	2021	26.89	40.59	16.61
ASPR	Bahamas	CD	1990	1092.95	1337.09	889.55
ASIR	Bahamas	CD	1990	3010.88	3742.27	2445.41
ASDR	Bahamas	CD	1990	27.05	41.62	16.48
ASPR	Bahrain	CD	2021	961.50	1168.82	784.24
ASIR	Bahrain	CD	2021	2661.28	3264.00	2160.25
ASDR	Bahrain	CD	2021	23.50	35.45	14.43
ASPR	Bahrain	CD	1990	967.68	1175.51	790.00
ASIR	Bahrain	CD	1990	2678.34	3284.34	2175.49
ASDR	Bahrain	CD	1990	23.85	36.03	14.80
ASPR	Bangladesh	CD	2021	954.62	1173.29	768.74
ASIR	Bangladesh	CD	2021	2639.60	3283.24	2131.69
ASDR	Bangladesh	CD	2021	23.44	35.56	14.23
ASPR	Bangladesh	CD	1990	948.97	1166.97	764.89
ASIR	Bangladesh	CD	1990	2623.24	3266.27	2116.80
ASDR	Bangladesh	CD	1990	23.29	35.42	14.07
ASPR	Barbados	CD	2021	1091.73	1336.25	888.37
ASIR	Barbados	CD	2021	3007.58	3737.81	2442.55
ASDR	Barbados	CD	2021	26.93	41.00	16.63
ASPR	Barbados	CD	1990	1093.71	1337.84	889.91
ASIR	Barbados	CD	1990	3013.14	3744.54	2447.02
ASDR	Barbados	CD	1990	27.05	41.18	16.64
ASPR	Belarus	CD	2021	1185.40	1433.79	965.84
ASIR	Belarus	CD	2021	3243.12	4004.86	2662.06
ASDR	Belarus	CD	2021	29.26	43.54	18.59
ASPR	Belarus	CD	1990	1192.05	1442.01	970.22
ASIR	Belarus	CD	1990	3261.33	4027.24	2676.51
ASDR	Belarus	CD	1990	29.50	44.40	18.70
ASPR	Belgium	CD	2021	455.25	556.14	365.91
ASIR	Belgium	CD	2021	1237.89	1542.37	1003.54
ASDR	Belgium	CD	2021	11.31	16.92	6.90
ASPR	Belgium	CD	1990	455.13	556.05	365.88
ASIR	Belgium	CD	1990	1237.60	1541.34	1003.13
ASDR	Belgium	CD	1990	11.34	17.11	7.02
ASPR	Belize	CD	2021	1088.66	1334.37	886.06
ASIR	Belize	CD	2021	2998.79	3728.20	2435.92
ASDR	Belize	CD	2021	26.87	41.36	16.31
ASPR	Belize	CD	1990	1087.18	1331.73	884.89

ASIR	Belize	CD	1990	2994.46	3722.76	2432.05
ASDR	Belize	CD	1990	26.97	41.12	16.35
ASPR	Benin	CD	2021	907.76	1102.18	739.22
ASIR	Benin	CD	2021	2498.77	3088.34	2033.71
ASDR	Benin	CD	2021	22.33	33.80	13.79
ASPR	Benin	CD	1990	908.78	1104.35	740.68
ASIR	Benin	CD	1990	2501.79	3092.80	2035.70
ASDR	Benin	CD	1990	22.23	33.73	13.67
ASPR	Bermuda	CD	2021	1090.83	1335.02	887.96
ASIR	Bermuda	CD	2021	3004.91	3735.05	2440.57
ASDR	Bermuda	CD	2021	27.08	41.51	16.96
ASPR	Bermuda	CD	1990	1092.49	1336.30	889.17
ASIR	Bermuda	CD	1990	3009.59	3740.54	2444.14
ASDR	Bermuda	CD	1990	27.11	41.23	16.70
ASPR	Bhutan	CD	2021	951.03	1169.22	767.06
ASIR	Bhutan	CD	2021	2629.41	3271.72	2121.28
ASDR	Bhutan	CD	2021	23.45	35.53	14.50
ASPR	Bhutan	CD	1990	951.28	1169.19	767.98
ASIR	Bhutan	CD	1990	2630.35	3271.16	2122.41
ASDR	Bhutan	CD	1990	23.44	34.96	14.38
ASPR	Bolivia (Plurinational State of)	CD	2021	1225.94	1496.23	994.27
ASIR	Bolivia (Plurinational State of)	CD	2021	3313.90	4098.25	2685.73
ASDR	Bolivia (Plurinational State of)	CD	2021	30.20	46.44	18.62
ASPR	Bolivia (Plurinational State of)	CD	1990	1227.43	1497.22	995.78
ASIR	Bolivia (Plurinational State of)	CD	1990	3317.88	4105.05	2688.99
ASDR	Bolivia (Plurinational State of)	CD	1990	30.29	47.27	18.75
ASPR	Bosnia and Herzegovina	CD	2021	1270.67	1545.75	1042.89
ASIR	Bosnia and Herzegovina	CD	2021	3452.13	4259.39	2812.47
ASDR	Bosnia and Herzegovina	CD	2021	31.27	47.28	19.16

ASPR	Bosnia and Herzegovina	CD	1990	1272.11	1547.39	1044.26
ASIR	Bosnia and Herzegovina	CD	1990	3455.50	4269.03	2814.34
ASDR	Bosnia and Herzegovina	CD	1990	31.41	47.49	19.89
ASPR	Botswana	CD	2021	895.16	1089.18	719.33
ASIR	Botswana	CD	2021	2481.01	3096.09	1994.49
ASDR	Botswana	CD	2021	21.77	33.07	13.41
ASPR	Botswana	CD	1990	896.85	1089.88	720.67
ASIR	Botswana	CD	1990	2485.49	3101.19	1999.26
ASDR	Botswana	CD	1990	22.06	33.19	13.83
ASPR	Brazil	CD	2021	1235.50	1523.60	990.50
ASIR	Brazil	CD	2021	3438.86	4330.96	2762.74
ASDR	Brazil	CD	2021	30.12	45.87	18.70
ASPR	Brazil	CD	1990	1234.54	1522.33	989.54
ASIR	Brazil	CD	1990	3435.97	4327.76	2761.27
ASDR	Brazil	CD	1990	30.10	45.66	18.63
ASPR	Brunei Darussalam	CD	2021	271.18	329.03	219.59
ASIR	Brunei Darussalam	CD	2021	761.95	934.38	624.15
ASDR	Brunei Darussalam	CD	2021	6.71	10.13	4.10
ASPR	Brunei Darussalam	CD	1990	270.17	327.95	218.78
ASIR	Brunei Darussalam	CD	1990	759.12	930.94	621.88
ASDR	Brunei Darussalam	CD	1990	6.70	10.24	4.19
ASPR	Bulgaria	CD	2021	1267.00	1541.68	1040.32
ASIR	Bulgaria	CD	2021	3442.14	4248.44	2804.67
ASDR	Bulgaria	CD	2021	31.26	48.06	19.29
ASPR	Bulgaria	CD	1990	1274.48	1550.11	1045.76
ASIR	Bulgaria	CD	1990	3462.68	4271.46	2820.48
ASDR	Bulgaria	CD	1990	31.42	47.80	19.86
ASPR	Burkina Faso	CD	2021	908.33	1103.28	740.16
ASIR	Burkina Faso	CD	2021	2500.40	3089.68	2034.56
ASDR	Burkina Faso	CD	2021	22.40	33.93	13.85
ASPR	Burkina Faso	CD	1990	909.25	1105.60	741.01
ASIR	Burkina Faso	CD	1990	2503.08	3094.58	2036.81
ASDR	Burkina Faso	CD	1990	22.29	33.19	13.83
ASPR	Burundi	CD	2021	889.72	1083.86	715.80
ASIR	Burundi	CD	2021	2465.56	3076.88	1984.89

ASDR	Burundi	CD	2021	21.90	33.53	13.56
ASPR	Burundi	CD	1990	895.41	1089.05	719.37
ASIR	Burundi	CD	1990	2481.54	3097.17	1995.06
ASDR	Burundi	CD	1990	22.04	33.13	13.47
ASPR	Côte d'Ivoire	CD	2021	901.80	1094.24	734.87
ASIR	Côte d'Ivoire	CD	2021	2482.27	3067.35	2017.75
ASDR	Côte d'Ivoire	CD	2021	22.18	33.40	13.72
ASPR	Côte d'Ivoire	CD	1990	901.20	1093.09	734.06
ASIR	Côte d'Ivoire	CD	1990	2480.33	3064.58	2017.41
ASDR	Côte d'Ivoire	CD	1990	21.93	33.72	13.59
ASPR	Cabo Verde	CD	2021	906.57	1099.13	737.82
ASIR	Cabo Verde	CD	2021	2495.51	3081.50	2027.99
ASDR	Cabo Verde	CD	2021	22.39	34.19	13.68
ASPR	Cabo Verde	CD	1990	913.37	1110.24	742.83
ASIR	Cabo Verde	CD	1990	2514.68	3111.78	2048.82
ASDR	Cabo Verde	CD	1990	22.59	34.50	14.06
ASPR	Cambodia	CD	2021	1374.68	1713.11	1114.21
ASIR	Cambodia	CD	2021	3686.34	4584.52	2995.92
ASDR	Cambodia	CD	2021	34.05	52.16	20.91
ASPR	Cambodia	CD	1990	1379.24	1720.70	1118.00
ASIR	Cambodia	CD	1990	3698.59	4602.69	3007.05
ASDR	Cambodia	CD	1990	34.00	51.34	20.97
ASPR	Cameroon	CD	2021	906.01	1099.88	738.06
ASIR	Cameroon	CD	2021	2493.97	3082.31	2029.10
ASDR	Cameroon	CD	2021	22.27	33.70	13.77
ASPR	Cameroon	CD	1990	906.70	1101.23	738.66
ASIR	Cameroon	CD	1990	2495.84	3084.02	2030.21
ASDR	Cameroon	CD	1990	22.17	33.74	13.81
ASPR	Canada	CD	2021	1126.46	1347.47	909.70
ASIR	Canada	CD	2021	2671.59	3337.34	2173.96
ASDR	Canada	CD	2021	27.93	42.25	17.33
ASPR	Canada	CD	1990	1126.43	1347.70	909.84
ASIR	Canada	CD	1990	2672.15	3337.18	2175.24
ASDR	Canada	CD	1990	27.95	41.69	17.75
ASPR	Central African Republic	CD	2021	895.85	1089.05	720.09
ASIR	Central African Republic	CD	2021	2482.84	3097.02	1997.25
ASDR	Central African Republic	CD	2021	21.82	33.48	13.88

ASPR	Central African Republic	CD	1990	895.05	1088.80	718.93
ASIR	Central African Republic	CD	1990	2480.56	3094.95	1994.51
ASDR	Central African Republic	CD	1990	21.74	32.91	13.64
ASPR	Chad	CD	2021	903.51	1097.63	736.42
ASIR	Chad	CD	2021	2486.85	3075.64	2022.19
ASDR	Chad	CD	2021	22.17	33.50	13.65
ASPR	Chad	CD	1990	908.31	1103.56	740.08
ASIR	Chad	CD	1990	2500.34	3092.62	2035.92
ASDR	Chad	CD	1990	22.23	33.61	13.77
ASPR	Chile	CD	2021	273.57	331.82	221.28
ASIR	Chile	CD	2021	768.81	942.82	629.02
ASDR	Chile	CD	2021	6.74	10.22	4.13
ASPR	Chile	CD	1990	274.28	332.65	221.75
ASIR	Chile	CD	1990	770.87	945.27	630.47
ASDR	Chile	CD	1990	6.76	10.17	4.19
ASPR	China	CD	2021	1322.99	1617.06	1061.03
ASIR	China	CD	2021	3672.53	4630.53	2957.15
ASDR	China	CD	2021	32.90	49.86	20.09
ASPR	China	CD	1990	1322.99	1617.03	1061.11
ASIR	China	CD	1990	3672.26	4624.82	2955.92
ASDR	China	CD	1990	32.86	49.86	20.18
ASPR	Colombia	CD	2021	1091.58	1335.81	888.01
ASIR	Colombia	CD	2021	3007.17	3737.42	2442.17
ASDR	Colombia	CD	2021	26.98	40.99	16.35
ASPR	Colombia	CD	1990	1090.16	1335.44	887.40
ASIR	Colombia	CD	1990	3002.95	3732.91	2439.15
ASDR	Colombia	CD	1990	26.92	40.57	16.60
ASPR	Comoros	CD	2021	893.18	1087.10	717.76
ASIR	Comoros	CD	2021	2475.49	3089.69	1989.93
ASDR	Comoros	CD	2021	21.97	33.67	13.44
ASPR	Comoros	CD	1990	893.07	1086.70	717.97
ASIR	Comoros	CD	1990	2475.00	3088.44	1991.68
ASDR	Comoros	CD	1990	21.96	33.29	13.51
ASPR	Congo	CD	2021	892.56	1086.01	717.51
ASIR	Congo	CD	2021	2473.63	3086.33	1990.61
ASDR	Congo	CD	2021	21.82	32.71	13.51
ASPR	Congo	CD	1990	895.53	1088.98	719.47
ASIR	Congo	CD	1990	2481.99	3097.86	1995.87

ASDR	Congo	CD	1990	21.93	33.25	13.73
ASPR	Cook Islands	CD	2021	1181.14	1438.11	967.99
ASIR	Cook Islands	CD	2021	3231.79	4026.84	2619.21
ASDR	Cook Islands	CD	2021	29.04	44.13	17.95
ASPR	Cook Islands	CD	1990	1173.94	1428.69	961.63
ASIR	Cook Islands	CD	1990	3212.06	4002.74	2602.99
ASDR	Cook Islands	CD	1990	29.09	44.79	18.11
ASPR	Costa Rica	CD	2021	1092.19	1337.22	888.86
ASIR	Costa Rica	CD	2021	3008.85	3739.89	2443.66
ASDR	Costa Rica	CD	2021	26.89	40.61	16.33
ASPR	Costa Rica	CD	1990	1089.46	1334.36	886.60
ASIR	Costa Rica	CD	1990	3001.05	3730.50	2437.39
ASDR	Costa Rica	CD	1990	26.94	41.03	16.65
ASPR	Croatia	CD	2021	1269.19	1544.04	1041.73
ASIR	Croatia	CD	2021	3448.23	4254.00	2809.25
ASDR	Croatia	CD	2021	31.39	47.17	19.30
ASPR	Croatia	CD	1990	1273.96	1550.26	1045.96
ASIR	Croatia	CD	1990	3460.49	4273.12	2818.95
ASDR	Croatia	CD	1990	31.47	47.62	19.82
ASPR	Cuba	CD	2021	1089.19	1333.29	886.01
ASIR	Cuba	CD	2021	3000.37	3729.35	2436.77
ASDR	Cuba	CD	2021	26.91	40.77	16.57
ASPR	Cuba	CD	1990	1088.63	1333.40	885.81
ASIR	Cuba	CD	1990	2998.81	3727.78	2435.46
ASDR	Cuba	CD	1990	26.93	41.05	16.53
ASPR	Cyprus	CD	2021	456.30	557.11	366.56
ASIR	Cyprus	CD	2021	1240.83	1544.01	1005.47
ASDR	Cyprus	CD	2021	11.35	17.41	6.96
ASPR	Cyprus	CD	1990	455.23	555.89	365.91
ASIR	Cyprus	CD	1990	1237.86	1541.12	1003.19
ASDR	Cyprus	CD	1990	11.33	17.15	7.10
ASPR	Czechia	CD	2021	1265.90	1540.42	1039.25
ASIR	Czechia	CD	2021	3439.42	4243.11	2802.78
ASDR	Czechia	CD	2021	31.21	47.69	19.05
ASPR	Czechia	CD	1990	1274.10	1549.88	1045.77
ASIR	Czechia	CD	1990	3461.16	4272.00	2818.67
ASDR	Czechia	CD	1990	31.39	47.33	19.78
ASPR	Democratic People's Republic of Korea	CD	2021	1180.19	1437.25	966.82
ASIR	Democratic People's Republic of	CD	2021	3229.76	4024.95	2619.35

	Korea					
ASDR	Democratic People's Republic of Korea	CD	2021	29.44	44.93	18.18
ASPR	Democratic People's Republic of Korea	CD	1990	1187.42	1447.03	972.98
ASIR	Democratic People's Republic of Korea	CD	1990	3249.44	4050.12	2633.97
ASDR	Democratic People's Republic of Korea	CD	1990	29.64	45.25	18.43
ASPR	Democratic Republic of the Congo	CD	2021	893.49	1087.57	717.98
ASIR	Democratic Republic of the Congo	CD	2021	2476.39	3089.36	1990.83
ASDR	Democratic Republic of the Congo	CD	2021	21.81	32.88	13.53
ASPR	Democratic Republic of the Congo	CD	1990	894.06	1087.97	718.34
ASIR	Democratic Republic of the Congo	CD	1990	2477.83	3093.69	1991.70
ASDR	Democratic Republic of the Congo	CD	1990	21.68	33.24	13.47
ASPR	Denmark	CD	2021	179.96	223.28	146.25
ASIR	Denmark	CD	2021	567.61	708.23	461.45
ASDR	Denmark	CD	2021	4.51	6.77	2.75
ASPR	Denmark	CD	1990	180.05	223.43	146.35
ASIR	Denmark	CD	1990	567.89	708.67	461.72
ASDR	Denmark	CD	1990	4.50	6.76	2.82
ASPR	Djibouti	CD	2021	886.54	1081.90	713.70

ASIR	Djibouti	CD	2021	2457.07	3066.30	1978.22
ASDR	Djibouti	CD	2021	21.94	33.66	13.30
ASPR	Djibouti	CD	1990	888.99	1084.08	715.11
ASIR	Djibouti	CD	1990	2463.84	3073.99	1982.06
ASDR	Djibouti	CD	1990	21.98	34.03	13.46
ASPR	Dominica	CD	2021	1087.95	1332.19	885.79
ASIR	Dominica	CD	2021	2996.67	3723.78	2433.71
ASDR	Dominica	CD	2021	26.82	40.77	16.18
ASPR	Dominica	CD	1990	1093.65	1336.53	889.10
ASIR	Dominica	CD	1990	3012.96	3743.07	2446.14
ASDR	Dominica	CD	1990	27.04	40.72	16.74
ASPR	Dominican Republic	CD	2021	1088.84	1333.22	885.93
ASIR	Dominican Republic	CD	2021	2999.25	3728.33	2435.71
ASDR	Dominican Republic	CD	2021	26.82	40.61	16.45
ASPR	Dominican Republic	CD	1990	1090.03	1335.76	887.25
ASIR	Dominican Republic	CD	1990	3002.55	3733.14	2438.86
ASDR	Dominican Republic	CD	1990	26.98	41.12	16.58
ASPR	Ecuador	CD	2021	1226.14	1496.61	994.42
ASIR	Ecuador	CD	2021	3314.53	4099.37	2686.19
ASDR	Ecuador	CD	2021	30.27	46.25	18.58
ASPR	Ecuador	CD	1990	1225.58	1494.90	994.57
ASIR	Ecuador	CD	1990	3312.86	4098.18	2684.39
ASDR	Ecuador	CD	1990	30.38	46.63	18.61
ASPR	Egypt	CD	2021	975.07	1185.60	793.82
ASIR	Egypt	CD	2021	2698.97	3311.57	2193.81
ASDR	Egypt	CD	2021	24.01	35.92	14.60
ASPR	Egypt	CD	1990	977.33	1187.90	795.68
ASIR	Egypt	CD	1990	2705.38	3318.58	2198.46
ASDR	Egypt	CD	1990	24.08	36.14	14.91
ASPR	El Salvador	CD	2021	1096.86	1342.66	892.56
ASIR	El Salvador	CD	2021	3022.21	3758.43	2453.96
ASDR	El Salvador	CD	2021	27.02	41.27	16.55
ASPR	El Salvador	CD	1990	1092.46	1338.08	889.31
ASIR	El Salvador	CD	1990	3009.49	3740.75	2444.18
ASDR	El Salvador	CD	1990	26.84	40.43	16.48
ASPR	Equatorial Guinea	CD	2021	892.96	1088.70	717.20

ASIR	Equatorial Guinea	CD	2021	2475.40	3090.72	1989.92
ASDR	Equatorial Guinea	CD	2021	21.77	33.34	13.47
ASPR	Equatorial Guinea	CD	1990	897.67	1090.26	721.19
ASIR	Equatorial Guinea	CD	1990	2487.77	3103.36	2002.04
ASDR	Equatorial Guinea	CD	1990	21.76	32.76	13.39
ASPR	Eritrea	CD	2021	895.46	1089.80	719.51
ASIR	Eritrea	CD	2021	2482.05	3097.29	1995.90
ASDR	Eritrea	CD	2021	21.94	33.16	13.56
ASPR	Eritrea	CD	1990	897.22	1090.66	720.87
ASIR	Eritrea	CD	1990	2486.86	3101.84	1999.77
ASDR	Eritrea	CD	1990	21.90	33.54	13.66
ASPR	Estonia	CD	2021	1177.24	1424.22	959.14
ASIR	Estonia	CD	2021	3219.96	3972.82	2641.84
ASDR	Estonia	CD	2021	29.18	44.97	18.17
ASPR	Estonia	CD	1990	1190.43	1439.36	969.33
ASIR	Estonia	CD	1990	3257.19	4024.70	2674.46
ASDR	Estonia	CD	1990	29.41	44.77	18.35
ASPR	Eswatini	CD	2021	898.06	1091.72	721.62
ASIR	Eswatini	CD	2021	2489.20	3106.33	2002.24
ASDR	Eswatini	CD	2021	21.81	33.14	13.67
ASPR	Eswatini	CD	1990	898.08	1090.54	722.06
ASIR	Eswatini	CD	1990	2488.93	3103.20	2003.86
ASDR	Eswatini	CD	1990	22.15	33.64	13.70
ASPR	Ethiopia	CD	2021	1004.32	1236.60	813.43
ASIR	Ethiopia	CD	2021	2802.34	3470.75	2248.79
ASDR	Ethiopia	CD	2021	24.59	37.32	15.25
ASPR	Ethiopia	CD	1990	1004.68	1236.13	813.94
ASIR	Ethiopia	CD	1990	2803.29	3474.55	2249.54
ASDR	Ethiopia	CD	1990	24.47	37.32	15.12
ASPR	Fiji	CD	2021	1178.55	1434.79	965.44
ASIR	Fiji	CD	2021	3225.10	4019.80	2614.41
ASDR	Fiji	CD	2021	29.01	44.47	18.29
ASPR	Fiji	CD	1990	1177.53	1433.43	964.54
ASIR	Fiji	CD	1990	3222.20	4016.68	2611.70
ASDR	Fiji	CD	1990	29.03	44.72	18.05
ASPR	Finland	CD	2021	454.37	554.96	365.27
ASIR	Finland	CD	2021	1235.51	1538.82	1001.63
ASDR	Finland	CD	2021	11.32	17.14	6.95
ASPR	Finland	CD	1990	455.53	556.61	366.36

ASIR	Finland	CD	1990	1238.75	1542.82	1003.90
ASDR	Finland	CD	1990	11.34	17.09	6.94
ASPR	France	CD	2021	1052.21	1273.91	847.31
ASIR	France	CD	2021	2236.39	2829.68	1780.89
ASDR	France	CD	2021	26.12	39.12	15.81
ASPR	France	CD	1990	1051.38	1273.20	846.27
ASIR	France	CD	1990	2234.63	2828.22	1779.31
ASDR	France	CD	1990	26.16	38.98	16.13
ASPR	Gabon	CD	2021	894.59	1087.80	718.90
ASIR	Gabon	CD	2021	2479.08	3091.98	1995.26
ASDR	Gabon	CD	2021	21.91	33.00	13.64
ASPR	Gabon	CD	1990	893.59	1087.97	718.22
ASIR	Gabon	CD	1990	2476.53	3090.89	1990.28
ASDR	Gabon	CD	1990	21.85	33.28	13.34
ASPR	Gambia	CD	2021	906.44	1100.72	738.52
ASIR	Gambia	CD	2021	2495.06	3083.46	2029.18
ASDR	Gambia	CD	2021	22.27	33.69	13.64
ASPR	Gambia	CD	1990	902.59	1095.32	735.36
ASIR	Gambia	CD	1990	2484.19	3069.53	2019.05
ASDR	Gambia	CD	1990	22.23	34.01	13.76
ASPR	Georgia	CD	2021	1273.60	1549.09	1045.34
ASIR	Georgia	CD	2021	3460.34	4273.08	2816.55
ASDR	Georgia	CD	2021	31.48	47.95	19.67
ASPR	Georgia	CD	1990	1285.95	1564.79	1055.26
ASIR	Georgia	CD	1990	3493.55	4313.54	2842.70
ASDR	Georgia	CD	1990	31.88	48.04	19.72
ASPR	Germany	CD	2021	745.90	875.61	627.11
ASIR	Germany	CD	2021	1801.76	2204.93	1436.42
ASDR	Germany	CD	2021	18.56	27.31	11.38
ASPR	Germany	CD	1990	747.06	877.48	627.55
ASIR	Germany	CD	1990	1805.49	2212.41	1438.57
ASDR	Germany	CD	1990	18.59	26.54	11.44
ASPR	Ghana	CD	2021	909.94	1105.07	741.22
ASIR	Ghana	CD	2021	2505.10	3096.08	2039.95
ASDR	Ghana	CD	2021	22.45	33.92	13.72
ASPR	Ghana	CD	1990	906.44	1100.92	738.64
ASIR	Ghana	CD	1990	2495.22	3082.92	2029.73
ASDR	Ghana	CD	1990	22.26	33.89	13.91
ASPR	Greece	CD	2021	456.07	557.16	366.56
ASIR	Greece	CD	2021	1240.19	1544.66	1005.21
ASDR	Greece	CD	2021	11.36	17.08	6.96
ASPR	Greece	CD	1990	456.03	557.20	366.61
ASIR	Greece	CD	1990	1240.09	1544.64	1005.20
ASDR	Greece	CD	1990	11.36	17.44	7.09

ASPR	Greenland	CD	2021	1116.10	1334.86	901.13
ASIR	Greenland	CD	2021	2648.48	3307.42	2154.63
ASDR	Greenland	CD	2021	27.55	41.16	17.20
ASPR	Greenland	CD	1990	1108.29	1322.82	895.45
ASIR	Greenland	CD	1990	2630.60	3286.06	2141.89
ASDR	Greenland	CD	1990	27.34	41.33	17.25
ASPR	Grenada	CD	2021	1088.37	1332.29	886.37
ASIR	Grenada	CD	2021	2998.00	3724.71	2434.94
ASDR	Grenada	CD	2021	26.76	40.47	16.58
ASPR	Grenada	CD	1990	1092.67	1336.30	888.93
ASIR	Grenada	CD	1990	3010.10	3740.93	2444.30
ASDR	Grenada	CD	1990	26.99	40.75	16.34
ASPR	Guam	CD	2021	1177.11	1432.48	963.95
ASIR	Guam	CD	2021	3221.15	4015.85	2611.91
ASDR	Guam	CD	2021	29.27	44.55	18.11
ASPR	Guam	CD	1990	1172.47	1426.63	959.97
ASIR	Guam	CD	1990	3208.42	4000.14	2600.97
ASDR	Guam	CD	1990	29.16	44.51	18.07
ASPR	Guatemala	CD	2021	1092.60	1338.22	889.27
ASIR	Guatemala	CD	2021	3010.01	3741.73	2444.56
ASDR	Guatemala	CD	2021	26.72	40.79	16.34
ASPR	Guatemala	CD	1990	1089.94	1335.73	887.43
ASIR	Guatemala	CD	1990	3002.25	3732.40	2438.86
ASDR	Guatemala	CD	1990	26.72	40.70	16.30
ASPR	Guinea	CD	2021	907.10	1102.69	739.37
ASIR	Guinea	CD	2021	2496.97	3088.17	2031.64
ASDR	Guinea	CD	2021	22.34	33.68	13.86
ASPR	Guinea	CD	1990	907.03	1102.47	739.18
ASIR	Guinea	CD	1990	2496.85	3086.42	2030.15
ASDR	Guinea	CD	1990	22.25	33.91	13.85
ASPR	Guinea-Bissau	CD	2021	909.33	1104.45	740.75
ASIR	Guinea-Bissau	CD	2021	2503.28	3094.76	2038.56
ASDR	Guinea-Bissau	CD	2021	22.34	33.83	13.81
ASPR	Guinea-Bissau	CD	1990	908.12	1103.49	739.96
ASIR	Guinea-Bissau	CD	1990	2499.85	3092.17	2035.62
ASDR	Guinea-Bissau	CD	1990	22.25	33.81	13.71
ASPR	Guyana	CD	2021	1091.19	1335.63	888.10
ASIR	Guyana	CD	2021	3005.99	3736.10	2441.64
ASDR	Guyana	CD	2021	26.55	40.92	16.58
ASPR	Guyana	CD	1990	1089.98	1334.79	887.01
ASIR	Guyana	CD	1990	3002.40	3732.42	2438.50
ASDR	Guyana	CD	1990	26.66	40.94	16.36
ASPR	Haiti	CD	2021	1091.29	1336.56	887.99
ASIR	Haiti	CD	2021	3006.34	3737.19	2441.61

ASDR	Haiti	CD	2021	26.66	40.17	16.34
ASPR	Haiti	CD	1990	1091.16	1336.74	888.63
ASIR	Haiti	CD	1990	3005.65	3736.23	2441.56
ASDR	Haiti	CD	1990	26.73	40.37	16.46
ASPR	Honduras	CD	2021	1092.68	1338.24	889.44
ASIR	Honduras	CD	2021	3010.27	3742.12	2444.73
ASDR	Honduras	CD	2021	26.80	41.08	16.37
ASPR	Honduras	CD	1990	1090.22	1335.87	887.66
ASIR	Honduras	CD	1990	3003.15	3733.38	2439.26
ASDR	Honduras	CD	1990	26.91	41.50	16.32
ASPR	Hungary	CD	2021	1271.60	1547.03	1043.83
ASIR	Hungary	CD	2021	3454.62	4263.67	2814.02
ASDR	Hungary	CD	2021	31.39	46.89	19.17
ASPR	Hungary	CD	1990	1278.00	1554.04	1048.59
ASIR	Hungary	CD	1990	3471.67	4284.60	2826.32
ASDR	Hungary	CD	1990	31.42	47.19	19.55
ASPR	Iceland	CD	2021	453.88	554.29	364.82
ASIR	Iceland	CD	2021	1234.17	1536.77	1000.43
ASDR	Iceland	CD	2021	11.29	17.10	6.86
ASPR	Iceland	CD	1990	454.80	555.62	365.60
ASIR	Iceland	CD	1990	1236.68	1540.29	1002.65
ASDR	Iceland	CD	1990	11.38	17.33	6.96
ASPR	India	CD	2021	1086.26	1323.32	877.28
ASIR	India	CD	2021	3009.76	3704.92	2443.06
ASDR	India	CD	2021	26.49	40.37	16.37
ASPR	India	CD	1990	1083.38	1321.01	874.31
ASIR	India	CD	1990	3002.07	3692.90	2438.31
ASDR	India	CD	1990	26.31	40.12	16.20
ASPR	Indonesia	CD	2021	1526.75	1878.29	1220.63
ASIR	Indonesia	CD	2021	4180.43	5253.26	3371.68
ASDR	Indonesia	CD	2021	37.91	58.30	23.35
ASPR	Indonesia	CD	1990	1528.73	1881.60	1221.97
ASIR	Indonesia	CD	1990	4185.69	5259.22	3375.68
ASDR	Indonesia	CD	1990	37.89	58.32	23.22
ASPR	Iran (Islamic Republic of)	CD	2021	1106.16	1370.88	893.29
ASIR	Iran (Islamic Republic of)	CD	2021	3084.36	3819.88	2476.03
ASDR	Iran (Islamic Republic of)	CD	2021	27.04	41.74	16.71
ASPR	Iran (Islamic Republic of)	CD	1990	1104.90	1370.54	892.23
ASIR	Iran (Islamic Republic of)	CD	1990	3080.76	3820.91	2474.28

ASDR	Iran (Islamic Republic of)	CD	1990	27.04	41.74	16.76
ASPR	Iraq	CD	2021	977.85	1188.17	796.34
ASIR	Iraq	CD	2021	2706.85	3319.21	2198.19
ASDR	Iraq	CD	2021	23.75	36.34	14.73
ASPR	Iraq	CD	1990	978.42	1188.84	796.76
ASIR	Iraq	CD	1990	2708.50	3321.40	2199.61
ASDR	Iraq	CD	1990	23.95	36.26	14.75
ASPR	Ireland	CD	2021	456.14	557.19	366.54
ASIR	Ireland	CD	2021	1240.35	1545.45	1005.36
ASDR	Ireland	CD	2021	11.34	17.47	6.85
ASPR	Ireland	CD	1990	455.42	556.46	366.09
ASIR	Ireland	CD	1990	1238.34	1543.17	1004.05
ASDR	Ireland	CD	1990	11.33	17.21	6.92
ASPR	Israel	CD	2021	455.56	556.50	366.21
ASIR	Israel	CD	2021	1238.78	1542.94	1004.10
ASDR	Israel	CD	2021	11.39	17.10	6.84
ASPR	Israel	CD	1990	456.95	558.35	367.34
ASIR	Israel	CD	1990	1242.62	1547.77	1007.09
ASDR	Israel	CD	1990	11.38	16.93	6.98
ASPR	Italy	CD	2021	643.04	788.85	510.35
ASIR	Italy	CD	2021	1693.03	2131.84	1366.68
ASDR	Italy	CD	2021	16.02	24.34	9.98
ASPR	Italy	CD	1990	644.46	790.63	511.63
ASIR	Italy	CD	1990	1696.91	2136.70	1369.79
ASDR	Italy	CD	1990	16.02	24.01	10.00
ASPR	Jamaica	CD	2021	1089.58	1334.30	886.92
ASIR	Jamaica	CD	2021	3001.49	3730.56	2438.45
ASDR	Jamaica	CD	2021	26.95	41.35	16.56
ASPR	Jamaica	CD	1990	1091.10	1335.97	888.15
ASIR	Jamaica	CD	1990	3005.63	3735.88	2441.21
ASDR	Jamaica	CD	1990	27.05	40.83	16.70
ASPR	Japan	CD	2021	308.73	380.04	251.83
ASIR	Japan	CD	2021	864.70	1070.24	698.13
ASDR	Japan	CD	2021	7.69	11.73	4.75
ASPR	Japan	CD	1990	309.54	380.78	252.55
ASIR	Japan	CD	1990	866.91	1072.98	699.79
ASDR	Japan	CD	1990	7.69	11.70	4.65
ASPR	Jordan	CD	2021	973.54	1183.39	793.24
ASIR	Jordan	CD	2021	2694.77	3305.35	2188.84
ASDR	Jordan	CD	2021	23.86	36.74	14.45
ASPR	Jordan	CD	1990	976.31	1187.05	795.23
ASIR	Jordan	CD	1990	2702.52	3313.90	2194.97
ASDR	Jordan	CD	1990	23.96	36.04	14.83

ASPR	Kazakhstan	CD	2021	1281.10	1558.51	1051.45
ASIR	Kazakhstan	CD	2021	3480.18	4295.29	2832.28
ASDR	Kazakhstan	CD	2021	31.61	47.53	19.79
ASPR	Kazakhstan	CD	1990	1282.64	1560.48	1052.31
ASIR	Kazakhstan	CD	1990	3483.79	4304.33	2838.08
ASDR	Kazakhstan	CD	1990	31.64	48.03	19.64
ASPR	Kenya	CD	2021	1007.30	1239.24	815.34
ASIR	Kenya	CD	2021	2810.63	3480.57	2254.16
ASDR	Kenya	CD	2021	24.68	37.58	15.32
ASPR	Kenya	CD	1990	1005.62	1237.67	814.32
ASIR	Kenya	CD	1990	2805.85	3474.50	2250.48
ASDR	Kenya	CD	1990	24.64	37.31	15.18
ASPR	Kiribati	CD	2021	1184.14	1441.78	970.19
ASIR	Kiribati	CD	2021	3240.75	4040.03	2628.21
ASDR	Kiribati	CD	2021	29.13	44.45	17.94
ASPR	Kiribati	CD	1990	1182.35	1439.71	968.52
ASIR	Kiribati	CD	1990	3235.72	4032.99	2623.18
ASDR	Kiribati	CD	1990	29.02	44.25	18.20
ASPR	Kuwait	CD	2021	971.86	1182.76	792.04
ASIR	Kuwait	CD	2021	2689.66	3300.09	2187.58
ASDR	Kuwait	CD	2021	23.81	35.59	14.66
ASPR	Kuwait	CD	1990	963.12	1172.38	786.39
ASIR	Kuwait	CD	1990	2665.07	3272.63	2167.01
ASDR	Kuwait	CD	1990	23.81	36.18	14.73
ASPR	Kyrgyzstan	CD	2021	1278.82	1555.35	1049.18
ASIR	Kyrgyzstan	CD	2021	3474.09	4286.69	2828.32
ASDR	Kyrgyzstan	CD	2021	31.68	47.84	19.55
ASPR	Kyrgyzstan	CD	1990	1280.16	1557.66	1050.37
ASIR	Kyrgyzstan	CD	1990	3477.27	4293.74	2832.77
ASDR	Kyrgyzstan	CD	1990	31.67	48.64	19.76
ASPR	Lao People's Democratic Republic	CD	2021	1369.61	1706.90	1109.48
ASIR	Lao People's Democratic Republic	CD	2021	3671.88	4564.39	2981.56
ASDR	Lao People's Democratic Republic	CD	2021	34.08	51.45	21.07
ASPR	Lao People's Democratic Republic	CD	1990	1372.89	1711.63	1112.34
ASIR	Lao People's Democratic	CD	1990	3680.74	4577.95	2991.06

	Republic					
ASDR	Lao People's Democratic Republic	CD	1990	33.98	51.97	20.76
ASPR	Latvia	CD	2021	1182.87	1431.04	963.65
ASIR	Latvia	CD	2021	3235.89	3994.39	2655.83
ASDR	Latvia	CD	2021	29.26	44.24	18.23
ASPR	Latvia	CD	1990	1192.67	1442.16	970.87
ASIR	Latvia	CD	1990	3263.27	4031.49	2679.49
ASDR	Latvia	CD	1990	29.41	44.33	18.43
ASPR	Lebanon	CD	2021	981.94	1192.34	799.21
ASIR	Lebanon	CD	2021	2718.62	3333.04	2206.89
ASDR	Lebanon	CD	2021	23.96	36.33	14.73
ASPR	Lebanon	CD	1990	981.90	1194.06	799.22
ASIR	Lebanon	CD	1990	2717.95	3331.90	2207.31
ASDR	Lebanon	CD	1990	24.08	36.56	14.69
ASPR	Lesotho	CD	2021	898.64	1092.55	721.99
ASIR	Lesotho	CD	2021	2490.82	3107.81	2003.73
ASDR	Lesotho	CD	2021	21.75	32.86	13.55
ASPR	Lesotho	CD	1990	904.41	1098.37	726.52
ASIR	Lesotho	CD	1990	2506.58	3126.86	2017.35
ASDR	Lesotho	CD	1990	22.23	33.85	13.72
ASPR	Liberia	CD	2021	902.94	1095.91	735.61
ASIR	Liberia	CD	2021	2485.35	3070.82	2020.64
ASDR	Liberia	CD	2021	21.93	33.20	13.74
ASPR	Liberia	CD	1990	902.32	1094.82	735.31
ASIR	Liberia	CD	1990	2483.52	3069.26	2019.04
ASDR	Liberia	CD	1990	21.89	33.01	13.52
ASPR	Libya	CD	2021	977.61	1188.23	796.15
ASIR	Libya	CD	2021	2706.09	3318.81	2198.65
ASDR	Libya	CD	2021	23.88	36.40	14.68
ASPR	Libya	CD	1990	972.94	1182.61	792.92
ASIR	Libya	CD	1990	2692.96	3303.39	2188.13
ASDR	Libya	CD	1990	24.00	36.16	14.84
ASPR	Lithuania	CD	2021	1184.08	1432.43	964.69
ASIR	Lithuania	CD	2021	3239.22	3998.16	2658.84
ASDR	Lithuania	CD	2021	29.28	44.97	18.32
ASPR	Lithuania	CD	1990	1192.57	1442.04	971.23
ASIR	Lithuania	CD	1990	3262.77	4029.26	2679.95
ASDR	Lithuania	CD	1990	29.43	44.32	18.37
ASPR	Luxembourg	CD	2021	454.32	554.89	365.18
ASIR	Luxembourg	CD	2021	1235.30	1539.38	1001.69
ASDR	Luxembourg	CD	2021	11.33	17.40	6.79

ASPR	Luxembourg	CD	1990	455.15	556.24	366.09
ASIR	Luxembourg	CD	1990	1237.68	1541.83	1003.30
ASDR	Luxembourg	CD	1990	11.33	17.11	7.05
ASPR	Madagascar	CD	2021	893.29	1087.04	717.87
ASIR	Madagascar	CD	2021	2475.60	3089.30	1991.33
ASDR	Madagascar	CD	2021	22.01	33.53	13.60
ASPR	Madagascar	CD	1990	891.48	1085.53	716.77
ASIR	Madagascar	CD	1990	2470.53	3084.05	1987.30
ASDR	Madagascar	CD	1990	21.87	32.91	13.60
ASPR	Malawi	CD	2021	895.60	1088.91	719.76
ASIR	Malawi	CD	2021	2482.11	3097.15	1996.22
ASDR	Malawi	CD	2021	22.06	33.81	13.78
ASPR	Malawi	CD	1990	893.78	1087.34	718.17
ASIR	Malawi	CD	1990	2477.03	3091.63	1991.62
ASDR	Malawi	CD	1990	21.85	33.05	13.60
ASPR	Malaysia	CD	2021	1366.17	1702.05	1106.33
ASIR	Malaysia	CD	2021	3662.45	4549.32	2972.06
ASDR	Malaysia	CD	2021	33.94	51.74	20.78
ASPR	Malaysia	CD	1990	1369.26	1706.50	1109.06
ASIR	Malaysia	CD	1990	3670.94	4563.44	2980.01
ASDR	Malaysia	CD	1990	33.96	51.55	20.89
ASPR	Maldives	CD	2021	1350.34	1678.43	1095.56
ASIR	Maldives	CD	2021	3618.84	4479.83	2942.39
ASDR	Maldives	CD	2021	33.66	50.62	20.63
ASPR	Maldives	CD	1990	1361.38	1696.93	1102.02
ASIR	Maldives	CD	1990	3648.52	4531.22	2963.58
ASDR	Maldives	CD	1990	33.76	51.57	20.64
ASPR	Mali	CD	2021	904.80	1099.06	737.35
ASIR	Mali	CD	2021	2490.61	3079.30	2025.52
ASDR	Mali	CD	2021	22.22	33.69	13.47
ASPR	Mali	CD	1990	906.26	1100.93	738.61
ASIR	Mali	CD	1990	2494.67	3083.51	2028.91
ASDR	Mali	CD	1990	22.19	33.69	13.75
ASPR	Malta	CD	2021	454.07	554.52	365.00
ASIR	Malta	CD	2021	1234.66	1537.73	1000.93
ASDR	Malta	CD	2021	11.34	17.36	7.01
ASPR	Malta	CD	1990	456.15	557.19	366.60
ASIR	Malta	CD	1990	1240.43	1544.04	1005.18
ASDR	Malta	CD	1990	11.38	17.03	6.87
ASPR	Marshall Islands	CD	2021	1175.74	1431.52	962.96
ASIR	Marshall Islands	CD	2021	3217.20	4011.16	2607.18
ASDR	Marshall	CD	2021	28.86	43.40	17.95

	Islands					
ASPR	Marshall Islands	CD	1990	1176.62	1432.58	963.96
ASIR	Marshall Islands	CD	1990	3219.72	4012.69	2608.83
ASDR	Marshall Islands	CD	1990	29.03	44.11	18.11
ASPR	Mauritania	CD	2021	906.22	1100.72	738.06
ASIR	Mauritania	CD	2021	2494.47	3083.97	2030.06
ASDR	Mauritania	CD	2021	22.39	34.17	13.86
ASPR	Mauritania	CD	1990	906.91	1101.22	738.72
ASIR	Mauritania	CD	1990	2496.41	3085.03	2031.58
ASDR	Mauritania	CD	1990	22.35	33.72	13.82
ASPR	Mauritius	CD	2021	1370.57	1708.14	1110.44
ASIR	Mauritius	CD	2021	3674.56	4567.48	2984.22
ASDR	Mauritius	CD	2021	33.83	51.32	21.29
ASPR	Mauritius	CD	1990	1371.53	1709.31	1111.40
ASIR	Mauritius	CD	1990	3677.26	4570.74	2987.59
ASDR	Mauritius	CD	1990	33.99	51.93	20.78
ASPR	Mexico	CD	2021	1235.16	1523.72	989.80
ASIR	Mexico	CD	2021	3437.82	4329.55	2762.40
ASDR	Mexico	CD	2021	30.28	46.09	18.85
ASPR	Mexico	CD	1990	1233.85	1522.15	988.73
ASIR	Mexico	CD	1990	3433.88	4324.84	2759.66
ASDR	Mexico	CD	1990	30.24	46.09	18.81
ASPR	Micronesia (Federated States of)	CD	2021	1178.23	1434.25	965.01
ASIR	Micronesia (Federated States of)	CD	2021	3224.22	4018.31	2614.20
ASDR	Micronesia (Federated States of)	CD	2021	29.07	44.37	18.24
ASPR	Micronesia (Federated States of)	CD	1990	1177.08	1433.28	964.33
ASIR	Micronesia (Federated States of)	CD	1990	3220.84	4012.22	2610.51
ASDR	Micronesia (Federated States of)	CD	1990	29.08	43.91	18.12
ASPR	Monaco	CD	2021	456.19	557.20	366.63

ASIR	Monaco	CD	2021	1240.53	1545.32	1005.35
ASDR	Monaco	CD	2021	11.38	17.24	7.10
ASPR	Monaco	CD	1990	456.55	557.79	366.92
ASIR	Monaco	CD	1990	1241.49	1546.11	1006.08
ASDR	Monaco	CD	1990	11.41	17.45	7.12
ASPR	Mongolia	CD	2021	1280.12	1556.74	1050.08
ASIR	Mongolia	CD	2021	3477.20	4291.67	2830.52
ASDR	Mongolia	CD	2021	31.60	47.78	19.60
ASPR	Mongolia	CD	1990	1272.06	1548.22	1043.82
ASIR	Mongolia	CD	1990	3456.10	4264.41	2815.11
ASDR	Mongolia	CD	1990	31.42	47.66	19.60
ASPR	Montenegro	CD	2021	1270.37	1545.19	1042.95
ASIR	Montenegro	CD	2021	3451.71	4259.89	2811.74
ASDR	Montenegro	CD	2021	31.35	47.66	19.30
ASPR	Montenegro	CD	1990	1272.66	1547.62	1044.79
ASIR	Montenegro	CD	1990	3457.15	4269.17	2815.97
ASDR	Montenegro	CD	1990	31.50	48.50	19.80
ASPR	Morocco	CD	2021	979.37	1190.29	797.60
ASIR	Morocco	CD	2021	2711.01	3324.06	2201.76
ASDR	Morocco	CD	2021	23.96	36.33	14.42
ASPR	Morocco	CD	1990	979.68	1190.62	797.70
ASIR	Morocco	CD	1990	2711.84	3325.77	2203.77
ASDR	Morocco	CD	1990	24.09	36.59	14.97
ASPR	Mozambique	CD	2021	896.98	1090.11	720.70
ASIR	Mozambique	CD	2021	2485.87	3100.60	1999.20
ASDR	Mozambique	CD	2021	21.80	32.73	13.74
ASPR	Mozambique	CD	1990	895.58	1088.21	719.82
ASIR	Mozambique	CD	1990	2481.83	3096.56	1997.65
ASDR	Mozambique	CD	1990	21.77	33.49	13.51
ASPR	Myanmar	CD	2021	1374.94	1714.02	1114.38
ASIR	Myanmar	CD	2021	3686.82	4586.41	2996.95
ASDR	Myanmar	CD	2021	34.17	51.14	20.98
ASPR	Myanmar	CD	1990	1372.13	1710.37	1111.79
ASIR	Myanmar	CD	1990	3678.80	4575.58	2988.42
ASDR	Myanmar	CD	1990	34.01	51.04	21.05
ASPR	Namibia	CD	2021	896.92	1090.37	720.65
ASIR	Namibia	CD	2021	2485.91	3101.59	1999.23
ASDR	Namibia	CD	2021	21.97	33.33	13.49
ASPR	Namibia	CD	1990	895.31	1088.75	719.53
ASIR	Namibia	CD	1990	2481.33	3095.62	1995.50
ASDR	Namibia	CD	1990	22.07	33.88	13.46
ASPR	Nauru	CD	2021	1180.99	1438.34	966.84
ASIR	Nauru	CD	2021	3232.29	4030.93	2620.36
ASDR	Nauru	CD	2021	29.08	44.01	18.07

ASPR	Nauru	CD	1990	1170.76	1425.30	958.23
ASIR	Nauru	CD	1990	3203.33	3994.55	2593.04
ASDR	Nauru	CD	1990	28.99	43.88	18.11
ASPR	Nepal	CD	2021	959.04	1177.79	772.43
ASIR	Nepal	CD	2021	2652.49	3296.51	2141.75
ASDR	Nepal	CD	2021	23.43	35.29	14.28
ASPR	Nepal	CD	1990	953.50	1172.17	768.26
ASIR	Nepal	CD	1990	2636.33	3278.97	2128.55
ASDR	Nepal	CD	1990	23.26	35.00	14.29
ASPR	Netherlands	CD	2021	408.67	503.02	329.57
ASIR	Netherlands	CD	2021	1133.83	1401.11	923.23
ASDR	Netherlands	CD	2021	10.17	15.45	6.26
ASPR	Netherlands	CD	1990	474.70	588.42	376.44
ASIR	Netherlands	CD	1990	1273.57	1592.07	1022.20
ASDR	Netherlands	CD	1990	11.85	18.13	7.26
ASPR	New Zealand	CD	2021	309.19	380.42	252.13
ASIR	New Zealand	CD	2021	866.00	1072.13	699.49
ASDR	New Zealand	CD	2021	7.60	11.57	4.77
ASPR	New Zealand	CD	1990	309.34	381.02	252.40
ASIR	New Zealand	CD	1990	866.38	1072.57	699.14
ASDR	New Zealand	CD	1990	7.56	11.49	4.65
ASPR	Nicaragua	CD	2021	1092.01	1336.47	888.49
ASIR	Nicaragua	CD	2021	3008.39	3738.89	2443.14
ASDR	Nicaragua	CD	2021	26.82	41.02	16.75
ASPR	Nicaragua	CD	1990	1091.91	1337.38	889.23
ASIR	Nicaragua	CD	1990	3007.86	3738.53	2443.42
ASDR	Nicaragua	CD	1990	26.95	41.26	16.68
ASPR	Niger	CD	2021	906.58	1100.78	737.93
ASIR	Niger	CD	2021	2495.39	3084.47	2031.64
ASDR	Niger	CD	2021	22.34	34.15	13.82
ASPR	Niger	CD	1990	904.07	1097.53	736.83
ASIR	Niger	CD	1990	2488.30	3075.22	2022.88
ASDR	Niger	CD	1990	22.17	33.39	13.55
ASPR	Nigeria	CD	2021	1065.92	1300.14	863.70
ASIR	Nigeria	CD	2021	2913.58	3614.72	2369.80
ASDR	Nigeria	CD	2021	26.17	39.71	16.11
ASPR	Nigeria	CD	1990	1064.54	1296.85	862.94
ASIR	Nigeria	CD	1990	2899.21	3607.11	2357.25
ASDR	Nigeria	CD	1990	26.02	39.64	16.11
ASPR	Niue	CD	2021	1178.90	1435.08	965.65
ASIR	Niue	CD	2021	3226.49	4020.99	2616.45
ASDR	Niue	CD	2021	29.06	44.06	17.98
ASPR	Niue	CD	1990	1179.77	1435.89	966.18
ASIR	Niue	CD	1990	3228.78	4023.31	2619.66

ASDR	Niue	CD	1990	29.19	44.18	18.01
ASPR	North Macedonia	CD	2021	1262.89	1536.51	1036.79
ASIR	North Macedonia	CD	2021	3431.44	4233.21	2797.06
ASDR	North Macedonia	CD	2021	31.10	46.96	19.28
ASPR	North Macedonia	CD	1990	1271.98	1546.87	1043.42
ASIR	North Macedonia	CD	1990	3455.78	4262.55	2815.70
ASDR	North Macedonia	CD	1990	31.39	47.05	19.68
ASPR	Northern Mariana Islands	CD	2021	1173.41	1427.88	961.60
ASIR	Northern Mariana Islands	CD	2021	3210.71	4002.83	2601.99
ASDR	Northern Mariana Islands	CD	2021	29.03	44.72	18.18
ASPR	Northern Mariana Islands	CD	1990	1167.55	1422.56	953.92
ASIR	Northern Mariana Islands	CD	1990	3193.59	3977.51	2582.68
ASDR	Northern Mariana Islands	CD	1990	29.03	44.42	17.75
ASPR	Norway	CD	2021	608.93	759.49	483.22
ASIR	Norway	CD	2021	1645.50	2054.12	1321.03
ASDR	Norway	CD	2021	15.13	23.19	9.43
ASPR	Norway	CD	1990	609.57	760.47	483.95
ASIR	Norway	CD	1990	1647.29	2056.49	1323.59
ASDR	Norway	CD	1990	15.10	22.72	9.27
ASPR	Oman	CD	2021	964.18	1172.18	786.89
ASIR	Oman	CD	2021	2668.56	3273.39	2166.00
ASDR	Oman	CD	2021	23.72	36.09	14.55
ASPR	Oman	CD	1990	964.34	1171.45	786.79
ASIR	Oman	CD	1990	2669.27	3272.38	2166.33
ASDR	Oman	CD	1990	23.68	35.53	14.56
ASPR	Pakistan	CD	2021	1072.96	1327.92	883.61

ASIR	Pakistan	CD	2021	2986.62	3692.99	2414.74
ASDR	Pakistan	CD	2021	26.24	40.49	16.15
ASPR	Pakistan	CD	1990	1069.25	1325.02	880.32
ASIR	Pakistan	CD	1990	2976.49	3680.74	2405.84
ASDR	Pakistan	CD	1990	26.18	40.13	16.23
ASPR	Palau	CD	2021	1167.98	1422.09	957.02
ASIR	Palau	CD	2021	3196.17	3988.37	2590.47
ASDR	Palau	CD	2021	28.75	43.41	17.82
ASPR	Palau	CD	1990	1176.16	1431.51	963.17
ASIR	Palau	CD	1990	3218.53	4012.79	2609.15
ASDR	Palau	CD	1990	29.09	44.36	17.95
ASPR	Palestine	CD	2021	979.56	1190.57	797.88
ASIR	Palestine	CD	2021	2711.51	3323.86	2202.45
ASDR	Palestine	CD	2021	23.96	35.70	15.07
ASPR	Palestine	CD	1990	983.49	1194.16	800.32
ASIR	Palestine	CD	1990	2722.83	3337.56	2210.62
ASDR	Palestine	CD	1990	24.14	36.70	14.72
ASPR	Panama	CD	2021	1088.63	1333.05	885.81
ASIR	Panama	CD	2021	2998.76	3727.47	2435.70
ASDR	Panama	CD	2021	26.86	40.83	16.42
ASPR	Panama	CD	1990	1087.54	1332.44	885.15
ASIR	Panama	CD	1990	2995.63	3724.06	2433.34
ASDR	Panama	CD	1990	26.95	41.22	16.54
ASPR	Papua New Guinea	CD	2021	1174.06	1428.67	961.30
ASIR	Papua New Guinea	CD	2021	3212.63	4004.95	2603.50
ASDR	Papua New Guinea	CD	2021	28.90	44.97	18.03
ASPR	Papua New Guinea	CD	1990	1174.27	1429.30	961.81
ASIR	Papua New Guinea	CD	1990	3213.30	4005.95	2603.55
ASDR	Papua New Guinea	CD	1990	28.84	44.07	18.07
ASPR	Paraguay	CD	2021	1088.95	1333.12	886.13
ASIR	Paraguay	CD	2021	2999.59	3728.28	2436.13
ASDR	Paraguay	CD	2021	26.75	40.52	16.37
ASPR	Paraguay	CD	1990	1089.25	1333.58	886.37
ASIR	Paraguay	CD	1990	3000.34	3729.66	2436.47
ASDR	Paraguay	CD	1990	26.84	40.29	16.38
ASPR	Peru	CD	2021	1329.87	1594.10	1104.30
ASIR	Peru	CD	2021	3504.99	4318.76	2862.24
ASDR	Peru	CD	2021	33.00	49.82	20.86

ASPR	Peru	CD	1990	1330.21	1594.26	1104.34
ASIR	Peru	CD	1990	3505.40	4318.24	2863.10
ASDR	Peru	CD	1990	32.97	49.16	20.46
ASPR	Philippines	CD	2021	1527.87	1879.56	1221.10
ASIR	Philippines	CD	2021	4183.58	5256.32	3374.06
ASDR	Philippines	CD	2021	37.81	58.05	23.27
ASPR	Philippines	CD	1990	1527.28	1879.68	1220.83
ASIR	Philippines	CD	1990	4181.93	5256.53	3372.82
ASDR	Philippines	CD	1990	37.69	57.75	23.21
ASPR	Poland	CD	2021	1415.01	1711.73	1162.10
ASIR	Poland	CD	2021	3906.63	4806.17	3161.11
ASDR	Poland	CD	2021	34.83	52.96	21.28
ASPR	Poland	CD	1990	1421.01	1720.20	1166.93
ASIR	Poland	CD	1990	3922.89	4825.11	3173.05
ASDR	Poland	CD	1990	34.83	52.30	21.17
ASPR	Portugal	CD	2021	457.01	558.33	367.25
ASIR	Portugal	CD	2021	1242.77	1547.55	1006.99
ASDR	Portugal	CD	2021	11.36	16.82	6.97
ASPR	Portugal	CD	1990	457.19	558.67	367.38
ASIR	Portugal	CD	1990	1243.30	1548.08	1007.37
ASDR	Portugal	CD	1990	11.34	17.15	6.99
ASPR	Puerto Rico	CD	2021	1092.24	1336.79	888.83
ASIR	Puerto Rico	CD	2021	3009.03	3739.83	2443.76
ASDR	Puerto Rico	CD	2021	26.94	41.20	16.34
ASPR	Puerto Rico	CD	1990	1092.39	1337.72	889.15
ASIR	Puerto Rico	CD	1990	3009.49	3740.59	2444.07
ASDR	Puerto Rico	CD	1990	27.02	41.14	16.54
ASPR	Qatar	CD	2021	951.55	1157.81	777.05
ASIR	Qatar	CD	2021	2633.13	3231.96	2137.97
ASDR	Qatar	CD	2021	23.33	34.98	14.61
ASPR	Qatar	CD	1990	951.24	1157.91	776.37
ASIR	Qatar	CD	1990	2632.33	3230.42	2137.86
ASDR	Qatar	CD	1990	23.48	36.02	14.23
ASPR	Republic of Korea	CD	2021	272.01	330.08	220.12
ASIR	Republic of Korea	CD	2021	764.33	937.34	625.60
ASDR	Republic of Korea	CD	2021	6.75	10.45	4.11
ASPR	Republic of Korea	CD	1990	273.48	331.72	221.17
ASIR	Republic of Korea	CD	1990	768.43	941.81	628.75

ASDR	Republic of Korea	CD	1990	6.78	10.43	4.19
ASPR	Republic of Moldova	CD	2021	1183.59	1431.50	964.38
ASIR	Republic of Moldova	CD	2021	3237.66	3996.18	2657.67
ASDR	Republic of Moldova	CD	2021	29.37	44.65	18.18
ASPR	Republic of Moldova	CD	1990	1195.09	1445.83	972.90
ASIR	Republic of Moldova	CD	1990	3270.33	4035.58	2685.02
ASDR	Republic of Moldova	CD	1990	29.51	44.69	18.38
ASPR	Romania	CD	2021	1268.15	1543.07	1041.13
ASIR	Romania	CD	2021	3445.31	4252.11	2807.37
ASDR	Romania	CD	2021	31.37	47.31	19.51
ASPR	Romania	CD	1990	1274.64	1550.39	1046.01
ASIR	Romania	CD	1990	3462.73	4273.22	2820.06
ASDR	Romania	CD	1990	31.43	47.69	19.69
ASPR	Russian Federation	CD	2021	1126.40	1302.98	963.47
ASIR	Russian Federation	CD	2021	3091.56	3693.25	2582.75
ASDR	Russian Federation	CD	2021	27.78	40.49	17.86
ASPR	Russian Federation	CD	1990	1157.24	1352.76	982.89
ASIR	Russian Federation	CD	1990	3176.62	3820.91	2640.04
ASDR	Russian Federation	CD	1990	28.53	41.47	18.43
ASPR	Rwanda	CD	2021	896.92	1090.52	720.47
ASIR	Rwanda	CD	2021	2485.87	3101.51	1998.86
ASDR	Rwanda	CD	2021	22.08	33.27	13.62
ASPR	Rwanda	CD	1990	895.74	1089.47	719.39
ASIR	Rwanda	CD	1990	2482.48	3098.22	1995.62
ASDR	Rwanda	CD	1990	21.96	33.26	13.86
ASPR	Saint Kitts and Nevis	CD	2021	1089.88	1333.69	887.26
ASIR	Saint Kitts and Nevis	CD	2021	3002.05	3731.17	2437.70
ASDR	Saint Kitts and Nevis	CD	2021	26.80	40.87	16.30

ASPR	Saint Kitts and Nevis	CD	1990	1092.29	1336.34	889.03
ASIR	Saint Kitts and Nevis	CD	1990	3009.01	3738.74	2443.79
ASDR	Saint Kitts and Nevis	CD	1990	26.90	41.23	16.52
ASPR	Saint Lucia	CD	2021	1089.03	1333.24	886.26
ASIR	Saint Lucia	CD	2021	2999.90	3728.59	2436.81
ASDR	Saint Lucia	CD	2021	26.77	41.00	16.51
ASPR	Saint Lucia	CD	1990	1092.99	1337.56	889.67
ASIR	Saint Lucia	CD	1990	3011.00	3741.82	2445.45
ASDR	Saint Lucia	CD	1990	26.97	40.53	16.78
ASPR	Saint Vincent and the Grenadines	CD	2021	1086.83	1331.49	884.63
ASIR	Saint Vincent and the Grenadines	CD	2021	2993.71	3721.39	2432.14
ASDR	Saint Vincent and the Grenadines	CD	2021	26.73	40.38	16.52
ASPR	Saint Vincent and the Grenadines	CD	1990	1091.28	1335.02	887.95
ASIR	Saint Vincent and the Grenadines	CD	1990	3006.18	3736.82	2441.55
ASDR	Saint Vincent and the Grenadines	CD	1990	26.90	40.48	16.30
ASPR	Samoa	CD	2021	1176.07	1431.55	963.18
ASIR	Samoa	CD	2021	3218.26	4012.03	2607.82
ASDR	Samoa	CD	2021	28.96	44.12	17.75
ASPR	Samoa	CD	1990	1175.95	1430.79	962.82
ASIR	Samoa	CD	1990	3218.04	4011.49	2609.94
ASDR	Samoa	CD	1990	29.09	44.15	18.00
ASPR	San Marino	CD	2021	457.66	559.04	367.65
ASIR	San Marino	CD	2021	1244.55	1549.47	1008.42
ASDR	San Marino	CD	2021	11.42	17.18	6.94
ASPR	San Marino	CD	1990	455.69	557.02	366.25
ASIR	San Marino	CD	1990	1239.11	1544.30	1004.74
ASDR	San Marino	CD	1990	11.37	17.08	6.80
ASPR	Sao Tome and Principe	CD	2021	905.48	1098.64	737.33

ASIR	Sao Tome and Principe	CD	2021	2492.37	3079.34	2026.96
ASDR	Sao Tome and Principe	CD	2021	22.33	33.96	13.78
ASPR	Sao Tome and Principe	CD	1990	907.87	1102.28	739.84
ASIR	Sao Tome and Principe	CD	1990	2499.19	3090.40	2034.27
ASDR	Sao Tome and Principe	CD	1990	22.45	34.40	13.94
ASPR	Saudi Arabia	CD	2021	964.80	1173.51	787.42
ASIR	Saudi Arabia	CD	2021	2670.30	3276.54	2170.42
ASDR	Saudi Arabia	CD	2021	23.56	35.81	14.38
ASPR	Saudi Arabia	CD	1990	965.54	1174.44	788.15
ASIR	Saudi Arabia	CD	1990	2672.23	3277.89	2171.34
ASDR	Saudi Arabia	CD	1990	23.68	35.80	14.40
ASPR	Senegal	CD	2021	906.40	1100.52	738.44
ASIR	Senegal	CD	2021	2495.21	3084.96	2031.34
ASDR	Senegal	CD	2021	22.30	33.65	13.75
ASPR	Senegal	CD	1990	906.00	1100.55	738.14
ASIR	Senegal	CD	1990	2493.77	3083.09	2028.94
ASDR	Senegal	CD	1990	22.23	33.89	13.57
ASPR	Serbia	CD	2021	1264.25	1537.65	1037.74
ASIR	Serbia	CD	2021	3434.94	4240.45	2799.28
ASDR	Serbia	CD	2021	31.20	46.96	19.55
ASPR	Serbia	CD	1990	1270.83	1545.39	1042.97
ASIR	Serbia	CD	1990	3452.27	4258.33	2813.32
ASDR	Serbia	CD	1990	31.41	47.64	19.46
ASPR	Seychelles	CD	2021	1364.44	1699.17	1105.65
ASIR	Seychelles	CD	2021	3657.64	4538.53	2969.43
ASDR	Seychelles	CD	2021	33.88	51.57	20.87
ASPR	Seychelles	CD	1990	1370.82	1708.39	1110.70
ASIR	Seychelles	CD	1990	3675.39	4566.86	2986.46
ASDR	Seychelles	CD	1990	34.17	52.19	20.99
ASPR	Sierra Leone	CD	2021	904.53	1098.17	736.86
ASIR	Sierra Leone	CD	2021	2489.68	3076.72	2024.58
ASDR	Sierra Leone	CD	2021	22.23	34.14	13.63
ASPR	Sierra Leone	CD	1990	904.85	1098.39	737.34
ASIR	Sierra Leone	CD	1990	2490.69	3077.91	2024.70
ASDR	Sierra Leone	CD	1990	22.17	33.98	13.29
ASPR	Singapore	CD	2021	271.95	330.22	219.89
ASIR	Singapore	CD	2021	764.22	937.18	625.43
ASDR	Singapore	CD	2021	6.81	10.52	4.23
ASPR	Singapore	CD	1990	273.30	331.43	221.08

ASIR	Singapore	CD	1990	768.04	942.12	628.35
ASDR	Singapore	CD	1990	6.83	10.54	4.31
ASPR	Slovakia	CD	2021	1268.36	1543.25	1041.30
ASIR	Slovakia	CD	2021	3445.87	4251.83	2807.32
ASDR	Slovakia	CD	2021	31.24	47.01	19.10
ASPR	Slovakia	CD	1990	1277.19	1552.96	1047.93
ASIR	Slovakia	CD	1990	3469.34	4281.57	2825.27
ASDR	Slovakia	CD	1990	31.50	47.18	18.99
ASPR	Slovenia	CD	2021	1262.03	1535.91	1035.98
ASIR	Slovenia	CD	2021	3428.97	4229.25	2795.00
ASDR	Slovenia	CD	2021	31.13	47.76	19.26
ASPR	Slovenia	CD	1990	1274.70	1550.98	1046.27
ASIR	Slovenia	CD	1990	3462.26	4275.32	2821.08
ASDR	Slovenia	CD	1990	31.40	47.45	19.56
ASPR	Solomon Islands	CD	2021	1177.02	1432.73	963.63
ASIR	Solomon Islands	CD	2021	3220.90	4015.26	2610.21
ASDR	Solomon Islands	CD	2021	29.03	44.12	17.69
ASPR	Solomon Islands	CD	1990	1172.19	1426.72	959.65
ASIR	Solomon Islands	CD	1990	3207.12	3996.79	2597.93
ASDR	Solomon Islands	CD	1990	29.03	43.94	17.87
ASPR	Somalia	CD	2021	895.41	1089.92	719.19
ASIR	Somalia	CD	2021	2481.88	3097.89	1995.54
ASDR	Somalia	CD	2021	21.91	33.15	13.62
ASPR	Somalia	CD	1990	892.11	1087.01	716.93
ASIR	Somalia	CD	1990	2472.63	3086.91	1987.64
ASDR	Somalia	CD	1990	21.88	33.47	13.47
ASPR	South Africa	CD	2021	1009.53	1241.83	816.74
ASIR	South Africa	CD	2021	2816.89	3486.83	2260.02
ASDR	South Africa	CD	2021	24.43	36.77	15.18
ASPR	South Africa	CD	1990	1010.19	1242.98	817.35
ASIR	South Africa	CD	1990	2818.70	3489.92	2260.63
ASDR	South Africa	CD	1990	24.69	37.65	15.34
ASPR	South Sudan	CD	2021	891.68	1085.37	717.25
ASIR	South Sudan	CD	2021	2471.07	3084.55	1990.70
ASDR	South Sudan	CD	2021	21.73	32.61	13.46
ASPR	South Sudan	CD	1990	884.77	1079.68	712.37
ASIR	South Sudan	CD	1990	2451.81	3058.68	1976.48
ASDR	South Sudan	CD	1990	21.60	32.69	13.55

ASPR	Spain	CD	2021	455.43	556.33	366.08
ASIR	Spain	CD	2021	1238.40	1542.78	1003.91
ASDR	Spain	CD	2021	11.33	17.14	6.91
ASPR	Spain	CD	1990	455.93	557.00	366.52
ASIR	Spain	CD	1990	1239.82	1543.90	1004.84
ASDR	Spain	CD	1990	11.37	16.97	6.96
ASPR	Sri Lanka	CD	2021	1548.72	1870.43	1274.53
ASIR	Sri Lanka	CD	2021	4002.43	4989.20	3210.06
ASDR	Sri Lanka	CD	2021	38.37	57.81	23.88
ASPR	Sri Lanka	CD	1990	1543.65	1866.25	1271.13
ASIR	Sri Lanka	CD	1990	3988.35	4970.01	3199.11
ASDR	Sri Lanka	CD	1990	38.32	57.64	24.12
ASPR	Sudan	CD	2021	976.85	1188.34	795.62
ASIR	Sudan	CD	2021	2703.81	3316.54	2197.76
ASDR	Sudan	CD	2021	23.98	36.41	14.62
ASPR	Sudan	CD	1990	978.43	1190.15	797.08
ASIR	Sudan	CD	1990	2708.11	3320.74	2200.98
ASDR	Sudan	CD	1990	23.99	36.30	14.68
ASPR	Suriname	CD	2021	1091.15	1335.50	888.12
ASIR	Suriname	CD	2021	3005.93	3735.93	2441.15
ASDR	Suriname	CD	2021	26.75	40.67	16.30
ASPR	Suriname	CD	1990	1088.96	1333.48	885.90
ASIR	Suriname	CD	1990	2999.80	3729.41	2436.57
ASDR	Suriname	CD	1990	26.88	40.55	16.52
ASPR	Sweden	CD	2021	500.39	620.26	402.41
ASIR	Sweden	CD	2021	1379.03	1707.84	1109.77
ASDR	Sweden	CD	2021	12.47	19.47	7.64
ASPR	Sweden	CD	1990	501.19	621.10	403.02
ASIR	Sweden	CD	1990	1381.27	1710.11	1111.61
ASDR	Sweden	CD	1990	12.46	18.91	7.66
ASPR	Switzerland	CD	2021	454.44	554.93	365.34
ASIR	Switzerland	CD	2021	1235.66	1539.22	1001.79
ASDR	Switzerland	CD	2021	11.28	17.08	7.09
ASPR	Switzerland	CD	1990	454.81	555.49	365.67
ASIR	Switzerland	CD	1990	1236.73	1539.69	1002.30
ASDR	Switzerland	CD	1990	11.27	17.21	7.07
ASPR	Syrian Arab Republic	CD	2021	982.79	1195.69	799.21
ASIR	Syrian Arab Republic	CD	2021	2720.05	3337.12	2212.56
ASDR	Syrian Arab Republic	CD	2021	24.04	36.46	14.74
ASPR	Syrian Arab Republic	CD	1990	976.88	1187.69	795.89

ASIR	Syrian Arab Republic	CD	1990	2703.95	3316.21	2197.42
ASDR	Syrian Arab Republic	CD	1990	24.01	36.71	14.87
ASPR	Taiwan (Province of China)	CD	2021	1178.49	1434.53	964.84
ASIR	Taiwan (Province of China)	CD	2021	3225.11	4021.02	2615.05
ASDR	Taiwan (Province of China)	CD	2021	29.37	45.11	18.25
ASPR	Taiwan (Province of China)	CD	1990	1174.48	1429.07	962.08
ASIR	Taiwan (Province of China)	CD	1990	3213.36	4003.06	2605.06
ASDR	Taiwan (Province of China)	CD	1990	29.33	45.00	18.13
ASPR	Tajikistan	CD	2021	1272.38	1547.42	1043.58
ASIR	Tajikistan	CD	2021	3457.27	4264.12	2816.54
ASDR	Tajikistan	CD	2021	31.55	47.86	19.60
ASPR	Tajikistan	CD	1990	1274.76	1550.64	1045.87
ASIR	Tajikistan	CD	1990	3463.01	4275.44	2821.58
ASDR	Tajikistan	CD	1990	31.65	48.08	19.64
ASPR	Thailand	CD	2021	1372.41	1711.00	1111.99
ASIR	Thailand	CD	2021	3679.76	4576.64	2988.97
ASDR	Thailand	CD	2021	34.14	51.67	21.27
ASPR	Thailand	CD	1990	1371.75	1709.96	1111.47
ASIR	Thailand	CD	1990	3677.89	4573.66	2987.11
ASDR	Thailand	CD	1990	33.97	52.06	20.93
ASPR	Timor-Leste	CD	2021	1368.52	1705.89	1108.26
ASIR	Timor-Leste	CD	2021	3668.82	4562.06	2976.88
ASDR	Timor-Leste	CD	2021	33.94	51.75	20.79
ASPR	Timor-Leste	CD	1990	1366.44	1702.84	1106.30
ASIR	Timor-Leste	CD	1990	3663.32	4552.69	2972.31
ASDR	Timor-Leste	CD	1990	33.73	51.60	20.80
ASPR	Togo	CD	2021	910.12	1105.13	741.27
ASIR	Togo	CD	2021	2505.68	3096.98	2039.27
ASDR	Togo	CD	2021	22.45	34.41	13.69
ASPR	Togo	CD	1990	908.47	1103.90	740.27

ASIR	Togo	CD	1990	2500.90	3092.71	2036.25
ASDR	Togo	CD	1990	22.29	33.67	13.92
ASPR	Tokelau	CD	2021	1177.39	1433.04	963.81
ASIR	Tokelau	CD	2021	3222.22	4017.38	2612.23
ASDR	Tokelau	CD	2021	29.10	44.50	17.59
ASPR	Tokelau	CD	1990	1180.61	1436.96	966.76
ASIR	Tokelau	CD	1990	3230.84	4027.01	2621.65
ASDR	Tokelau	CD	1990	29.25	44.91	18.50
ASPR	Tonga	CD	2021	1180.80	1437.35	967.45
ASIR	Tonga	CD	2021	3231.40	4028.15	2619.52
ASDR	Tonga	CD	2021	29.19	44.43	18.07
ASPR	Tonga	CD	1990	1179.84	1435.77	966.11
ASIR	Tonga	CD	1990	3228.63	4024.95	2618.10
ASDR	Tonga	CD	1990	29.29	44.79	17.95
ASPR	Trinidad and Tobago	CD	2021	1088.89	1333.03	886.15
ASIR	Trinidad and Tobago	CD	2021	2999.42	3728.07	2436.04
ASDR	Trinidad and Tobago	CD	2021	26.74	41.08	16.32
ASPR	Trinidad and Tobago	CD	1990	1089.61	1334.03	886.83
ASIR	Trinidad and Tobago	CD	1990	3001.45	3730.37	2437.70
ASDR	Trinidad and Tobago	CD	1990	26.91	41.13	16.60
ASPR	Tunisia	CD	2021	980.75	1192.06	798.47
ASIR	Tunisia	CD	2021	2714.90	3328.41	2204.85
ASDR	Tunisia	CD	2021	23.99	36.30	14.85
ASPR	Tunisia	CD	1990	978.39	1189.46	797.12
ASIR	Tunisia	CD	1990	2708.21	3320.95	2201.11
ASDR	Tunisia	CD	1990	24.08	36.72	14.91
ASPR	Turkey	CD	2021	980.51	1191.44	798.34
ASIR	Turkey	CD	2021	2714.25	3327.37	2204.11
ASDR	Turkey	CD	2021	24.15	36.18	14.85
ASPR	Turkey	CD	1990	980.43	1191.32	798.45
ASIR	Turkey	CD	1990	2714.03	3327.00	2204.15
ASDR	Turkey	CD	1990	24.20	36.36	14.97
ASPR	Turkmenistan	CD	2021	1262.88	1535.74	1036.75
ASIR	Turkmenistan	CD	2021	3430.20	4236.31	2796.61
ASDR	Turkmenistan	CD	2021	31.29	47.80	19.44
ASPR	Turkmenistan	CD	1990	1279.89	1557.72	1050.49
ASIR	Turkmenistan	CD	1990	3477.00	4292.78	2832.04
ASDR	Turkmenistan	CD	1990	31.68	48.34	19.53

ASPR	Tuvalu	CD	2021	1176.26	1432.44	963.39
ASIR	Tuvalu	CD	2021	3219.21	4013.86	2609.65
ASDR	Tuvalu	CD	2021	29.13	44.32	18.16
ASPR	Tuvalu	CD	1990	1186.52	1444.34	972.85
ASIR	Tuvalu	CD	1990	3246.99	4045.27	2634.67
ASDR	Tuvalu	CD	1990	29.42	45.28	18.30
ASPR	Uganda	CD	2021	896.33	1089.73	720.11
ASIR	Uganda	CD	2021	2484.21	3099.39	1997.62
ASDR	Uganda	CD	2021	21.99	33.00	13.56
ASPR	Uganda	CD	1990	892.97	1086.48	717.70
ASIR	Uganda	CD	1990	2474.68	3087.97	1990.95
ASDR	Uganda	CD	1990	21.80	32.98	13.67
ASPR	Ukraine	CD	2021	1304.96	1598.94	1065.21
ASIR	Ukraine	CD	2021	3583.40	4440.17	2921.75
ASDR	Ukraine	CD	2021	32.06	48.08	20.24
ASPR	Ukraine	CD	1990	1312.06	1608.33	1071.15
ASIR	Ukraine	CD	1990	3602.80	4467.20	2936.47
ASDR	Ukraine	CD	1990	32.21	48.51	20.22
ASPR	United Arab Emirates	CD	2021	945.60	1152.68	771.10
ASIR	United Arab Emirates	CD	2021	2615.94	3222.84	2128.76
ASDR	United Arab Emirates	CD	2021	23.23	35.45	14.55
ASPR	United Arab Emirates	CD	1990	955.05	1161.37	778.66
ASIR	United Arab Emirates	CD	1990	2642.97	3245.46	2146.52
ASDR	United Arab Emirates	CD	1990	23.54	35.55	14.14
ASPR	United Kingdom	CD	2021	496.04	614.77	399.03
ASIR	United Kingdom	CD	2021	1364.15	1690.39	1100.93
ASDR	United Kingdom	CD	2021	12.29	18.78	7.59
ASPR	United Kingdom	CD	1990	494.92	611.57	399.21
ASIR	United Kingdom	CD	1990	1361.14	1684.25	1100.36
ASDR	United Kingdom	CD	1990	12.29	18.77	7.58
ASPR	United Republic of	CD	2021	894.26	1087.65	718.53

	Tanzania					
ASIR	United Republic of Tanzania	CD	2021	2478.23	3092.14	1994.26
ASDR	United Republic of Tanzania	CD	2021	21.99	33.32	13.82
ASPR	United Republic of Tanzania	CD	1990	894.01	1087.38	718.48
ASIR	United Republic of Tanzania	CD	1990	2477.48	3091.44	1993.84
ASDR	United Republic of Tanzania	CD	1990	21.81	33.06	13.87
ASPR	United States of America	CD	2021	1394.44	1637.79	1183.09
ASIR	United States of America	CD	2021	3513.50	4288.59	2894.27
ASDR	United States of America	CD	2021	34.14	49.82	21.67
ASPR	United States of America	CD	1990	2020.34	2399.95	1680.66
ASIR	United States of America	CD	1990	4837.64	5962.69	3953.18
ASDR	United States of America	CD	1990	49.92	73.54	31.36
ASPR	United States Virgin Islands	CD	2021	1091.09	1335.91	888.30
ASIR	United States Virgin Islands	CD	2021	3005.69	3735.94	2441.39
ASDR	United States Virgin Islands	CD	2021	26.84	40.84	16.37
ASPR	United States Virgin Islands	CD	1990	1092.78	1338.00	890.01
ASIR	United States Virgin Islands	CD	1990	3010.50	3741.52	2445.42
ASDR	United States Virgin Islands	CD	1990	27.04	41.15	16.57
ASPR	Uruguay	CD	2021	274.20	332.59	221.66
ASIR	Uruguay	CD	2021	770.60	944.97	630.20
ASDR	Uruguay	CD	2021	6.79	10.36	4.25

ASPR	Uruguay	CD	1990	274.06	332.44	221.56
ASIR	Uruguay	CD	1990	770.20	944.46	629.92
ASDR	Uruguay	CD	1990	6.79	10.17	4.21
ASPR	Uzbekistan	CD	2021	1275.62	1551.41	1046.43
ASIR	Uzbekistan	CD	2021	3465.77	4275.32	2822.26
ASDR	Uzbekistan	CD	2021	31.49	48.10	19.77
ASPR	Uzbekistan	CD	1990	1278.16	1555.43	1048.91
ASIR	Uzbekistan	CD	1990	3471.84	4286.69	2828.62
ASDR	Uzbekistan	CD	1990	31.57	47.73	19.74
ASPR	Vanuatu	CD	2021	1178.33	1434.69	964.81
ASIR	Vanuatu	CD	2021	3224.48	4019.03	2613.00
ASDR	Vanuatu	CD	2021	29.20	44.65	17.95
ASPR	Vanuatu	CD	1990	1173.77	1429.12	961.25
ASIR	Vanuatu	CD	1990	3211.58	4002.09	2600.99
ASDR	Vanuatu	CD	1990	29.03	44.90	18.06
ASPR	Venezuela (Bolivarian Republic of)	CD	2021	1092.47	1337.84	889.70
ASIR	Venezuela (Bolivarian Republic of)	CD	2021	3009.49	3740.40	2444.19
ASDR	Venezuela (Bolivarian Republic of)	CD	2021	26.94	40.83	16.66
ASPR	Venezuela (Bolivarian Republic of)	CD	1990	1090.35	1334.91	887.35
ASIR	Venezuela (Bolivarian Republic of)	CD	1990	3003.54	3733.39	2439.49
ASDR	Venezuela (Bolivarian Republic of)	CD	1990	26.97	40.69	16.30
ASPR	Viet Nam	CD	2021	1371.76	1709.37	1111.55
ASIR	Viet Nam	CD	2021	3678.17	4571.32	2988.21
ASDR	Viet Nam	CD	2021	34.24	52.24	20.68
ASPR	Viet Nam	CD	1990	1376.48	1715.95	1115.78
ASIR	Viet Nam	CD	1990	3691.04	4593.06	3000.82
ASDR	Viet Nam	CD	1990	34.24	51.71	20.92
ASPR	Yemen	CD	2021	979.90	1190.69	797.70
ASIR	Yemen	CD	2021	2712.55	3326.47	2203.48
ASDR	Yemen	CD	2021	23.90	36.07	14.60
ASPR	Yemen	CD	1990	980.80	1192.72	798.83
ASIR	Yemen	CD	1990	2714.83	3326.95	2205.07

ASDR	Yemen	CD	1990	23.92	36.51	14.73
ASPR	Zambia	CD	2021	892.84	1086.29	717.79
ASIR	Zambia	CD	2021	2474.41	3087.68	1990.78
ASDR	Zambia	CD	2021	21.89	33.22	13.75
ASPR	Zambia	CD	1990	891.11	1084.89	716.81
ASIR	Zambia	CD	1990	2469.35	3082.16	1987.82
ASDR	Zambia	CD	1990	21.85	33.07	13.65
ASPR	Zimbabwe	CD	2021	897.60	1090.60	721.29
ASIR	Zimbabwe	CD	2021	2487.77	3105.75	2001.30
ASDR	Zimbabwe	CD	2021	21.95	33.37	13.74
ASPR	Zimbabwe	CD	1990	893.65	1087.18	718.40
ASIR	Zimbabwe	CD	1990	2476.54	3088.20	1994.16
ASDR	Zimbabwe	CD	1990	21.98	33.51	13.51
ASPR	Afghanistan	Urticaria	2021	909.66	1038.05	799.00
ASIR	Afghanistan	Urticaria	2021	1604.97	1808.89	1417.94
ASDR	Afghanistan	Urticaria	2021	54.09	75.97	35.85
ASPR	Afghanistan	Urticaria	1990	918.72	1047.09	806.69
ASIR	Afghanistan	Urticaria	1990	1620.65	1821.93	1432.72
ASDR	Afghanistan	Urticaria	1990	54.53	76.64	36.17
ASPR	Albania	Urticaria	2021	1083.48	1248.42	956.46
ASIR	Albania	Urticaria	2021	1906.71	2169.98	1671.59
ASDR	Albania	Urticaria	2021	65.60	94.39	43.73
ASPR	Albania	Urticaria	1990	1078.73	1243.94	951.42
ASIR	Albania	Urticaria	1990	1898.33	2161.22	1664.43
ASDR	Albania	Urticaria	1990	65.18	92.33	43.35
ASPR	Algeria	Urticaria	2021	908.08	1036.59	797.46
ASIR	Algeria	Urticaria	2021	1602.25	1805.68	1415.58
ASDR	Algeria	Urticaria	2021	54.45	77.26	35.69
ASPR	Algeria	Urticaria	1990	909.46	1038.19	798.73
ASIR	Algeria	Urticaria	1990	1604.66	1808.67	1417.63
ASDR	Algeria	Urticaria	1990	54.53	76.99	36.13
ASPR	American Samoa	Urticaria	2021	717.67	821.89	634.81
ASIR	American Samoa	Urticaria	2021	1269.13	1449.99	1112.84
ASDR	American Samoa	Urticaria	2021	43.06	62.33	28.15
ASPR	American Samoa	Urticaria	1990	717.35	821.43	634.64
ASIR	American Samoa	Urticaria	1990	1268.55	1449.01	1112.11
ASDR	American Samoa	Urticaria	1990	43.16	61.16	28.01
ASPR	Andorra	Urticaria	2021	578.97	649.64	511.47

ASIR	Andorra	Urticaria	2021	1024.24	1141.17	911.92
ASDR	Andorra	Urticaria	2021	34.45	47.97	22.32
ASPR	Andorra	Urticaria	1990	571.83	641.40	505.52
ASIR	Andorra	Urticaria	1990	1011.66	1127.62	900.86
ASDR	Andorra	Urticaria	1990	34.11	47.47	22.29
ASPR	Angola	Urticaria	2021	813.10	931.44	719.01
ASIR	Angola	Urticaria	2021	1434.88	1627.17	1260.79
ASDR	Angola	Urticaria	2021	48.75	69.78	32.07
ASPR	Angola	Urticaria	1990	807.55	925.03	714.62
ASIR	Angola	Urticaria	1990	1425.18	1616.72	1251.32
ASDR	Angola	Urticaria	1990	48.11	68.79	31.82
ASPR	Antigua and Barbuda	Urticaria	2021	809.22	927.02	715.97
ASIR	Antigua and Barbuda	Urticaria	2021	1428.15	1619.82	1254.60
ASDR	Antigua and Barbuda	Urticaria	2021	48.63	69.43	32.04
ASPR	Antigua and Barbuda	Urticaria	1990	811.62	929.71	717.88
ASIR	Antigua and Barbuda	Urticaria	1990	1432.30	1624.39	1258.41
ASDR	Antigua and Barbuda	Urticaria	1990	48.82	68.54	31.68
ASPR	Argentina	Urticaria	2021	773.95	879.61	684.02
ASIR	Argentina	Urticaria	2021	1364.87	1544.37	1205.78
ASDR	Argentina	Urticaria	2021	46.48	66.72	30.60
ASPR	Argentina	Urticaria	1990	774.51	880.04	684.56
ASIR	Argentina	Urticaria	1990	1365.93	1545.62	1206.75
ASDR	Argentina	Urticaria	1990	46.43	66.03	30.49
ASPR	Armenia	Urticaria	2021	1053.02	1209.49	927.51
ASIR	Armenia	Urticaria	2021	1853.88	2121.41	1628.05
ASDR	Armenia	Urticaria	2021	63.56	90.50	41.95
ASPR	Armenia	Urticaria	1990	1054.86	1211.15	929.30
ASIR	Armenia	Urticaria	1990	1857.22	2126.13	1630.71
ASDR	Armenia	Urticaria	1990	63.54	90.42	42.21
ASPR	Australia	Urticaria	2021	916.77	1034.09	801.75
ASIR	Australia	Urticaria	2021	1611.17	1819.37	1411.58
ASDR	Australia	Urticaria	2021	54.91	77.17	36.08
ASPR	Australia	Urticaria	1990	915.38	1032.75	800.69
ASIR	Australia	Urticaria	1990	1608.77	1815.97	1409.35
ASDR	Australia	Urticaria	1990	54.83	77.64	36.17
ASPR	Austria	Urticaria	2021	583.96	655.04	515.69
ASIR	Austria	Urticaria	2021	1032.84	1150.56	919.53
ASDR	Austria	Urticaria	2021	34.73	48.25	22.62

ASPR	Austria	Urticaria	1990	588.48	659.62	519.48
ASIR	Austria	Urticaria	1990	1040.67	1158.75	927.15
ASDR	Austria	Urticaria	1990	34.97	48.88	23.37
ASPR	Azerbaijan	Urticaria	2021	1048.31	1204.18	923.10
ASIR	Azerbaijan	Urticaria	2021	1845.60	2112.35	1620.10
ASDR	Azerbaijan	Urticaria	2021	63.36	90.76	41.78
ASPR	Azerbaijan	Urticaria	1990	1056.82	1213.20	931.02
ASIR	Azerbaijan	Urticaria	1990	1860.56	2129.86	1633.87
ASDR	Azerbaijan	Urticaria	1990	63.68	89.98	42.00
ASPR	Bahamas	Urticaria	2021	811.03	929.01	717.38
ASIR	Bahamas	Urticaria	2021	1431.26	1623.26	1257.45
ASDR	Bahamas	Urticaria	2021	48.76	69.42	31.63
ASPR	Bahamas	Urticaria	1990	810.81	928.69	717.22
ASIR	Bahamas	Urticaria	1990	1430.90	1622.86	1257.30
ASDR	Bahamas	Urticaria	1990	48.81	69.25	32.07
ASPR	Bahrain	Urticaria	2021	882.10	1010.39	774.19
ASIR	Bahrain	Urticaria	2021	1557.16	1764.19	1373.36
ASDR	Bahrain	Urticaria	2021	53.09	75.65	35.09
ASPR	Bahrain	Urticaria	1990	891.90	1020.67	783.09
ASIR	Bahrain	Urticaria	1990	1574.09	1780.98	1388.78
ASDR	Bahrain	Urticaria	1990	53.53	75.49	35.37
ASPR	Bangladesh	Urticaria	2021	1140.70	1299.71	1007.53
ASIR	Bangladesh	Urticaria	2021	2011.38	2278.57	1780.58
ASDR	Bangladesh	Urticaria	2021	68.61	97.19	45.24
ASPR	Bangladesh	Urticaria	1990	1129.93	1287.06	997.82
ASIR	Bangladesh	Urticaria	1990	1992.75	2257.77	1763.43
ASDR	Bangladesh	Urticaria	1990	67.44	94.87	44.78
ASPR	Barbados	Urticaria	2021	809.35	927.04	716.09
ASIR	Barbados	Urticaria	2021	1428.39	1620.16	1255.05
ASDR	Barbados	Urticaria	2021	48.69	69.08	32.50
ASPR	Barbados	Urticaria	1990	811.29	929.23	717.60
ASIR	Barbados	Urticaria	1990	1431.77	1623.80	1258.23
ASDR	Barbados	Urticaria	1990	48.84	70.05	31.94
ASPR	Belarus	Urticaria	2021	1053.99	1210.19	928.84
ASIR	Belarus	Urticaria	2021	1855.62	2124.44	1629.56
ASDR	Belarus	Urticaria	2021	63.73	90.74	41.98
ASPR	Belarus	Urticaria	1990	1058.06	1214.54	932.57
ASIR	Belarus	Urticaria	1990	1862.74	2133.11	1636.19
ASDR	Belarus	Urticaria	1990	63.90	91.13	42.43
ASPR	Belgium	Urticaria	2021	585.40	656.68	516.93
ASIR	Belgium	Urticaria	2021	1035.51	1153.16	921.81
ASDR	Belgium	Urticaria	2021	34.80	48.84	23.01
ASPR	Belgium	Urticaria	1990	585.72	656.76	517.21
ASIR	Belgium	Urticaria	1990	1035.94	1153.69	922.48

ASDR	Belgium	Urticaria	1990	34.89	48.60	22.91
ASPR	Belize	Urticaria	2021	808.71	926.54	715.58
ASIR	Belize	Urticaria	2021	1427.26	1618.87	1253.39
ASDR	Belize	Urticaria	2021	48.61	70.67	32.12
ASPR	Belize	Urticaria	1990	806.60	923.90	713.95
ASIR	Belize	Urticaria	1990	1423.54	1614.92	1249.92
ASDR	Belize	Urticaria	1990	48.49	68.88	31.93
ASPR	Benin	Urticaria	2021	810.69	928.68	717.22
ASIR	Benin	Urticaria	2021	1430.65	1622.52	1256.87
ASDR	Benin	Urticaria	2021	48.54	69.78	31.75
ASPR	Benin	Urticaria	1990	812.01	930.09	718.27
ASIR	Benin	Urticaria	1990	1433.08	1625.37	1259.39
ASDR	Benin	Urticaria	1990	48.34	68.02	31.60
ASPR	Bermuda	Urticaria	2021	808.92	926.48	715.76
ASIR	Bermuda	Urticaria	2021	1427.58	1619.35	1254.01
ASDR	Bermuda	Urticaria	2021	48.86	69.31	32.11
ASPR	Bermuda	Urticaria	1990	810.22	927.99	716.70
ASIR	Bermuda	Urticaria	1990	1429.79	1621.71	1256.19
ASDR	Bermuda	Urticaria	1990	48.88	69.61	32.31
ASPR	Bhutan	Urticaria	2021	1132.06	1289.05	999.40
ASIR	Bhutan	Urticaria	2021	1996.36	2261.61	1767.02
ASDR	Bhutan	Urticaria	2021	68.08	96.84	45.55
ASPR	Bhutan	Urticaria	1990	1127.55	1283.69	995.23
ASIR	Bhutan	Urticaria	1990	1988.76	2251.15	1760.31
ASDR	Bhutan	Urticaria	1990	67.31	95.15	44.74
ASPR	Bolivia (Plurinational State of)	Urticaria	2021	808.14	925.63	715.16
ASIR	Bolivia (Plurinational State of)	Urticaria	2021	1426.26	1617.86	1252.78
ASDR	Bolivia (Plurinational State of)	Urticaria	2021	48.44	68.95	31.87
ASPR	Bolivia (Plurinational State of)	Urticaria	1990	809.99	927.84	716.65
ASIR	Bolivia (Plurinational State of)	Urticaria	1990	1429.46	1621.31	1255.79
ASDR	Bolivia (Plurinational State of)	Urticaria	1990	48.43	68.73	31.53

ASPR	Bosnia and Herzegovina	Urticaria	2021	1086.38	1252.09	958.98
ASIR	Bosnia and Herzegovina	Urticaria	2021	1911.70	2175.87	1676.70
ASDR	Bosnia and Herzegovina	Urticaria	2021	65.60	94.86	42.66
ASPR	Bosnia and Herzegovina	Urticaria	1990	1087.99	1253.39	960.39
ASIR	Bosnia and Herzegovina	Urticaria	1990	1914.51	2179.47	1679.57
ASDR	Bosnia and Herzegovina	Urticaria	1990	65.60	93.48	43.33
ASPR	Botswana	Urticaria	2021	810.23	927.85	716.83
ASIR	Botswana	Urticaria	2021	1429.89	1621.85	1256.37
ASDR	Botswana	Urticaria	2021	48.37	69.08	31.62
ASPR	Botswana	Urticaria	1990	814.19	932.74	719.90
ASIR	Botswana	Urticaria	1990	1436.74	1629.23	1262.50
ASDR	Botswana	Urticaria	1990	48.77	69.07	32.31
ASPR	Brazil	Urticaria	2021	854.40	974.78	753.51
ASIR	Brazil	Urticaria	2021	1508.41	1710.95	1329.26
ASDR	Brazil	Urticaria	2021	51.06	72.08	33.56
ASPR	Brazil	Urticaria	1990	854.71	975.31	753.60
ASIR	Brazil	Urticaria	1990	1508.96	1711.72	1329.48
ASDR	Brazil	Urticaria	1990	50.99	72.79	33.72
ASPR	Brunei Darussalam	Urticaria	2021	758.56	862.17	670.51
ASIR	Brunei Darussalam	Urticaria	2021	1337.79	1514.83	1180.97
ASDR	Brunei Darussalam	Urticaria	2021	45.86	64.83	30.17
ASPR	Brunei Darussalam	Urticaria	1990	755.80	858.83	668.12
ASIR	Brunei Darussalam	Urticaria	1990	1333.19	1511.61	1176.74
ASDR	Brunei Darussalam	Urticaria	1990	45.65	64.98	30.15
ASPR	Bulgaria	Urticaria	2021	1084.96	1250.39	957.45
ASIR	Bulgaria	Urticaria	2021	1909.17	2173.59	1674.48
ASDR	Bulgaria	Urticaria	2021	65.59	93.85	43.44
ASPR	Bulgaria	Urticaria	1990	1088.23	1254.22	960.69
ASIR	Bulgaria	Urticaria	1990	1915.02	2179.45	1679.73
ASDR	Bulgaria	Urticaria	1990	65.81	93.65	43.68
ASPR	Burkina Faso	Urticaria	2021	812.34	930.65	718.56
ASIR	Burkina Faso	Urticaria	2021	1433.55	1625.80	1259.46

ASDR	Burkina Faso	Urticaria	2021	48.51	69.00	31.72
ASPR	Burkina Faso	Urticaria	1990	813.23	931.60	719.13
ASIR	Burkina Faso	Urticaria	1990	1435.17	1627.57	1261.17
ASDR	Burkina Faso	Urticaria	1990	48.21	68.64	31.66
ASPR	Burundi	Urticaria	2021	807.43	924.95	714.64
ASIR	Burundi	Urticaria	2021	1424.95	1616.45	1251.06
ASDR	Burundi	Urticaria	2021	48.39	69.20	31.86
ASPR	Burundi	Urticaria	1990	812.65	931.02	718.79
ASIR	Burundi	Urticaria	1990	1434.12	1626.30	1260.02
ASDR	Burundi	Urticaria	1990	48.48	69.98	32.05
ASPR	Côte d'Ivoire	Urticaria	2021	804.07	920.88	711.59
ASIR	Côte d'Ivoire	Urticaria	2021	1419.19	1610.47	1245.94
ASDR	Côte d'Ivoire	Urticaria	2021	48.15	68.67	31.52
ASPR	Côte d'Ivoire	Urticaria	1990	804.52	921.56	712.00
ASIR	Côte d'Ivoire	Urticaria	1990	1419.89	1611.00	1246.68
ASDR	Côte d'Ivoire	Urticaria	1990	47.66	68.17	31.12
ASPR	Cabo Verde	Urticaria	2021	807.73	924.83	714.81
ASIR	Cabo Verde	Urticaria	2021	1425.48	1617.09	1252.28
ASDR	Cabo Verde	Urticaria	2021	48.64	68.57	31.68
ASPR	Cabo Verde	Urticaria	1990	816.77	935.42	721.78
ASIR	Cabo Verde	Urticaria	1990	1441.32	1633.80	1267.37
ASDR	Cabo Verde	Urticaria	1990	49.08	69.48	32.38
ASPR	Cambodia	Urticaria	2021	748.71	855.00	650.52
ASIR	Cambodia	Urticaria	2021	1325.88	1521.61	1158.77
ASDR	Cambodia	Urticaria	2021	44.99	65.04	29.51
ASPR	Cambodia	Urticaria	1990	745.64	852.16	648.19
ASIR	Cambodia	Urticaria	1990	1320.30	1516.04	1152.90
ASDR	Cambodia	Urticaria	1990	44.48	63.22	29.32
ASPR	Cameroon	Urticaria	2021	808.67	926.29	715.62
ASIR	Cameroon	Urticaria	2021	1427.16	1618.87	1253.60
ASDR	Cameroon	Urticaria	2021	48.53	69.00	31.94
ASPR	Cameroon	Urticaria	1990	810.09	928.09	716.82
ASIR	Cameroon	Urticaria	1990	1429.67	1621.59	1255.96
ASDR	Cameroon	Urticaria	1990	48.31	69.86	31.59
ASPR	Canada	Urticaria	2021	935.63	1068.96	819.77
ASIR	Canada	Urticaria	2021	1645.56	1862.62	1447.84
ASDR	Canada	Urticaria	2021	56.42	80.62	37.41
ASPR	Canada	Urticaria	1990	936.76	1069.99	821.05
ASIR	Canada	Urticaria	1990	1647.54	1864.57	1449.66
ASDR	Canada	Urticaria	1990	56.59	79.99	36.94
ASPR	Central African Republic	Urticaria	2021	811.54	929.45	717.74

ASIR	Central African Republic	Urticaria	2021	1432.14	1624.39	1258.39
ASDR	Central African Republic	Urticaria	2021	48.17	68.79	31.77
ASPR	Central African Republic	Urticaria	1990	811.16	929.23	717.62
ASIR	Central African Republic	Urticaria	1990	1431.51	1623.34	1257.89
ASDR	Central African Republic	Urticaria	1990	47.93	67.60	32.14
ASPR	Chad	Urticaria	2021	808.29	926.24	715.04
ASIR	Chad	Urticaria	2021	1426.48	1618.09	1252.45
ASDR	Chad	Urticaria	2021	48.35	69.65	31.93
ASPR	Chad	Urticaria	1990	812.54	931.08	718.68
ASIR	Chad	Urticaria	1990	1433.87	1626.05	1259.66
ASDR	Chad	Urticaria	1990	48.34	69.45	31.75
ASPR	Chile	Urticaria	2021	771.02	876.48	681.42
ASIR	Chile	Urticaria	2021	1359.70	1538.65	1200.94
ASDR	Chile	Urticaria	2021	46.31	66.20	30.00
ASPR	Chile	Urticaria	1990	775.59	881.46	685.59
ASIR	Chile	Urticaria	1990	1367.76	1547.62	1208.44
ASDR	Chile	Urticaria	1990	46.55	65.59	30.32
ASPR	China	Urticaria	2021	756.14	857.19	666.91
ASIR	China	Urticaria	2021	1337.61	1514.69	1172.35
ASDR	China	Urticaria	2021	45.81	65.41	30.13
ASPR	China	Urticaria	1990	758.89	860.64	669.20
ASIR	China	Urticaria	1990	1342.35	1520.53	1176.00
ASDR	China	Urticaria	1990	45.84	65.56	30.28
ASPR	Colombia	Urticaria	2021	809.03	926.66	715.83
ASIR	Colombia	Urticaria	2021	1427.81	1619.48	1254.51
ASDR	Colombia	Urticaria	2021	48.81	69.85	31.94
ASPR	Colombia	Urticaria	1990	809.36	927.14	716.18
ASIR	Colombia	Urticaria	1990	1428.37	1620.17	1254.66
ASDR	Colombia	Urticaria	1990	48.68	68.72	32.01
ASPR	Comoros	Urticaria	2021	808.57	926.04	715.48
ASIR	Comoros	Urticaria	2021	1426.98	1618.74	1253.50
ASDR	Comoros	Urticaria	2021	48.60	68.66	32.09
ASPR	Comoros	Urticaria	1990	809.70	927.65	716.39
ASIR	Comoros	Urticaria	1990	1428.95	1620.81	1255.10

ASDR	Comoros	Urticaria	1990	48.50	68.90	31.64
ASPR	Congo	Urticaria	2021	809.12	926.79	715.87
ASIR	Congo	Urticaria	2021	1427.96	1619.79	1254.08
ASDR	Congo	Urticaria	2021	48.32	69.76	31.98
ASPR	Congo	Urticaria	1990	812.13	930.22	718.25
ASIR	Congo	Urticaria	1990	1433.17	1625.29	1259.18
ASDR	Congo	Urticaria	1990	48.41	68.28	31.85
ASPR	Cook Islands	Urticaria	2021	720.34	824.58	637.05
ASIR	Cook Islands	Urticaria	2021	1273.65	1454.79	1117.11
ASDR	Cook Islands	Urticaria	2021	43.39	61.83	28.30
ASPR	Cook Islands	Urticaria	1990	716.02	820.14	633.29
ASIR	Cook Islands	Urticaria	1990	1266.16	1446.90	1109.96
ASDR	Cook Islands	Urticaria	1990	43.15	63.30	28.72
ASPR	Costa Rica	Urticaria	2021	810.54	928.49	717.04
ASIR	Costa Rica	Urticaria	2021	1430.46	1622.40	1256.85
ASDR	Costa Rica	Urticaria	2021	48.79	69.67	32.37
ASPR	Costa Rica	Urticaria	1990	808.03	925.55	715.10
ASIR	Costa Rica	Urticaria	1990	1426.07	1617.71	1252.61
ASDR	Costa Rica	Urticaria	1990	48.71	68.73	32.65
ASPR	Croatia	Urticaria	2021	1085.88	1251.57	958.44
ASIR	Croatia	Urticaria	2021	1910.82	2175.08	1675.93
ASDR	Croatia	Urticaria	2021	65.67	93.21	42.87
ASPR	Croatia	Urticaria	1990	1090.36	1256.15	962.58
ASIR	Croatia	Urticaria	1990	1918.53	2183.47	1683.47
ASDR	Croatia	Urticaria	1990	65.92	93.69	44.00
ASPR	Cuba	Urticaria	2021	806.40	923.55	713.66
ASIR	Cuba	Urticaria	2021	1423.17	1614.52	1250.10
ASDR	Cuba	Urticaria	2021	48.59	69.78	32.06
ASPR	Cuba	Urticaria	1990	806.93	924.33	714.05
ASIR	Cuba	Urticaria	1990	1424.10	1615.56	1250.77
ASDR	Cuba	Urticaria	1990	48.67	68.67	32.53
ASPR	Cyprus	Urticaria	2021	588.13	660.18	519.15
ASIR	Cyprus	Urticaria	2021	1040.12	1159.32	926.00
ASDR	Cyprus	Urticaria	2021	35.07	49.10	23.05
ASPR	Cyprus	Urticaria	1990	585.25	656.52	516.84
ASIR	Cyprus	Urticaria	1990	1035.09	1153.42	921.38
ASDR	Cyprus	Urticaria	1990	34.85	48.08	23.41
ASPR	Czechia	Urticaria	2021	1083.87	1249.56	956.33
ASIR	Czechia	Urticaria	2021	1907.34	2171.68	1672.57
ASDR	Czechia	Urticaria	2021	65.54	94.30	43.41
ASPR	Czechia	Urticaria	1990	1090.19	1255.99	962.51
ASIR	Czechia	Urticaria	1990	1918.31	2183.26	1683.13
ASDR	Czechia	Urticaria	1990	65.84	93.10	43.72

ASPR	Democratic People's Republic of Korea	Urticaria	2021	727.70	829.57	640.47
ASIR	Democratic People's Republic of Korea	Urticaria	2021	1287.12	1458.76	1131.89
ASDR	Democratic People's Republic of Korea	Urticaria	2021	44.01	62.89	29.00
ASPR	Democratic People's Republic of Korea	Urticaria	1990	737.44	840.53	648.96
ASIR	Democratic People's Republic of Korea	Urticaria	1990	1304.02	1477.25	1146.94
ASDR	Democratic People's Republic of Korea	Urticaria	1990	44.54	64.03	28.69
ASPR	Democratic Republic of the Congo	Urticaria	2021	808.35	925.70	715.28
ASIR	Democratic Republic of the Congo	Urticaria	2021	1426.60	1618.22	1253.20
ASDR	Democratic Republic of the Congo	Urticaria	2021	48.20	68.79	31.58
ASPR	Democratic Republic of the Congo	Urticaria	1990	810.76	928.86	717.19
ASIR	Democratic Republic of the Congo	Urticaria	1990	1430.80	1622.78	1256.97
ASDR	Democratic Republic of the Congo	Urticaria	1990	47.96	67.84	31.90
ASPR	Denmark	Urticaria	2021	679.45	760.50	601.45
ASIR	Denmark	Urticaria	2021	1189.17	1320.81	1054.98

ASDR	Denmark	Urticaria	2021	40.52	55.92	26.86
ASPR	Denmark	Urticaria	1990	681.00	761.95	603.08
ASIR	Denmark	Urticaria	1990	1191.77	1323.51	1057.35
ASDR	Denmark	Urticaria	1990	40.51	56.46	27.07
ASPR	Djibouti	Urticaria	2021	798.73	914.70	706.59
ASIR	Djibouti	Urticaria	2021	1409.91	1600.24	1238.33
ASDR	Djibouti	Urticaria	2021	48.04	67.96	31.26
ASPR	Djibouti	Urticaria	1990	802.10	918.51	709.94
ASIR	Djibouti	Urticaria	1990	1415.66	1606.50	1243.23
ASDR	Djibouti	Urticaria	1990	48.09	68.48	31.24
ASPR	Dominica	Urticaria	2021	806.24	923.43	713.55
ASIR	Dominica	Urticaria	2021	1422.93	1614.13	1249.57
ASDR	Dominica	Urticaria	2021	48.45	69.24	31.82
ASPR	Dominica	Urticaria	1990	808.78	925.91	715.79
ASIR	Dominica	Urticaria	1990	1427.41	1619.02	1254.99
ASDR	Dominica	Urticaria	1990	48.68	70.02	32.35
ASPR	Dominican Republic	Urticaria	2021	807.32	924.68	714.54
ASIR	Dominican Republic	Urticaria	2021	1424.81	1616.32	1251.37
ASDR	Dominican Republic	Urticaria	2021	48.53	69.25	32.31
ASPR	Dominican Republic	Urticaria	1990	810.34	928.38	716.92
ASIR	Dominican Republic	Urticaria	1990	1430.06	1621.95	1256.02
ASDR	Dominican Republic	Urticaria	1990	48.73	70.61	32.07
ASPR	Ecuador	Urticaria	2021	808.47	926.08	715.42
ASIR	Ecuador	Urticaria	2021	1426.84	1618.45	1253.34
ASDR	Ecuador	Urticaria	2021	48.72	69.54	32.52
ASPR	Ecuador	Urticaria	1990	808.80	926.50	715.73
ASIR	Ecuador	Urticaria	1990	1427.41	1619.11	1253.71
ASDR	Ecuador	Urticaria	1990	48.76	69.87	32.06
ASPR	Egypt	Urticaria	2021	1084.30	1224.40	942.15
ASIR	Egypt	Urticaria	2021	1892.39	2130.72	1642.66
ASDR	Egypt	Urticaria	2021	65.18	92.60	42.41
ASPR	Egypt	Urticaria	1990	1085.95	1226.14	943.74
ASIR	Egypt	Urticaria	1990	1895.32	2133.88	1645.68
ASDR	Egypt	Urticaria	1990	65.24	92.63	42.76
ASPR	El Salvador	Urticaria	2021	813.70	931.71	719.48
ASIR	El Salvador	Urticaria	2021	1435.96	1628.25	1262.61
ASDR	El Salvador	Urticaria	2021	48.99	69.26	32.05
ASPR	El Salvador	Urticaria	1990	811.25	929.36	717.68

ASIR	El Salvador	Urticaria	1990	1431.73	1623.76	1258.04
ASDR	El Salvador	Urticaria	1990	48.56	68.79	32.29
ASPR	Equatorial Guinea	Urticaria	2021	801.97	917.45	709.53
ASIR	Equatorial Guinea	Urticaria	2021	1415.74	1606.44	1244.61
ASDR	Equatorial Guinea	Urticaria	2021	47.96	68.13	32.00
ASPR	Equatorial Guinea	Urticaria	1990	813.85	931.89	719.60
ASIR	Equatorial Guinea	Urticaria	1990	1436.20	1628.78	1262.46
ASDR	Equatorial Guinea	Urticaria	1990	48.05	69.08	31.69
ASPR	Eritrea	Urticaria	2021	808.46	925.60	715.34
ASIR	Eritrea	Urticaria	2021	1426.79	1618.49	1253.94
ASDR	Eritrea	Urticaria	2021	48.38	69.39	31.89
ASPR	Eritrea	Urticaria	1990	810.88	928.49	717.17
ASIR	Eritrea	Urticaria	1990	1431.06	1623.15	1258.01
ASDR	Eritrea	Urticaria	1990	48.21	69.20	31.79
ASPR	Estonia	Urticaria	2021	1049.07	1204.63	924.31
ASIR	Estonia	Urticaria	2021	1847.04	2115.21	1621.09
ASDR	Estonia	Urticaria	2021	63.28	90.15	42.07
ASPR	Estonia	Urticaria	1990	1057.39	1213.76	932.00
ASIR	Estonia	Urticaria	1990	1861.57	2131.67	1635.15
ASDR	Estonia	Urticaria	1990	63.69	91.03	42.65
ASPR	Eswatini	Urticaria	2021	811.69	929.27	717.86
ASIR	Eswatini	Urticaria	2021	1432.51	1624.79	1259.45
ASDR	Eswatini	Urticaria	2021	48.33	69.11	32.03
ASPR	Eswatini	Urticaria	1990	815.45	934.02	720.84
ASIR	Eswatini	Urticaria	1990	1438.99	1631.81	1264.62
ASDR	Eswatini	Urticaria	1990	48.93	70.59	31.89
ASPR	Ethiopia	Urticaria	2021	852.02	972.36	750.89
ASIR	Ethiopia	Urticaria	2021	1504.29	1706.87	1325.40
ASDR	Ethiopia	Urticaria	2021	51.02	72.49	33.45
ASPR	Ethiopia	Urticaria	1990	852.72	973.25	751.43
ASIR	Ethiopia	Urticaria	1990	1505.51	1708.52	1326.11
ASDR	Ethiopia	Urticaria	1990	50.75	72.70	33.32
ASPR	Fiji	Urticaria	2021	718.81	823.02	635.90
ASIR	Fiji	Urticaria	2021	1271.12	1452.01	1114.87
ASDR	Fiji	Urticaria	2021	43.18	61.28	28.38
ASPR	Fiji	Urticaria	1990	718.16	822.40	635.21
ASIR	Fiji	Urticaria	1990	1269.99	1451.07	1113.80
ASDR	Fiji	Urticaria	1990	43.12	61.41	28.27

ASPR	Finland	Urticaria	2021	582.68	653.57	514.70
ASIR	Finland	Urticaria	2021	1030.70	1148.04	917.57
ASDR	Finland	Urticaria	2021	34.69	48.62	23.01
ASPR	Finland	Urticaria	1990	587.37	658.41	518.61
ASIR	Finland	Urticaria	1990	1038.77	1156.49	925.35
ASDR	Finland	Urticaria	1990	34.96	49.37	23.06
ASPR	France	Urticaria	2021	767.70	850.26	683.46
ASIR	France	Urticaria	2021	1336.46	1475.69	1188.99
ASDR	France	Urticaria	2021	45.57	64.28	30.65
ASPR	France	Urticaria	1990	766.98	848.94	682.99
ASIR	France	Urticaria	1990	1335.15	1474.41	1188.08
ASDR	France	Urticaria	1990	45.58	63.28	30.23
ASPR	Gabon	Urticaria	2021	812.57	930.94	718.70
ASIR	Gabon	Urticaria	2021	1433.84	1625.95	1259.44
ASDR	Gabon	Urticaria	2021	48.47	68.99	31.93
ASPR	Gabon	Urticaria	1990	809.83	927.51	716.72
ASIR	Gabon	Urticaria	1990	1429.15	1620.94	1255.70
ASDR	Gabon	Urticaria	1990	48.09	67.34	31.73
ASPR	Gambia	Urticaria	2021	809.92	927.81	716.54
ASIR	Gambia	Urticaria	2021	1429.31	1621.21	1255.38
ASDR	Gambia	Urticaria	2021	48.49	69.15	31.86
ASPR	Gambia	Urticaria	1990	807.00	924.44	714.15
ASIR	Gambia	Urticaria	1990	1424.20	1615.80	1250.13
ASDR	Gambia	Urticaria	1990	48.21	68.80	32.14
ASPR	Georgia	Urticaria	2021	1050.11	1206.00	925.17
ASIR	Georgia	Urticaria	2021	1848.79	2116.51	1622.93
ASDR	Georgia	Urticaria	2021	63.46	89.30	41.54
ASPR	Georgia	Urticaria	1990	1058.06	1214.65	932.23
ASIR	Georgia	Urticaria	1990	1862.79	2132.51	1636.00
ASDR	Georgia	Urticaria	1990	63.97	90.83	42.44
ASPR	Germany	Urticaria	2021	477.97	525.04	433.09
ASIR	Germany	Urticaria	2021	855.35	935.84	769.76
ASDR	Germany	Urticaria	2021	28.39	39.51	18.75
ASPR	Germany	Urticaria	1990	482.89	530.11	437.67
ASIR	Germany	Urticaria	1990	864.06	944.43	777.63
ASDR	Germany	Urticaria	1990	28.65	39.99	19.41
ASPR	Ghana	Urticaria	2021	811.90	929.94	718.08
ASIR	Ghana	Urticaria	2021	1432.81	1624.97	1259.31
ASDR	Ghana	Urticaria	2021	48.65	68.64	32.21
ASPR	Ghana	Urticaria	1990	809.34	927.12	716.13
ASIR	Ghana	Urticaria	1990	1428.34	1620.31	1254.67
ASDR	Ghana	Urticaria	1990	48.24	69.47	32.09
ASPR	Greece	Urticaria	2021	658.71	732.77	581.68
ASIR	Greece	Urticaria	2021	1156.71	1284.31	1024.32

ASDR	Greece	Urticaria	2021	39.13	54.36	25.74
ASPR	Greece	Urticaria	1990	658.59	732.59	581.45
ASIR	Greece	Urticaria	1990	1156.49	1284.27	1024.33
ASDR	Greece	Urticaria	1990	39.26	54.12	25.68
ASPR	Greenland	Urticaria	2021	926.69	1059.91	812.41
ASIR	Greenland	Urticaria	2021	1630.15	1845.40	1432.08
ASDR	Greenland	Urticaria	2021	55.74	79.16	36.57
ASPR	Greenland	Urticaria	1990	915.68	1048.13	802.08
ASIR	Greenland	Urticaria	1990	1610.76	1823.97	1415.14
ASDR	Greenland	Urticaria	1990	55.00	77.60	36.14
ASPR	Grenada	Urticaria	2021	805.42	922.41	712.86
ASIR	Grenada	Urticaria	2021	1421.49	1612.60	1248.47
ASDR	Grenada	Urticaria	2021	48.32	68.28	31.89
ASPR	Grenada	Urticaria	1990	810.12	927.81	716.70
ASIR	Grenada	Urticaria	1990	1429.74	1621.55	1256.33
ASDR	Grenada	Urticaria	1990	48.55	69.65	32.07
ASPR	Guam	Urticaria	2021	717.48	821.78	634.68
ASIR	Guam	Urticaria	2021	1268.79	1449.60	1112.83
ASDR	Guam	Urticaria	2021	43.35	62.80	28.71
ASPR	Guam	Urticaria	1990	715.62	820.24	633.06
ASIR	Guam	Urticaria	1990	1265.56	1446.52	1109.56
ASDR	Guam	Urticaria	1990	43.30	62.64	28.60
ASPR	Guatemala	Urticaria	2021	811.59	929.82	717.88
ASIR	Guatemala	Urticaria	2021	1432.31	1624.37	1258.50
ASDR	Guatemala	Urticaria	2021	48.57	68.71	31.64
ASPR	Guatemala	Urticaria	1990	810.14	928.15	716.81
ASIR	Guatemala	Urticaria	1990	1429.71	1621.55	1255.69
ASDR	Guatemala	Urticaria	1990	48.28	68.38	31.57
ASPR	Guinea	Urticaria	2021	811.49	929.90	717.69
ASIR	Guinea	Urticaria	2021	1432.09	1624.14	1258.09
ASDR	Guinea	Urticaria	2021	48.53	69.14	32.41
ASPR	Guinea	Urticaria	1990	811.28	929.59	717.51
ASIR	Guinea	Urticaria	1990	1431.74	1624.06	1257.70
ASDR	Guinea	Urticaria	1990	48.32	69.55	32.21
ASPR	Guinea-Bissau	Urticaria	2021	812.08	930.31	718.19
ASIR	Guinea-Bissau	Urticaria	2021	1433.12	1625.21	1259.40
ASDR	Guinea-Bissau	Urticaria	2021	48.67	69.69	32.65
ASPR	Guinea-Bissau	Urticaria	1990	812.72	931.34	718.82
ASIR	Guinea-Bissau	Urticaria	1990	1434.22	1626.34	1260.01
ASDR	Guinea-Bissau	Urticaria	1990	48.42	68.42	31.85
ASPR	Guyana	Urticaria	2021	809.39	927.11	716.07
ASIR	Guyana	Urticaria	2021	1428.38	1620.21	1254.78
ASDR	Guyana	Urticaria	2021	48.34	69.02	32.00
ASPR	Guyana	Urticaria	1990	809.52	927.34	716.23

ASIR	Guyana	Urticaria	1990	1428.53	1620.32	1254.56
ASDR	Guyana	Urticaria	1990	48.37	68.47	32.24
ASPR	Haiti	Urticaria	2021	810.51	928.54	716.96
ASIR	Haiti	Urticaria	2021	1430.39	1622.31	1256.56
ASDR	Haiti	Urticaria	2021	48.17	67.49	31.78
ASPR	Haiti	Urticaria	1990	811.74	929.99	717.97
ASIR	Haiti	Urticaria	1990	1432.47	1624.61	1258.14
ASDR	Haiti	Urticaria	1990	48.20	68.56	31.33
ASPR	Honduras	Urticaria	2021	811.21	929.33	717.52
ASIR	Honduras	Urticaria	2021	1431.63	1623.70	1257.98
ASDR	Honduras	Urticaria	2021	48.75	69.30	31.80
ASPR	Honduras	Urticaria	1990	809.30	927.09	716.18
ASIR	Honduras	Urticaria	1990	1428.28	1620.05	1254.65
ASDR	Honduras	Urticaria	1990	48.64	69.22	31.78
ASPR	Hungary	Urticaria	2021	1088.24	1253.82	960.64
ASIR	Hungary	Urticaria	2021	1914.91	2179.71	1679.82
ASDR	Hungary	Urticaria	2021	65.83	94.26	43.40
ASPR	Hungary	Urticaria	1990	1092.23	1258.26	964.54
ASIR	Hungary	Urticaria	1990	1921.89	2186.87	1686.58
ASDR	Hungary	Urticaria	1990	65.90	93.69	43.88
ASPR	Iceland	Urticaria	2021	580.76	651.56	513.07
ASIR	Iceland	Urticaria	2021	1027.37	1144.85	914.60
ASDR	Iceland	Urticaria	2021	34.66	48.27	22.61
ASPR	Iceland	Urticaria	1990	583.89	654.92	515.73
ASIR	Iceland	Urticaria	1990	1032.87	1150.01	919.50
ASDR	Iceland	Urticaria	1990	34.82	48.44	22.78
ASPR	India	Urticaria	2021	925.55	1060.24	811.94
ASIR	India	Urticaria	2021	1635.70	1856.70	1443.71
ASDR	India	Urticaria	2021	55.26	79.80	36.43
ASPR	India	Urticaria	1990	923.89	1058.63	810.25
ASIR	India	Urticaria	1990	1632.92	1853.56	1441.07
ASDR	India	Urticaria	1990	54.82	78.70	36.29
ASPR	Indonesia	Urticaria	2021	787.54	899.19	688.77
ASIR	Indonesia	Urticaria	2021	1394.41	1586.25	1220.45
ASDR	Indonesia	Urticaria	2021	47.51	68.46	31.32
ASPR	Indonesia	Urticaria	1990	786.73	898.15	687.98
ASIR	Indonesia	Urticaria	1990	1392.94	1584.94	1219.48
ASDR	Indonesia	Urticaria	1990	47.27	67.90	31.16
ASPR	Iran (Islamic Republic of)	Urticaria	2021	958.98	1089.83	845.78
ASIR	Iran (Islamic Republic of)	Urticaria	2021	1693.48	1929.43	1500.06
ASDR	Iran (Islamic Republic of)	Urticaria	2021	57.46	80.91	38.00

ASPR	Iran (Islamic Republic of)	Urticaria	1990	959.26	1090.33	846.03
ASIR	Iran (Islamic Republic of)	Urticaria	1990	1694.03	1930.02	1500.53
ASDR	Iran (Islamic Republic of)	Urticaria	1990	57.39	81.55	38.02
ASPR	Iraq	Urticaria	2021	905.71	1034.35	795.32
ASIR	Iraq	Urticaria	2021	1598.13	1801.93	1411.67
ASDR	Iraq	Urticaria	2021	54.17	76.29	35.87
ASPR	Iraq	Urticaria	1990	906.69	1035.44	796.20
ASIR	Iraq	Urticaria	1990	1599.85	1803.69	1413.23
ASDR	Iraq	Urticaria	1990	54.15	75.89	35.65
ASPR	Ireland	Urticaria	2021	587.95	659.70	519.07
ASIR	Ireland	Urticaria	2021	1040.00	1158.34	925.61
ASDR	Ireland	Urticaria	2021	34.91	49.27	23.01
ASPR	Ireland	Urticaria	1990	586.12	657.34	517.56
ASIR	Ireland	Urticaria	1990	1036.75	1154.19	922.96
ASDR	Ireland	Urticaria	1990	34.95	48.69	23.38
ASPR	Israel	Urticaria	2021	526.91	584.04	471.15
ASIR	Israel	Urticaria	2021	937.50	1036.31	837.41
ASDR	Israel	Urticaria	2021	31.44	43.57	20.95
ASPR	Israel	Urticaria	1990	530.70	588.34	474.40
ASIR	Israel	Urticaria	1990	944.18	1043.50	843.38
ASDR	Israel	Urticaria	1990	31.63	43.47	20.89
ASPR	Italy	Urticaria	2021	498.60	560.19	439.78
ASIR	Italy	Urticaria	2021	884.63	988.06	785.19
ASDR	Italy	Urticaria	2021	29.56	41.20	19.44
ASPR	Italy	Urticaria	1990	501.82	563.85	442.60
ASIR	Italy	Urticaria	1990	890.30	994.40	789.93
ASDR	Italy	Urticaria	1990	29.71	41.41	19.60
ASPR	Jamaica	Urticaria	2021	808.33	925.97	715.21
ASIR	Jamaica	Urticaria	2021	1426.60	1618.20	1252.94
ASDR	Jamaica	Urticaria	2021	48.62	69.87	32.04
ASPR	Jamaica	Urticaria	1990	810.17	928.03	716.76
ASIR	Jamaica	Urticaria	1990	1429.80	1621.76	1255.96
ASDR	Jamaica	Urticaria	1990	48.70	69.21	31.74
ASPR	Japan	Urticaria	2021	810.71	922.40	715.69
ASIR	Japan	Urticaria	2021	1431.25	1625.45	1262.34
ASDR	Japan	Urticaria	2021	49.04	70.24	32.29
ASPR	Japan	Urticaria	1990	814.04	926.05	718.79
ASIR	Japan	Urticaria	1990	1437.02	1632.19	1267.69
ASDR	Japan	Urticaria	1990	49.16	69.73	32.39
ASPR	Jordan	Urticaria	2021	900.85	1029.60	790.92
ASIR	Jordan	Urticaria	2021	1589.68	1794.15	1403.82

ASDR	Jordan	Urticaria	2021	54.10	76.43	35.55
ASPR	Jordan	Urticaria	1990	904.07	1032.84	793.90
ASIR	Jordan	Urticaria	1990	1595.32	1799.05	1409.19
ASDR	Jordan	Urticaria	1990	54.34	76.91	36.28
ASPR	Kazakhstan	Urticaria	2021	1055.21	1211.54	929.81
ASIR	Kazakhstan	Urticaria	2021	1857.81	2126.57	1631.40
ASDR	Kazakhstan	Urticaria	2021	63.62	90.77	42.14
ASPR	Kazakhstan	Urticaria	1990	1057.14	1213.33	931.65
ASIR	Kazakhstan	Urticaria	1990	1861.16	2131.56	1634.68
ASDR	Kazakhstan	Urticaria	1990	63.60	92.01	42.55
ASPR	Kenya	Urticaria	2021	854.42	975.22	753.29
ASIR	Kenya	Urticaria	2021	1508.44	1711.30	1328.86
ASDR	Kenya	Urticaria	2021	51.29	73.35	33.63
ASPR	Kenya	Urticaria	1990	854.20	975.02	752.98
ASIR	Kenya	Urticaria	1990	1508.06	1711.08	1328.43
ASDR	Kenya	Urticaria	1990	51.16	73.54	33.55
ASPR	Kiribati	Urticaria	2021	721.22	825.13	638.14
ASIR	Kiribati	Urticaria	2021	1275.32	1455.74	1119.24
ASDR	Kiribati	Urticaria	2021	43.30	62.01	28.68
ASPR	Kiribati	Urticaria	1990	720.57	824.67	637.57
ASIR	Kiribati	Urticaria	1990	1274.19	1454.68	1118.16
ASDR	Kiribati	Urticaria	1990	43.03	61.18	28.50
ASPR	Kuwait	Urticaria	2021	902.54	1030.92	792.26
ASIR	Kuwait	Urticaria	2021	1592.66	1795.57	1406.81
ASDR	Kuwait	Urticaria	2021	54.17	77.31	35.61
ASPR	Kuwait	Urticaria	1990	890.87	1019.84	781.95
ASIR	Kuwait	Urticaria	1990	1572.21	1778.19	1387.42
ASDR	Kuwait	Urticaria	1990	53.74	75.97	35.59
ASPR	Kyrgyzstan	Urticaria	2021	1053.24	1209.53	927.95
ASIR	Kyrgyzstan	Urticaria	2021	1854.45	2122.83	1628.18
ASDR	Kyrgyzstan	Urticaria	2021	63.64	89.40	42.15
ASPR	Kyrgyzstan	Urticaria	1990	1055.50	1211.72	930.10
ASIR	Kyrgyzstan	Urticaria	1990	1858.35	2128.13	1631.94
ASDR	Kyrgyzstan	Urticaria	1990	63.53	89.61	42.13
ASPR	Lao People's Democratic Republic	Urticaria	2021	749.77	856.15	651.71
ASIR	Lao People's Democratic Republic	Urticaria	2021	1327.57	1523.42	1160.48
ASDR	Lao People's Democratic Republic	Urticaria	2021	45.20	64.61	29.94

ASPR	Lao People's Democratic Republic	Urticaria	1990	748.33	854.69	650.42
ASIR	Lao People's Democratic Republic	Urticaria	1990	1324.98	1520.66	1157.95
ASDR	Lao People's Democratic Republic	Urticaria	1990	44.84	65.09	29.51
ASPR	Latvia	Urticaria	2021	1052.34	1208.30	927.31
ASIR	Latvia	Urticaria	2021	1852.73	2121.28	1626.72
ASDR	Latvia	Urticaria	2021	63.61	90.40	42.25
ASPR	Latvia	Urticaria	1990	1058.40	1214.83	932.91
ASIR	Latvia	Urticaria	1990	1863.33	2133.81	1636.80
ASDR	Latvia	Urticaria	1990	63.90	90.37	42.32
ASPR	Lebanon	Urticaria	2021	908.19	1036.32	797.94
ASIR	Lebanon	Urticaria	2021	1602.49	1805.48	1415.86
ASDR	Lebanon	Urticaria	2021	54.34	77.88	35.87
ASPR	Lebanon	Urticaria	1990	911.68	1039.69	800.74
ASIR	Lebanon	Urticaria	1990	1608.55	1810.19	1421.73
ASDR	Lebanon	Urticaria	1990	54.57	77.39	35.76
ASPR	Lesotho	Urticaria	2021	813.10	931.03	719.08
ASIR	Lesotho	Urticaria	2021	1434.90	1627.21	1261.41
ASDR	Lesotho	Urticaria	2021	48.31	68.84	32.03
ASPR	Lesotho	Urticaria	1990	820.79	939.10	725.42
ASIR	Lesotho	Urticaria	1990	1448.33	1641.73	1274.39
ASDR	Lesotho	Urticaria	1990	49.18	71.08	32.11
ASPR	Liberia	Urticaria	2021	806.16	923.39	713.47
ASIR	Liberia	Urticaria	2021	1422.74	1614.22	1249.18
ASDR	Liberia	Urticaria	2021	47.82	68.59	31.69
ASPR	Liberia	Urticaria	1990	807.11	924.73	714.33
ASIR	Liberia	Urticaria	1990	1424.43	1616.07	1250.32
ASDR	Liberia	Urticaria	1990	47.65	68.19	31.19
ASPR	Libya	Urticaria	2021	907.15	1035.81	796.56
ASIR	Libya	Urticaria	2021	1600.67	1804.61	1413.90
ASDR	Libya	Urticaria	2021	54.31	77.15	35.99
ASPR	Libya	Urticaria	1990	904.07	1034.09	793.39
ASIR	Libya	Urticaria	1990	1595.26	1801.32	1408.24
ASDR	Libya	Urticaria	1990	54.34	77.34	36.21
ASPR	Lithuania	Urticaria	2021	1052.93	1209.02	927.80
ASIR	Lithuania	Urticaria	2021	1853.81	2122.59	1627.79
ASDR	Lithuania	Urticaria	2021	63.58	90.85	41.94
ASPR	Lithuania	Urticaria	1990	1057.48	1214.08	932.08
ASIR	Lithuania	Urticaria	1990	1861.81	2131.75	1635.35

ASDR	Lithuania	Urticaria	1990	63.83	90.67	42.45
ASPR	Luxembourg	Urticaria	2021	582.33	653.25	514.32
ASIR	Luxembourg	Urticaria	2021	1030.11	1147.12	917.20
ASDR	Luxembourg	Urticaria	2021	34.72	48.20	23.21
ASPR	Luxembourg	Urticaria	1990	585.94	656.84	517.45
ASIR	Luxembourg	Urticaria	1990	1036.35	1153.43	923.08
ASDR	Luxembourg	Urticaria	1990	34.84	48.80	22.48
ASPR	Madagascar	Urticaria	2021	810.13	928.08	716.72
ASIR	Madagascar	Urticaria	2021	1429.66	1621.49	1255.80
ASDR	Madagascar	Urticaria	2021	48.66	70.33	32.35
ASPR	Madagascar	Urticaria	1990	808.58	926.39	715.55
ASIR	Madagascar	Urticaria	1990	1426.98	1618.53	1253.15
ASDR	Madagascar	Urticaria	1990	48.14	67.87	32.08
ASPR	Malawi	Urticaria	2021	812.25	930.32	718.31
ASIR	Malawi	Urticaria	2021	1433.37	1625.57	1259.36
ASDR	Malawi	Urticaria	2021	48.71	69.85	32.26
ASPR	Malawi	Urticaria	1990	810.84	928.85	717.31
ASIR	Malawi	Urticaria	1990	1430.93	1622.84	1256.94
ASDR	Malawi	Urticaria	1990	48.11	68.19	32.07
ASPR	Malaysia	Urticaria	2021	751.06	857.33	653.00
ASIR	Malaysia	Urticaria	2021	1329.85	1525.70	1162.58
ASDR	Malaysia	Urticaria	2021	45.16	65.33	29.96
ASPR	Malaysia	Urticaria	1990	749.82	856.13	651.86
ASIR	Malaysia	Urticaria	1990	1327.65	1523.41	1160.57
ASDR	Malaysia	Urticaria	1990	45.20	65.08	29.49
ASPR	Maldives	Urticaria	2021	759.59	866.07	661.18
ASIR	Maldives	Urticaria	2021	1344.97	1540.25	1176.22
ASDR	Maldives	Urticaria	2021	45.76	65.68	30.23
ASPR	Maldives	Urticaria	1990	750.71	857.17	653.24
ASIR	Maldives	Urticaria	1990	1328.96	1524.88	1161.66
ASDR	Maldives	Urticaria	1990	44.96	64.18	29.22
ASPR	Mali	Urticaria	2021	808.86	926.71	715.51
ASIR	Mali	Urticaria	2021	1427.51	1619.35	1253.63
ASDR	Mali	Urticaria	2021	48.15	69.03	31.58
ASPR	Mali	Urticaria	1990	810.32	928.45	716.82
ASIR	Mali	Urticaria	1990	1430.05	1622.12	1256.13
ASDR	Mali	Urticaria	1990	48.22	68.02	32.04
ASPR	Malta	Urticaria	2021	581.44	652.25	513.65
ASIR	Malta	Urticaria	2021	1028.52	1145.67	915.63
ASDR	Malta	Urticaria	2021	34.68	48.64	22.62
ASPR	Malta	Urticaria	1990	588.23	659.82	519.40
ASIR	Malta	Urticaria	1990	1040.33	1158.98	926.04
ASDR	Malta	Urticaria	1990	35.06	49.98	23.31
ASPR	Marshall	Urticaria	2021	717.37	821.57	634.42

	Islands					
ASIR	Marshall Islands	Urticaria	2021	1268.61	1449.81	1112.35
ASDR	Marshall Islands	Urticaria	2021	43.00	61.28	28.12
ASPR	Marshall Islands	Urticaria	1990	718.32	822.50	635.41
ASIR	Marshall Islands	Urticaria	1990	1270.22	1451.30	1113.57
ASDR	Marshall Islands	Urticaria	1990	43.09	61.45	28.37
ASPR	Mauritania	Urticaria	2021	810.10	928.17	716.76
ASIR	Mauritania	Urticaria	2021	1429.66	1621.52	1255.80
ASDR	Mauritania	Urticaria	2021	48.69	69.71	31.36
ASPR	Mauritania	Urticaria	1990	809.61	927.42	716.34
ASIR	Mauritania	Urticaria	1990	1428.78	1620.60	1255.20
ASDR	Mauritania	Urticaria	1990	48.42	68.46	32.03
ASPR	Mauritius	Urticaria	2021	750.07	856.43	651.93
ASIR	Mauritius	Urticaria	2021	1328.13	1523.85	1161.01
ASDR	Mauritius	Urticaria	2021	45.00	64.67	29.13
ASPR	Mauritius	Urticaria	1990	749.91	856.32	651.66
ASIR	Mauritius	Urticaria	1990	1327.86	1523.69	1160.67
ASDR	Mauritius	Urticaria	1990	44.97	64.32	29.53
ASPR	Mexico	Urticaria	2021	855.24	975.82	754.08
ASIR	Mexico	Urticaria	2021	1509.94	1712.74	1330.52
ASDR	Mexico	Urticaria	2021	51.45	73.84	33.83
ASPR	Mexico	Urticaria	1990	855.11	975.83	753.80
ASIR	Mexico	Urticaria	1990	1509.71	1712.70	1329.98
ASDR	Mexico	Urticaria	1990	51.34	73.30	33.71
ASPR	Micronesia (Federated States of)	Urticaria	2021	718.33	822.53	635.48
ASIR	Micronesia (Federated States of)	Urticaria	2021	1270.30	1450.97	1114.29
ASDR	Micronesia (Federated States of)	Urticaria	2021	43.16	62.26	28.41
ASPR	Micronesia (Federated States of)	Urticaria	1990	717.93	821.89	635.03
ASIR	Micronesia (Federated States of)	Urticaria	1990	1269.57	1450.41	1113.35

ASDR	Micronesia (Federated States of)	Urticaria	1990	43.09	61.43	27.95
ASPR	Monaco	Urticaria	2021	588.48	660.21	519.27
ASIR	Monaco	Urticaria	2021	1040.85	1160.12	926.71
ASDR	Monaco	Urticaria	2021	35.03	49.13	22.91
ASPR	Monaco	Urticaria	1990	589.47	661.30	520.33
ASIR	Monaco	Urticaria	1990	1042.54	1160.61	928.04
ASDR	Monaco	Urticaria	1990	35.16	49.37	23.07
ASPR	Mongolia	Urticaria	2021	1053.78	1210.16	928.51
ASIR	Mongolia	Urticaria	2021	1855.39	2124.25	1629.14
ASDR	Mongolia	Urticaria	2021	63.53	90.28	42.16
ASPR	Mongolia	Urticaria	1990	1049.53	1205.41	924.32
ASIR	Mongolia	Urticaria	1990	1848.05	2115.77	1621.91
ASDR	Mongolia	Urticaria	1990	63.14	89.57	42.11
ASPR	Montenegro	Urticaria	2021	1085.78	1250.97	958.68
ASIR	Montenegro	Urticaria	2021	1910.63	2174.58	1675.61
ASDR	Montenegro	Urticaria	2021	65.72	93.56	43.35
ASPR	Montenegro	Urticaria	1990	1087.97	1253.56	960.61
ASIR	Montenegro	Urticaria	1990	1914.48	2179.09	1679.29
ASDR	Montenegro	Urticaria	1990	65.97	94.32	43.64
ASPR	Morocco	Urticaria	2021	909.41	1038.07	798.62
ASIR	Morocco	Urticaria	2021	1604.61	1807.99	1417.65
ASDR	Morocco	Urticaria	2021	54.48	77.25	35.80
ASPR	Morocco	Urticaria	1990	912.07	1041.17	800.87
ASIR	Morocco	Urticaria	1990	1609.20	1813.23	1421.65
ASDR	Morocco	Urticaria	1990	54.68	77.48	36.06
ASPR	Mozambique	Urticaria	2021	813.86	932.35	719.72
ASIR	Mozambique	Urticaria	2021	1436.21	1628.44	1262.20
ASDR	Mozambique	Urticaria	2021	48.41	68.40	31.67
ASPR	Mozambique	Urticaria	1990	813.77	932.39	719.65
ASIR	Mozambique	Urticaria	1990	1436.07	1628.36	1261.83
ASDR	Mozambique	Urticaria	1990	48.23	68.04	31.99
ASPR	Myanmar	Urticaria	2021	748.03	854.38	649.98
ASIR	Myanmar	Urticaria	2021	1324.59	1520.35	1157.53
ASDR	Myanmar	Urticaria	2021	44.92	64.40	29.85
ASPR	Myanmar	Urticaria	1990	748.97	855.33	650.89
ASIR	Myanmar	Urticaria	1990	1326.15	1521.75	1159.03
ASDR	Myanmar	Urticaria	1990	44.77	64.67	29.29
ASPR	Namibia	Urticaria	2021	812.69	930.76	718.67
ASIR	Namibia	Urticaria	2021	1434.18	1626.40	1260.37
ASDR	Namibia	Urticaria	2021	48.60	70.20	32.28
ASPR	Namibia	Urticaria	1990	811.71	929.75	717.93
ASIR	Namibia	Urticaria	1990	1432.44	1624.59	1258.46

ASDR	Namibia	Urticaria	1990	48.67	68.39	32.07
ASPR	Nauru	Urticaria	2021	718.97	822.86	636.21
ASIR	Nauru	Urticaria	2021	1271.43	1451.63	1115.38
ASDR	Nauru	Urticaria	2021	43.18	62.54	28.36
ASPR	Nauru	Urticaria	1990	715.90	820.38	633.04
ASIR	Nauru	Urticaria	1990	1265.90	1447.49	1108.92
ASDR	Nauru	Urticaria	1990	43.10	61.92	28.11
ASPR	Nepal	Urticaria	2021	1476.34	1605.27	1317.43
ASIR	Nepal	Urticaria	2021	2563.35	2794.79	2274.38
ASDR	Nepal	Urticaria	2021	88.44	124.89	57.09
ASPR	Nepal	Urticaria	1990	1461.09	1587.99	1306.37
ASIR	Nepal	Urticaria	1990	2537.62	2768.41	2253.65
ASDR	Nepal	Urticaria	1990	86.92	121.05	56.65
ASPR	Netherlands	Urticaria	2021	584.79	656.11	516.42
ASIR	Netherlands	Urticaria	2021	1034.43	1152.21	920.90
ASDR	Netherlands	Urticaria	2021	34.85	48.61	22.92
ASPR	Netherlands	Urticaria	1990	585.12	656.10	516.73
ASIR	Netherlands	Urticaria	1990	1034.92	1152.42	921.50
ASDR	Netherlands	Urticaria	1990	34.97	48.66	23.02
ASPR	New Zealand	Urticaria	2021	897.97	1010.98	793.67
ASIR	New Zealand	Urticaria	2021	1585.95	1791.88	1401.91
ASDR	New Zealand	Urticaria	2021	53.74	76.51	35.13
ASPR	New Zealand	Urticaria	1990	902.28	1015.13	796.85
ASIR	New Zealand	Urticaria	1990	1593.56	1800.01	1408.85
ASDR	New Zealand	Urticaria	1990	53.88	77.12	36.22
ASPR	Nicaragua	Urticaria	2021	809.35	927.04	716.12
ASIR	Nicaragua	Urticaria	2021	1428.38	1620.11	1255.12
ASDR	Nicaragua	Urticaria	2021	48.66	69.03	32.29
ASPR	Nicaragua	Urticaria	1990	810.97	929.04	717.48
ASIR	Nicaragua	Urticaria	1990	1431.17	1623.14	1257.34
ASDR	Nicaragua	Urticaria	1990	48.69	69.46	31.82
ASPR	Niger	Urticaria	2021	809.77	927.78	716.55
ASIR	Niger	Urticaria	2021	1429.09	1620.74	1255.44
ASDR	Niger	Urticaria	2021	48.59	69.34	32.14
ASPR	Niger	Urticaria	1990	808.50	926.28	715.57
ASIR	Niger	Urticaria	1990	1426.79	1618.48	1252.75
ASDR	Niger	Urticaria	1990	48.16	67.95	31.41
ASPR	Nigeria	Urticaria	2021	859.10	980.23	757.39
ASIR	Nigeria	Urticaria	2021	1516.57	1720.02	1336.22
ASDR	Nigeria	Urticaria	2021	51.33	73.36	33.64
ASPR	Nigeria	Urticaria	1990	852.01	973.93	750.28
ASIR	Nigeria	Urticaria	1990	1504.06	1707.64	1323.77
ASDR	Nigeria	Urticaria	1990	50.61	72.39	33.16
ASPR	Niue	Urticaria	2021	718.22	822.62	635.58

ASIR	Niue	Urticaria	2021	1270.01	1449.32	1114.34
ASDR	Niue	Urticaria	2021	43.20	61.88	28.25
ASPR	Niue	Urticaria	1990	718.08	822.68	635.39
ASIR	Niue	Urticaria	1990	1269.77	1449.32	1114.35
ASDR	Niue	Urticaria	1990	43.26	62.02	28.44
ASPR	North Macedonia	Urticaria	2021	1080.87	1246.13	953.66
ASIR	North Macedonia	Urticaria	2021	1902.10	2165.83	1667.70
ASDR	North Macedonia	Urticaria	2021	65.35	94.24	43.50
ASPR	North Macedonia	Urticaria	1990	1085.93	1251.82	958.69
ASIR	North Macedonia	Urticaria	1990	1911.03	2174.92	1675.93
ASDR	North Macedonia	Urticaria	1990	65.68	94.67	43.76
ASPR	Northern Mariana Islands	Urticaria	2021	715.91	819.99	633.27
ASIR	Northern Mariana Islands	Urticaria	2021	1266.10	1447.11	1110.12
ASDR	Northern Mariana Islands	Urticaria	2021	43.18	61.40	28.07
ASPR	Northern Mariana Islands	Urticaria	1990	718.39	823.05	634.93
ASIR	Northern Mariana Islands	Urticaria	1990	1270.11	1452.06	1110.81
ASDR	Northern Mariana Islands	Urticaria	1990	43.37	62.24	28.31
ASPR	Norway	Urticaria	2021	615.29	689.15	545.03
ASIR	Norway	Urticaria	2021	1090.41	1213.87	965.22
ASDR	Norway	Urticaria	2021	36.65	50.70	24.15
ASPR	Norway	Urticaria	1990	618.52	693.07	548.03
ASIR	Norway	Urticaria	1990	1096.01	1219.92	970.40
ASDR	Norway	Urticaria	1990	36.77	51.06	24.32
ASPR	Oman	Urticaria	2021	886.71	1015.60	778.56
ASIR	Oman	Urticaria	2021	1565.04	1772.85	1380.62
ASDR	Oman	Urticaria	2021	53.30	74.33	35.42

ASPR	Oman	Urticaria	1990	885.22	1014.20	777.41
ASIR	Oman	Urticaria	1990	1562.59	1770.52	1378.03
ASDR	Oman	Urticaria	1990	53.12	75.67	34.81
ASPR	Pakistan	Urticaria	2021	1196.03	1357.49	1055.86
ASIR	Pakistan	Urticaria	2021	2111.54	2399.16	1867.18
ASDR	Pakistan	Urticaria	2021	71.43	101.49	47.38
ASPR	Pakistan	Urticaria	1990	1190.34	1350.84	1050.79
ASIR	Pakistan	Urticaria	1990	2101.63	2388.59	1858.24
ASDR	Pakistan	Urticaria	1990	70.93	100.44	46.78
ASPR	Palau	Urticaria	2021	712.93	817.22	630.54
ASIR	Palau	Urticaria	2021	1261.04	1441.79	1105.02
ASDR	Palau	Urticaria	2021	42.92	60.56	28.21
ASPR	Palau	Urticaria	1990	717.18	821.60	634.39
ASIR	Palau	Urticaria	1990	1268.27	1449.28	1112.21
ASDR	Palau	Urticaria	1990	43.19	62.57	28.68
ASPR	Palestine	Urticaria	2021	908.50	1037.32	797.81
ASIR	Palestine	Urticaria	2021	1602.99	1806.46	1416.17
ASDR	Palestine	Urticaria	2021	54.39	75.71	35.95
ASPR	Palestine	Urticaria	1990	912.50	1041.15	801.59
ASIR	Palestine	Urticaria	1990	1610.01	1813.61	1422.62
ASDR	Palestine	Urticaria	1990	54.71	77.47	35.93
ASPR	Panama	Urticaria	2021	806.71	924.00	713.90
ASIR	Panama	Urticaria	2021	1423.73	1615.13	1250.43
ASDR	Panama	Urticaria	2021	48.58	68.62	31.63
ASPR	Panama	Urticaria	1990	806.60	923.93	713.87
ASIR	Panama	Urticaria	1990	1423.55	1614.96	1250.04
ASDR	Panama	Urticaria	1990	48.61	69.06	31.70
ASPR	Papua New Guinea	Urticaria	2021	715.52	819.71	632.92
ASIR	Papua New Guinea	Urticaria	2021	1265.30	1445.63	1109.44
ASDR	Papua New Guinea	Urticaria	2021	42.94	61.24	28.31
ASPR	Papua New Guinea	Urticaria	1990	716.01	820.06	633.37
ASIR	Papua New Guinea	Urticaria	1990	1266.22	1446.78	1110.14
ASDR	Papua New Guinea	Urticaria	1990	42.73	61.32	28.25
ASPR	Paraguay	Urticaria	2021	806.81	924.07	714.08
ASIR	Paraguay	Urticaria	2021	1423.91	1615.30	1250.64
ASDR	Paraguay	Urticaria	2021	48.44	68.78	32.05
ASPR	Paraguay	Urticaria	1990	807.68	925.10	714.86
ASIR	Paraguay	Urticaria	1990	1425.43	1616.98	1251.94

ASDR	Paraguay	Urticaria	1990	48.48	69.56	31.75
ASPR	Peru	Urticaria	2021	806.60	923.89	713.69
ASIR	Peru	Urticaria	2021	1423.63	1615.06	1250.61
ASDR	Peru	Urticaria	2021	48.59	69.85	31.95
ASPR	Peru	Urticaria	1990	808.83	926.54	715.72
ASIR	Peru	Urticaria	1990	1427.45	1619.14	1253.72
ASDR	Peru	Urticaria	1990	48.60	68.09	31.77
ASPR	Philippines	Urticaria	2021	787.92	899.52	689.16
ASIR	Philippines	Urticaria	2021	1395.11	1587.17	1221.19
ASDR	Philippines	Urticaria	2021	47.39	67.91	31.30
ASPR	Philippines	Urticaria	1990	787.06	898.68	688.35
ASIR	Philippines	Urticaria	1990	1393.54	1585.30	1219.89
ASDR	Philippines	Urticaria	1990	47.17	68.06	31.17
ASPR	Poland	Urticaria	2021	1238.52	1341.99	1143.63
ASIR	Poland	Urticaria	2021	2181.82	2356.23	2012.04
ASDR	Poland	Urticaria	2021	74.84	104.99	50.28
ASPR	Poland	Urticaria	1990	1289.84	1454.25	1155.47
ASIR	Poland	Urticaria	1990	2271.57	2546.19	2030.02
ASDR	Poland	Urticaria	1990	77.76	110.04	51.89
ASPR	Portugal	Urticaria	2021	465.07	521.12	414.15
ASIR	Portugal	Urticaria	2021	833.63	929.89	737.02
ASDR	Portugal	Urticaria	2021	27.67	38.83	18.41
ASPR	Portugal	Urticaria	1990	465.73	521.85	414.72
ASIR	Portugal	Urticaria	1990	834.78	930.97	738.03
ASDR	Portugal	Urticaria	1990	27.66	38.66	18.39
ASPR	Puerto Rico	Urticaria	2021	809.85	927.62	716.47
ASIR	Puerto Rico	Urticaria	2021	1429.24	1621.07	1255.84
ASDR	Puerto Rico	Urticaria	2021	48.74	69.05	32.08
ASPR	Puerto Rico	Urticaria	1990	810.64	928.64	717.10
ASIR	Puerto Rico	Urticaria	1990	1430.62	1622.60	1257.04
ASDR	Puerto Rico	Urticaria	1990	48.80	69.39	32.16
ASPR	Qatar	Urticaria	2021	870.66	999.40	764.21
ASIR	Qatar	Urticaria	2021	1537.21	1745.09	1354.12
ASDR	Qatar	Urticaria	2021	52.38	73.90	34.69
ASPR	Qatar	Urticaria	1990	869.28	997.27	763.18
ASIR	Qatar	Urticaria	1990	1534.84	1742.88	1352.67
ASDR	Qatar	Urticaria	1990	52.40	75.49	35.06
ASPR	Republic of Korea	Urticaria	2021	764.14	868.27	675.40
ASIR	Republic of Korea	Urticaria	2021	1347.68	1525.58	1189.89
ASDR	Republic of Korea	Urticaria	2021	46.20	66.62	30.20

ASPR	Republic of Korea	Urticaria	1990	771.06	876.10	681.90
ASIR	Republic of Korea	Urticaria	1990	1359.63	1538.06	1200.81
ASDR	Republic of Korea	Urticaria	1990	46.55	66.38	30.76
ASPR	Republic of Moldova	Urticaria	2021	1051.97	1208.10	926.92
ASIR	Republic of Moldova	Urticaria	2021	1852.14	2120.68	1626.08
ASDR	Republic of Moldova	Urticaria	2021	63.61	89.47	41.48
ASPR	Republic of Moldova	Urticaria	1990	1058.63	1215.47	932.80
ASIR	Republic of Moldova	Urticaria	1990	1863.87	2133.64	1636.92
ASDR	Republic of Moldova	Urticaria	1990	63.84	90.70	42.42
ASPR	Romania	Urticaria	2021	1005.38	1161.14	879.84
ASIR	Romania	Urticaria	2021	1777.62	2025.98	1560.06
ASDR	Romania	Urticaria	2021	60.75	85.95	39.88
ASPR	Romania	Urticaria	1990	1004.15	1123.78	900.14
ASIR	Romania	Urticaria	1990	1775.92	1981.59	1592.97
ASDR	Romania	Urticaria	1990	60.57	85.65	40.31
ASPR	Russian Federation	Urticaria	2021	1109.16	1275.66	974.62
ASIR	Russian Federation	Urticaria	2021	1954.44	2235.34	1714.53
ASDR	Russian Federation	Urticaria	2021	66.84	95.26	44.56
ASPR	Russian Federation	Urticaria	1990	1112.41	1279.07	977.30
ASIR	Russian Federation	Urticaria	1990	1960.02	2241.75	1719.61
ASDR	Russian Federation	Urticaria	1990	66.96	94.85	44.25
ASPR	Rwanda	Urticaria	2021	812.51	930.63	718.57
ASIR	Rwanda	Urticaria	2021	1433.85	1626.01	1260.19
ASDR	Rwanda	Urticaria	2021	48.81	69.50	32.12
ASPR	Rwanda	Urticaria	1990	812.70	931.05	718.83
ASIR	Rwanda	Urticaria	1990	1434.13	1626.18	1260.09
ASDR	Rwanda	Urticaria	1990	48.50	69.50	31.75
ASPR	Saint Kitts and Nevis	Urticaria	2021	808.26	925.68	715.30

ASIR	Saint Kitts and Nevis	Urticaria	2021	1426.44	1618.11	1252.76
ASDR	Saint Kitts and Nevis	Urticaria	2021	48.56	68.35	31.40
ASPR	Saint Kitts and Nevis	Urticaria	1990	810.25	927.97	716.85
ASIR	Saint Kitts and Nevis	Urticaria	1990	1429.93	1621.79	1256.39
ASDR	Saint Kitts and Nevis	Urticaria	1990	48.61	69.14	32.47
ASPR	Saint Lucia	Urticaria	2021	807.05	924.39	714.20
ASIR	Saint Lucia	Urticaria	2021	1424.36	1615.70	1251.03
ASDR	Saint Lucia	Urticaria	2021	48.42	68.34	31.78
ASPR	Saint Lucia	Urticaria	1990	811.62	929.68	717.93
ASIR	Saint Lucia	Urticaria	1990	1432.29	1624.42	1258.36
ASDR	Saint Lucia	Urticaria	1990	48.69	69.44	31.57
ASPR	Saint Vincent and the Grenadines	Urticaria	2021	806.06	923.35	713.22
ASIR	Saint Vincent and the Grenadines	Urticaria	2021	1422.62	1613.92	1249.17
ASDR	Saint Vincent and the Grenadines	Urticaria	2021	48.30	69.09	31.47
ASPR	Saint Vincent and the Grenadines	Urticaria	1990	809.09	926.63	715.91
ASIR	Saint Vincent and the Grenadines	Urticaria	1990	1427.88	1619.57	1254.38
ASDR	Saint Vincent and the Grenadines	Urticaria	1990	48.57	69.28	32.18
ASPR	Samoa	Urticaria	2021	717.41	821.58	634.67
ASIR	Samoa	Urticaria	2021	1268.63	1449.44	1112.41
ASDR	Samoa	Urticaria	2021	43.11	61.50	28.49
ASPR	Samoa	Urticaria	1990	715.82	820.06	633.40
ASIR	Samoa	Urticaria	1990	1265.95	1445.74	1110.64
ASDR	Samoa	Urticaria	1990	43.07	61.80	28.09
ASPR	San Marino	Urticaria	2021	592.13	664.65	522.51
ASIR	San Marino	Urticaria	2021	1047.20	1166.68	932.09
ASDR	San Marino	Urticaria	2021	35.22	49.84	22.81
ASPR	San Marino	Urticaria	1990	586.80	658.14	518.25

ASIR	San Marino	Urticaria	1990	1038.09	1155.34	924.01
ASDR	San Marino	Urticaria	1990	34.97	49.36	22.44
ASPR	Sao Tome and Principe	Urticaria	2021	808.06	925.48	715.09
ASIR	Sao Tome and Principe	Urticaria	2021	1426.03	1617.63	1252.41
ASDR	Sao Tome and Principe	Urticaria	2021	48.58	69.01	32.33
ASPR	Sao Tome and Principe	Urticaria	1990	810.55	928.56	717.03
ASIR	Sao Tome and Principe	Urticaria	1990	1430.45	1622.29	1256.68
ASDR	Sao Tome and Principe	Urticaria	1990	48.68	69.90	31.94
ASPR	Saudi Arabia	Urticaria	2021	888.78	1016.72	780.02
ASIR	Saudi Arabia	Urticaria	2021	1568.85	1772.95	1384.68
ASDR	Saudi Arabia	Urticaria	2021	53.32	76.44	35.39
ASPR	Saudi Arabia	Urticaria	1990	891.33	1020.33	782.07
ASIR	Saudi Arabia	Urticaria	1990	1573.24	1778.78	1388.24
ASDR	Saudi Arabia	Urticaria	1990	53.39	74.93	35.34
ASPR	Senegal	Urticaria	2021	808.32	925.97	715.13
ASIR	Senegal	Urticaria	2021	1426.63	1618.30	1253.37
ASDR	Senegal	Urticaria	2021	48.27	68.16	31.87
ASPR	Senegal	Urticaria	1990	811.18	929.52	717.60
ASIR	Senegal	Urticaria	1990	1431.47	1623.43	1257.12
ASDR	Senegal	Urticaria	1990	48.32	68.89	31.89
ASPR	Serbia	Urticaria	2021	1081.36	1245.96	954.58
ASIR	Serbia	Urticaria	2021	1902.86	2166.04	1668.63
ASDR	Serbia	Urticaria	2021	65.41	93.56	43.33
ASPR	Serbia	Urticaria	1990	1089.60	1256.44	960.82
ASIR	Serbia	Urticaria	1990	1917.56	2185.14	1681.43
ASDR	Serbia	Urticaria	1990	65.85	93.91	43.20
ASPR	Seychelles	Urticaria	2021	753.25	859.50	655.08
ASIR	Seychelles	Urticaria	2021	1333.72	1529.38	1166.07
ASDR	Seychelles	Urticaria	2021	45.36	65.33	30.07
ASPR	Seychelles	Urticaria	1990	750.76	856.89	652.50
ASIR	Seychelles	Urticaria	1990	1329.37	1524.89	1162.23
ASDR	Seychelles	Urticaria	1990	45.35	65.57	29.60
ASPR	Sierra Leone	Urticaria	2021	808.67	926.39	715.59
ASIR	Sierra Leone	Urticaria	2021	1427.12	1618.85	1253.11
ASDR	Sierra Leone	Urticaria	2021	48.36	68.60	31.92
ASPR	Sierra Leone	Urticaria	1990	809.49	927.43	716.21
ASIR	Sierra Leone	Urticaria	1990	1428.59	1620.61	1254.48
ASDR	Sierra Leone	Urticaria	1990	48.14	69.15	31.75

ASPR	Singapore	Urticaria	2021	766.79	870.23	677.42
ASIR	Singapore	Urticaria	2021	1352.47	1532.98	1194.56
ASDR	Singapore	Urticaria	2021	46.49	66.46	30.67
ASPR	Singapore	Urticaria	1990	769.71	874.76	680.51
ASIR	Singapore	Urticaria	1990	1357.27	1535.70	1198.70
ASDR	Singapore	Urticaria	1990	46.49	66.24	30.07
ASPR	Slovakia	Urticaria	2021	1086.07	1251.75	958.49
ASIR	Slovakia	Urticaria	2021	1911.11	2175.63	1676.30
ASDR	Slovakia	Urticaria	2021	65.67	94.08	43.19
ASPR	Slovakia	Urticaria	1990	1091.43	1257.51	963.82
ASIR	Slovakia	Urticaria	1990	1920.53	2185.50	1685.15
ASDR	Slovakia	Urticaria	1990	65.98	94.01	43.90
ASPR	Slovenia	Urticaria	2021	1081.05	1246.56	953.72
ASIR	Slovenia	Urticaria	2021	1902.39	2166.21	1668.05
ASDR	Slovenia	Urticaria	2021	65.48	93.47	43.25
ASPR	Slovenia	Urticaria	1990	1090.74	1256.79	962.87
ASIR	Slovenia	Urticaria	1990	1919.17	2184.11	1684.06
ASDR	Slovenia	Urticaria	1990	65.87	93.40	43.50
ASPR	Solomon Islands	Urticaria	2021	717.27	821.40	634.52
ASIR	Solomon Islands	Urticaria	2021	1268.38	1448.82	1112.49
ASDR	Solomon Islands	Urticaria	2021	43.07	61.83	28.11
ASPR	Solomon Islands	Urticaria	1990	715.45	819.70	632.64
ASIR	Solomon Islands	Urticaria	1990	1265.16	1446.20	1108.72
ASDR	Solomon Islands	Urticaria	1990	42.95	61.48	28.39
ASPR	Somalia	Urticaria	2021	808.39	925.60	715.33
ASIR	Somalia	Urticaria	2021	1426.68	1618.37	1253.92
ASDR	Somalia	Urticaria	2021	48.24	68.49	32.13
ASPR	Somalia	Urticaria	1990	804.71	921.56	712.11
ASIR	Somalia	Urticaria	1990	1420.37	1611.71	1248.03
ASDR	Somalia	Urticaria	1990	47.94	68.49	31.82
ASPR	South Africa	Urticaria	2021	855.52	976.16	754.73
ASIR	South Africa	Urticaria	2021	1510.39	1712.99	1331.26
ASDR	South Africa	Urticaria	2021	50.92	72.36	33.73
ASPR	South Africa	Urticaria	1990	857.66	978.66	756.46
ASIR	South Africa	Urticaria	1990	1514.07	1717.06	1334.26
ASDR	South Africa	Urticaria	1990	51.21	72.91	33.63
ASPR	South Sudan	Urticaria	2021	807.91	925.73	714.45
ASIR	South Sudan	Urticaria	2021	1425.90	1617.59	1252.53

ASDR	South Sudan	Urticaria	2021	48.07	68.24	31.36
ASPR	South Sudan	Urticaria	1990	799.96	916.40	707.60
ASIR	South Sudan	Urticaria	1990	1411.93	1602.35	1239.95
ASDR	South Sudan	Urticaria	1990	47.45	67.32	31.37
ASPR	Spain	Urticaria	2021	544.38	603.46	492.50
ASIR	Spain	Urticaria	2021	967.66	1064.69	873.61
ASDR	Spain	Urticaria	2021	32.28	44.93	21.65
ASPR	Spain	Urticaria	1990	546.48	605.99	494.59
ASIR	Spain	Urticaria	1990	971.25	1068.56	876.54
ASDR	Spain	Urticaria	1990	32.51	44.84	21.93
ASPR	Sri Lanka	Urticaria	2021	786.63	895.19	687.62
ASIR	Sri Lanka	Urticaria	2021	1389.52	1572.26	1218.12
ASDR	Sri Lanka	Urticaria	2021	47.28	67.69	31.17
ASPR	Sri Lanka	Urticaria	1990	788.65	897.02	689.48
ASIR	Sri Lanka	Urticaria	1990	1392.87	1575.70	1221.36
ASDR	Sri Lanka	Urticaria	1990	47.37	67.70	30.63
ASPR	Sudan	Urticaria	2021	907.52	1035.84	797.01
ASIR	Sudan	Urticaria	2021	1601.31	1803.62	1415.05
ASDR	Sudan	Urticaria	2021	54.30	77.83	35.89
ASPR	Sudan	Urticaria	1990	909.06	1036.99	798.34
ASIR	Sudan	Urticaria	1990	1603.77	1806.09	1417.36
ASDR	Sudan	Urticaria	1990	54.11	76.87	36.22
ASPR	Suriname	Urticaria	2021	808.64	926.20	715.49
ASIR	Suriname	Urticaria	2021	1427.16	1618.94	1253.86
ASDR	Suriname	Urticaria	2021	48.38	69.36	31.70
ASPR	Suriname	Urticaria	1990	808.38	926.10	715.04
ASIR	Suriname	Urticaria	1990	1426.68	1618.17	1252.92
ASDR	Suriname	Urticaria	1990	48.49	68.43	31.59
ASPR	Sweden	Urticaria	2021	807.45	904.49	714.69
ASIR	Sweden	Urticaria	2021	1429.49	1596.98	1268.83
ASDR	Sweden	Urticaria	2021	48.13	67.34	31.68
ASPR	Sweden	Urticaria	1990	811.36	908.80	718.51
ASIR	Sweden	Urticaria	1990	1436.31	1604.45	1275.28
ASDR	Sweden	Urticaria	1990	48.34	67.77	31.99
ASPR	Switzerland	Urticaria	2021	582.70	653.67	514.65
ASIR	Switzerland	Urticaria	2021	1030.70	1148.01	917.60
ASDR	Switzerland	Urticaria	2021	34.58	48.20	22.65
ASPR	Switzerland	Urticaria	1990	584.57	655.51	516.27
ASIR	Switzerland	Urticaria	1990	1033.89	1151.73	920.71
ASDR	Switzerland	Urticaria	1990	34.69	48.82	22.95
ASPR	Syrian Arab Republic	Urticaria	2021	919.16	1047.35	807.32
ASIR	Syrian Arab Republic	Urticaria	2021	1621.48	1822.76	1433.36

ASDR	Syrian Arab Republic	Urticaria	2021	54.91	77.73	36.42
ASPR	Syrian Arab Republic	Urticaria	1990	907.37	1035.96	796.75
ASIR	Syrian Arab Republic	Urticaria	1990	1601.05	1804.61	1414.37
ASDR	Syrian Arab Republic	Urticaria	1990	54.40	77.88	36.01
ASPR	Taiwan (Province of China)	Urticaria	2021	724.99	826.77	638.00
ASIR	Taiwan (Province of China)	Urticaria	2021	1282.31	1453.58	1127.69
ASDR	Taiwan (Province of China)	Urticaria	2021	43.91	62.53	28.74
ASPR	Taiwan (Province of China)	Urticaria	1990	722.34	824.34	635.40
ASIR	Taiwan (Province of China)	Urticaria	1990	1277.51	1449.58	1122.62
ASDR	Taiwan (Province of China)	Urticaria	1990	43.81	63.27	28.68
ASPR	Tajikistan	Urticaria	2021	1047.24	1203.22	922.00
ASIR	Tajikistan	Urticaria	2021	1844.07	2111.03	1618.35
ASDR	Tajikistan	Urticaria	2021	63.22	89.87	41.91
ASPR	Tajikistan	Urticaria	1990	1051.17	1207.11	926.03
ASIR	Tajikistan	Urticaria	1990	1850.87	2119.46	1624.65
ASDR	Tajikistan	Urticaria	1990	63.33	89.98	41.99
ASPR	Thailand	Urticaria	2021	748.52	854.88	650.56
ASIR	Thailand	Urticaria	2021	1325.40	1521.36	1158.31
ASDR	Thailand	Urticaria	2021	45.20	65.17	29.87
ASPR	Thailand	Urticaria	1990	748.99	855.40	650.89
ASIR	Thailand	Urticaria	1990	1326.20	1522.06	1159.02
ASDR	Thailand	Urticaria	1990	45.06	64.46	29.37
ASPR	Timor-Leste	Urticaria	2021	749.85	856.24	652.14
ASIR	Timor-Leste	Urticaria	2021	1327.65	1523.53	1160.24
ASDR	Timor-Leste	Urticaria	2021	45.07	64.70	29.89
ASPR	Timor-Leste	Urticaria	1990	750.37	856.80	652.52
ASIR	Timor-Leste	Urticaria	1990	1328.61	1524.82	1161.54
ASDR	Timor-Leste	Urticaria	1990	44.80	64.51	29.42

ASPR	Togo	Urticaria	2021	811.76	929.75	717.77
ASIR	Togo	Urticaria	2021	1432.65	1624.81	1259.19
ASDR	Togo	Urticaria	2021	48.58	69.79	31.64
ASPR	Togo	Urticaria	1990	812.24	930.70	718.38
ASIR	Togo	Urticaria	1990	1433.41	1625.55	1259.45
ASDR	Togo	Urticaria	1990	48.38	68.78	32.11
ASPR	Tokelau	Urticaria	2021	716.94	821.02	634.39
ASIR	Tokelau	Urticaria	2021	1267.83	1447.62	1112.17
ASDR	Tokelau	Urticaria	2021	43.17	61.25	28.50
ASPR	Tokelau	Urticaria	1990	718.12	822.43	635.16
ASIR	Tokelau	Urticaria	1990	1269.91	1449.89	1114.80
ASDR	Tokelau	Urticaria	1990	43.29	62.75	28.58
ASPR	Tonga	Urticaria	2021	718.86	823.00	636.05
ASIR	Tonga	Urticaria	2021	1271.10	1451.10	1115.36
ASDR	Tonga	Urticaria	2021	43.25	61.93	28.59
ASPR	Tonga	Urticaria	1990	718.16	822.40	635.31
ASIR	Tonga	Urticaria	1990	1269.93	1450.21	1114.42
ASDR	Tonga	Urticaria	1990	43.35	61.29	28.88
ASPR	Trinidad and Tobago	Urticaria	2021	807.14	924.44	714.34
ASIR	Trinidad and Tobago	Urticaria	2021	1424.49	1615.98	1251.06
ASDR	Trinidad and Tobago	Urticaria	2021	48.41	68.78	31.65
ASPR	Trinidad and Tobago	Urticaria	1990	808.33	925.89	715.36
ASIR	Trinidad and Tobago	Urticaria	1990	1426.59	1618.16	1252.96
ASDR	Trinidad and Tobago	Urticaria	1990	48.53	68.98	32.11
ASPR	Tunisia	Urticaria	2021	909.39	1037.38	798.78
ASIR	Tunisia	Urticaria	2021	1604.50	1806.95	1417.90
ASDR	Tunisia	Urticaria	2021	54.54	77.58	36.34
ASPR	Tunisia	Urticaria	1990	909.07	1037.69	798.31
ASIR	Tunisia	Urticaria	1990	1603.97	1807.35	1417.10
ASDR	Tunisia	Urticaria	1990	54.66	78.52	36.12
ASPR	Turkey	Urticaria	2021	848.73	959.31	750.12
ASIR	Turkey	Urticaria	2021	1503.16	1687.63	1332.02
ASDR	Turkey	Urticaria	2021	50.93	72.15	33.85
ASPR	Turkey	Urticaria	1990	848.66	959.28	750.24
ASIR	Turkey	Urticaria	1990	1503.05	1687.67	1331.85
ASDR	Turkey	Urticaria	1990	50.97	72.00	33.86
ASPR	Turkmenistan	Urticaria	2021	1044.49	1199.78	919.92
ASIR	Turkmenistan	Urticaria	2021	1839.05	2106.61	1613.38

ASDR	Turkmenistan	Urticaria	2021	63.24	90.15	42.22
ASPR	Turkmenistan	Urticaria	1990	1054.58	1210.68	929.10
ASIR	Turkmenistan	Urticaria	1990	1856.77	2126.34	1630.30
ASDR	Turkmenistan	Urticaria	1990	63.39	89.21	42.08
ASPR	Tuvalu	Urticaria	2021	716.31	820.37	633.84
ASIR	Tuvalu	Urticaria	2021	1266.83	1446.82	1111.00
ASDR	Tuvalu	Urticaria	2021	43.18	61.34	28.02
ASPR	Tuvalu	Urticaria	1990	720.37	824.01	637.40
ASIR	Tuvalu	Urticaria	1990	1273.71	1452.99	1118.32
ASDR	Tuvalu	Urticaria	1990	43.30	61.31	28.63
ASPR	Uganda	Urticaria	2021	811.56	929.56	717.83
ASIR	Uganda	Urticaria	2021	1432.27	1624.38	1258.80
ASDR	Uganda	Urticaria	2021	48.61	69.13	31.94
ASPR	Uganda	Urticaria	1990	810.30	928.30	716.99
ASIR	Uganda	Urticaria	1990	1429.98	1621.82	1255.94
ASDR	Uganda	Urticaria	1990	48.06	67.62	31.87
ASPR	Ukraine	Urticaria	2021	1108.57	1275.05	974.14
ASIR	Ukraine	Urticaria	2021	1953.40	2234.31	1713.60
ASDR	Ukraine	Urticaria	2021	66.85	95.86	44.72
ASPR	Ukraine	Urticaria	1990	1113.37	1280.15	978.26
ASIR	Ukraine	Urticaria	1990	1961.78	2243.46	1721.39
ASDR	Ukraine	Urticaria	1990	67.03	95.74	44.35
ASPR	United Arab Emirates	Urticaria	2021	872.60	999.52	766.05
ASIR	United Arab Emirates	Urticaria	2021	1540.43	1746.39	1357.04
ASDR	United Arab Emirates	Urticaria	2021	52.51	75.29	35.03
ASPR	United Arab Emirates	Urticaria	1990	874.27	1001.97	767.20
ASIR	United Arab Emirates	Urticaria	1990	1543.46	1751.53	1360.37
ASDR	United Arab Emirates	Urticaria	1990	52.59	74.92	34.82
ASPR	United Kingdom	Urticaria	2021	656.58	732.42	579.02
ASIR	United Kingdom	Urticaria	2021	1162.25	1297.62	1029.35
ASDR	United Kingdom	Urticaria	2021	39.01	54.39	25.66
ASPR	United Kingdom	Urticaria	1990	654.69	730.47	577.51
ASIR	United Kingdom	Urticaria	1990	1158.75	1292.98	1026.83

ASDR	United Kingdom	Urticaria	1990	38.91	54.25	25.53
ASPR	United Republic of Tanzania	Urticaria	2021	811.61	929.87	717.96
ASIR	United Republic of Tanzania	Urticaria	2021	1432.31	1624.36	1258.26
ASDR	United Republic of Tanzania	Urticaria	2021	48.65	70.27	31.92
ASPR	United Republic of Tanzania	Urticaria	1990	811.59	929.89	718.03
ASIR	United Republic of Tanzania	Urticaria	1990	1432.26	1624.34	1258.15
ASDR	United Republic of Tanzania	Urticaria	1990	48.02	67.85	31.81
ASPR	United States of America	Urticaria	2021	904.23	954.77	858.27
ASIR	United States of America	Urticaria	2021	1597.53	1684.58	1511.32
ASDR	United States of America	Urticaria	2021	54.12	75.56	36.55
ASPR	United States of America	Urticaria	1990	884.06	940.64	831.91
ASIR	United States of America	Urticaria	1990	1561.30	1654.40	1461.93
ASDR	United States of America	Urticaria	1990	53.15	73.45	35.66
ASPR	United States Virgin Islands	Urticaria	2021	808.07	925.44	715.15
ASIR	United States Virgin Islands	Urticaria	2021	1426.19	1617.98	1253.16
ASDR	United States Virgin Islands	Urticaria	2021	48.55	69.21	31.86
ASPR	United States Virgin Islands	Urticaria	1990	811.47	929.63	717.71
ASIR	United States Virgin Islands	Urticaria	1990	1432.03	1624.14	1258.22
ASDR	United States Virgin Islands	Urticaria	1990	48.84	70.40	31.88

ASPR	Uruguay	Urticaria	2021	775.05	881.04	685.18
ASIR	Uruguay	Urticaria	2021	1366.73	1546.37	1207.45
ASDR	Uruguay	Urticaria	2021	46.61	66.02	30.80
ASPR	Uruguay	Urticaria	1990	774.65	880.44	684.71
ASIR	Uruguay	Urticaria	1990	1366.09	1545.70	1206.89
ASDR	Uruguay	Urticaria	1990	46.58	66.87	31.09
ASPR	Uzbekistan	Urticaria	2021	1050.40	1206.47	925.09
ASIR	Uzbekistan	Urticaria	2021	1849.49	2116.91	1623.55
ASDR	Uzbekistan	Urticaria	2021	63.33	89.67	41.71
ASPR	Uzbekistan	Urticaria	1990	1053.60	1209.63	928.27
ASIR	Uzbekistan	Urticaria	1990	1855.09	2124.59	1628.69
ASDR	Uzbekistan	Urticaria	1990	63.37	89.75	42.44
ASPR	Vanuatu	Urticaria	2021	718.33	822.53	635.46
ASIR	Vanuatu	Urticaria	2021	1270.26	1450.93	1114.24
ASDR	Vanuatu	Urticaria	2021	43.28	63.09	28.83
ASPR	Vanuatu	Urticaria	1990	716.58	820.85	633.78
ASIR	Vanuatu	Urticaria	1990	1267.13	1448.13	1110.57
ASDR	Vanuatu	Urticaria	1990	43.08	61.61	28.34
ASPR	Venezuela (Bolivarian Republic of)	Urticaria	2021	810.85	928.81	717.33
ASIR	Venezuela (Bolivarian Republic of)	Urticaria	2021	1430.97	1623.20	1257.27
ASDR	Venezuela (Bolivarian Republic of)	Urticaria	2021	48.82	69.19	32.06
ASPR	Venezuela (Bolivarian Republic of)	Urticaria	1990	808.82	926.45	715.68
ASIR	Venezuela (Bolivarian Republic of)	Urticaria	1990	1427.42	1619.12	1253.86
ASDR	Venezuela (Bolivarian Republic of)	Urticaria	1990	48.63	69.33	32.08
ASPR	Viet Nam	Urticaria	2021	749.83	856.13	651.71
ASIR	Viet Nam	Urticaria	2021	1327.77	1523.70	1160.65
ASDR	Viet Nam	Urticaria	2021	45.32	64.87	29.96
ASPR	Viet Nam	Urticaria	1990	747.47	853.82	649.45
ASIR	Viet Nam	Urticaria	1990	1323.58	1519.35	1156.39
ASDR	Viet Nam	Urticaria	1990	45.06	64.89	29.27
ASPR	Yemen	Urticaria	2021	909.92	1038.54	799.07
ASIR	Yemen	Urticaria	2021	1605.48	1808.83	1418.46

ASDR	Yemen	Urticaria	2021	54.00	76.49	35.55
ASPR	Yemen	Urticaria	1990	908.75	1036.93	798.08
ASIR	Yemen	Urticaria	1990	1603.51	1805.43	1416.95
ASDR	Yemen	Urticaria	1990	53.84	75.93	35.93
ASPR	Zambia	Urticaria	2021	809.62	927.30	716.33
ASIR	Zambia	Urticaria	2021	1428.83	1620.77	1254.84
ASDR	Zambia	Urticaria	2021	48.25	68.42	31.77
ASPR	Zambia	Urticaria	1990	809.74	927.92	716.57
ASIR	Zambia	Urticaria	1990	1429.03	1620.79	1254.71
ASDR	Zambia	Urticaria	1990	48.17	68.46	31.68
ASPR	Zimbabwe	Urticaria	2021	813.93	932.20	719.63
ASIR	Zimbabwe	Urticaria	2021	1436.37	1628.96	1262.41
ASDR	Zimbabwe	Urticaria	2021	48.73	69.76	31.81
ASPR	Zimbabwe	Urticaria	1990	810.99	929.15	717.38
ASIR	Zimbabwe	Urticaria	1990	1431.19	1623.15	1257.03
ASDR	Zimbabwe	Urticaria	1990	48.66	69.31	32.18

Supplemental Table 5: APC and AAPC values for ASIR, ASPR, and ASDR by stage from 1990 to 2021. (Average annual percentage change: AAPC; Average percentage change: APCAge-standardized disability-adjusted life year rates: ASDR; Age-standardized incidence rates: ASIR; Age-standardized prevalence rates: ASPR)

Diseases	Measure	Start to end year	APC (95% CI)	AAPC (95% CI)
**Atopic dermatitis**	ASIR	1990 to 1994	-0.21 (-0.22, -0.20)	-0.20 (-0.21, -0.20)
		1994 to 2001	-0.13 (-0.14, -0.12)	
		2001 to 2005	-0.20 (-0.21, -0.18)	
		2005 to 2011	-0.22 (-0.23, -0.21)	
		2011 to 2015	-0.30 (-0.32, -0.28)	
		2015 to 2018	-0.14 (-0.18, -0.11)	
		2018 to 2021	-0.25 (-0.27, -0.23)	
	ASPR	1990 to 1996	-0.31 (-0.32, -0.31)	-0.28 (-0.28, -0.28)
		1996 to 2001	-0.26 (-0.28, -0.26)	
		2001 to 2005	-0.24 (-0.26, -0.23)	
		2005 to 2011	-0.26 (-0.27, -0.26)	
		2011 to 2015	-0.35 (-0.36, -0.33)	
		2015 to 2018	-0.18 (-0.21, -0.14)	
		2018 to 2021	-0.33 (-0.35, -0.31)	
	ASDR	1990 to 1998	-0.30 (-0.31, -0.29)	-0.27 (-0.28, -0.26)
		1998 to 2011	-0.24 (-0.25, -0.24)	
		2011 to 2015	-0.34 (-0.37, -0.31)	
		2015 to 2018	-0.15 (-0.21, -0.10)	
		2018 to 2021	-0.36 (-0.38, -0.33)	
**Urticaria**	ASIR	1990 to 1994	-0.01 (-0.02, -0.00)	0.01 (0.01, 0.01)

		1994 to 2005	0.03 (0.03, 0.03)	
		2005 to 2013	0.01 (0.01, 0.02)	
		2013 to 2018	-0.04 (-0.05, -0.03)	
		2018 to 2021	0.03 (0.02, 0.04)	
	ASPR	1990 to 1993	-0.01 (-0.02, -0.00)	0.01 (0.01, 0.01)
		1993 to 1997	0.02 (0.01, 0.02)	
		1997 to 2005	0.04 (0.03, 0.04)	
		2005 to 2013	0.01 (0.01, 0.02)	
		2013 to 2019	-0.03 (-0.04, -0.03)	
		2019 to 2021	0.03 (0.01, 0.05)	
	ASDR	1990 to 1996	0.01 (0.01, 0.02)	0.02 (0.01, 0.02)
		1996 to 2007	0.04 (0.04, 0.04)	
		2007 to 2013	0.02 (0.02, 0.03)	
		2013 to 2021	-0.03 (-0.03, -0.02)	
**Contact dermatitis**	ASIR	1990 to 1994	-0.52 (-0.54, -0.50)	-0.02 (-0.02, -0.02)
		1994 to 2015	0.06 (0.06, 0.06)	
		2015 to 2021	0.02 (0.01, 0.03)	
	ASPR	1990 to 1994	-0.67 (-0.71, -0.64)	-0.05 (-0.06, -0.04)
		1994 to 2021	0.04 (0.04, 0.04)	
	ASDR	1990 to 1993	-0.78 (-0.82, -0.74)	-0.06 (-0.07, -0.05)
		1993 to 1996	-0.11 (-0.20, -0.03)	
		1996 to 2016	0.05 (0.05, 0.06)	
		2016 to 2021	-0.03 (-0.05, -0.01)	

Supplemental Table 6: Predicted values of the ARIMA model for the next 15 years.

Disease	Category	Year	Value	Type
AD	DALYs	1990	82.13008	Actual
AD	DALYs	1991	81.87106	Actual
AD	DALYs	1992	81.61701	Actual
AD	DALYs	1993	81.3601	Actual
AD	DALYs	1994	81.11021	Actual
AD	DALYs	1995	80.87245	Actual
AD	DALYs	1996	80.6429	Actual
AD	DALYs	1997	80.41011	Actual
AD	DALYs	1998	80.19387	Actual
AD	DALYs	1999	79.97555	Actual
AD	DALYs	2000	79.76216	Actual
AD	DALYs	2001	79.55756	Actual
AD	DALYs	2002	79.36901	Actual
AD	DALYs	2003	79.18943	Actual
AD	DALYs	2004	79.02284	Actual
AD	DALYs	2005	78.83225	Actual
AD	DALYs	2006	78.6273	Actual
AD	DALYs	2007	78.4304	Actual
AD	DALYs	2008	78.2397	Actual
AD	DALYs	2009	78.05862	Actual
AD	DALYs	2010	77.86432	Actual
AD	DALYs	2011	77.65859	Actual
AD	DALYs	2012	77.4061	Actual
AD	DALYs	2013	77.13368	Actual
AD	DALYs	2014	76.86036	Actual
AD	DALYs	2015	76.64506	Actual
AD	DALYs	2016	76.49588	Actual
AD	DALYs	2017	76.37741	Actual
AD	DALYs	2018	76.25191	Actual
AD	DALYs	2019	76.0504	Actual
AD	DALYs	2020	75.71007	Actual
AD	DALYs	2021	75.45247	Actual
AD	DALYs	2022	75.28387	Forecast
AD	DALYs	2023	75.10222	Forecast
AD	DALYs	2024	74.88806	Forecast
AD	DALYs	2025	74.67391	Forecast
AD	DALYs	2026	74.45976	Forecast
AD	DALYs	2027	74.24561	Forecast
AD	DALYs	2028	74.03145	Forecast
AD	DALYs	2029	73.8173	Forecast
AD	DALYs	2030	73.60315	Forecast
AD	DALYs	2031	73.38899	Forecast

AD	DALYs	2032	73.17484	Forecast
AD	DALYs	2033	72.96069	Forecast
AD	DALYs	2034	72.74654	Forecast
AD	DALYs	2035	72.53238	Forecast
AD	DALYs	2036	72.31823	Forecast
AD	ASIR	1990	234.7812	Actual
AD	ASIR	1991	234.2405	Actual
AD	ASIR	1992	233.7327	Actual
AD	ASIR	1993	233.256	Actual
AD	ASIR	1994	232.8401	Actual
AD	ASIR	1995	232.4752	Actual
AD	ASIR	1996	232.1256	Actual
AD	ASIR	1997	231.8202	Actual
AD	ASIR	1998	231.5571	Actual
AD	ASIR	1999	231.2903	Actual
AD	ASIR	2000	230.9849	Actual
AD	ASIR	2001	230.6287	Actual
AD	ASIR	2002	230.2143	Actual
AD	ASIR	2003	229.7577	Actual
AD	ASIR	2004	229.2992	Actual
AD	ASIR	2005	228.8286	Actual
AD	ASIR	2006	228.3289	Actual
AD	ASIR	2007	227.8378	Actual
AD	ASIR	2008	227.3579	Actual
AD	ASIR	2009	226.864	Actual
AD	ASIR	2010	226.3732	Actual
AD	ASIR	2011	225.8386	Actual
AD	ASIR	2012	225.216	Actual
AD	ASIR	2013	224.519	Actual
AD	ASIR	2014	223.8328	Actual
AD	ASIR	2015	223.2517	Actual
AD	ASIR	2016	222.8279	Actual
AD	ASIR	2017	222.5324	Actual
AD	ASIR	2018	222.2324	Actual
AD	ASIR	2019	221.7726	Actual
AD	ASIR	2020	221.0978	Actual
AD	ASIR	2021	220.5803	Actual
AD	ASIR	2022	220.1813	Forecast
AD	ASIR	2023	219.8337	Forecast
AD	ASIR	2024	219.4782	Forecast
AD	ASIR	2025	219.0822	Forecast
AD	ASIR	2026	218.6411	Forecast
AD	ASIR	2027	218.1689	Forecast
AD	ASIR	2028	217.6856	Forecast

AD	ASIR	2029	217.2072	Forecast
AD	ASIR	2030	216.7415	Forecast
AD	ASIR	2031	216.2885	Forecast
AD	ASIR	2032	215.8434	Forecast
AD	ASIR	2033	215.4005	Forecast
AD	ASIR	2034	214.9556	Forecast
AD	ASIR	2035	214.5067	Forecast
AD	ASIR	2036	214.0544	Forecast
AD	ASPR	1990	1885.428	Actual
AD	ASPR	1991	1879.378	Actual
AD	ASPR	1992	1873.319	Actual
AD	ASPR	1993	1867.234	Actual
AD	ASPR	1994	1861.432	Actual
AD	ASPR	1995	1855.91	Actual
AD	ASPR	1996	1850.373	Actual
AD	ASPR	1997	1845.132	Actual
AD	ASPR	1998	1840.181	Actual
AD	ASPR	1999	1835.345	Actual
AD	ASPR	2000	1830.464	Actual
AD	ASPR	2001	1825.664	Actual
AD	ASPR	2002	1821.171	Actual
AD	ASPR	2003	1816.757	Actual
AD	ASPR	2004	1812.405	Actual
AD	ASPR	2005	1807.959	Actual
AD	ASPR	2006	1803.189	Actual
AD	ASPR	2007	1798.381	Actual
AD	ASPR	2008	1793.677	Actual
AD	ASPR	2009	1789.075	Actual
AD	ASPR	2010	1784.626	Actual
AD	ASPR	2011	1779.538	Actual
AD	ASPR	2012	1773.478	Actual
AD	ASPR	2013	1766.999	Actual
AD	ASPR	2014	1760.783	Actual
AD	ASPR	2015	1755.586	Actual
AD	ASPR	2016	1751.822	Actual
AD	ASPR	2017	1748.851	Actual
AD	ASPR	2018	1745.482	Actual
AD	ASPR	2019	1740.684	Actual
AD	ASPR	2020	1734.002	Actual
AD	ASPR	2021	1728.507	Actual
AD	ASPR	2022	1724.121	Forecast
AD	ASPR	2023	1720.196	Forecast
AD	ASPR	2024	1716.029	Forecast
AD	ASPR	2025	1711.252	Forecast

AD	ASPR	2026	1705.932	Forecast
AD	ASPR	2027	1700.405	Forecast
AD	ASPR	2028	1695.02	Forecast
AD	ASPR	2029	1689.947	Forecast
AD	ASPR	2030	1685.14	Forecast
AD	ASPR	2031	1680.423	Forecast
AD	ASPR	2032	1675.626	Forecast
AD	ASPR	2033	1670.669	Forecast
AD	ASPR	2034	1665.583	Forecast
AD	ASPR	2035	1660.458	Forecast
AD	ASPR	2036	1655.379	Forecast
CD	ASDR	1990	27.72687	Actual
CD	ASDR	1991	27.49658	Actual
CD	ASDR	1992	27.28113	Actual
CD	ASDR	1993	27.11471	Actual
CD	ASDR	1994	27.01199	Actual
CD	ASDR	1995	26.98433	Actual
CD	ASDR	1996	26.99594	Actual
CD	ASDR	1997	27.00531	Actual
CD	ASDR	1998	27.0176	Actual
CD	ASDR	1999	27.03255	Actual
CD	ASDR	2000	27.03938	Actual
CD	ASDR	2001	27.05606	Actual
CD	ASDR	2002	27.06681	Actual
CD	ASDR	2003	27.08166	Actual
CD	ASDR	2004	27.09587	Actual
CD	ASDR	2005	27.10927	Actual
CD	ASDR	2006	27.1239	Actual
CD	ASDR	2007	27.14231	Actual
CD	ASDR	2008	27.1581	Actual
CD	ASDR	2009	27.18142	Actual
CD	ASDR	2010	27.19935	Actual
CD	ASDR	2011	27.20902	Actual
CD	ASDR	2012	27.22001	Actual
CD	ASDR	2013	27.23325	Actual
CD	ASDR	2014	27.24074	Actual
CD	ASDR	2015	27.25002	Actual
CD	ASDR	2016	27.25552	Actual
CD	ASDR	2017	27.25572	Actual
CD	ASDR	2018	27.264	Actual
CD	ASDR	2019	27.26357	Actual
CD	ASDR	2020	27.23491	Actual
CD	ASDR	2021	27.21736	Actual
CD	ASDR	2022	27.21165	Forecast

CD	ASDR	2023	27.2173	Forecast
CD	ASDR	2024	27.23275	Forecast
CD	ASDR	2025	27.25564	Forecast
CD	ASDR	2026	27.28308	Forecast
CD	ASDR	2027	27.31199	Forecast
CD	ASDR	2028	27.33942	Forecast
CD	ASDR	2029	27.36281	Forecast
CD	ASDR	2030	27.38022	Forecast
CD	ASDR	2031	27.39046	Forecast
CD	ASDR	2032	27.39317	Forecast
CD	ASDR	2033	27.38871	Forecast
CD	ASDR	2034	27.37814	Forecast
CD	ASDR	2035	27.36302	Forecast
CD	ASDR	2036	27.34522	Forecast
CD	ASIR	1990	3045.092	Actual
CD	ASIR	1991	3024.564	Actual
CD	ASIR	1992	3005.981	Actual
CD	ASIR	1993	2991.311	Actual
CD	ASIR	1994	2982.422	Actual
CD	ASIR	1995	2980.414	Actual
CD	ASIR	1996	2982.619	Actual
CD	ASIR	1997	2984.547	Actual
CD	ASIR	1998	2986.409	Actual
CD	ASIR	1999	2988.364	Actual
CD	ASIR	2000	2990.01	Actual
CD	ASIR	2001	2991.784	Actual
CD	ASIR	2002	2993.401	Actual
CD	ASIR	2003	2995.059	Actual
CD	ASIR	2004	2996.744	Actual
CD	ASIR	2005	2998.237	Actual
CD	ASIR	2006	3000.059	Actual
CD	ASIR	2007	3001.844	Actual
CD	ASIR	2008	3003.722	Actual
CD	ASIR	2009	3005.783	Actual
CD	ASIR	2010	3007.612	Actual
CD	ASIR	2011	3009.434	Actual
CD	ASIR	2012	3011.155	Actual
CD	ASIR	2013	3012.87	Actual
CD	ASIR	2014	3014.419	Actual
CD	ASIR	2015	3015.695	Actual
CD	ASIR	2016	3016.921	Actual
CD	ASIR	2017	3017.918	Actual
CD	ASIR	2018	3018.771	Actual
CD	ASIR	2019	3019.513	Actual

CD	ASIR	2020	3019.986	Actual
CD	ASIR	2021	3020.33	Actual
CD	ASIR	2022	3020.622	Forecast
CD	ASIR	2023	3020.878	Forecast
CD	ASIR	2024	3021.112	Forecast
CD	ASIR	2025	3021.331	Forecast
CD	ASIR	2026	3021.539	Forecast
CD	ASIR	2027	3021.741	Forecast
CD	ASIR	2028	3021.938	Forecast
CD	ASIR	2029	3022.133	Forecast
CD	ASIR	2030	3022.326	Forecast
CD	ASIR	2031	3022.518	Forecast
CD	ASIR	2032	3022.708	Forecast
CD	ASIR	2033	3022.899	Forecast
CD	ASIR	2034	3023.088	Forecast
CD	ASIR	2035	3023.278	Forecast
CD	ASIR	2036	3023.467	Forecast
CD	ASPR	1990	1123.848	Actual
CD	ASPR	1991	1114.046	Actual
CD	ASPR	1992	1105.161	Actual
CD	ASPR	1993	1098.076	Actual
CD	ASPR	1994	1093.622	Actual
CD	ASPR	1995	1092.361	Actual
CD	ASPR	1996	1093.014	Actual
CD	ASPR	1997	1093.546	Actual
CD	ASPR	1998	1094.042	Actual
CD	ASPR	1999	1094.575	Actual
CD	ASPR	2000	1095.004	Actual
CD	ASPR	2001	1095.479	Actual
CD	ASPR	2002	1095.903	Actual
CD	ASPR	2003	1096.347	Actual
CD	ASPR	2004	1096.808	Actual
CD	ASPR	2005	1097.215	Actual
CD	ASPR	2006	1097.744	Actual
CD	ASPR	2007	1098.271	Actual
CD	ASPR	2008	1098.844	Actual
CD	ASPR	2009	1099.488	Actual
CD	ASPR	2010	1100.048	Actual
CD	ASPR	2011	1100.597	Actual
CD	ASPR	2012	1101.129	Actual
CD	ASPR	2013	1101.666	Actual
CD	ASPR	2014	1102.134	Actual
CD	ASPR	2015	1102.505	Actual
CD	ASPR	2016	1102.844	Actual

CD	ASPR	2017	1103.103	Actual
CD	ASPR	2018	1103.306	Actual
CD	ASPR	2019	1103.465	Actual
CD	ASPR	2020	1103.54	Actual
CD	ASPR	2021	1103.565	Actual
CD	ASPR	2022	1103.741	Forecast
CD	ASPR	2023	1103.911	Forecast
CD	ASPR	2024	1104.075	Forecast
CD	ASPR	2025	1104.234	Forecast
CD	ASPR	2026	1104.388	Forecast
CD	ASPR	2027	1104.536	Forecast
CD	ASPR	2028	1104.679	Forecast
CD	ASPR	2029	1104.818	Forecast
CD	ASPR	2030	1104.952	Forecast
CD	ASPR	2031	1105.081	Forecast
CD	ASPR	2032	1105.206	Forecast
CD	ASPR	2033	1105.327	Forecast
CD	ASPR	2034	1105.443	Forecast
CD	ASPR	2035	1105.556	Forecast
CD	ASPR	2036	1105.665	Forecast
Urticaria	ASDR	1990	51.86257	Actual
Urticaria	ASDR	1991	51.85844	Actual
Urticaria	ASDR	1992	51.86673	Actual
Urticaria	ASDR	1993	51.86704	Actual
Urticaria	ASDR	1994	51.87616	Actual
Urticaria	ASDR	1995	51.88458	Actual
Urticaria	ASDR	1996	51.8997	Actual
Urticaria	ASDR	1997	51.91244	Actual
Urticaria	ASDR	1998	51.93742	Actual
Urticaria	ASDR	1999	51.95392	Actual
Urticaria	ASDR	2000	51.97882	Actual
Urticaria	ASDR	2001	52.0056	Actual
Urticaria	ASDR	2002	52.03623	Actual
Urticaria	ASDR	2003	52.05036	Actual
Urticaria	ASDR	2004	52.07009	Actual
Urticaria	ASDR	2005	52.08867	Actual
Urticaria	ASDR	2006	52.11503	Actual
Urticaria	ASDR	2007	52.13812	Actual
Urticaria	ASDR	2008	52.14988	Actual
Urticaria	ASDR	2009	52.16156	Actual
Urticaria	ASDR	2010	52.17255	Actual
Urticaria	ASDR	2011	52.18994	Actual
Urticaria	ASDR	2012	52.20754	Actual
Urticaria	ASDR	2013	52.20839	Actual

Urticaria	ASDR	2014	52.20541	Actual
Urticaria	ASDR	2015	52.19821	Actual
Urticaria	ASDR	2016	52.1844	Actual
Urticaria	ASDR	2017	52.16036	Actual
Urticaria	ASDR	2018	52.14303	Actual
Urticaria	ASDR	2019	52.14525	Actual
Urticaria	ASDR	2020	52.11369	Actual
Urticaria	ASDR	2021	52.11301	Actual
Urticaria	ASDR	2022	52.09315	Forecast
Urticaria	ASDR	2023	52.08521	Forecast
Urticaria	ASDR	2024	52.06986	Forecast
Urticaria	ASDR	2025	52.05911	Forecast
Urticaria	ASDR	2026	52.0455	Forecast
Urticaria	ASDR	2027	52.03367	Forecast
Urticaria	ASDR	2028	52.02074	Forecast
Urticaria	ASDR	2029	52.00849	Forecast
Urticaria	ASDR	2030	51.99581	Forecast
Urticaria	ASDR	2031	51.9834	Forecast
Urticaria	ASDR	2032	51.97083	Forecast
Urticaria	ASDR	2033	51.95835	Forecast
Urticaria	ASDR	2034	51.94582	Forecast
Urticaria	ASDR	2035	51.93332	Forecast
Urticaria	ASDR	2036	51.9208	Forecast
Urticaria	ASIR	1990	1529.244	Actual
Urticaria	ASIR	1991	1528.968	Actual
Urticaria	ASIR	1992	1528.779	Actual
Urticaria	ASIR	1993	1528.704	Actual
Urticaria	ASIR	1994	1528.806	Actual
Urticaria	ASIR	1995	1529.061	Actual
Urticaria	ASIR	1996	1529.367	Actual
Urticaria	ASIR	1997	1529.747	Actual
Urticaria	ASIR	1998	1530.204	Actual
Urticaria	ASIR	1999	1530.761	Actual
Urticaria	ASIR	2000	1531.361	Actual
Urticaria	ASIR	2001	1531.896	Actual
Urticaria	ASIR	2002	1532.357	Actual
Urticaria	ASIR	2003	1532.807	Actual
Urticaria	ASIR	2004	1533.257	Actual
Urticaria	ASIR	2005	1533.668	Actual
Urticaria	ASIR	2006	1533.996	Actual
Urticaria	ASIR	2007	1534.203	Actual
Urticaria	ASIR	2008	1534.282	Actual
Urticaria	ASIR	2009	1534.346	Actual
Urticaria	ASIR	2010	1534.607	Actual

Urticaria	ASIR	2011	1535.053	Actual
Urticaria	ASIR	2012	1535.253	Actual
Urticaria	ASIR	2013	1535.123	Actual
Urticaria	ASIR	2014	1534.844	Actual
Urticaria	ASIR	2015	1534.29	Actual
Urticaria	ASIR	2016	1533.495	Actual
Urticaria	ASIR	2017	1532.827	Actual
Urticaria	ASIR	2018	1532.531	Actual
Urticaria	ASIR	2019	1532.589	Actual
Urticaria	ASIR	2020	1532.98	Actual
Urticaria	ASIR	2021	1533.71	Actual
Urticaria	ASIR	2022	1534.633	Forecast
Urticaria	ASIR	2023	1535.516	Forecast
Urticaria	ASIR	2024	1536.221	Forecast
Urticaria	ASIR	2025	1536.688	Forecast
Urticaria	ASIR	2026	1536.922	Forecast
Urticaria	ASIR	2027	1536.967	Forecast
Urticaria	ASIR	2028	1536.884	Forecast
Urticaria	ASIR	2029	1536.734	Forecast
Urticaria	ASIR	2030	1536.568	Forecast
Urticaria	ASIR	2031	1536.42	Forecast
Urticaria	ASIR	2032	1536.309	Forecast
Urticaria	ASIR	2033	1536.242	Forecast
Urticaria	ASIR	2034	1536.215	Forecast
Urticaria	ASIR	2035	1536.217	Forecast
Urticaria	ASIR	2036	1536.237	Forecast
Urticaria	ASPR	1990	1529.244	Actual
Urticaria	ASPR	1991	1528.968	Actual
Urticaria	ASPR	1992	1528.779	Actual
Urticaria	ASPR	1993	1528.704	Actual
Urticaria	ASPR	1994	1528.806	Actual
Urticaria	ASPR	1995	1529.061	Actual
Urticaria	ASPR	1996	1529.367	Actual
Urticaria	ASPR	1997	1529.747	Actual
Urticaria	ASPR	1998	1530.204	Actual
Urticaria	ASPR	1999	1530.761	Actual
Urticaria	ASPR	2000	1531.361	Actual
Urticaria	ASPR	2001	1531.896	Actual
Urticaria	ASPR	2002	1532.357	Actual
Urticaria	ASPR	2003	1532.807	Actual
Urticaria	ASPR	2004	1533.257	Actual
Urticaria	ASPR	2005	1533.668	Actual
Urticaria	ASPR	2006	1533.996	Actual
Urticaria	ASPR	2007	1534.203	Actual

Urticaria	ASPR	2008	1534.282	Actual
Urticaria	ASPR	2009	1534.346	Actual
Urticaria	ASPR	2010	1534.607	Actual
Urticaria	ASPR	2011	1535.053	Actual
Urticaria	ASPR	2012	1535.253	Actual
Urticaria	ASPR	2013	1535.123	Actual
Urticaria	ASPR	2014	1534.844	Actual
Urticaria	ASPR	2015	1534.29	Actual
Urticaria	ASPR	2016	1533.495	Actual
Urticaria	ASPR	2017	1532.827	Actual
Urticaria	ASPR	2018	1532.531	Actual
Urticaria	ASPR	2019	1532.589	Actual
Urticaria	ASPR	2020	1532.98	Actual
Urticaria	ASPR	2021	1533.71	Actual
Urticaria	ASPR	2022	1534.633	Forecast
Urticaria	ASPR	2023	1535.516	Forecast
Urticaria	ASPR	2024	1536.221	Forecast
Urticaria	ASPR	2025	1536.688	Forecast
Urticaria	ASPR	2026	1536.922	Forecast
Urticaria	ASPR	2027	1536.967	Forecast
Urticaria	ASPR	2028	1536.884	Forecast
Urticaria	ASPR	2029	1536.734	Forecast
Urticaria	ASPR	2030	1536.568	Forecast
Urticaria	ASPR	2031	1536.42	Forecast
Urticaria	ASPR	2032	1536.309	Forecast
Urticaria	ASPR	2033	1536.242	Forecast
Urticaria	ASPR	2034	1536.215	Forecast
Urticaria	ASPR	2035	1536.217	Forecast
Urticaria	ASPR	2036	1536.237	Forecast

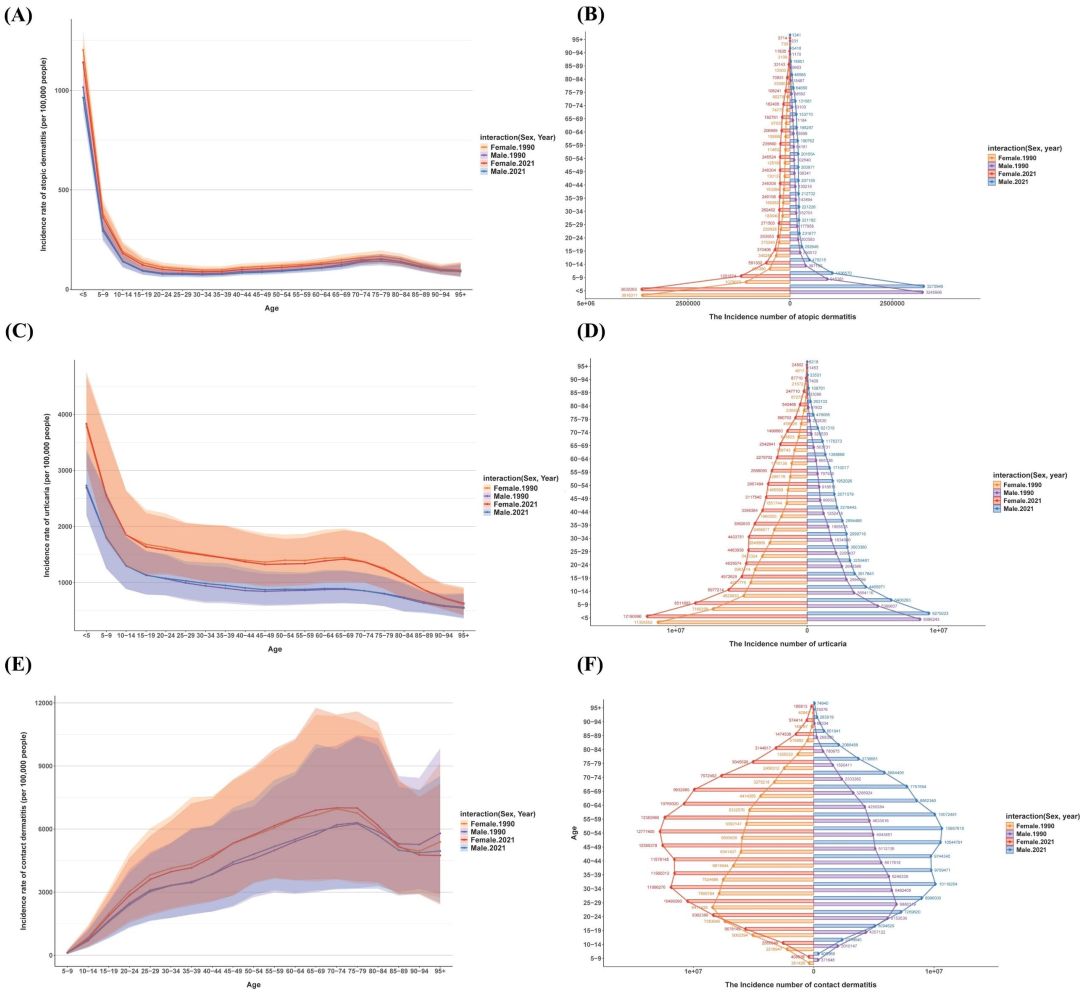

Supplemental Figure 1: The changing trends of AD, urticaria, and CD in different genders and age groups from 1990 to 2021. (A) Incidence rate of AD; (B) Incidence number of AD; (C) Incidence rate of urticaria; (D) Incidence number of urticaria; (E) Incidence rate of CD; (F) Incidence number of CD.

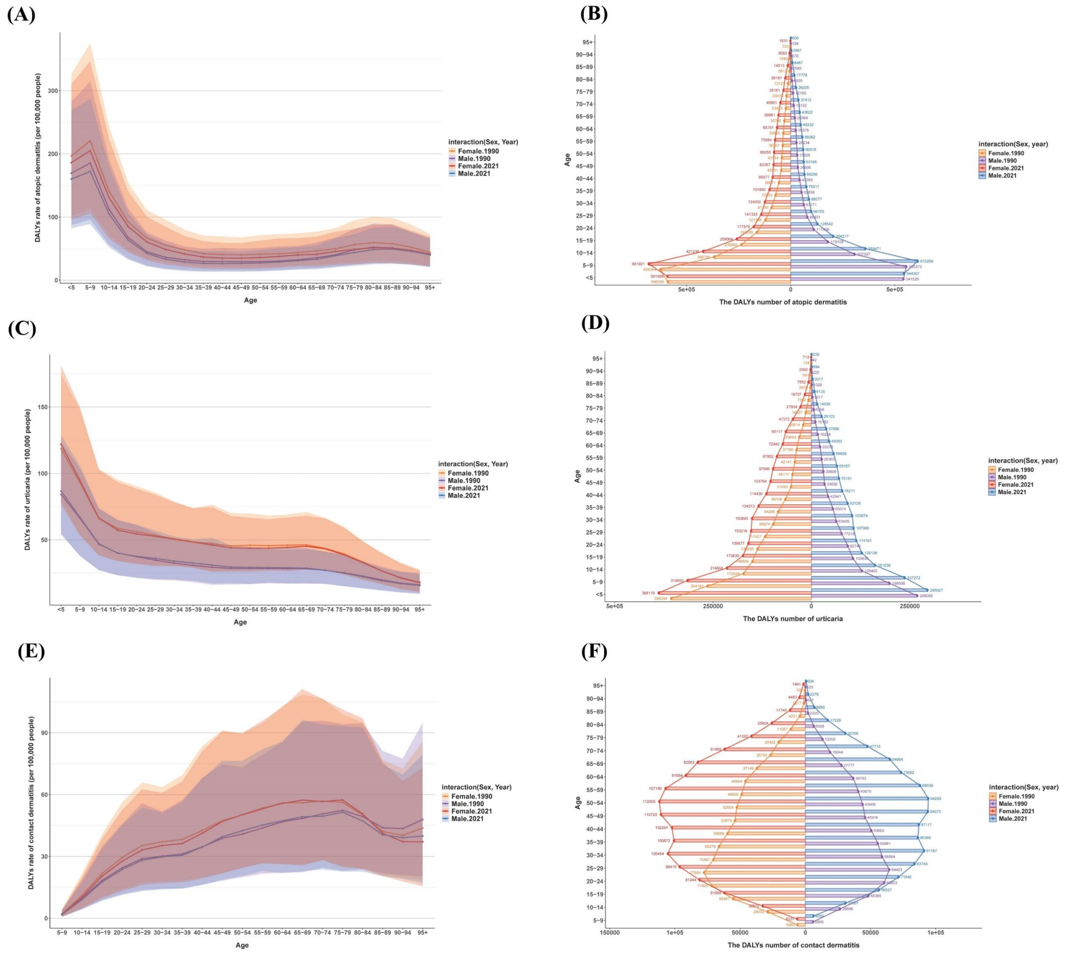

Supplemental Figure 2: Trends in DALYs rates and numbers for AD, urticaria, and CD by age and gender in 1990 and 2021. (A) DALYs rate of AD; (B) DALYs number of AD; (C) DALYs rate of urticaria; (D) DALYs number of urticaria; (E) DALYs rate of CD; (F) DALYs number of CD.

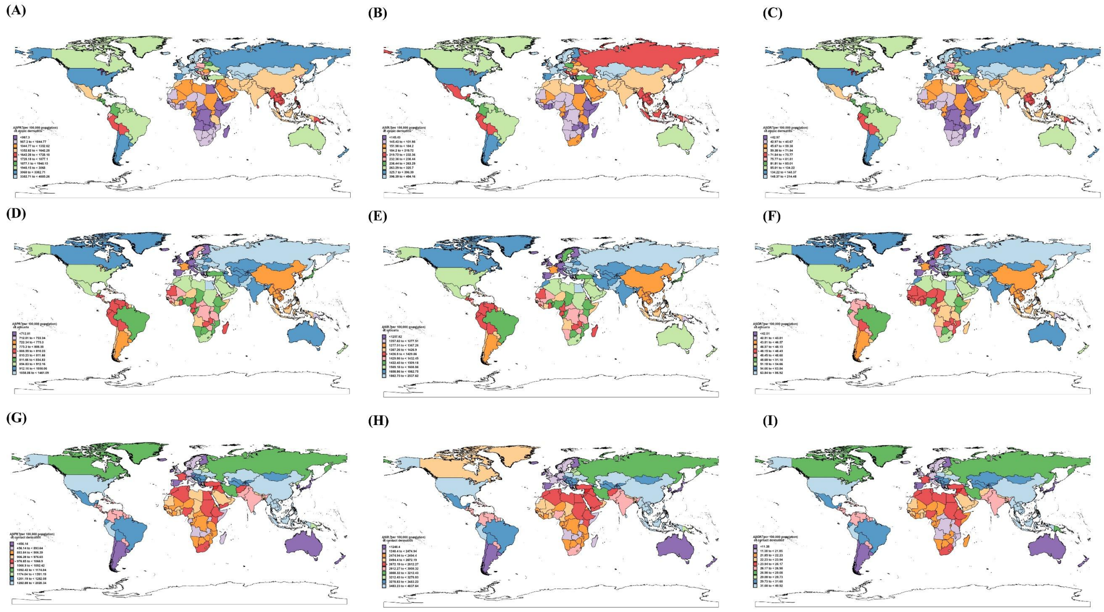

Supplemental Figure 3: Global distribution of 1990 ASPR, ASIR, and ASDR for allergic skin diseases. (A-C) AD; (D-F) Urticaria; (G-I) CD. White regions indicate missing data.

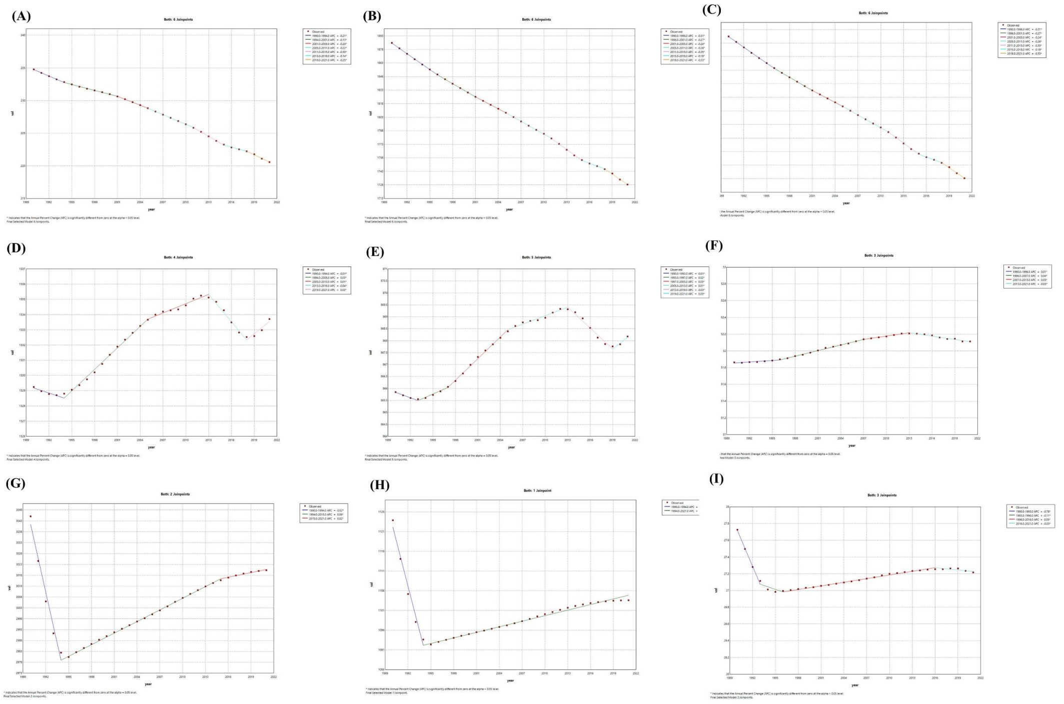

Supplemental Figure 4: Joinpoint regression analysis for AD (A to C), urticaria (D to F), and CD (G to I) from 1990 to 2021. The figure shows the best-fit joinpoint regression curves. Statistical differences between segments are indicated (*P*<0.05).

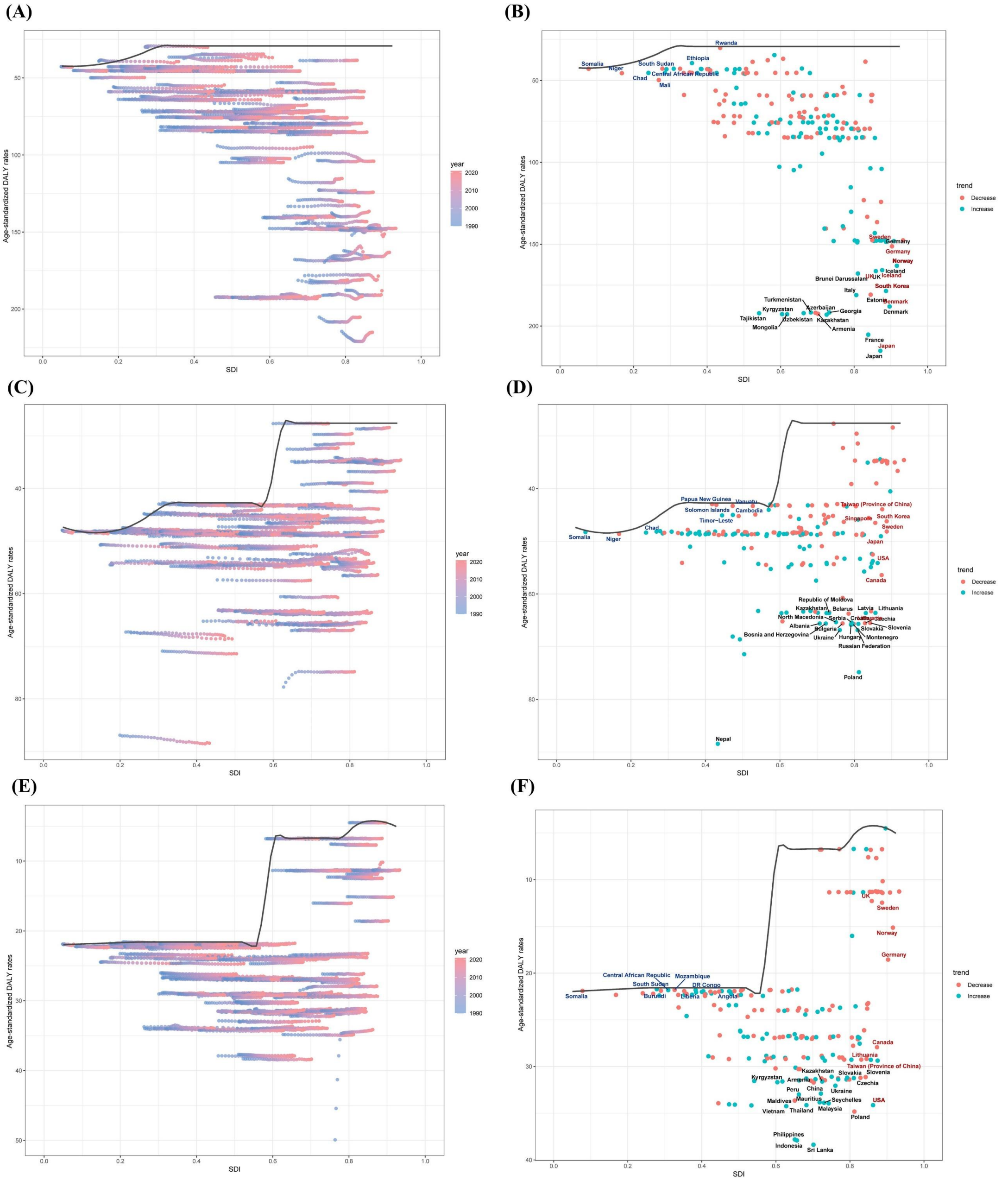

Supplemental Figure 5: Frontier analysis of SDI and DALYs rates (1990 to 2021). Black lines show frontiers; the top 20 regions with largest differences are in black. Low-SDI, low-difference regions are blue; high-SDI, high-difference regions are red. AD (A, B), urticaria (C, D), and CD (E, F).

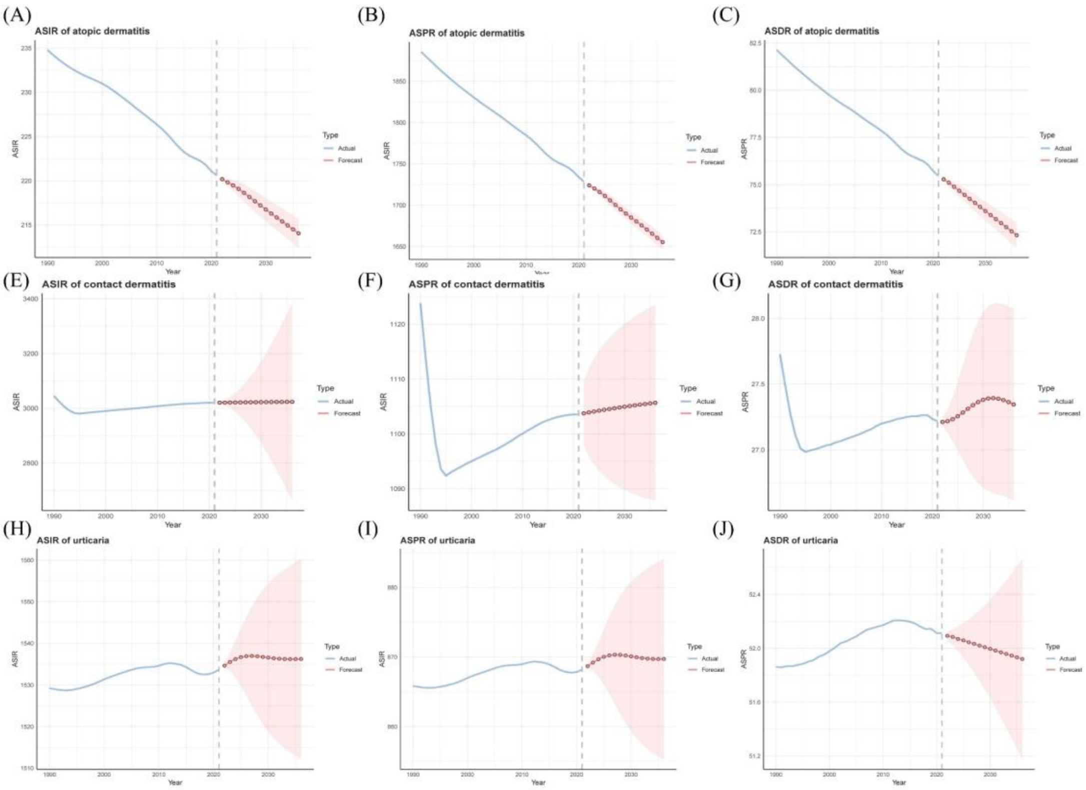

Supplemental Figure 6: ARIMA prediction models of allergic-related skin diseases. (A-C) ARIMA prediction models for ASIR, ASPR, ASDR for AD. (E-G) ARIMA prediction models for ASIR, ASPR, and ASDR for CD. (H-J) ARIMA prediction models for ASIR, ASPR, and ASDR for urticaria.

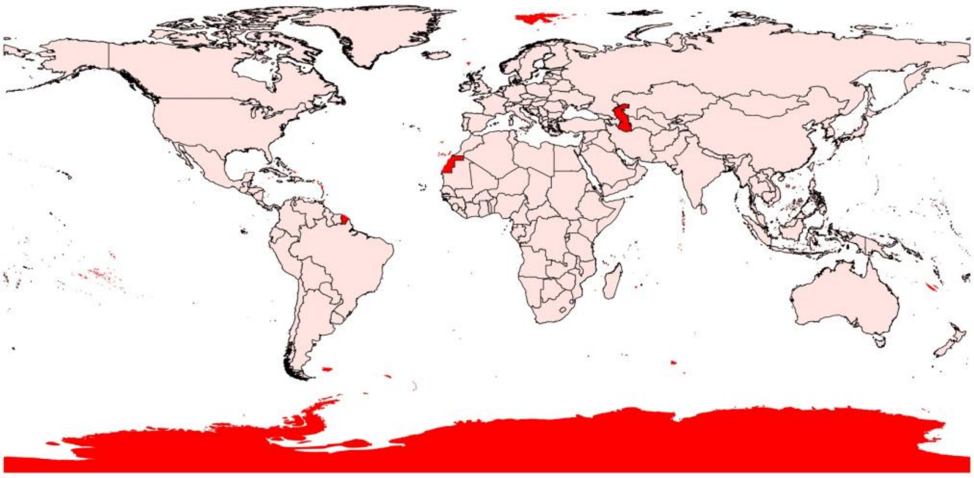

Supplemental Figure 7: Countries/regions missing in the analysis (Deep Red Areas).
